# Supplementary material for: Menopausal hormone therapy and women’s health: An umbrella review
Source: PLoS Med. 2021 Aug 2;18(8):e1003731. doi: 10.1371/journal.pmed.1003731 (PMC8366967; doi:10.1371/journal.pmed.1003731)
Supplement: S4 Text — (PDF) [file pmed.1003731.s005.pdf]

Meta-Analysis of Randomized Controlled Trials of Menopausal Hormone Therapy and Multiple Health Outcomes

1. Location of Datasets and Files

```
data.dir <- "/Users/xzhagu/Library/Mobile Documents/com~apple~CloudDocs/Documents/MHT and Women's Health_umbrella review/MHT umbrella review project_R/RCTs/Primary Prevention"
file.dir <- "/Users/xzhagu/Library/Mobile Documents/com~apple~CloudDocs/Documents/MHT and Women's Health_umbrella review/MHT umbrella review project_R/PI Calculation"
```

2. Load Packages

```
library(readxl)
library(MetaUtility)
library(metafor)
library(dplyr)
library(robumeta)
library(rio)
library(weightr)
library(PublicationBias)
```

3. Obtain Data for Analysis

3.1 Import data

```
setwd(data.dir)
rawdata1 <- read_excel( "Outcome.xlsx", skip = 2 )

# manually check if row 2 is the correct cut-point for the dataset
View(rawdata1)

# remove any blank rows at the end
rawdata2 <- rawdata1[ !is.na(rawdata1$NumberOfControlGroup), ]
```

3.2 Drop variables not needed for analysis

```
##### For Continuous Outcome Reporting only MD or SMD with 95% CI #####
d <- rawdata2 %>% select( AuthorOfPaper, YearOfPublication, HRTDefinition, NumberOfInterventionGroup, NumberOfControlGroup, TypeOfEffectEstimate, PointEstimate, `Lower95%CI`, `Upper95%CI` )
```

```
##### For Continuous Outcome Reporting Mean and SD #####
d <- rawdata2 %>% select( AuthorOfPaper, YearOfPublication, HRTDefinition, Mean_Intervention,   SD_Intervention, NumberOfInterventionGroup, Mean_Control,    SD_Control, NumberOfControlGroup, TypeOfEffectEstimate, PointEstimate, `Lower95%CI`, `Upper95%CI` )
```

```
##### For Binary Outcome #####
rawdata3 <- rawdata2 %>% select( AuthorOfPaper, YearOfPublication, HRTDefinition, NumberOfEvents_Intervention, NumberOfInterventionGroup, NumberOfEvents_Control, NumberOfControlGroup )

# remove studies with no events in both intervention and control groups
d <- filter( rawdata3, rawdata3$NumberOfEvents_Intervention != 0 | rawdata3$NumberOfEvents_Control != 0 )
```

3.3 Check data

```
# check if there is any missing value
sum( is.na(d) )

# check if data type is correct
str(d)
```

4. Calculate Total Number of Events and Participants in Intervention and Control Groups

```
##### For Continuous Outcome #####
sum <- apply( d %>% select( NumberOfInterventionGroup, NumberOfControlGroup ), 2, FUN = sum )
matrix( data = sum, nrow = 1, dimnames = list( "No. of participants", c( "Intervention", "Control" ) ) )
```

```
##### For Binary Outcome #####
sum <- apply( d %>% select( NumberOfEvents_Intervention, NumberOfInterventionGroup, NumberOfEvents_Control, NumberOfControlGroup ), 2, FUN = sum )
matrix( data = sum, nrow = 2, byrow = TRUE, dimnames = list( c( "Intervention group", "Control group" ), c( "No. of events", "No. of participants" ) ) )
```

5. Calculate Point Estimates and Corresponding Sampling Variances

```
##### For Continuous Outcome Reporting only MD with 95% CI #####
# calculate approximate variance of MD from CI limits
d$yi.md <- as.numeric(d$PointEstimate)
d$vi.md <- MetaUtility::scrape_meta( type = "raw", est = d$yi.md, hi = as.numeric(d$`Upper95%CI`) )$vyi
d$sei.md <- sqrt(d$vi.md)
```

```
##### For Continuous Outcome Reporting Mean and SD for MD #####
# calculate MD and corresponding variance
d <- escalc( measure = "MD", m1i = as.numeric(d$Mean_Intervention), sd1i = as.numeric(d$SD_Intervention), n1i = d$NumberOfInterventionGroup, m2i = as.numeric(d$Mean_Control), sd2i = as.numeric(d$SD_Control), n2i = d$NumberOfControlGroup, data = d, var.names = c( "yi.md", "vi.md" ) )
d$sei.md <- sqrt(d$vi.md)

# approximately convert MD to logRR
d$logRR <- escalc( measure = "D2ORL", m1i = as.numeric(d$Mean_Intervention), sd1i = as.numeric(d$SD_Intervention), n1i = d$NumberOfInterventionGroup, m2i = as.numeric(d$Mean_Control), sd2i = as.numeric(d$SD_Control), n2i = d$NumberOfControlGroup, data = d )$yi / 2
d$varlogRR <- escalc( measure = "D2ORL", m1i = as.numeric(d$Mean_Intervention), sd1i = as.numeric(d$SD_Intervention), n1i = d$NumberOfInterventionGroup, m2i = as.numeric(d$Mean_Control), sd2i = as.numeric(d$SD_Control), n2i = d$NumberOfControlGroup, data = d )$vi / 4
d$seilogRR <- sqrt(d$varlogRR)
```

```
##### For Continuous Outcome Reporting only SMD with 95% CI #####
# calculate approximate variance of SMD from CI limits
d$yi.smd <- as.numeric(d$PointEstimate)
d$vi.smd <- MetaUtility::scrape_meta( type = "raw", est = d$yi.smd, hi = as.numeric(d$`Upper95%CI`) )$vyi
d$sei.smd <- sqrt(d$vi.smd)

# approximately convert SMD to logRR
d$logRR <- ( pi / ( sqrt(3)*2 ) ) * d$yi.smd
d$varlogRR <- ( pi / ( sqrt(3)*2 ) )^2 * d$vi.smd
d$seilogRR <- sqrt(d$varlogRR)
```

```
##### For Continuous Outcome Reporting Mean and SD for SMD #####
# calculate SMD and corresponding variance
d <- escalc( measure = "SMD", m1i = as.numeric(d$Mean_Intervention), sd1i = as.numeric(d$SD_Intervention), n1i = d$NumberOfInterventionGroup, m2i = as.numeric(d$Mean_Control), sd2i = as.numeric(d$SD_Control), n2i = d$NumberOfControlGroup, data = d, var.names = c( "yi.smd", "vi.smd" ) )
d$sei.smd <- sqrt(d$vi.smd)

# approximately convert SMD to logRR
d$logRR <- escalc( measure = "D2ORL", m1i = as.numeric(d$Mean_Intervention), sd1i = as.numeric(d$SD_Intervention), n1i = d$NumberOfInterventionGroup, m2i = as.numeric(d$Mean_Control), sd2i = as.numeric(d$SD_Control), n2i = d$NumberOfControlGroup, data = d )$yi / 2
d$varlogRR <- escalc( measure = "D2ORL", m1i = as.numeric(d$Mean_Intervention), sd1i = as.numeric(d$SD_Intervention), n1i = d$NumberOfInterventionGroup, m2i = as.numeric(d$Mean_Control), sd2i = as.numeric(d$SD_Control), n2i = d$NumberOfControlGroup, data = d )$vi / 4
d$seilogRR <- sqrt(d$varlogRR)
```

```
##### For Binary Outcome #####
d <- escalc( measure = "RR", ai = d$NumberOfEvents_Intervention, ci = d$NumberOfEvents_Control, n1i = d$NumberOfInterventionGroup, n2i = d$NumberOfControlGroup, data = d, var.names = c( "logRR", "varlogRR" ) )
d$seilogRR <- sqrt(d$varlogRR)
```

6. Obtain Effect Estimate for the Most Precise Study

```
# on MD scale
largest.study.md <- d[ which.min(d$sei.md), ] # the study with the smallest SE
round( matrix(
  c( largest.study.md$yi.md,
     largest.study.md$yi.md - qnorm(.975) * largest.study.md$sei.md,
     largest.study.md$yi.md + qnorm(.975) * largest.study.md$sei.md ),
  nrow = 1,
  dimnames = list( "Mean difference", c( "Point estimate", "Lower 95% CI", "Upper 95% CI" ) )
  ), 2 )
```

```
# on SMD scale
largest.study.smd <- d[ which.min(d$sei.smd), ] # the study with the smallest SE
round( matrix(
  c( largest.study.smd$yi.smd,
     largest.study.smd$yi.smd - qnorm(.975) * largest.study.smd$sei.smd,
     largest.study.smd$yi.smd + qnorm(.975) * largest.study.smd$sei.smd ),
  nrow = 1,
  dimnames = list( "Standardized mean difference", c( "Point estimate", "Lower 95% CI", "Upper 95% CI" ) )
  ), 2 )
```

```
# on RR scale
largest.study.logRR <- d[ which.min(d$seilogRR), ] # the study with the smallest SE
round( matrix(
  transf.exp.int( c( largest.study.logRR$logRR,
                     largest.study.logRR$logRR - qnorm(.975) * largest.study.logRR$seilogRR,
                     largest.study.logRR$logRR + qnorm(.975) * largest.study.logRR$seilogRR ) ),
  nrow = 1,
  dimnames = list( "Risk ratio", c( "Point estimate", "Lower 95% CI", "Upper 95% CI" ) )
  ), 2 )
```

7. Robust Random-Effects Meta-Analysis

7.1 Fit robust variance estimation model

```
##### Meta-Analysis of Independent Effect Sizes #####
# on MD scale
meta.naive.md <- robu( yi.md ~ 1, data = d, studynum = 1:nrow(d), var.eff.size = vi.md, small = TRUE )

# on SMD scale
meta.naive.smd <- robu( yi.smd ~ 1, data = d, studynum = 1:nrow(d), var.eff.size = vi.smd, small = TRUE )

# on RR scale
meta.naive.logRR <- robu( logRR ~ 1, data = d, studynum = 1:nrow(d), var.eff.size = varlogRR, small = TRUE )
```

```
##### Meta-Analysis of NON-independent Effect Sizes #####
# make a unique identifier for estimates that should be considered clustered
# outcome "Lipoprotein (a)" as an example:
d$author <- unlist( lapply( X = as.list(d$AuthorOfPaper), FUN = function(x) strsplit(x, " ")[[1]][1] ) )
d$cluster <- paste( d$author, d$YearOfPublication ) # clustering is at the level of papers (author & year)

# on MD scale
meta.naive.md <- robu( yi.md ~ 1, data = d, studynum = cluster, model = "CORR", var.eff.size = vi.md, small = TRUE )

# on SMD scale
meta.naive.smd <- robu( yi.smd ~ 1, data = d, studynum = cluster, model = "CORR", var.eff.size = vi.smd, small = TRUE )

# on RR scale
meta.naive.logRR <- robu( logRR ~ 1, data = d, studynum = cluster, model = "CORR", var.eff.size = varlogRR, small = TRUE )
```

7.2 Extract meta-analysis results

```
# on MD scale
muhat.naive.md <- meta.naive.md$b.r # pooled point estimate
muhat.lo.naive.md <- meta.naive.md$reg_table$CI.L # lower CI limit
muhat.hi.naive.md <- meta.naive.md$reg_table$CI.U # upper CI limit

round( matrix(
  c( muhat.naive.md, muhat.lo.naive.md, muhat.hi.naive.md ),
  nrow = 1,
  dimnames = list( "Mean difference", c( "Summary estimate", "Lower 95% CI", "Upper 95% CI" ) )
  ), 2 )

format.pval( meta.naive.md$reg_table$prob, digits = 2, scientific = TRUE ) # p-value
round( sqrt(meta.naive.md$mod_info$tau.sq), 2 ) # tau
```

```
# on SMD scale
muhat.naive.smd <- meta.naive.smd$b.r # pooled point estimate
muhat.lo.naive.smd <- meta.naive.smd$reg_table$CI.L # lower CI limit
muhat.hi.naive.smd <- meta.naive.smd$reg_table$CI.U # upper CI limit

round( matrix(
  c( muhat.naive.smd, muhat.lo.naive.smd, muhat.hi.naive.smd ),
  nrow = 1,
  dimnames = list( "Standardized mean difference", c( "Summary estimate", "Lower 95% CI", "Upper 95% CI" ) )
  ), 2 )

format.pval( meta.naive.smd$reg_table$prob, digits = 2, scientific = TRUE ) # p-value
round( sqrt(meta.naive.smd$mod_info$tau.sq), 2 ) # tau
```

```
# on RR scale
muhat.naive.logRR <- meta.naive.logRR$b.r # pooled point estimate
muhat.lo.naive.logRR <- meta.naive.logRR$reg_table$CI.L # lower CI limit
muhat.hi.naive.logRR <- meta.naive.logRR$reg_table$CI.U # upper CI limit

round( matrix(
  transf.exp.int( c( muhat.naive.logRR, muhat.lo.naive.logRR, muhat.hi.naive.logRR ) ),
  nrow = 1,
  dimnames = list( "Risk ratio", c( "Summary estimate", "Lower 95% CI", "Upper 95% CI" ) )
  ), 2 )

format.pval( meta.naive.logRR$reg_table$prob, digits = 2, scientific = TRUE ) # p-value
round( sqrt(meta.naive.logRR$mod_info$tau.sq), 2 ) # tau
```

8. Calculate 95% Non-parametric Prediction Interval

```
# on MD scale
setwd(file.dir)
export( d %>% select( AuthorOfPaper, yi.md, sei.md ), "Outcome.xlsx" )
# import "Outcome.xlsx" into a ready-to-use spreadsheet to calculate 95% PI (citation: Wang 2019, available from https://onlinelibrary.wiley.com/doi/abs/10.1002/jrsm.1345)
round( matrix(
  c( , ),
  nrow = 1,
  dimnames = list( "95% nonparametric PI (MD)", c( "Lower limit", "Upper limit" ) )
  ), 2 )
```

```
# on SMD scale
setwd(file.dir)
export( d %>% select( AuthorOfPaper, yi.smd, sei.smd ), "Outcome.xlsx" )
# import "Outcome.xlsx" into a ready-to-use spreadsheet to calculate 95% PI (citation: Wang 2019, available from https://onlinelibrary.wiley.com/doi/abs/10.1002/jrsm.1345)
round( matrix(
  c( , ),
  nrow = 1,
  dimnames = list( "95% nonparametric PI (SMD)", c( "Lower limit", "Upper limit" ) )
  ), 2 )
```

```
# on RR scale
setwd(file.dir)
export( d %>% select( AuthorOfPaper, logRR, seilogRR ), "Outcome.xlsx" )
# import "Outcome.xlsx" into a ready-to-use spreadsheet to calculate 95% PI (citation: Wang 2019, available from https://onlinelibrary.wiley.com/doi/abs/10.1002/jrsm.1345)
round( matrix(
 transf.exp.int( c( , ) ),
  nrow = 1,
  dimnames = list( "95% nonparametric PI (RR)", c( "Lower limit", "Upper limit" ) )
  ), 2 )
```

9. Estimate Proportion of True Effects Above or Below a Threshold of Scientific Importance

```
# on MD scale
# proportion of true effect sizes below 0
Phat.below.0.md <- prop_stronger( q = 0, tail = "below", estimate.method = "calibrated", ci.method = "calibrated", dat = d, R = 2000, yi.name = "yi.md", vi.name = "vi.md" )

# proportion of true effect sizes above 0
Phat.above.0.md <- prop_stronger( q = 0, tail = "above", estimate.method = "calibrated", ci.method = "calibrated", dat = d, R = 2000, yi.name = "yi.md", vi.name = "vi.md" )

round( matrix(
  c( Phat.below.0.md$est, Phat.below.0.md$lo, Phat.below.0.md$hi, # below 0
     Phat.above.0.md$est, Phat.above.0.md$lo, Phat.above.0.md$hi  # above 0
     ) * 100,
  nrow = 2,
  byrow = TRUE,
  dimnames = list( c( "Below 0", "Above 0" ), c( "Point estimate (%)", "Lower limit (%)", "Upper limit (%)" ) )
  ) )
```

```
# on SMD scale
# proportion of true effect sizes below -0.2
`Phat.below.-0.2.smd` <- prop_stronger( q = -0.2, tail = "below", estimate.method = "calibrated", ci.method = "calibrated", dat = d, R = 2000, yi.name = "yi.smd", vi.name = "vi.smd" )

# proportion of true effect sizes below 0
Phat.below.0.smd <- prop_stronger( q = 0, tail = "below", estimate.method = "calibrated", ci.method = "calibrated", dat = d, R = 2000, yi.name = "yi.smd", vi.name = "vi.smd" )

# proportion of true effect sizes above 0
Phat.above.0.smd <- prop_stronger( q = 0, tail = "above", estimate.method = "calibrated", ci.method = "calibrated", dat = d, R = 2000, yi.name = "yi.smd", vi.name = "vi.smd" )

# proportion of true effect sizes above 0.2
Phat.above.0.2.smd <- prop_stronger( q = 0.2, tail = "above", estimate.method = "calibrated", ci.method = "calibrated", dat = d, R = 2000, yi.name = "yi.smd", vi.name = "vi.smd" )

round( matrix(
  c( `Phat.below.-0.2.smd`$est, `Phat.below.-0.2.smd`$lo, `Phat.below.-0.2.smd`$hi, # below -0.2
     Phat.below.0.smd$est, Phat.below.0.smd$lo, Phat.below.0.smd$hi, # below 0
     Phat.above.0.smd$est, Phat.above.0.smd$lo, Phat.above.0.smd$hi, # above 0
     Phat.above.0.2.smd$est, Phat.above.0.2.smd$lo, Phat.above.0.2.smd$hi # above +0.2
     ) * 100,
  nrow = 4,
  byrow = TRUE,
  dimnames = list( c( "Below -0.2", "Below 0", "Above 0", "Above +0.2" ), c( "Point estimate (%)", "Lower limit (%)", "Upper limit (%)" ) )
  ) )
```

```
# on RR scale
# proportion of true effect sizes below 0.9
Phat.below.0.9.logRR <- prop_stronger( q = log(0.9), tail = "below", estimate.method = "calibrated", ci.method = "calibrated", dat = d, R = 2000, yi.name = "logRR", vi.name = "varlogRR" )

# proportion of true effect sizes below 1.0
Phat.below.1.0.logRR <- prop_stronger( q = log(1), tail = "below", estimate.method = "calibrated", ci.method = "calibrated", dat = d, R = 2000, yi.name = "logRR", vi.name = "varlogRR" )

# proportion of true effect sizes above 1.0
Phat.above.1.0.logRR <- prop_stronger( q = log(1), tail = "above", estimate.method = "calibrated", ci.method = "calibrated", dat = d, R = 2000, yi.name = "logRR", vi.name = "varlogRR" )

# proportion of true effect sizes above 1.1
Phat.above.1.1.logRR <- prop_stronger( q = log(1.1), tail = "above", estimate.method = "calibrated", ci.method = "calibrated", dat = d, R = 2000, yi.name = "logRR", vi.name = "varlogRR" )

round( matrix(
  c( Phat.below.0.9.logRR$est, Phat.below.0.9.logRR$lo, Phat.below.0.9.logRR$hi, # below 0.9
     Phat.below.1.0.logRR$est, Phat.below.1.0.logRR$lo, Phat.below.1.0.logRR$hi, # below 1.0
     Phat.above.1.0.logRR$est, Phat.above.1.0.logRR$lo, Phat.above.1.0.logRR$hi, # above 1.0
     Phat.above.1.1.logRR$est, Phat.above.1.1.logRR$lo, Phat.above.1.1.logRR$hi # above 1.1
     ) * 100,
  nrow = 4,
  byrow = TRUE,
  dimnames = list( c( "Below 0.9", "Below 1.0", "Above 1.0", "Above 1.1" ), c( "Point estimate (%)", "Lower limit (%)", "Upper limit (%)" ) )
  ) )
```

10. Evaluate Small-Study Effects

```
##### For Continuous Outcome on MD Scale #####
m <- rma( yi = d$yi.md, vi = d$vi.md, data = d, measure = "MD" ) # fitting a parametric random-effects model
regtest(m) # Egger's regression test
```

```
##### For Continuous Outcome on SMD Scale #####
m <- rma( yi = d$yi.smd, vi = d$vi.smd, data = d, measure = "SMD" ) # fitting a parametric random-effects model
regtest(m) # Egger's regression test
```

```
##### For Binary Outcome #####
m <- rma( yi = d$logRR, vi = d$varlogRR, data = d, measure = "RR" ) # fitting a parametric random-effects model
regtest(m) # Egger's regression test
```

11. Evaluate Publication Bias

11.1 Assess the direction of potential selection bias

```
##### For Continuous Outcome on MD Scale #####
pval_plot( yi = d$yi.md, vi = d$vi.md ) # plot one-tailed p-values
muhat.naive.md >0 # whether pooled point estimate is > 0
```

```
##### For Continuous Outcome on SMD Scale #####
pval_plot( yi = d$yi.smd, vi = d$vi.smd ) # plot one-tailed p-values
muhat.naive.smd >0 # whether pooled point estimate is > 0
```

```
##### For Binary Outcome #####
pval_plot( yi = d$logRR, vi = d$varlogRR ) # plot one-tailed p-values
muhat.naive.logRR >0 # whether pooled point estimate is > 0
```

11.2 Vevea & Hedges selection model

```
##### For Continuous Outcome on MD Scale #####
# flip the direction of d$yi.md to -d$yi.md if selection bias is likely to favor negative estimates
# start with three p-value cut-points
( m1 <- weightfunct( effect = d$yi.md, v = d$vi.md, steps = c( 0.025, 0.975, 1 ), table = TRUE ) )

# reduce to two p-value cut-points if fewer cut-points are required
( m1 <- weightfunct( effect = d$yi.md, v = d$vi.md, steps = c( 0.025, 1 ), table = TRUE ) )

# extract the corrected point estimate, CI, etc.
# get SEs via the Hessian
H <- m1[[2]]$hessian
ses <- sqrt( diag( solve(H) ) )

# flip sign back if the direction of d$yi.md is reversed above
muhat.corrected <- m1[[2]]$par[2] # point estimate
muhat.lo.corrected <- muhat.corrected - qnorm(.975) * ses[2] # CI lower limit
muhat.hi.corrected <- muhat.corrected + qnorm(.975) * ses[2] # CI upper limit

round( matrix(
  c( muhat.corrected, muhat.lo.corrected, muhat.hi.corrected ),
  nrow = 1,
  dimnames = list( "Mean difference", c( "Corrected estimate", "Lower 95% CI", "Upper 95% CI" ) )
  ), 2 )

# p-value of likelihood-ratio test
format.pval(  , digits = 2, scientific = TRUE )
```

```
##### For Continuous Outcome on SMD Scale #####
# flip the direction of d$yi.smd to -d$yi.smd if selection bias is likely to favor negative estimates
# start with three p-value cut-points
( m1 <- weightfunct( effect = d$yi.smd, v = d$vi.smd, steps = c( 0.025, 0.975, 1 ), table = TRUE ) )

# reduce to two p-value cut-points if fewer cut-points are required
( m1 <- weightfunct( effect = d$yi.smd, v = d$vi.smd, steps = c( 0.025, 1 ), table = TRUE ) )

# extract the corrected point estimate, CI, etc.
# get SEs via the Hessian
H <- m1[[2]]$hessian
ses <- sqrt( diag( solve(H) ) )

# flip sign back if the direction of d$yi.smd is reversed above
muhat.corrected <- m1[[2]]$par[2] # point estimate
muhat.lo.corrected <- muhat.corrected - qnorm(.975) * ses[2] # CI lower limit
muhat.hi.corrected <- muhat.corrected + qnorm(.975) * ses[2] # CI upper limit

round( matrix(
  c( muhat.corrected, muhat.lo.corrected, muhat.hi.corrected ),
  nrow = 1,
  dimnames = list( "Standardized mean difference", c( "Corrected estimate", "Lower 95% CI", "Upper 95% CI" ) )
  ), 2 )

# p-value of likelihood-ratio test
format.pval(   , digits = 2, scientific = TRUE )
```

```
##### For Binary Outcome #####
# flip the direction of d$logRR to -d$logRR if selection bias is likely to favor negative estimates
# start with three p-value cut-points
( m1 <- weightfunct( effect = d$logRR, v = d$varlogRR, steps = c( 0.025, 0.975, 1 ), table = TRUE ) )

# reduce to two p-value cut-points if fewer cut-points are required
( m1 <- weightfunct( effect = d$logRR, v = d$varlogRR, steps = c( 0.025, 1 ), table = TRUE ) )

# extract the corrected point estimate, CI, etc.
# get SEs via the Hessian
H <- m1[[2]]$hessian
ses <- sqrt( diag( solve(H) ) )

# flip sign back if the direction of d$logRR is reversed above
muhat.corrected <- m1[[2]]$par[2] # point estimate
muhat.lo.corrected <- muhat.corrected - qnorm(.975) * ses[2] # CI lower limit
muhat.hi.corrected <- muhat.corrected + qnorm(.975) * ses[2] # CI upper limit

round( matrix(
  transf.exp.int( c( muhat.corrected, muhat.lo.corrected, muhat.hi.corrected ) ),
  nrow = 1,
  dimnames = list( "Risk ratio", c( "Corrected estimate", "Lower 95% CI", "Upper 95% CI" ) )
  ), 2 )

# p-value of likelihood-ratio test
format.pval(  , digits = 2, scientific = TRUE )
```

11.3 Worst-case meta-analysis

11.3.1 Fit robust variance estimation model

```
##### Worst-Case Meta-Analysis of Independent Effect Sizes #####
# affirmative studies: p < 0.05 and estimate in same direction as the pooled point estimate
# nonaffirmative studies: p >= 0.05 or estimate in the opposite direction
# meta-analyze only the nonaffirmative studies

# For Continuous Outcome on MD Scale
d$affirm <- ( sign(d$yi.md) == sign( c(muhat.naive.md) ) ) & ( abs( d$yi.md / d$sei.md ) > qnorm(.975) )
meta.worst <- robu( yi.md ~ 1, data = d[ d$affirm == FALSE, ], studynum = 1:nrow( d[ d$affirm == FALSE, ] ), var.eff.size = vi.md, small = TRUE )

# For Continuous Outcome on SMD Scale
d$affirm <- ( sign(d$yi.smd) == sign( c(muhat.naive.smd) ) ) & ( abs( d$yi.smd / d$sei.smd ) > qnorm(.975) )
meta.worst <- robu( yi.smd ~ 1, data = d[ d$affirm == FALSE, ], studynum = 1:nrow( d[ d$affirm == FALSE, ] ), var.eff.size = vi.smd, small = TRUE )

# For Binary Outcome
d$affirm <- ( sign(d$logRR) == sign( c(muhat.naive.logRR) ) ) & ( abs( d$logRR / d$seilogRR ) > qnorm(.975) )
meta.worst <- robu( logRR ~ 1, data = d[ d$affirm == FALSE, ], studynum = 1:nrow( d[ d$affirm == FALSE, ] ), var.eff.size = varlogRR, small = TRUE )
```

```
##### Worst-Case Meta-Analysis of NON-independent Effect Sizes #####
# affirmative studies: p < 0.05 and estimate in same direction as the pooled point estimate
# nonaffirmative studies: p >= 0.05 or estimate in the opposite direction
# meta-analyze only the nonaffirmative studies

# For Continuous Outcome on MD Scale
d$affirm <- ( sign(d$yi.md) == sign( c(muhat.naive.md) ) ) & ( abs( d$yi.md / d$sei.md ) > qnorm(.975) )
meta.worst <- robu( yi.md ~ 1, data = d[ d$affirm == FALSE, ], studynum = cluster, var.eff.size = vi.md, modelweights = "CORR", small = TRUE )

# For Continuous Outcome on SMD Scale
d$affirm <- ( sign(d$yi.smd) == sign( c(muhat.naive.smd) ) ) & ( abs( d$yi.smd / d$sei.smd ) > qnorm(.975) )
meta.worst <- robu( yi.smd ~ 1, data = d[ d$affirm == FALSE, ], studynum = cluster, var.eff.size = vi.smd, modelweights = "CORR", small = TRUE )

# For Binary Outcome
d$affirm <- ( sign(d$logRR) == sign( c(muhat.naive.logRR) ) ) & ( abs( d$logRR / d$seilogRR ) > qnorm(.975) )
meta.worst <- robu( logRR ~ 1, data = d[ d$affirm == FALSE, ], studynum = cluster, var.eff.size = varlogRR, modelweights = "CORR", small = TRUE )
```

11.3.2 Extract worst-case meta-analysis results

```
##### For Continuous Outcome on MD Scale #####
muhat.worst <- meta.worst$b.r
muhat.lo.worst <- meta.worst$reg_table$CI.L
muhat.hi.worst <- meta.worst$reg_table$CI.U

round( matrix(
  c( muhat.worst, muhat.lo.worst, muhat.hi.worst ),
  nrow = 1,
  dimnames = list( "Mean difference", c( "Worst-case estimate", "Lower 95% CI", "Upper 95% CI" ) )
  ), 2 )

# when there is only one nonaffirmative study
nonaffirmative.study <- d[ d$affirm == FALSE, ]
round( matrix(
  c( nonaffirmative.study$yi.md,
     nonaffirmative.study$yi.md - qnorm(.975) * nonaffirmative.study$sei.md,
     nonaffirmative.study$yi.md + qnorm(.975) * nonaffirmative.study$sei.md ) ,
  nrow = 1,
  dimnames = list( "Mean difference", c( "Worst-case estimate", "Lower 95% CI", "Upper 95% CI" ) )
  ), 2 )
```

```
##### For Continuous Outcome on SMD Scale #####
muhat.worst <- meta.worst$b.r
muhat.lo.worst <- meta.worst$reg_table$CI.L
muhat.hi.worst <- meta.worst$reg_table$CI.U

round( matrix(
  c( muhat.worst, muhat.lo.worst, muhat.hi.worst ),
  nrow = 1,
  dimnames = list( "Standardized mean difference", c( "Worst-case estimate", "Lower 95% CI", "Upper 95% CI" ) )
  ), 2 )

# when there is only one nonaffirmative study
nonaffirmative.study <- d[ d$affirm == FALSE, ]
round( matrix(
  c( nonaffirmative.study$yi.smd,
     nonaffirmative.study$yi.smd - qnorm(.975) * nonaffirmative.study$sei.smd,
     nonaffirmative.study$yi.smd + qnorm(.975) * nonaffirmative.study$sei.smd ) ,
  nrow = 1,
  dimnames = list( "Standardized mean difference", c( "Worst-case estimate", "Lower 95% CI", "Upper 95% CI" ) )
  ), 2 )
```

```
##### For Binary Outcome #####
muhat.worst <- meta.worst$b.r
muhat.lo.worst <- meta.worst$reg_table$CI.L
muhat.hi.worst <- meta.worst$reg_table$CI.U

round( matrix(
  transf.exp.int( c( muhat.worst, muhat.lo.worst, muhat.hi.worst ) ),
  nrow = 1,
  dimnames = list( "Risk ratio", c( "Worst-case estimate", "Lower 95% CI", "Upper 95% CI" ) )
  ), 2 )

# when there is only one nonaffirmative study
nonaffirmative.study <- d[ d$affirm == FALSE, ]
round( matrix(
  transf.exp.int( c( nonaffirmative.study$logRR,
                     nonaffirmative.study$logRR - qnorm(.975) * nonaffirmative.study$seilogRR,
                     nonaffirmative.study$logRR + qnorm(.975) * nonaffirmative.study$seilogRR ) ),
  nrow = 1,
  dimnames = list( "Risk ratio", c( "Worst-case estimate", "Lower 95% CI", "Upper 95% CI" ) )
  ), 2 )
```

11.4 S-value

```
##### Independent Effect Sizes #####

# For Continuous Outcome on MD Scale
# if selection bias favors postive estimates
svalue( yi = d$yi.md, vi = d$vi.md, q = 0, model = "robust", favor.positive = TRUE, small = TRUE )
# if selection bias favors negative estimates
svalue( yi = d$yi.md, vi = d$vi.md, q = 0, model = "robust", favor.positive = FALSE, small = TRUE )

# For Continuous Outcome on SMD Scale
# if selection bias favors postive estimates
svalue( yi = d$yi.smd, vi = d$vi.smd, q = 0, model = "robust", favor.positive = TRUE, small = TRUE )
svalue( yi = d$yi.smd, vi = d$vi.smd, q = 0.2, model = "robust", favor.positive = TRUE, small = TRUE )
# if selection bias favors negative estimates
svalue( yi = d$yi.smd, vi = d$vi.smd, q = 0, model = "robust", favor.positive = FALSE, small = TRUE )
svalue( yi = d$yi.smd, vi = d$vi.smd, q = -0.2, model = "robust", favor.positive = FALSE, small = TRUE )

# For Binary Outcome
# if selection bias favors postive estimates
svalue( yi = d$logRR, vi = d$varlogRR, q = log(1.0), model = "robust", favor.positive = TRUE, small = TRUE )
svalue( yi = d$logRR, vi = d$varlogRR, q = log(1.1), model = "robust", favor.positive = TRUE, small = TRUE )
# if selection bias favors negative estimates
svalue( yi = d$logRR, vi = d$varlogRR, q = log(1.0), model = "robust", favor.positive = FALSE, small = TRUE )
svalue( yi = d$logRR, vi = d$varlogRR, q = log(0.9), model = "robust", favor.positive = FALSE, small = TRUE )
```

```
##### NON-independent Effect Sizes #####

# For Continuous Outcome on MD Scale
# if selection bias favors postive estimates
svalue( yi = d$yi.md, vi = d$vi.md, q = 0, clustervar = d$cluster, model = "robust", favor.positive = TRUE, small = TRUE )
# if selection bias favors negative estimates
svalue( yi = d$yi.md, vi = d$vi.md, q = 0, clustervar = d$cluster, model = "robust", favor.positive = FALSE, small = TRUE )

# For Continuous Outcome on SMD Scale
# if selection bias favors postive estimates
svalue( yi = d$yi.smd, vi = d$vi.smd, q = 0, clustervar = d$cluster, model = "robust", favor.positive = TRUE, small = TRUE )
svalue( yi = d$yi.smd, vi = d$vi.smd, q = 0.2, clustervar = d$cluster, model = "robust", favor.positive = TRUE, small = TRUE )
# if selection bias favors negative estimates
svalue( yi = d$yi.smd, vi = d$vi.smd, q = 0, clustervar = d$cluster, model = "robust", favor.positive = FALSE, small = TRUE )
svalue( yi = d$yi.smd, vi = d$vi.smd, q = -0.2, clustervar = d$cluster, model = "robust", favor.positive = FALSE, small = TRUE )

# For Binary Outcome
# if selection bias favors postive estimates
svalue( yi = d$logRR, vi = d$varlogRR, q = log(1.0), clustervar = d$cluster, model = "robust", favor.positive = TRUE, small = TRUE )
svalue( yi = d$logRR, vi = d$varlogRR, q = log(1.1), clustervar = d$cluster, model = "robust", favor.positive = TRUE, small = TRUE )
# if selection bias favors negative estimates
svalue( yi = d$logRR, vi = d$varlogRR, q = log(1.0), clustervar = d$cluster, model = "robust", favor.positive = FALSE, small = TRUE )
svalue( yi = d$logRR, vi = d$varlogRR, q = log(0.9), clustervar = d$cluster, model = "robust", favor.positive = FALSE, small = TRUE )
```
