# Supplementary material for: Menopausal hormone therapy and women’s health: An umbrella review
Source: PLoS Med. 2021 Aug 2;18(8):e1003731. doi: 10.1371/journal.pmed.1003731 (PMC8366967; doi:10.1371/journal.pmed.1003731)
Supplement: S2 Text — Table A: Characteristics of systematic reviews and/or meta-analyses on menopausal hormone therapy and multiple outcomes, updated on December 17, 2020. Fig A: Flow chart for study selection process, updated on December 17, 2020. (PDF) [file pmed.1003731.s003.pdf]

Updated Database Search for Umbrella Review on Menopausal Hormone Therapy and Women's Health

Given that our literature search for the umbrella review ended on November 26, 2017, to ascertain the amount of studies published since the last search, we performed an updated search of MEDLINE, EMBASE, Cochrane Database of Systematic Reviews, Database of Abstracts of Reviews of Effects, ISI Web of Science, CINAHL, AMED, Global Health, PsycINFO and CAB International from November 27, 2017 to December 17, 2020 using the same search strategies as the initial search (Table A in S1 Text). No language restriction was applied. Two investigators independently screened the titles and/or abstracts and reviewed full-text articles for eligibility. Any discrepancies were resolved by discussion.

In total, we identified 1,455 records, scrutinized 68 full-text articles, and ultimately obtained 37 articles from updated search that we judged were relevant to the review (Fig A below). Of the 37 articles, 10 meta-analyses of randomized controlled trials (RCTs), six meta-analyses of observational studies, nine meta-analyses of both RCTs and observational studies, and 12 systematic reviews without meta-analyses of RCTs and/or observational studies. The studies were published between 2017 and 2020. Given the time required to extract data, appraise the quality of the studies, and integrate the evidence from the studies into the umbrella review (we estimated that this may take a minimum of 12 months, during which time we anticipate that additional reviews must have been published on the topic that may also need to be integrated into the umbrella review, thereby making the process an unending one), we decided not to include the studies from the updated searches into the umbrella review. Rather, we take the evidence from the updated searches as explorative and therefore summarize the characteristics of each systematic review in the Table A below, including a statement of how the evidence generated from each systematic review may likely impact the current umbrella review if integrated in the review. This evidence analyses will be taken into account in the future when the current umbrella review is updated.

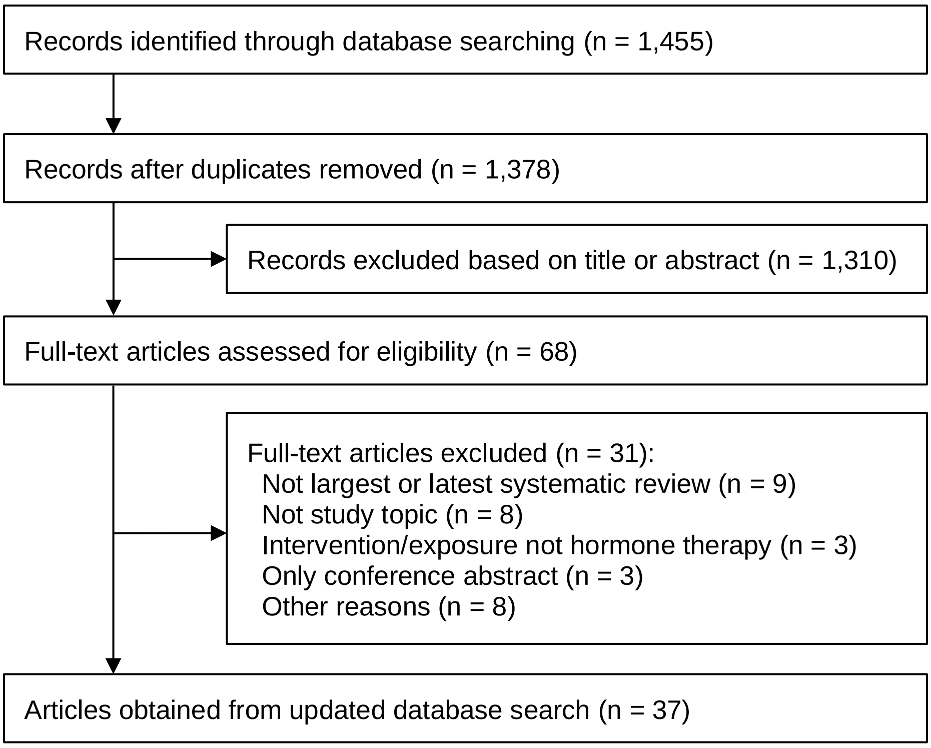

Fig A. Flow Chart for Study Selection Process, Updated on December 17, 2020

Table A. Characteristics of Systematic Reviews and/or Meta-Analyses on Menopausal Hormone Therapy and Multiple Outcomes, Updated on December 17, 2020

Outcome[a]	Reference	No. of Studies	Population	Intervention /Exposure[b]	Conclusions and Comments	Feature of Studies[c]
**Meta-analyses of both RCTs and observational studies**						
Dry eye disease	Dang 2020[1]	10 RCTs, 2 COs, 4 CSs	PM with dry eye disease	Any MHT	MHT showed a nonsignificant improvement in postoperative tear production and tear breakup time; not a critical outcome for clinical practice	
Urinary protein excretion	Kattah 2018[2]	3 RCTs, 1 case series, 4 COs, 1 CC, 3 CSs	PM	Any MHT	MHT was associated with a decreased risk of albuminuria; not a critical outcome for clinical practice	
Nephrolithiasis	Yu 2017[3]	2 RCTs, 1 CO, 1 CC	PM	Any MHT	There was no significant association between MHT and nephrolithiasis; not a critical outcome for clinical practice	
Lung cancer	Yin 2020[4]	38 studies (study design unspecified)	PPM	Any MHT	Overall, MHT was not associated with lung cancer risk	
Breast cancer	Kim 2018[5]	2 RCTs, 23 COs	PPM	Any MHT	Both ET and EPT were associated with an increased risk of breast cancer	
Breast cancer	Yang 2017[6]	5 RCTs, 6 COs, 3 CCs	PPM	Estradiol with/without progestin	Estradiol combined with progestin was associated with an increased risk of breast cancer, while the effect estimate for estradiol alone did not reach statistical significance	
Cardiovascular disease	Kim 2020[7]	26 RCTs, 30 COs, 17 CCs	PPM	Any MHT	MHT was associated with increased risks of venous thromboembolism, pulmonary embolism and stroke, but a decreased risk of myocardial infarction only in observational studies	
Dementia	Wu 2020[8]	2 RCTs, 3 COs	PPM	Any MHT	MHT was associated with an increased risk of dementia	
Breast cancer recurrence and survival	Mudhune 2019[9]	4 RCTs, 4 COs, 1 CC	Breast cancer survivors > 50 years old at diagnosis	Any MHT	In RCTs, MHT may increase the risk of recurrence, while in observational studies, MHT was associated with a decreased risk of recurrence and improved survival	

Table A. Characteristics of Systematic Reviews and/or Meta-Analyses on Menopausal Hormone Therapy and Multiple Outcomes, Updated on December 17, 2020 (continued)

Outcome[a]	Reference	No. of Studies	Population	Intervention /Exposure[b]	Conclusions and Comments	Feature of Studies[c]
**Meta-analyses of RCTs**						
Left ventricular diastolic function	Chen 2020[10]	8 RCTs	PM	ET/EPT	MHT may improve cardiac diastolic function; a surrogate outcome	
Endometrial cancer recurrence	Edey 2018[11]	1 RCT	Endometrial cancer survivors	Unspecified formulation	There was insufficient high-quality evidence; included only one trial	
Bone mineral density	Abdi 2017[12]	9 RCTs	PM	Transdermal ET with/without progestin	Transdermal estrogen increased bone mineral density	
Hip fracture	Barrionuevo 2019[13]	7 RCTs	PM	ET/EPT	MHT showed a significant reduction in hip facture	
Vertebral fracture	Barrionuevo 2019[13]	10 RCTs	PM	ET/EPT	MHT showed a significant reduction in vertebral facture	
Non-vertebral fracture	Barrionuevo 2019[13]	15 RCTs	PM	ET/EPT	MHT showed a significant reduction in non-vertebral facture	
Ovarian cancer overall survival	Saeaib 2020[14]	3 RCTs	Ovarian cancer survivors	ET/EPT	MHT may slightly improve overall survival, but the certainty of the evidence was low	
Sexual interest/arousal	Javadivala 2019[15]	18 RCTs	PPM with low sexual interest	ET/EPT	MHT improved sexual interest/arousal	
All-cause mortality	Nudy 2019[16]	28 RCTs	PPM	Systemic ET/EPT	Overall, MHT was not associated with all-cause mortality	
Cardiac death	Nudy 2019[16]	17 RCTs	PPM	Systemic ET/EPT	Overall, MHT was not associated with cardiac death	
Coronary heart disease	Nudy 2019[16]	22 RCTs	PPM	Systemic ET/EPT	Overall, MHT was not associated with coronary heart disease	
Recurrent urinary tract infection	Chen 2020[17]	8 RCTs	PM with/without recurrent urinary tract infection	ET/EPT	Vaginal ET reduced recurrent urinary tract infection	
Lean body mass	Javed 2019[18]	12 RCTs	PM	ET/EPT	MHT did not show a significant beneficial or detrimental effect on muscle mass; not a critical outcome for clinical practice	
Cognitive function	Zhou 2020[19]	2 RCTs	PPM with Alzheimer's disease	ET/EPT	MHT may improve the Alzheimer's Disease Assessment Scale-cognitive subscale score; included only two small trials, and a fixed-effect model was used	

Table A. Characteristics of Systematic Reviews and/or Meta-Analyses on Menopausal Hormone Therapy and Multiple Outcomes, Updated on December 17, 2020 (continued)

Outcome[a]	Reference	No. of Studies	Population	Intervention /Exposure[b]	Conclusions and Comments	Feature of Studies[c]
**Meta-analyses of observational studies**						
Colorectal cancer survival	Jang 2019[20]	5 COs	Colorectal cancer survivors	Any MHT	Current use of MHT was associated with improved colorectal cancer specific and overall survival	
Kidney cancer	Zhang 2020[21]	6 COs, 8 CCs	PPM	Any MHT	Overall, MHT was not associated with kidney cancer risk	
Colorectal adenoma	Song 2019[22]	8 studies (study design unspecified)	PPM	Any MHT	MHT was associated with a decreased risk of colorectal adenoma	
Glioma	Lan 2018[23]	4 COs, 10 CCs	PPM	Any MHT	MHT was associated with a decreased risk of glioma	
Ovarian cancer	Liu 2019[24]	15 COs, 19 CCs	PPM	Any MHT	MHT was associated with an increased risk of ovarian cancer, especially for serous and endometrioid tumors	
Melanoma	Sun 2020[25]	5 COs, 11 CCs	PPM	Any MHT	MHT was associated with an increased risk of melanoma	
Alzheimer's disease	Wu 2020[8]	6 COs, 9 CCs	PPM	Any MHT	MHT was associated with an increased risk of Alzheimer's disease, especially for EPT users	
Parkinson's disease	Wu 2020[8]	4 COs, 5 CCs	PPM	Any MHT	EPT was associated with an increased risk of Parkinson's disease	
**Systematic reviews without meta-analyses**						
Mammographic density	Azam 2020[26]	6 RCTs, 6 COs, 5 CCs, 5 CSs	PPM	Any MHT	MHT was positively associated with mammographic density with the highest increase among current users and continuous EPT users	
Faecal incontinence	Bach 2020[27]	1 RCT, 1 case series, 2 COs, 4 CSs	PM with/without faecal incontinence	Any MHT	All studies had a high risk of bias and had conflicting views on the effects of MHT on faecal incontinence	
Genitourinary syndrome of menopause	Biehl 2019[28]	24 RCTs	PM with genitourinary symptoms	Vaginal ET	Vaginal ET was effective for the treatment of genitourinary syndrome of menopause	

Table A. Characteristics of Systematic Reviews and/or Meta-Analyses on Menopausal Hormone Therapy and Multiple Outcomes, Updated on December 17, 2020 (continued)

Outcome[a]	Reference	No. of Studies	Population	Intervention /Exposure[b]	Conclusions and Comments	Feature of Studies[c]
Endometrial safety	Constantine 2019[29]	20 RCTs, 8 case series, 4 COs, 6 CCs	PPM	Vaginal ET	Current evidence did not support an increased risk of endometrial hyperplasia or cancer with low-dose vaginal ET	
Endometrial cancer	Tempfer 2020[30]	2 RCTs, 15 COs, 10 CCs	PPM	ET/EPT	Continuous EPT reduced the risk of endometrial cancer, whereas ET increased the risk	
Endometriosis recurrence	Zanello 2019[31]	2 RCTs, 2 COs	PPM with a history of endometriosis	Any MHT	The impact of MHT use on endometriosis recurrence and malignant transformation remained unclear	
Breast cancer recurrence	Wang 2018[32]	1 RCT, 3 COs	Breast cancer survivors < 50 years old	Any MHT	In RCTs, MHT may increase the risk of recurrence, while in observational studies, no significant association was found	
Cervical cancer incidence and survival	Vargiu 2020[33]	2 RCTs, 5 COs, 3 CCs	Women with/without cervical cancer	Any MHT	The relation between MHT and cervical cancer incidence was controversial, and no evidence reported a harmful effect of MHT on cervical cancer outcomes	
Cardiovascular disease	Bezwada 2017[34]	3 COs, 3 CCs	PPM	Transdermal MHT	There was some evidence suggestive of a possible protective cardiovascular effect with transdermal estrogen therapy	
Heart failure	Bolijn 2017[35]	1 CO, 1 RCT	PPM	Any MHT	Conflicting results existed for MHT and heart failure	
Periodontal parameters and dental implants osseointegration	Chaves 2020[36]	1 RCT, 6 CSs, 8 other studies (unspecified study design)	PM	Any MHT	There was no evidence to support MHT prescription for periodontal/implant placement purposes	
Body weight	Coquoz 2019[37]	8 RCTs, 2 COs	PM	Estrogen with micronized progesterone	Estrogen combined with micronized progesterone did not change or reduce body weight	
Body mass index	Coquoz 2019[37]	6 RCTs, 1 CO	PM	Estrogen with micronized progesterone	Estrogen combined with micronized progesterone had no impact on body mass index	
Glucose metabolism	Coquoz 2019[37]	7 RCTs, 4 COs	PM	Estrogen with micronized progesterone	Estrogen combined with micronized progesterone had a neutral or beneficial effect on glucose metabolism	

Abbreviations: CC, case-control study; CO, cohort study; CS, cross-sectional study; EPT, estrogen plus progestin therapy; ET, estrogen-alone therapy; MHT, menopausal hormone therapy; PM, postmenopausal women; PPM, peri-/post-menopausal women; RCT, randomized controlled trial.
[a] Incidence unless otherwise indicated.
[b] "Any MHT": any type of menopausal hormone therapy, such as estrogen alone, estrogen plus progestin, tibolone, selective estrogen receptor modulators, or unspecified; the comparator group is placebo or no treatment, unless otherwise indicated.
[c] The results of systematic reviews and/or meta-analyses were categorized into the following five categories:

(blue)	New outcome, not originally included in the umbrella review
(green)	Reached similar conclusion(s) as the current umbrella review
(yellow)	May likely adjust the evidence level in an updated umbrella review
(red)	Reached different conclusion(s) as the current umbrella review
(purple)	Systematic reviews without meta-analyses

References

1. Dang A, Nayeni M, Mather R, Malvankar-Mehta MS. Hormone replacement therapy for dry eye disease patients: systematic review and meta-analysis. *Can J Opthalmol* 2020;55(1):3-11.
2. Kattah AG, Suarez MLG, Milic N, et al. Hormone therapy and urine protein excretion: a multiracial cohort study, systematic review, and meta-analysis. *Menopause* 2018;25(6):625-34.
3. Yu J, Yin BY. Postmenopausal Hormone and the Risk of Nephrolithiasis: A Meta-Analysis. *Excli J* 2017;16:986-94.
4. Yin X, Zhu Z, Hosgood HD, Lan Q, Seow WJ. Reproductive factors and lung cancer risk: a comprehensive systematic review and meta-analysis. *BMC Public Health* 2020;20(1):1458.
5. Kim S, Ko Y, Lee H, Lim J. Menopausal hormone therapy and the risk of breast cancer by histological type and race: a meta-analysis of randomized controlled trials and cohort studies. *Breast Cancer Res Treat* 2018;170(3):667-75.
6. Yang ZL, Hu Y, Zhang J, Xu LZ, Zeng RJ, Kang DY. Estradiol therapy and breast cancer risk in perimenopausal and postmenopausal women: a systematic review and meta-analysis. *Gynecol Endocrinol* 2017;33(2):87-92.
7. Kim JE, Chang JH, Jeong MJ, et al. A systematic review and meta-analysis of effects of menopausal hormone therapy on cardiovascular diseases. *Sci Rep* 2020;10(1):20631.
8. Wu M, Li M, Yuan J, et al. Postmenopausal hormone therapy and Alzheimer's disease, dementia, and Parkinson's disease: A systematic review and time-response meta-analysis. *Pharmacol Res* 2020;155:104693.
9. Mudhune GH, Armour M, McBride KA. Safety of menopausal hormone therapy in breast cancer survivors older than fifty at diagnosis: A systematic review and meta-analysis. *Breast* 2019;47:43-55.
10. Chen JS, Mou YP, Li CE, Li YN, Yu J. Effects of hormone replacement therapy on left ventricular diastolic function in postmenopausal women: a systematic review and meta-analysis. *Gynecol Endocrinol* 2021;37(4):300-6.
11. Edey KA, Rundle S, Hickey M. Hormone replacement therapy for women previously treated for endometrial cancer. *Cochrane Database Syst Rev* 2018;5(5):CD008830.
12. Abdi F, Mobedi H, Bayat F, Mosaffa N, Dolatian M, Tehranif FR. The Effects of Transdermal Estrogen Delivery on Bone Mineral Density in Postmenopausal Women: A Meta-analysis. *Iran J Pharm Res* 2017;16(1):380-9.
13. Barrionuevo P, Kapoor E, Asi N, et al. Efficacy of Pharmacological Therapies for the Prevention of Fractures in Postmenopausal Women: A Network Meta-Analysis. *J Clin Endocrinol Metab* 2019;104(5):1623-30.
14. Saeaib N, Peeyananjarassri K, Liabsuetrakul T, Buhachat R, Myriokefalitaki E. Hormone replacement therapy after surgery for epithelial ovarian cancer. *Cochrane Database Syst Rev* 2020;1(1):CD012559.
15. Javadivala Z, Merghati-Khoei E, Jafarabadi MA, et al. Efficacy of pharmacological and non-pharmacological interventions on low sexual interest/arousal of peri- and post-menopausal women: a meta-analysis. *Sex Relation Ther* 2019;34(2):242-70.
16. Nudy M, Chinchilli VM, Foy AJ. A systematic review and meta-regression analysis to examine the 'timing hypothesis' of hormone replacement therapy on mortality, coronary heart disease, and stroke. *Int J Cardiol Heart Vasc* 2019;22:123-31.
17. Chen YY, Su TH, Lau HH. Estrogen for the prevention of recurrent urinary tract infections in postmenopausal women: a meta-analysis of randomized controlled trials. *Int Urogynecol J* 2021;32(1):17-25.
18. Javed AA, Mayhew AJ, Shea AK, Raina P. Association Between Hormone Therapy and Muscle Mass in Postmenopausal Women: A Systematic Review and Meta-analysis. *JAMA Netw Open* 2019;2(8):e1910154.
19. Zhou C, Wu Q, Wang Z, Wang Q, Liang Y, Liu S. The Effect of Hormone Replacement Therapy on Cognitive Function in Female Patients With Alzheimer's Disease: A Meta-Analysis. *Am J Alzheimers Dis Other Demen* 2020;35:1533317520938585.
20. Jang Y, Huang H, Leung C. Association of hormone replacement therapy with mortality in colorectal cancer survivor: a systematic review and meta-analysis. *BMC Cancer* 2019;19(1):1199.
21. Zhang XJ, Du YL, Tan XJ, et al. The Relationship Between Hormone Replacement Therapy and Risk of Kidney Cancer in Women: A Meta-Analysis. *Cancer Control* 2020;27(2):1073274820930194.
22. Song J, Jin Z, Han H, et al. Hormone replacement therapies, oral contraceptives, reproductive factors and colorectal adenoma risk: a systematic review and dose-response meta-analysis of observational studies. *Colorectal Dis* 2019;21(7):748-59.
23. Lan Y-L, Wang X, Lou J-C, et al. Update on the effect of exogenous hormone use on glioma risk in women: a meta-analysis of case-control and cohort studies. *J Neurooncol* 2018;137(2):357-65.

24. Liu Y, Ma L, Yang X, et al. Menopausal Hormone Replacement Therapy and the Risk of Ovarian Cancer: A Meta-Analysis. *Front Endocrinol* 2019;10:801.
25. Sun Q, Sun H, Cong L, Zheng Y, Wu N, Cong X. Effects of exogenous hormones and reproductive factors on female melanoma: A meta-analysis. *Clin Epidemiol* 2020;12:1183-203.
26. Azam S, Jacobsen KK, Aro AR, Lynge E, Andersen ZJ. Hormone replacement therapy and mammographic density: a systematic literature review. *Breast Cancer Res Treat* 2020;182(3):555-79.
27. Bach FL, Sairally BZF, Latthe P. Effect of oestrogen therapy on faecal incontinence in postmenopausal women: a systematic review. *Int Urogynecol J* 2020;31(7):1289-97.
28. Biehl C, Plotsker O, Mirkin S. A systematic review of the efficacy and safety of vaginal estrogen products for the treatment of genitourinary syndrome of menopause. *Menopause* 2019;26(4):431-53.
29. Constantine GD, Graham S, Lapane K, et al. Endometrial safety of low-dose vaginal estrogens in menopausal women: a systematic evidence review. *Menopause* 2019;26(7):800-7.
30. Tempfer CB, Hilal Z, Kern P, Juhasz-Boess I, Rezniczek GA. Menopausal Hormone Therapy and Risk of Endometrial Cancer: A Systematic Review. *Cancers* 2020;12(8):2195.
31. Zanello M, Borghese G, Manzara F, et al. Hormonal Replacement Therapy in Menopausal Women with History of Endometriosis: A Review of Literature. *Medicina* 2019;55(8):477.
32. Wang YM, Lewin N, Qaoud Y, Rajaee AN, Scheer AS. The oncologic impact of hormone replacement therapy in premenopausal breast cancer survivors: A systematic review. *Breast* 2018;40:123-30.
33. Vargiu V, Amar ID, Rosati A, et al. Hormone replacement therapy and cervical cancer: a systematic review of the literature. *Climacteric* 2021;24(2):120-7.
34. Bezwada P, Shaikh A, Misra D. The Effect of Transdermal Estrogen Patch Use on Cardiovascular Outcomes: A Systematic Review. *J Womens Health* 2017;26(12):1319-25.
35. Bolijn R, Onland-Moret NC, Asselbergs FW, van der Schouw YT. Reproductive factors in relation to heart failure in women: A systematic review. *Maturitas* 2017;106:57-72.
36. Chaves JDP, Figueredo TFM, Warnavin SVC, Pannuti CM, Steffens JP. Sex hormone replacement therapy in periodontology-A systematic review. *Oral Dis* 2020;26(2):270-84.
37. Coquoz A, Gruetter C, Stute P. Impact of micronized progesterone on body weight, body mass index, and glucose metabolism: a systematic review. *Climacteric* 2019;22(2):148-61.
