# Supplementary material for: Menopausal hormone therapy and women’s health: An umbrella review
Source: PLoS Med. 2021 Aug 2;18(8):e1003731. doi: 10.1371/journal.pmed.1003731 (PMC8366967; doi:10.1371/journal.pmed.1003731)
Supplement: S3 Text — (PDF) [file pmed.1003731.s004.pdf]

# Statistical Analysis Protocol (Version 4, July 18, 2020)

## 1. Prepare data for analysis

***Randomized controlled trials (RCTs)***

- For binary outcome, calculate log risk ratio (RR) and corresponding sampling variance.
- For continuous outcome measured as mean difference (MD), first convert MD to standardized mean difference (SMD)[1]:

$$SMD = \frac{MD}{SD_{pooled}}$$

$$SD_{pooled} = \sqrt{\frac{(N_E - 1){SD_E}^2 + (N_C - 1){SD_C}^2}{N_E + N_C - 2}}$$

where $SD_{pooled}$ is the pooled standard deviation for both intervention and control groups, $N_E$ and $SD_E$ are respectively the number of participants and standard deviation in intervention group, and $N_C$ and $SD_C$ are respectively the number of participants and standard deviation in control group. The variance of SMD would then be:

$$V_{SMD} = \frac{V_{MD}}{{SD_{pooled}}^2}$$

where $V_{MD}$ is the variance of MD. Then approximately convert SMD to log RR using[2,3]:

$$\log RR = SMD \times \frac{\pi}{\sqrt{3}} \times \frac{1}{2}$$

with variance

$$V_{\log RR} = V_{SMD} \times \frac{\pi^2}{3} \times \frac{1}{4}$$

***Observational epidemiological studies***

- Exclude cross-sectional studies, case-control studies with unclear temporality, studies that followed only the exposed group and reported standardized incidence or mortality ratios, and studies that reported only the unadjusted effect estimates.
- Include cohort and case-control studies with any type of effect estimate, i.e., maximally adjusted hazard ratio (HR)/incidence rate ratio (IRR)/odds ratio (OR)/RR.
- For IRR, approximately convert to RR[4]:

$$RR \approx IRR$$

- For HR/OR, when the outcome is relatively rare (< 15% by the end of follow-up), approximately convert to RR using[4]:

$$RR \approx HR \; or \; OR$$

when the outcome is common (≥ 15%)[3]:

$$RR \approx \frac{1 - 0.5^{\sqrt{HR}}}{1 - 0.5^{\sqrt{\frac{1}{HR}}}} \; or \; \sqrt{OR}$$

- Finally, calculate log RR and corresponding sampling variance.

## 2. Robust random-effects meta-analysis

The two commonly applied statistical models for meta-analysis are the fixed-effect model and the random-effects model.[5] The fixed-effect model assumes that all studies in the meta-analysis share a common true

effect size (or alternatively draws inference to only the sampled studies rather than the underlying distribution), whereas the random-effects model assumes that there is a distribution of true effect sizes. The random-effects assumption is generally a more plausible match to the underlying effect distribution than the fixed-effect, because studies in the meta-analysis are usually gathered from published literature.[5] Therefore, we used the random-effects model throughout the umbrella review. Of note, under the random-effects assumption the summary effect is an estimate of the **mean** of a distribution of true effects, rather than an estimate of a common true effect computed under fixed-effect assumption.

We used the robust variance estimation (RVE) method[6,7] (with random-effects weights) for meta-analysis. The RVE approach is increasingly being used in fields such as education, medicine, and psychology.[8,9] Compared to the conventional random-effects model with a normality assumption,[10] the RVE method has the following appealing advantages:

- It makes no distributional assumptions on the true population effects.
- With small-sample adjustments,[11] it can provide valid inference in meta-analyses with a small number of studies.
- It can accommodate dependence among the point estimates that can arise when some papers contribute multiple point estimates, without requiring knowledge of the exact structure of this dependence.

3. **Characterize the distribution of true effect sizes**
From random-effects meta-analysis, the summary estimate and its confidence interval only represent an estimate of the **mean effect size** and its **precision**. In presence of heterogeneity, they are usually insufficient to summarize the whole body of evidence. The **predictive distribution**, which describes how the true effects are distributed around the mean effect, is perhaps the most relevant and complete statistical inference to be drawn from random-effects meta-analysis.[10] We characterized this random-effects distribution by using three recently proposed metrics (see below), in addition to the heterogeneity estimate. These new metrics use “calibrated” estimates[12] without imposing any distributional assumptions. Based on simulation results,[13] they are recommended for use only in meta-analyses of ≥ 10 studies.

- **Tau ($\boldsymbol{T}$)**—the estimate of the standard deviation of true effect sizes[14]—it quantifies the absolute amount of heterogeneity. *Notes: The widely used metric $I^2$ is not a direct measure of absolute heterogeneity, and it only tells what proportion of the variation in observed effects is due to variation in true effects.[14,15] Therefore, $I^2$ was not reported.*
- **95% prediction interval**—estimation of the middle 95% area of the effect distribution[12]—it predicts with 95% confidence the true effect in a new study that is similar to the studies in the meta-analysis.
- $\boldsymbol{\hat{P}(\theta < q)}$—estimation of the lower tail of the effect distribution[13,16]—it estimates the proportion of true effects ($\theta$) below a threshold ($q$) of scientific importance. $q$ is defined as follows: for outcome measured as MD, $q$ = 0; for SMD, $q$ = -0.2 or 0; for RR, $q$ = 0.9 or 1.0.
- $\boldsymbol{\hat{P}(\theta > q^*)}$—estimation of the upper tail of the effect distribution[13,16]—it estimates the proportion of true effects ($\theta$) above a threshold ($q^*$) of scientific importance. $q^*$ is defined as follows: for outcome measured as MD, $q^*$ = 0; for SMD, $q^*$ = 0 or 0.2; for RR, $q^*$ = 1.0 or 1.1.

4. **Assess small-study effects**
Small-study effects describe the tendency for the smaller studies in a meta-analysis to show larger treatment effects.[17] Possible causes include dissemination bias (see Section 5), poor methodological quality, true heterogeneity, etc.[18] When there is an indication of small-study effects, particular caution is warranted when interpreting meta-analysis results. We used a random-effects Egger’s regression[18] to examine whether there was an association between treatment effect size and its standard error, and whether smaller studies tended to show more pronounced effects than larger studies. Based on current recommendation,[19] Egger’s regression was applied only in meta-analyses of ≥ 10 studies. *Notes: It is important to note that Egger’s regression and other statistical tests for funnel plot asymmetry should be seen as a generic means of examining small-study effects rather than a tool to diagnose specific types of bias.[17]*

- Indication of small-study effects[18]: “$\boldsymbol{P < 0.10}$ (two-sided) of Egger’s regression which indicates an association between treatment effect size and its standard error” plus “the random-effects summary estimate being larger than the point estimate of the largest study (the study with the smallest standard error) in the meta-analysis”.

5. **Assess dissemination bias**

Dissemination bias describes the "iceberg phenomenon" where the studies that appear in a systematic review and meta-analysis are **systematically** unrepresentative of all studies that have been conducted on a topic (Fig A).[20,21] It occurs when the dissemination profile of a study is determined by the direction or strength of the study findings, and may lead to an exaggerated or wholly distorted conclusion of the actual body of evidence. *Notes: Dissemination bias is not unique to systematic reviews and meta-analyses. It can threaten the validity of any type of literature-based review.*

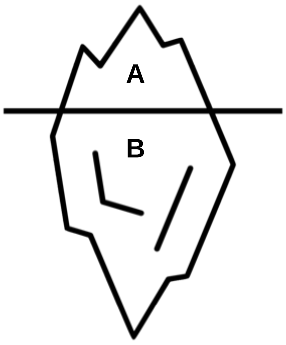


The whole iceberg represents all studies that have been conducted; the visible part (A) above waterline represents studies that are included in a systematic review and meta-analysis; the invisible part (B) below waterline represents unpublished or published (in any format) studies that are not identified by reviewers. The waterline can move upward or downward to hide or reveal more studies depending on the severity of dissemination bias.

**Fig A. The "Iceberg Phenomenon" of Dissemination Bias**

There are numerous information suppression mechanisms that can bias the dissemination of studies (see Song et al 2010[21] for a review), including **publication bias**, outcome reporting bias, language bias, etc. As illustrated in Fig A, the more severe the dissemination bias, the more difficult it will be to locate relevant studies and the less representative the studies included in a systematic review will be. Because the term "publication bias" has long been widely used in the literature, we used it to refer to dissemination bias in the paper.

Many statistical methods have been proposed to deal with publication bias in meta-analysis. In this umbrella review, we employed two modelling methods: Vevea and Hedges selection model[22] and S-value[23] (see below). They assume that the dissemination process selects studies with both point estimates in the direction of summary estimate and small *P*-values (e.g., $< 0.05$), which is termed as "one-tailed selection".[22] This assumption is justified by empirical findings on how applied researchers interpret *P*-values.[24] *Notes: Because the mechanisms of publication bias are quite complex and available statistical methods are often based on certain strong assumptions, these methods are mainly used as sensitivity analyses rather than as confirmatory tests.*[21,25]

- **Vevea and Hedges selection model**[22]—It offers a test for the presence of publication bias as well as a bias-corrected summary estimate. To model the one-tailed selection pattern, the following one-sided *P*-value intervals were used: $< 0.025$, $\geq 0.025$ and $\leq 0.975$, $> 0.975$. However, as noted in Vevea et al 2019,[26] the selection model requires a relatively large number of studies, and can perform poorly when there are only few or no observed effect sizes in the pre-specified *P*-value intervals. Therefore, we applied this method only in meta-analyses of $\geq 30$ studies, and reduced to two *P*-value intervals (i.e., $<$ 0.025, $\geq 0.025$) or did not use this method at all under certain circumstances.

- **S-value**[23]—In contrast to Vevea and Hedges selection model, this method can accommodate small meta-analyses, non-normal true effects, and non-independent effect sizes. The one-tailed selection process was modeled using a single two-sided *P*-value cutoff of 0.05 such that publication selects "affirmative" results (i.e., statistically significant point estimates in the direction of summary estimate) over "nonaffirmative" results (i.e., significant point estimates but in the opposite direction, or nonsignificant ones). It enables calculation of the following metrics: a. a summary estimate corrected for worst-case publication bias; b. the severity of publication bias (i.e., the ratio, $\eta$, by which affirmative studies are more likely to be published than nonaffirmative studies) that would be required to shift the pooled estimate or its confidence interval limit to a chosen threshold of scientific importance (i.e., the null or a non-null value $q$). The threshold is defined as follows: for outcome measured as MD, null = 0; for SMD, null = 0, $q$ = -0.2 or 0.2, and for RR, null = 1.0, $q$ = 0.9 or 1.1 ($q$ is the value in the same direction of the pooled estimate). A large $\eta$ would indicate that the meta-analysis is relatively robust to publication bias, whereas a small $\eta$ would indicate that the meta-analysis is relatively sensitive to publication bias. Informed by the empirical benchmarks for plausible values of $\eta$ in medicine, a $\eta$ of $\geq 4$ would represent implausibly severe or extreme publication bias.

**6. Sensitivity analysis for residual confounding in meta-analyses of observational studies**

Unlike (meta-analyses of) RCTs which are generally thought not to be confounded, a key concern in (meta-analyses of) observational studies is bias by residual confounding. Unfortunately, the fact that we seldomly know all the unmeasured confounders often leaves us uncertain as to how much the severity of residual confounding influences study effect estimates. Sensitivity analysis is an important approach to dealing with this issue. It assesses how robust an observed exposure-outcome association is to potential residual confounding, for example how strong residual confounding would have to be to "explain away" an observed association. In the umbrella review, we used three recently proposed metrics (see below), which adopt the sensitivity analysis idea, to assess the robustness of meta-analysis results to potential residual confounding.

- **E-value**[4,27]—The minimum strength of association, on RR scale, that residual confounding would need to have with both the exposure and outcome, conditional on the measured covariates and on average across studies, to shift the pooled estimate or its confidence interval limit to a chosen threshold of scientific importance (i.e., the null or a non-null value $q$). The threshold is defined as follows: null = 1.0, $q$ = 0.9 or 1.1 ($q$ is the value in the same direction of the pooled estimate). A large E-value would indicate that the meta-analysis mean estimate is relatively robust to residual confounding, whereas a small E-value would indicate that the meta-analysis mean estimate is relatively sensitive to residual confounding. We reported the E-value only in meta-analyses of $<$ 10 studies (for reasons, see below). *Notes: As noted in VanderWeele et al 2017,[4] the E-value is a continuous measure, and use of any threshold cutoff is discouraged; a small E-value only implies that the evidence for an effect is weak, but does not mean that there is evidence for no effect.*

- $\boldsymbol{\hat{T}(r,q)}$ **and** $\boldsymbol{\hat{G}(r,q)}$[13,27]—The minimum bias factor on RR scale, $\hat{T}(r,q)$, and the minimum confounding association strength, $\hat{G}(r,q)$, in all studies that would be required to reduce to less than $r$ the proportion of studies with true effects exceeding a chosen threshold of scientific importance (i.e., the null or a non-null value $q$), conditional on the measured covariates. The confounding association strength is the strength of association, on RR scale, that residual confounder(s) would have with both the exposure and outcome, conditional on the measured covariates (in direct analog to the E-value). The threshold and $r$ are defined as follows: null = 1.0, $q$ = 0.9 or 1.1 ($q$ is the value in the same direction of the pooled estimate); $r$ = 0.1 and 0.2 for meta-analyses of $\geq$ 16 studies and of 10–15 studies, respectively.[27] A large $\hat{T}(r,q)$ or $\hat{G}(r,q)$ would indicate that the meta-analysis result is relatively robust to residual confounding, whereas a small $\hat{T}(r,q)$ or $\hat{G}(r,q)$ would indicate that the meta-analysis result is relatively sensitive to residual confounding. In contrast to the E-value, these two metrics take into account effect heterogeneity which is central to random-effects meta-analyses. However, based on simulation results,[13] they are recommended for use only in meta-analyses of $\geq$ 10 studies. Therefore, for meta-analyses of $<$ 10 studies, only the E-value was reported.

## References


1. Hedges LV. Distribution theory for Glass's estimator of effect size and related estimators. *J Educ Behav Stat* 1981;6(2):107-28.
2. Hasselblad V, Hedges LV. Meta-analysis of screening and diagnostic tests. *Psychol Bull* 1995;117(1):167-78.
3. VanderWeele TJ. Optimal approximate conversions of odds ratios and hazard ratios to risk ratios. *Biometrics* 2020;76(3):746-52.
4. VanderWeele TJ, Ding P. Sensitivity Analysis in Observational Research: Introducing the E-Value. *Ann Intern Med* 2017;167(4):268-74.
5. Borenstein M, Hedges LV, Higgins JP, Rothstein HR. A basic introduction to fixed-effect and random-effects models for meta-analysis. *Res Synth Methods* 2010;1(2):97-111.
6. Hedges LV, Tipton E, Johnson MC. Robust variance estimation in meta-regression with dependent effect size estimates. *Res Synth Methods* 2010;1(1):39-65.
7. Hedges LV, Tipton E, Johnson MC. Erratum: Robust variance estimation in meta-regression with dependent effect size estimates. *Res Synth Methods* 2010;1(2):164-5.
8. Fisher Z, Tipton E. Robumeta: An R-package for robust variance estimation in meta-analysis. *arXiv preprint arXiv:150302220.* 2015.
9. Tanner-Smith EE, Tipton E. Robust variance estimation with dependent effect sizes: Practical considerations including a software tutorial in Stata and SPSS. *Res Synth Methods* 2014;5(1):13-30.
10. Higgins JP, Thompson SG, Spiegelhalter DJ. A re-evaluation of random-effects meta-analysis. *J R Stat Soc Ser A Stat Soc* 2009;172(1):137-59.
11. Tipton E. Small sample adjustments for robust variance estimation with meta-regression. *Psychol Methods* 2015;20(3):375-93.
12. Wang CC, Lee WC. A simple method to estimate prediction intervals and predictive distributions: Summarizing meta-analyses beyond means and confidence intervals. *Res Synth Methods* 2019;10(2):255-66.
13. Mathur MB, VanderWeele TJ. Robust Metrics and Sensitivity Analyses for Meta-analyses of Heterogeneous Effects. *Epidemiology* 2020;31(3):356-8.
14. Borenstein M, Higgins JP, Hedges LV, Rothstein HR. Basics of meta-analysis: $I^2$ is not an absolute measure of heterogeneity. *Res Synth Methods* 2017;8(1):5-18.
15. Borenstein M. Research Note: In a meta-analysis, the $I^2$ index does not tell us how much the effect size varies across studies. *J Physiother* 2020;66(2):135-9.
16. Mathur MB, VanderWeele TJ. New metrics for meta-analyses of heterogeneous effects. *Stat Med* 2019;38(8):1336-42.
17. Sterne JA, Gavaghan D, Egger M. Publication and related bias in meta-analysis: power of statistical tests and prevalence in the literature. *J Clin Epidemiol* 2000;53(11):1119-29.
18. Egger M, Davey Smith G, Schneider M, Minder C. Bias in meta-analysis detected by a simple, graphical test. *BMJ* 1997;315(7109):629-34.
19. Sterne JA, Sutton AJ, Ioannidis JP, et al. Recommendations for examining and interpreting funnel plot asymmetry in meta-analyses of randomised controlled trials. *BMJ* 2011;343:d4002.
20. Boissel JP, Haugh MC. The iceberg phenomenon and publication bias: the editors' fault? *Clin Trials Metaanal* 1993;28(6):309-15.
21. Song F, Parekh S, Hooper L, et al. Dissemination and publication of research findings: an updated review of related biases. *Health Technol Assess* 2010;14(8):iii, ix-xi, 1-193.
22. Vevea JL, Hedges LV. A general linear model for estimating effect size in the presence of publication bias. *Psychometrika* 1995;60(3):419-35.
23. Mathur MB, VanderWeele TJ. Sensitivity analysis for publication bias in meta-analyses. *J R Stat Soc Ser C Appl Stat* 2020;69(5):1091-119.
24. McShane BB, Gal D. Statistical significance and the dichotomization of evidence. *J Am Stat Assoc* 2017;112(519):885-95.
25. McShane BB, Böckenholt U, Hansen KT. Adjusting for Publication Bias in Meta-Analysis: An Evaluation of Selection Methods and Some Cautionary Notes. *Perspect Psychol Sci* 2016;11(5):730-49.
26. Vevea JL, Coburn K, Sutton A. Chapter 18: Publication bias. In: Cooper H, Hedges LV, Valentine JC (editors). The Handbook of Research Synthesis and Meta-Analysis. 3rd Edition. New York (USA): Russell Sage Foundation, 2019:383-429.

27. Mathur MB, VanderWeele TJ. Sensitivity analysis for unmeasured confounding in meta-analyses. *J Am Stat Assoc* 2020;115(529):163-72.
