# Supplementary material for: Menopausal hormone therapy and women’s health: An umbrella review
Source: PLoS Med. 2021 Aug 2;18(8):e1003731. doi: 10.1371/journal.pmed.1003731 (PMC8366967; doi:10.1371/journal.pmed.1003731)
Supplement: S5 Text — (PDF) [file pmed.1003731.s006.pdf]

Meta-Analysis of Observational Epidemiological Studies of Menopausal Hormone Therapy and Multiple Health Outcomes

1. Location of Datasets, Files and Helper Functions

```
data.dir <- "/Users/xzhagu/Library/Mobile Documents/com~apple~CloudDocs/Documents/MHT and Women's Health_umbrella
review/MHT umbrella review project_R/Observational Studies/Primary Prevention"
file.dir <- "/Users/xzhagu/Library/Mobile Documents/com~apple~CloudDocs/Documents/MHT and Women's Health_umbrella
review/MHT umbrella review project_R/PI Calculation"
code.dir <- "~/Library/Mobile Documents/com~apple~CloudDocs/Documents/MHT and Women's Health_umbrella review/MHT
umbrella review project_R/R Script/helper.R" # helper script is available at the Open Science Framework (https://
osf.io/dsy37/?view_only=a5b0bfea96694a54a1b5886e3fb03765)
```

2. Load Packages

```
library(boot)
library(readxl)
library(MetaUtility)
library(metafor)
library(dplyr)
library(robumeta)
library(rio)
library(weightr)
library(PublicationBias)
library(EValue)
source(code.dir)
```

3. Obtain Data for Analysis

```
setwd(data.dir)
rawdata1 <- read_excel( "Outcome.xlsx", skip = 2 )

# manually check if row 2 is the correct cut-point for the dataset
# manually check if there is any missing value in the variable 'NumberOfCases'
View(rawdata1)

# remove any blank rows at the end
rawdata2 <- rawdata1[ !is.na(rawdata1$NumberOfCases), ]

# drop variables not needed for analysis
d <- rawdata2 %>% select( AuthorOfPaper, YearOfPublication, StudyDesign, TimingOfHRT, HRTDefinition, NumberOfCase
s, NumberOfPopulation, TypeOfEffectEstimate, PointEstimate, `Lower95%CI`, `Upper95%CI` )

# check if there is any missing value
sum( is.na(d) )

# check if data type is correct
str(d)
```

4. Calculate Number of Studies, Cases and Population

```
d$study.design <- ifelse( d$StudyDesign == "Cohort", "CS", "CC" ) # CS, cohort study; CC, case-control study

matrix(
  c( nrow( d[ d$study.design == "CC", ] ), # number of case-control studies
     nrow( d[ d$study.design == "CS", ] ) ), # number of cohort studies
  nrow = 1,
  dimnames = list( "No. of studies", c( "Case-control", "Cohort" ) )
)

d$total.population <- ifelse( d$study.design == "CS",
                              d$NumberOfPopulation,
                              d$NumberOfCases + d$NumberOfPopulation )

sum <- apply( d %>% select( NumberOfCases, total.population ), 2, FUN = sum )
matrix( data = sum, nrow = 1, dimnames = list( "No. of participants", c( "Cases", "Population" ) ) )
```

5. Calculate Log Risk Ratios and Corresponding Sampling Variances

```
##### If the Outcome is Relatively Rare (< 15% by the End of Follow-Up) #####
d$logRR <- log( as.numeric(d$PointEstimate) )
d$varlogRR <- MetaUtility::scrape_meta( type = "RR",
                                        est = as.numeric(d$PointEstimate),
                                        hi = as.numeric(d$`Upper95%CI`) )$vyi
d$seilogRR <- sqrt(d$varlogRR)
```

```
##### If the Outcome is Common (>= 15% by the End of Follow-Up) #####
d$point.estimate <- as.numeric(d$PointEstimate)
d$upper.ci <- as.numeric(d$`Upper95%CI`)

d$RR <- ifelse( d$TypeOfEffectEstimate == "OR", sqrt(d$point.estimate),
                ifelse( d$TypeOfEffectEstimate == "HR", HR_to_RR(d$point.estimate),
                        d$point.estimate )
                )
d$RR.hi <- ifelse( d$TypeOfEffectEstimate == "OR", sqrt(d$upper.ci),
                   ifelse( d$TypeOfEffectEstimate == "HR", HR_to_RR(d$upper.ci),
                           d$upper.ci )
                   )

d$logRR <- log(d$RR)
d$varlogRR <- MetaUtility::scrape_meta( type = "RR",
                                        est = d$RR,
                                        hi = d$RR.hi )$vyi
d$seilogRR <- sqrt(d$varlogRR)
```

6. Obtain Effect Estimate for the Most Precise Study

```
largest.study <- d[ which.min(d$seilogRR), ] # the study with the smallest SE
round( matrix(
  transf.exp.int( c( largest.study$logRR,
                     largest.study$logRR - qnorm(.975) * largest.study$seilogRR,
                     largest.study$logRR + qnorm(.975) * largest.study$seilogRR ) ),
  nrow = 1,
  dimnames = list( "Risk ratio", c( "Point estimate", "Lower 95% CI", "Upper 95% CI" ) )
  ), 2 )
```

7. Robust Random-Effects Meta-Analysis

7.1 Fit robust variance estimation model

```
##### Meta-Analysis of Independent Effect Sizes #####
meta.naive.logRR <- robu( logRR ~ 1, data = d, studynum = 1:nrow(d), var.eff.size = varlogRR, small = TRUE )
```

```
##### Meta-Analysis of NON-independent Effect Sizes #####
# make a unique identifier for estimates that should be considered clustered
# outcome "Colorectal Cancer Incidence" as an example:
d$author <- unlist( lapply( X = as.list(d$AuthorOfPaper), FUN = function(x) strsplit(x, " ")[[1]][1] ) )
d$cluster <- paste( d$author, d$YearOfPublication ) # clustering is at the level of papers (author & year)

meta.naive.logRR <- robu( logRR ~ 1, data = d, studynum = cluster, model = "HIER", var.eff.size = varlogRR, small
= TRUE ) # model = "CORR" for correlated effects; model = "HIER" for hierarchical effects
```

7.2 Extract meta-analysis results

```
muhat.naive.logRR <- meta.naive.logRR$b.r # pooled point estimate
muhat.lo.naive.logRR <- meta.naive.logRR$reg_table$CI.L # lower CI limit
muhat.hi.naive.logRR <- meta.naive.logRR$reg_table$CI.U # upper CI limit

round( matrix(
  transf.exp.int( c( muhat.naive.logRR, muhat.lo.naive.logRR, muhat.hi.naive.logRR ) ),
  nrow = 1,
  dimnames = list( "Risk ratio", c( "Summary estimate", "Lower 95% CI", "Upper 95% CI" ) )
  ), 2 )

format.pval( meta.naive.logRR$reg_table$prob, digits = 2, scientific = TRUE ) # p-value
round( sqrt(meta.naive.logRR$mod_info$tau.sq), 2 ) # tau
```

8. Calculate 95% Non-parametric Prediction Interval

```
setwd(file.dir)
export( d %>% select( AuthorOfPaper, logRR, seilogRR ), "Outcome.xlsx" )
# import "Outcome.xlsx" into a ready-to-use spreadsheet to calculate 95% PI (citation: Wang 2019, available from
https://onlinelibrary.wiley.com/doi/abs/10.1002/jrsm.1345)

round( matrix(
 transf.exp.int( c(  ,  ) ),
  nrow = 1,
  dimnames = list( "95% nonparametric PI (RR)", c( "Lower limit", "Upper limit" ) )
  ), 2 )
```

9. Estimate Proportion of True Effects Above or Below a Threshold of Scientific Importance

```
# proportion of true effect sizes below 0.9
Phat.below.0.9.logRR <- prop_stronger( q = log(0.9), tail = "below", estimate.method = "calibrated", ci.method =
"calibrated", dat = d, R = 2000, yi.name = "logRR", vi.name = "varlogRR" )

# proportion of true effect sizes below 1.0
Phat.below.1.0.logRR <- prop_stronger( q = log(1.0), tail = "below", estimate.method = "calibrated", ci.method =
"calibrated", dat = d, R = 2000, yi.name = "logRR", vi.name = "varlogRR" )

# proportion of true effect sizes above 1.0
Phat.above.1.0.logRR <- prop_stronger( q = log(1.0), tail = "above", estimate.method = "calibrated", ci.method =
"calibrated", dat = d, R = 2000, yi.name = "logRR", vi.name = "varlogRR" )

# proportion of true effect sizes above 1.1
Phat.above.1.1.logRR <- prop_stronger( q = log(1.1), tail = "above", estimate.method = "calibrated", ci.method =
"calibrated", dat = d, R = 2000, yi.name = "logRR", vi.name = "varlogRR" )

round( matrix(
  c( Phat.below.0.9.logRR$est, Phat.below.0.9.logRR$lo, Phat.below.0.9.logRR$hi, # below 0.9
     Phat.below.1.0.logRR$est, Phat.below.1.0.logRR$lo, Phat.below.1.0.logRR$hi, # below 1.0
     Phat.above.1.0.logRR$est, Phat.above.1.0.logRR$lo, Phat.above.1.0.logRR$hi, # above 1.0
     Phat.above.1.1.logRR$est, Phat.above.1.1.logRR$lo, Phat.above.1.1.logRR$hi # above 1.1
     ) * 100,
  nrow = 4,
  byrow = TRUE,
  dimnames = list( c( "Below 0.9", "Below 1.0", "Above 1.0", "Above 1.1" ), c( "Point estimate (%)", "Lower limit
(%)", "Upper limit (%)" ) )
  ) )
```

10. Evaluate Small-Study Effects

```
m <- rma( yi = d$logRR, vi = d$varlogRR, data = d, measure = "RR" ) # fitting a parametric random-effects model
regtest(m) # Egger's regression test
```

11. Evaluate Publication Bias

11.1 Assess the direction of potential selection bias

```
pval_plot( yi = d$logRR, vi = d$varlogRR ) # plot one-tailed p-values
muhat.naive.logRR > 0 # whether pooled point estimate is > 0
```

11.2 Vevea & Hedges selection model

```
# flip the direction of d$logRR to -d$logRR if selection bias is likely to favor negative estimates
# start with three p-value cut-points
( m1 <- weightfunct( effect = d$logRR, v = d$varlogRR, steps = c( 0.025, 0.975, 1 ), table = TRUE ) )

# reduce to two p-value cut-points if fewer cut-points are required
( m1 <- weightfunct( effect = d$logRR, v = d$varlogRR, steps = c( 0.025, 1 ), table = TRUE ) )

# extract the corrected point estimate, CI, etc.
# get SEs via the Hessian
H <- m1[[2]]$hessian
ses <- sqrt( diag( solve(H) ) )

# flip sign back if the direction of d$logRR is reversed above
muhat.corrected <- m1[[2]]$par[2] # point estimate
muhat.lo.corrected <- muhat.corrected - qnorm(.975) * ses[2] # CI lower limit
muhat.hi.corrected <- muhat.corrected + qnorm(.975) * ses[2] # CI upper limit

round( matrix(
  transf.exp.int( c( muhat.corrected, muhat.lo.corrected, muhat.hi.corrected ) ),
  nrow = 1,
  dimnames = list( "Risk ratio", c( "Corrected estimate", "Lower 95% CI", "Upper 95% CI" ) )
  ), 2 )

# p-value of likelihood-ratio test
format.pval(  , digits = 2, scientific = TRUE )
```

11.3 Worst-case meta-analysis

11.3.1 Fit robust variance estimation model

```
##### Worst-Case Meta-Analysis of Independent Effect Sizes #####
# affirmative studies: p < 0.05 and estimate in same direction as the pooled point estimate
# nonaffirmative studies: p >= 0.05 or estimate in the opposite direction
# meta-analyze only the nonaffirmative studies
d$affirm <- ( sign(d$logRR) == sign( c(muhat.naive.logRR) ) ) & ( abs( d$logRR / d$seilogRR ) > qnorm(.975) )
meta.worst <- robu( logRR ~ 1,
                    data = d[ d$affirm == FALSE, ],
                    studynum = 1:nrow( d[ d$affirm == FALSE, ] ),
                    var.eff.size = varlogRR,
                    small = TRUE )
```

```
##### Worst-Case Meta-Analysis of NON-independent Effect Sizes #####
# affirmative studies: p < 0.05 and estimate in same direction as the pooled point estimate
# nonaffirmative studies: p >= 0.05 or estimate in the opposite direction
# meta-analyze only the nonaffirmative studies
d$affirm <- ( sign(d$logRR) == sign( c(muhat.naive.logRR) ) ) & ( abs( d$logRR / d$seilogRR ) > qnorm(.975) )
meta.worst <- robu( logRR ~ 1,
                    data = d[ d$affirm == FALSE, ],
                    studynum = cluster,
                    model = "HIER",
                    var.eff.size = varlogRR,
                    small = TRUE )
```

11.3.2 Extract worst-case meta-analysis results

```
muhat.worst <- meta.worst$b.r
muhat.lo.worst <- meta.worst$reg_table$CI.L
muhat.hi.worst <- meta.worst$reg_table$CI.U

round( matrix(
  transf.exp.int( c( muhat.worst, muhat.lo.worst, muhat.hi.worst ) ),
  nrow = 1,
  dimnames = list( "Risk ratio", c( "Worst-case estimate", "Lower 95% CI", "Upper 95% CI" ) )
  ), 2 )
```

```
# when there is only one nonaffirmative study
nonaffirmative.study <- d[ d$affirm == FALSE, ]
round( matrix(
  transf.exp.int( c( nonaffirmative.study$logRR,
                     nonaffirmative.study$logRR - qnorm(.975) * nonaffirmative.study$seilogRR,
                     nonaffirmative.study$logRR + qnorm(.975) * nonaffirmative.study$seilogRR ) ),
  nrow = 1,
  dimnames = list( "Risk ratio", c( "Worst-case estimate", "Lower 95% CI", "Upper 95% CI" ) )
  ), 2 )
```

11.4 S-value

```
##### Independent Effect Sizes #####
# If Selection Bias Favors Postive Estimates
svalue( yi = d$logRR, vi = d$varlogRR, q = log(1.0), model = "robust", favor.positive = TRUE, small = TRUE )
svalue( yi = d$logRR, vi = d$varlogRR, q = log(1.1), model = "robust", favor.positive = TRUE, small = TRUE )

# If Selection Bias Favors Negative Estimates
svalue( yi = d$logRR, vi = d$varlogRR, q = log(1.0), model = "robust", favor.positive = FALSE, small = TRUE )
svalue( yi = d$logRR, vi = d$varlogRR, q = log(0.9), model = "robust", favor.positive = FALSE, small = TRUE )
```

```
##### NON-independent Effect Sizes #####
# If Selection Bias Favors Postive Estimates
svalue( yi = d$logRR, vi = d$varlogRR, q = log(1.0), clustervar = d$cluster, model = "robust", favor.positive = T
RUE, small = TRUE )
svalue( yi = d$logRR, vi = d$varlogRR, q = log(1.1), clustervar = d$cluster, model = "robust", favor.positive = T
RUE, small = TRUE )

# If Selection Bias Favors Negative Estimates
svalue( yi = d$logRR, vi = d$varlogRR, q = log(1.0), clustervar = d$cluster, model = "robust", favor.positive = F
ALSE, small = TRUE )
svalue( yi = d$logRR, vi = d$varlogRR, q = log(0.9), clustervar = d$cluster, model = "robust", favor.positive = F
ALSE, small = TRUE )
```

12. Sensitivity Analysis for Unmeasured Confounding

12.1 E-value

```
##### If muhat.naive.logRR > 0 #####
evalues.RR( est = transf.exp.int(muhat.naive.logRR),
            lo = transf.exp.int(muhat.lo.naive.logRR),
            hi = transf.exp.int(muhat.hi.naive.logRR),
            true = 1 ) # shift to the null

evalues.RR( est = transf.exp.int(muhat.naive.logRR),
            lo = transf.exp.int(muhat.lo.naive.logRR),
            hi = transf.exp.int(muhat.hi.naive.logRR),
            true = 1.1 ) # shift to RR = 1.1
```

```
##### If muhat.naive.logRR < 0 #####
evalues.RR( est = transf.exp.int(muhat.naive.logRR),
            lo = transf.exp.int(muhat.lo.naive.logRR),
            hi = transf.exp.int(muhat.hi.naive.logRR),
            true = 1 ) # shift to the null

evalues.RR( est = transf.exp.int(muhat.naive.logRR),
            lo = transf.exp.int(muhat.lo.naive.logRR),
            hi = transf.exp.int(muhat.hi.naive.logRR),
            true = 0.9 ) # shift to RR = 0.9
```

12.2 That/Ghat

```
##### If muhat.naive.logRR > 0 #####
r <- 0.2 # for meta-analysis of 10-15 studies
# or
r <- 0.1 # for meta-analysis of >= 16 studies

d$calib.logRR <- calib_ests( yi = d$logRR, sei = d$seilogRR )
causative <- muhat.naive.logRR > 0

q.logRR <- log(1.0)
# or
q.logRR <- log(1.1)

# proportion of true effects more extreme than q.logRR as function of B
temp <- That_causal( .q = q.logRR,
                     .r = r,
                     .B.vec = seq( 1, 4, 0.01 ),
                     .calib = d$calib.logRR,
                     .tail = "above",
                     .causative = causative,

                     .give.CI = TRUE,
                     .R = 2000,
                     .dat = d,
                     .calib.name = "calib.logRR")


( That <- temp$Est[ temp$Stat == "That" ] )
( Ghat <- temp$Est[ temp$Stat == "Ghat" ] )
```

```
##### If muhat.naive.logRR < 0 #####
r <- 0.2 # for meta-analysis of 10-15 studies
# or
r <- 0.1 # for meta-analysis of >= 16 studies

q.logRR <- log(1.0)
# or
q.logRR <- log(0.9)

d$calib.logRR <- calib_ests( yi = d$logRR, sei = d$seilogRR )
causative <- muhat.naive.logRR > 0

# proportion of true effects more extreme than q.logRR as function of B
temp <- That_causal( .q = q.logRR,
                     .r = r,
                     .B.vec = seq( 1, 4, 0.01 ),
                     .calib = d$calib.logRR,
                     .tail = "below",
                     .causative = causative,

                     .give.CI = TRUE,
                     .R = 2000,
                     .dat = d,
                     .calib.name = "calib.logRR")


( That <- temp$Est[ temp$Stat == "That" ] )
( Ghat <- temp$Est[ temp$Stat == "Ghat" ] )
```
