# Supplementary material for: Menopausal hormone therapy and women’s health: An umbrella review
Source: PLoS Med. 2021 Aug 2;18(8):e1003731. doi: 10.1371/journal.pmed.1003731 (PMC8366967; doi:10.1371/journal.pmed.1003731)
Supplement: S1 Text — Results: Supplementary results for umbrella review on menopausal hormone therapy and women’s health. Table A: Search strategies used to retrieve papers from different databases. Table B: Characteristics of included systematic reviews and/or meta-analyses of randomized controlled trials on menopausal hormone therapy and multiple outcomes. Table C: Characteristics of included systematic reviews and/or meta-analyses of observational epidemiological studies on menopausal hormone therapy and multiple outcomes. Table D: Quality assessment of included systematic reviews and/or meta-analyses of randomized controlled trials on menopausal hormone therapy and multiple outcomes. Table E: Quality assessment of included systematic reviews and/or meta-analyses of observational epidemiological studies on menopausal hormone therapy and multiple outcomes. Table F: Any menopausal hormone therapy for primary prevention of multiple outcomes in included systematic reviews and meta-analyses of randomized controlled trials. Table G: Estrogen-alone therapy for primary prevention of multiple outcomes in included systematic reviews and meta-analyses of randomized controlled trials. Table H: Estrogen plus progestin therapy for primary prevention of multiple outcomes in included systematic reviews and meta-analyses of randomized controlled trials. Table I: Any menopausal hormone therapy for secondary prevention of multiple outcomes in included systematic reviews and meta-analyses of randomized controlled trials. Table J: Estrogen-alone therapy for secondary prevention of multiple outcomes in included systematic reviews and meta-analyses of randomized controlled trials. Table K: Estrogen plus progestin therapy for secondary prevention of multiple outcomes in included systematic reviews and meta-analyses of randomized controlled trials. Table L: Any menopausal hormone therapy for primary prevention of multiple outcomes in included systematic reviews and meta-analyses of observational epidemiologi [file pmed.1003731.s002.pdf]

Supplementary Online Content

CLICK on a section to jump to it

Results. Supplementary Results for Umbrella Review on Menopausal Hormone Therapy and Women's Health

Table A. Search Strategies Used to Retrieve Papers from Different Databases
Table B. Characteristics of Included Systematic Reviews and/or Meta-Analyses of Randomized Controlled Trials on Menopausal Hormone Therapy and Multiple Outcomes
Table C. Characteristics of Included Systematic Reviews and/or Meta-Analyses of Observational Epidemiological Studies on Menopausal Hormone Therapy and Multiple Outcomes
Table D. Quality Assessment of Included Systematic Reviews and/or Meta-Analyses of Randomized Controlled Trials on Menopausal Hormone Therapy and Multiple Outcomes
Table E. Quality Assessment of Included Systematic Reviews and/or Meta-Analyses of Observational Epidemiological Studies on Menopausal Hormone Therapy and Multiple Outcomes
Table F. Any Menopausal Hormone Therapy for Primary Prevention of Multiple Outcomes in Included Systematic Reviews and Meta-Analyses of Randomized Controlled Trials
Table G. Estrogen-Alone Therapy for Primary Prevention of Multiple Outcomes in Included Systematic Reviews and Meta-Analyses of Randomized Controlled Trials
Table H. Estrogen Plus Progestin Therapy for Primary Prevention of Multiple Outcomes in Included Systematic Reviews and Meta-Analyses of Randomized Controlled Trials
Table I. Any Menopausal Hormone Therapy for Secondary Prevention of Multiple Outcomes in Included Systematic Reviews and Meta-Analyses of Randomized Controlled Trials
Table J. Estrogen-Alone Therapy for Secondary Prevention of Multiple Outcomes in Included Systematic Reviews and Meta-Analyses of Randomized Controlled Trials
Table K. Estrogen Plus Progestin Therapy for Secondary Prevention of Multiple Outcomes in Included Systematic Reviews and Meta-Analyses of Randomized Controlled Trials
Table L. Any Menopausal Hormone Therapy for Primary Prevention of Multiple Outcomes in Included Systematic Reviews and Meta-Analyses of Observational Epidemiological Studies
Table M. Estrogen-Alone Therapy for Primary Prevention of Multiple Outcomes in Included Systematic Reviews and Meta-Analyses of Observational Epidemiological Studies
Table N. Estrogen Plus Progestin Therapy for Primary Prevention of Multiple Outcomes in Included Systematic Reviews and Meta-Analyses of Observational Epidemiological Studies
Table O. Any Menopausal Hormone Therapy for Secondary Prevention of Multiple Outcomes in Included Systematic Reviews and Meta-Analyses of Observational Epidemiological Studies
Table P. Estrogen-Alone Therapy for Secondary Prevention of Multiple Outcomes in Included Systematic Reviews and Meta-Analyses of Observational Epidemiological Studies
Table Q. Estrogen Plus Progestin Therapy for Secondary Prevention of Multiple Outcomes in Included Systematic Reviews and Meta-Analyses of Observational Epidemiological Studies
Table R. Assessment of Small-Study Effects and Publication Bias: Any Menopausal Hormone Therapy for Primary Prevention of Multiple Outcomes in Included Systematic Reviews and Meta-Analyses of Randomized Controlled Trials
Table S. Assessment of Small-Study Effects and Publication Bias: Any Menopausal Hormone Therapy for Secondary Prevention of Multiple Outcomes in Included Systematic Reviews and Meta-Analyses of Randomized Controlled Trials
Table T. Assessment of Small-Study Effects and Publication Bias: Any Menopausal Hormone Therapy for Primary Prevention of Multiple Outcomes in Included Systematic Reviews and Meta-Analyses of Observational Epidemiological Studies
Table U. Sensitivity Analysis for Residual Confounding: Any Menopausal Hormone Therapy for Primary Prevention of Multiple Outcomes in Included Systematic Reviews and Meta-Analyses of Observational Epidemiological Studies
Table V. Assessment of Small-Study Effects and Publication Bias: Any Menopausal Hormone Therapy for Secondary Prevention of Multiple Outcomes in Included Systematic Reviews and Meta-Analyses of Observational Epidemiological Studies
Table W. Sensitivity Analysis for Residual Confounding: Any Menopausal Hormone Therapy for Secondary Prevention of Multiple Outcomes in Included Systematic Reviews and Meta-Analyses of Observational Epidemiological Studies

Table X. Summary of Results for Outcomes with No Available Data for Meta-Analysis

Fig A. Pre-specified Protocol for Extracting Data from Included Systematic Reviews and/or Meta-Analyses of Randomized Controlled Trials on Menopausal Hormone Therapy and Multiple Outcomes
Fig B. Pre-specified Protocol for Extracting Data from Included Systematic Reviews and/or Meta-Analyses of Observational Epidemiological Studies on Menopausal Hormone Therapy and Multiple Outcomes
Fig C. Consistent or Highly Suggestive Evidence from Meta-Analyses of Randomized Controlled Trials on Menopausal Hormone Therapy and Multiple Surrogate Outcomes
Fig D. Suggestive Evidence from Meta-Analyses of Randomized Controlled Trials on Menopausal Hormone Therapy and Incidence of Diseases and Other Health Outcomes
Fig E. Suggestive Evidence from Meta-Analyses of Observational Epidemiological Studies on Menopausal Hormone Therapy and Incidence of Diseases and Other Health Outcomes

References

Results. Supplementary Results for Umbrella Review on Menopausal Hormone Therapy and Women's Health

Results for Outcomes with Suggestive or Controversial Evidence

Fig D and Fig E present the risk ratios (RRs) with 95% confidence intervals (CIs) for outcomes with suggestive evidence from meta-analyses of randomized controlled trials (RCTs) and of observational epidemiological studies, respectively. Below we described the meta-analysis results for outcomes with suggestive or controversial evidence.

Outcomes with suggestive evidence

Neoplasms. In RCTs, estrogen plus progestin therapy (EPT) was associated with a decreased risk of endometrial cancer (RR 0.67, 95% CI 0.50-0.92), but an increased risk of breast cancer (1.24, 95% CI 1.10-1.40). Estrogen-alone therapy (ET) was associated with a decreased risk of breast cancer (0.82, 95% CI 0.70-0.97). In observational studies, among breast and lung cancer survivors, women using menopausal hormone therapy (MHT) after diagnosis had a decreased risk of breast cancer recurrence (0.72, 95% CI 0.57-0.91), and women using MHT before diagnosis had improved lung cancer overall survival (0.77, 95% CI 0.65-0.92). ET and EPT were associated with increased risks of cutaneous melanoma (2.08, 95% CI 1.38-3.14) and endometrial cancer (1.71, 95% CI 1.57-1.86), respectively.

Diseases of the circulatory system. In RCTs, EPT was associated with an increased risk of pulmonary embolism (RR 1.28, 95% CI 1.02-1.61). In observational studies, MHT was associated with a decreased risk of coronary heart disease mortality (0.67, 95% CI 0.53-0.85).

Genitourinary system. In RCTs, intravaginal ET was associated with a decreased risk of recurrent urinary tract infection (RR 0.64, 95% CI 0.47-0.86). ET was associated with an increased risk of irregular vaginal bleeding (1.73, 95% CI 1.10-2.71).

Functioning assessment. In RCTs, EPT was associated with improved sleep quality (RR 0.92, 95% CI 0.89-0.95) and skeletal muscle strength (0.65, 95% CI 0.46-0.93).

Bone loss and fracture. In RCTs, EPT was associated with a decreased risk of hip fracture (RR 0.82, 95% CI 0.69-0.97).

Mental or behavioural disorders. In RCTs, EPT was associated with an increased risk of dementia (RR 1.97, 95% CI 1.16-3.33).

Diseases of the nervous system. In observational studies, MHT was associated with an increased risk of Parkinson's disease (RR 1.24, 95% CI 1.00-1.53).

Endocrine, nutritional or metabolic diseases. In observational studies, MHT was associated with a decreased risk of diabetes mellitus (RR 0.63, 95% CI 0.46-0.87).

Diseases of the immune system. In observational studies, MHT was associated with an increased risk of systemic lupus erythematosus (RR 1.90, 95% CI 1.16-3.10).

Others, not elsewhere classified. In observational studies, MHT was associated with decreased risks of cardiovascular disease incidence (RR 0.77, 95% CI 0.68-0.87) and mortality (0.66, 95% CI 0.48-0.90).

Outcomes with controversial evidence

In observational studies, contradictory evidence existed for breast cancer mortality, with predictive distribution containing a non-negligible proportion (> 30%) of strong beneficial (RR < 0.9) and harmful (> 1.1) effects. Controversial results were also found for pancreatic and lung cancer (Table L).

Small-Study Effects

In meta-analyses of RCTs, small-study effects were present for all fracture, sexual function, urinary incontinence and deep vein thrombosis (Table R and Table S). In meta-analyses of observational studies, small-

study effects were present for breast cancer specific survival and overall survival, and glioma (Table T and Table V).

Publication Bias

In meta-analyses of RCTs, meta-analysis results were robust to severe or extreme publication bias for vasomotor symptom, all fracture, stroke, non-fatal stroke, venous thromboembolism, gallbladder disease requiring surgery, and endometrial hyperplasia (ET) (Table R and Table S). In meta-analyses of observational studies, meta-analysis results were robust to severe or extreme publication bias for esophageal, gastric and colorectal cancer, breast cancer (EPT), breast cancer (ET), breast cancer specific survival and overall survival, endometrial cancer (ET), ovarian cancer incidence and overall survival, coronary heart disease, venous thromboembolism, asthma and cholelithiasis (Table T and Table V).

Residual Confounding in Meta-Analyses of Observational Studies

In general, the meta-analysis results were not robust to severe residual confounding. Using an arbitrary cutoff of RR 3.0, that is, the minimum confounding association strength that residual confounder(s) would need to have with both the exposure and outcome to explain away the meta-analysis results, only two outcomes (breast cancer and endometrial cancer) surpassed this threshold (Table U and Table W).

Table A. Search Strategies Used to Retrieve Papers from Different Databases

Databases	Date Searched	No. of Papers Retrieved	Search Terms Used for Search Strategies
EMBASE (OVID)	2017-11-26	7,698	1. hormone replacement therapy.mp. OR exp hormone substitution/<br>2. hormonal therapy.mp. OR exp hormonal therapy/<br>3. hormone replacement.mp.<br>4. hormone treatment.mp.<br>5. *conjugated estrogen/ OR *estradiol/ OR *estrogen/ OR combined hormone therapy.mp.<br>6. estrogen therapy.mp. OR exp estrogen therapy/<br>7. *medroxyprogesterone acetate/ OR *estradiol plus norethisterone acetate/ OR *conjugated estrogen plus medroxyprogesterone acetate/ OR estrogen-progestin therapy.mp. OR *bazedoxifene plus conjugated estrogen/<br>8. 1 OR 2 OR 3 OR 4 OR 5 OR 6 OR 7<br>9. exp meta analysis/<br>10. exp "systematic review"/<br>11. (systematic$ adj2 (review$ OR overview)).ti,ab.<br>12. (meta?anal$ OR meta anal$ OR meta-anal$ OR metaanal$ OR metanal$).ti,ab.<br>13. 9 OR 10 OR 11 OR 12<br>14. 8 AND 13
AMED (OVID)	2017-11-26	3	Same as EMBASE
Global Health (OVID)	2017-11-26	129	Same as EMBASE
PsycINFO (OVID)	2017-11-26	35	Same as EMBASE
CAB International (OVID)	2017-11-26	44	Same as EMBASE
Cochrane Library, Cochrane Database of Systematic Reviews	2017-11-26	893	1. hormone replacement therapy<br>2. estrogen therapy<br>3. estrogen-progestin<br>4. combined hormone therapy<br>5. systematic review<br>6. meta-analysis<br>7. 1 OR 2 OR 3 OR 4<br>8. 5 OR 6<br>9. 7 AND 8

Table A. Search Strategies Used to Retrieve Papers from Different Databases (continued)

Databases	Date Searched	No. of Papers Retrieved	Search Terms Used for Search Strategies
MEDLINE (OVID)	2017-11-24	1,100	1. hormone replacement therapy.mp. OR exp hormone replacement therapy/<br>2. exp estrogen replacement therapy/ OR hormone therapy.mp.<br>3. hormone treatment.mp.<br>4. estrogen therapy.mp.<br>5. hormone replacement.mp.<br>6. combined hormone therapy.mp.<br>7. "estrogens, conjugated (USP)"/ OR estrogen-progestin therapy.mp.<br>8. hormonal therapy.mp.<br>9. 1 OR 2 OR 3 OR 4 OR 5 OR 6 OR 7 OR 8<br>10. systematic review.mp.<br>11. (systematic$ adj2 (review$ OR overview)).ti,ab.<br>12. (systematic$ adj5 review$).tw,sh.<br>13. meta-analysis.mp. OR exp meta-analysis/<br>14. 10 OR 11 OR 12 OR 13<br>15. 9 AND 14
CINAHL	2017-11-26	26	1. (MH "Hormone Replacement Therapy+") OR (MH "Hormone Therapy+") OR (MM "Estrogens, Conjugated") OR "hormone replacement therapy OR estrogen therapy OR combined hormone therapy OR estrogen-progestin therapy"<br>2. (MM "Systematic Review") OR (MM "Meta Analysis") OR "systematic review OR meta-analysis"<br>3. 1 AND 2
ISI Web of Science, ISI Conference Proceedings Citation Index via Web of Knowledge<br><br>NOTE: Excluding MEDLINE database	2017-11-26	313	1. TS=hormone replacement therapy<br>2. TS=estrogen therapy<br>3. TS=combined hormone therapy<br>4. 1 OR 2 OR 3<br>5. TS=systematic review<br>6. TS=meta-analysis<br>7. 5 OR 6<br>8. 4 AND 7

Table A. Search Strategies Used to Retrieve Papers from Different Databases (continued)

Databases	Date Searched	No. of Papers Retrieved	Search Terms Used for Search Strategies
Database of Abstracts of Reviews of Effects (DARE)	2017-11-26	107	hormone replacement therapy AND systematic review
Google Scholar	2017-11-26	First 200 hits	1. "hormone replacement therapy" OR "estrogen therapy" OR "combined hormone therapy" OR "estrogen-progestin therapy" OR "hormone therapy"<br>2. "systematic review" OR "meta-analysis"<br>3. 1 AND 2
WHO Global Health Library<br><br>NOTE: Excluding MEDLINE database	2017-11-26	2	1. (tw:(hormone replacement therapy)) OR (tw:(estrogen therapy)) OR (tw:(combined hormone therapy)) OR (tw:(estrogen-progestin therapy)) OR (tw:(hormone therapy))<br>2. (tw:(systematic review)) OR (tw:(meta-analysis))<br>3. (instance:"ghl")<br>4. (db:("WPRIM" OR "IMEMR" OR "BBO" OR "WHOLIS") AND type_of_study:("systematic_reviews"))<br>5. 1 AND 2 AND 3 AND 4
Total	2017-11-26	10,550	
Total after de-duplication	2017-11-26	9,167	

Table B. Characteristics of Included Systematic Reviews and/or Meta-Analyses of Randomized Controlled Trials on Menopausal Hormone Therapy and Multiple Outcomes

Outcome[a]	Reference	Study Design	No. of Studies	Population	Intervention[b]	Comments
**Neoplasms**						
All cancer						
Incidence	Zhu 2016[1]	RCT	12	PM	E/EP	The review only searched for studies of MHT and fracture; we added the 2 WHI hormone therapy trials[2] missing from Zhu 2016 review
Mortality	Marjoribanks 2017[3]	DBRCT	5	PM	E/EP ≥12 mo[c]	We added 1 trial that was not included for mortality in Marjoribanks 2017 review; additionally included 1 trial from Benkhadra 2015[4]
Colorectal cancer						
Incidence	Marjoribanks 2017[3]	DBRCT	8	PM	E/EP ≥12 mo[c]	
Mortality	Marjoribanks 2017[3]	DBRCT	2	PM	E/EP ≥12 mo[c]	
Lung cancer						
Incidence	Marjoribanks 2017[3]	DBRCT	4	PM	E/EP ≥12 mo[c]	We added 1 trial that was not included for incidence in Marjoribanks 2017 review
Mortality	Marjoribanks 2017[3]	DBRCT	4	PM	E/EP ≥12 mo[c]	We added 1 trial that was not included for mortality in Marjoribanks 2017 review
Breast cancer						
Incidence	Marjoribanks 2017[3]	DBRCT	15	PPM	E/EP ≥12 mo[c]	
Recurrence	Col 2005[5]	RCT	4	PM with breast cancer	E/EP after diagnosis	We additionally included 1 trial from Marjoribanks 2017[3]
Mortality	Benkhadra 2015[4]	RCT	4	PM	E/EP	RCTs with follow-up of ≥6 mo; the review combined breast cancer mortality with breast cancer survival; we excluded 1 study; added 2 trials that were not included for mortality in Benkhadra 2015 review
Endometrial cancer	Marjoribanks 2017[3]	DBRCT	6	PPM	E/EP ≥12 mo[c]	

Table B. Characteristics of Included Systematic Reviews and/or Meta-Analyses of Randomized Controlled Trials on Menopausal Hormone Therapy and Multiple Outcomes (continued)

Outcome[a]	Reference	Study Design	No. of Studies	Population	Intervention[b]	Comments
Ovarian cancer						
Incidence	Marjoribanks 2017[3]	DBRCT	2	PM	E/EP ≥12 mo[c]	
Overall survival	Li 2015[6]	RCT	2	PM with ovarian cancer	E/EP after diagnosis	The review included only patients who had received surgical treatment for ovarian cancer
**Symptoms, signs or clinical findings of blood, blood-forming organs, or the immune system**						
C-reactive protein	Salpeter 2006[7]	RCT	12	PM without diabetes	E/EP ≥2 mo[d]	We excluded 3 studies
C-reactive protein	Casanova 2015[8]	RCT	4	Healthy PM	E/EP[e]	We excluded 1 study
Immunological factors	Abdi 2016[9]	Unclear	13	PM	Unclear	Unclear on the type of MHT and control; no meta-analysis conducted
PAI-1 antigen	Salpeter 2006[7]	RCT	13	PM without diabetes	E/EP ≥2 mo[d]	We excluded 3 studies
Protein C	Salpeter 2006[7]	RCT	Unclear	PM without diabetes	E/EP ≥2 mo[d]	
Protein S	Salpeter 2006[7]	RCT	Unclear	PM without diabetes	E/EP ≥2 mo[d]	
Fibrinogen	Salpeter 2006[7]	RCT	20	PM without diabetes	E/EP ≥2 mo[d]	We excluded 4 studies
E-selectin	Salpeter 2006[7]	RCT	7	PM without diabetes	E/EP ≥2 mo[d]	We excluded 1 study
**Endocrine, nutritional or metabolic diseases**						
Diabetes mellitus	Salpeter 2006[7]	RCT	Unclear	PM	E/EP ≥2 mo[d]	
**Symptoms, signs or clinical findings of endocrine, nutritional or metabolic diseases**						
Fasting glucose	Salpeter 2006[7]	RCT	Unclear	PM with diabetes	E/EP ≥2 mo[d]	
Fasting glucose	Xu 2014[10]	RCT	5	PM with diabetes	Oral EP	We excluded 1 study
Fasting glucose	Salpeter 2006[7]	RCT	Unclear	PM without diabetes	E/EP ≥2 mo[d]	
Fasting insulin	Salpeter 2006[7]	RCT	Unclear	PM with diabetes	E/EP ≥2 mo[d]	

Table B. Characteristics of Included Systematic Reviews and/or Meta-Analyses of Randomized Controlled Trials on Menopausal Hormone Therapy and Multiple Outcomes (continued)

Outcome[a]	Reference	Study Design	No. of Studies	Population	Intervention[b]	Comments
Fasting insulin	Salpeter 2006[7]	RCT	Unclear	PM without diabetes	E/EP ≥2 mo[d]	
Hemoglobin A1c	Xu 2014[10]	RCT	4	PM with diabetes	Oral EP	We excluded 1 study
Insulin resistance	Salpeter 2006[7]	RCT	Unclear	PM with diabetes	E/EP ≥2 mo[d]	
Insulin resistance	Salpeter 2006[7]	RCT	18	PM without diabetes	E/EP ≥2 mo[d]	
Total cholesterol	Casanova 2015[8]	RCT	11	Healthy PM	E/EP[e]	We excluded 1 study
Total cholesterol	Ramesh 2016[11]	RCT	2	PM with chronic kidney disease	E/EP	The review combined observational studies with RCTs; we excluded 3 studies
Total cholesterol	Xu 2014[10]	RCT	5	PM with diabetes	Oral EP	We excluded 2 studies
HDL cholesterol	Casanova 2015[8]	RCT	12	Healthy PM	E/EP[e]	We excluded 1 study
HDL cholesterol	Ramesh 2016[11]	RCT	2	PM with chronic kidney disease	E/EP	We excluded 2 studies
HDL cholesterol	Xu 2014[10]	RCT	5	PM with diabetes	Oral EP	We excluded 2 studies
HDL cholesterol	Salpeter 2006[7]	RCT	Unclear	PM without diabetes	E/EP ≥2 mo[d]	
LDL cholesterol	Casanova 2015[8]	RCT	11	Healthy PM	E/EP[e]	We excluded 1 study
LDL cholesterol	Ramesh 2016[11]	RCT	2	PM with chronic kidney disease	E/EP	We excluded 2 studies
LDL cholesterol	Xu 2014[10]	RCT	5	PM with diabetes	Oral EP	We excluded 2 studies
LDL cholesterol	Salpeter 2006[7]	RCT	Unclear	PM without diabetes	E/EP ≥2 mo[d]	
LDL/HDL ratio	Salpeter 2006[7]	RCT	55	PM without diabetes	E/EP ≥2 mo[d]	We excluded 6 studies
Lipoprotein (a)	Anagnostis 2017[12]	RCT	21	PM	E/EP	RCTs with follow-up of ≥8 wks; we excluded 3 studies
Triglyceride	Casanova 2015[8]	RCT	10	Healthy PM	E/EP[e]	We excluded 1 study
Triglyceride	Ramesh 2016[11]	RCT	2	PM with chronic kidney disease	E/EP	The review combined observational studies with RCTs; we excluded 3 studies

Table B. Characteristics of Included Systematic Reviews and/or Meta-Analyses of Randomized Controlled Trials on Menopausal Hormone Therapy and Multiple Outcomes (continued)

Outcome[a]	Reference	Study Design	No. of Studies	Population	Intervention[b]	Comments
Triglyceride	Xu 2014[10]	RCT	4	PM with diabetes	Oral EP	We excluded 2 studies
Triglyceride	Salpeter 2006[7]	RCT	52	PM without diabetes	E/EP ≥2 mo[d]	We excluded 2 studies
Body mass index	Kongnyuy 1999[13]	RCT	12	PM	E/EP ≥3 mo	
Body mass index	Casanova 2015[8]	RCT	2	Healthy PM	E/EP[e]	We excluded 1 study
Body mass index	Xu 2014[10]	RCT	2	PM with diabetes	Oral EP	
Body weight	Kongnyuy 1999[13]	RCT	18	PPM	E/EP ≥3 mo	
Lean body mass	Salpeter 2006[7]	RCT	Unclear	PM without diabetes	E/EP ≥2 mo[d]	
Abdominal fat	Salpeter 2006[7]	RCT	4	PM without diabetes	E/EP ≥2 mo[d]	
Waist circumference	Salpeter 2006[7]	RCT	5	PM without diabetes	E/EP ≥2 mo[d]	
Waist/hip ratio	Kongnyuy 1999[13]	RCT	2	PM	E/EP ≥3 mo	
Bone mineral density of lumbar spine	Wells 2002[14]	RCT	21	PM	E/EP	RCTs with follow-up of ≥1 y
Bone mineral density of lumbar spine	Rudic 2011[15]	RCT	2	PM with primary biliary cirrhosis	EP	
Bone mineral density of forearm	Wells 2002[14]	RCT	14	PM	E/EP	RCTs with follow-up of ≥1 y
Bone mineral density of femoral neck	Wells 2002[14]	RCT	9	PM	E/EP	RCTs with follow-up of ≥1 y
Bone mineral density of proximal femur	Rudic 2011[15]	RCT	2	PM with primary biliary cirrhosis	EP	
**Mental or behavioural disorders**						
Dementia (probable)	Marjoribanks 2017[3]	DBRCT	2	PM	E/EP ≥12 mo[c]	
**Mental or behavioural symptoms, signs or clinical findings**						
Depressive symptom	Whedon 2017[16]	RCT	10	PPM	E/EP[f]	RCTs with follow-up of ≥4 wks

Table B. Characteristics of Included Systematic Reviews and/or Meta-Analyses of Randomized Controlled Trials on Menopausal Hormone Therapy and Multiple Outcomes (continued)

Outcome[a]	Reference	Study Design	No. of Studies	Population	Intervention[b]	Comments
**Diseases of the nervous system**						
Cerebrovascular disease	Sare 2008[17]	RCT	28	PPM	E/EP	We additionally included 2 trials from Yang 2013[18] and Zhu 2016[1]
Stroke	Sare 2008[17]	RCT	19	PPM	E/EP	We excluded 1 study; additionally included 2 trials from Yang 2013[18] and Zhu 2016[1]
Fatal stroke	Sare 2008[17]	RCT	15	PPM	E/EP	We added 3 trials that were not included for fatal stroke in Sare 2008 review; additionally included 1 trial from Zhu 2016[1]
Non-fatal stroke	Sare 2008[17]	RCT	14	PPM	E/EP	We excluded 1 study; added 4 trials that were not included for non-fatal stroke in Sare 2008 review; additionally included 1 trial from Zhu 2016[1]
Transient ischaemic attack	Marjoribanks 2017[3]	DBRCT	7	PM	E/EP ≥12 mo[c]	
Alzheimer disease	O'Brien 2014[19]	RCT	2	PM	E/EP	
**Diseases of the circulatory system**						
Coronary heart disease						
Incidence	Sare 2008[17]	RCT	30	PPM	E/EP	We additionally included 5 trials from Yang 2013,[18] Zhu 2016,[1] and Marjoribanks 2017[3]
Mortality	Marjoribanks 2017[3]	DBRCT	10	PM	E/EP ≥12 mo[c]	
Myocardial infarction	Sare 2008[17]	RCT	24	PPM	E/EP	We additionally included 5 trials from Yang 2013,[18] Zhu 2016,[1] and Boardman 2015[20]
Fatal myocardial infarction	Sare 2008[17]	RCT	13	PM	E/EP	We additionally included 1 trial from Zhu 2016[1]
Non-fatal myocardial infarction	Sare 2008[17]	RCT	16	PM	E/EP	We additionally included 1 trial from Zhu 2016[1]
Angina pectoris						
Any angina	Boardman 2015[20]	RCT	5	PM	Oral E/EP	RCTs with follow-up of ≥6 mo
Unstable angina	Sare 2008[17]	RCT	5	PM	E/EP	

Table B. Characteristics of Included Systematic Reviews and/or Meta-Analyses of Randomized Controlled Trials on Menopausal Hormone Therapy and Multiple Outcomes (continued)

Outcome[a]	Reference	Study Design	No. of Studies	Population	Intervention[b]	Comments
Venous thromboembolism	Sare 2008[17]	RCT	30	PPM	E/EP	We additionally included 8 trials from Zhu 2016,[1] Canonico 2008,[21] and Marjoribanks 2017[3]
Deep vein thrombosis	Sare 2008[17]	RCT	16	PPM	E/EP	We additionally included 3 trials from Zhu 2016[1] and Canonico 2008[21]
Pulmonary embolism	Sare 2008[17]	RCT	12	PPM	E/EP	We additionally included 2 trials from Zhu 2016[1] and Canonico 2008[21]
**Symptoms, signs or clinical findings of the circulatory system**						
Cardiac death	Yang 2013[18]	RCT	9	PM	E/EP	The review only included trials that were primarily designed to investigate MHT and cardiovascular outcomes
Coronary revascularization	Boardman 2015[20]	RCT	6	PM	Oral E/EP	RCTs with follow-up of ≥6 mo
Mean blood pressure	Salpeter 2006[7]	RCT	Unclear	PM without diabetes	E/EP ≥2 mo[d]	
Systolic blood pressure	Casanova 2015[8]	RCT	6	Healthy PM	E/EP[e]	We excluded 1 study
Diastolic blood pressure	Casanova 2015[8]	RCT	6	Healthy PM	E/EP[e]	We excluded 1 study
**Diseases of the digestive system**						
Gallbladder disease requiring surgery	Marjoribanks 2017[3]	DBRCT	5	PM	E/EP ≥12 mo[c]	
**Diseases of the genitourinary system**						
Recurrent urinary tract infection	Perrotta 2008[22]	RCT	3	PM with recurrent urinary tract infection	Oral/vaginal E	We excluded 3 studies
Endometrial hyperplasia	Furness 2012[23]	RCT	17	PM	Oral E/EP ≥12 mo	
Vaginal atrophy	Lethaby 2016[24]	RCT	5	PM	Vaginal E ≥3 mo	

Table B. Characteristics of Included Systematic Reviews and/or Meta-Analyses of Randomized Controlled Trials on Menopausal Hormone Therapy and Multiple Outcomes (continued)

Outcome[a]	Reference	Study Design	No. of Studies	Population	Intervention[b]	Comments
Irregular vaginal bleeding	Lethaby 1999[25]	RCT	9	PM	E/EP ≥6 mo	
**Symptoms, signs or clinical findings of the genitourinary system**						
Vasomotor symptom	Maclennan 2004[26]	DBRCT	9	PPM	Oral E/EP ≥3 mo	
Vasomotor symptom severity	Maclennan 2004[26]	DBRCT	7	PPM	Oral E/EP ≥3 mo	
Urinary incontinence	Cody 2012[27]	RCT	13	PM with urinary incontinence	E/EP	
**Injury, poisoning or certain other consequences of external causes**						
All fracture	Zhu 2016[1]	RCT	30	PM	E/EP	We additionally included 2 trials from Marjoribanks 2017[3]
Vertebral fracture	Torgerson 2001[28]	RCT	16	PM	E/EP ≥12 mo	We additionally included 3 trials from Zhu 2016[1] and Marjoribanks 2017[3]
Nonvertebral fracture	Torgerson 2001[29]	RCT	26	PM	E/EP ≥12 mo	We additionally included 4 trials from Zhu 2016[1]
Hip fracture	Marjoribanks 2017[3]	DBRCT	6	PM	E/EP ≥12 mo[c]	
**Functioning assessment**						
Cognitive function	Lethaby 2008[30]	DBRCT	16	Healthy PM	E/EP ≥2 wks	
Cognitive function	Marjoribanks 2017[3]	DBRCT	5	PPM without major health problems	E/EP ≥12 mo[c]	
Cognitive function	Hogervorst 2009[31]	DBRCT	7	PM with dementia	E/EP ≥2 wks	
Sleep quality	Cintron 2017[32]	RCT	7	PM	E/EP ≥8 wks	The review only searched for studies published between 2002 and 2015
Sexual function	Nastri 2013[33]	RCT	10	PM	E/EP	RCTs with follow-up of ≥1 mo
Skeletal muscle strength	Greising 2009[34]	RCT	5	PM	E/EP	The review combined observational studies with RCTs; we excluded 18 studies

Table B. Characteristics of Included Systematic Reviews and/or Meta-Analyses of Randomized Controlled Trials on Menopausal Hormone Therapy and Multiple Outcomes (continued)

Outcome[a]	Reference	Study Design	No. of Studies	Population	Intervention[b]	Comments
**Others, not elsewhere classified**						
All-cause mortality	Benkhadra 2015[4]	RCT	38	PM	E/EP	RCTs with follow-up of ≥6 mo; we excluded 2 studies; additionally included 9 trials from Salpeter 2004[35] and Marjoribanks 2017[3]
Cardiovascular disease						
Incidence	Sare 2008[17]	RCT	36	PPM	E/EP	We additionally included 5 trials from Yang 2013[18] and Zhu 2016[1]
Mortality	Salpeter 2004[35]	RCT	18	PM	Transdermal or oral E/EP	RCTs with follow-up of ≥6 mo; we excluded 1 study; additionally included 4 trials from Benkhadra 2015[4]

Abbreviations: DBRCT, double-blinded randomized controlled trial; E, estrogen alone; EP, estrogen plus progestin; HDL, high-density lipoprotein; LDL, low-density lipoprotein; MHT, menopausal hormone therapy; PAI-1, plasminogen activator inhibitor-1; PM, postmenopausal women; PPM, peri-/post-menopausal women; RCT, randomized controlled trial; WHI, Women's Health Initiative.

[a] Incidence unless otherwise indicated.

[b] The comparator group is placebo or no treatment, unless otherwise indicated.

[c] Estrogen with/without progestin (oral/transdermal/subcutaneous/intranasal) for at least 12 months.

[d] Conjugated equine estrogen, oral esterified estrogen or transdermal estrogen, alone or in combination with progestin for at least 2 months.

[e] Low-dose estrogen (0.3 mg or less conjugated equine estrogen, 1 mg or less estradiol valerate or oral 17β-estradiol, 100 µg or less percutaneous 17β-estradiol gel, <50 µg 17β-estradiol patches, or <300 µg intranasal estradiol) with/without progestin.

[f] Bioidentical estrogen (17β-estradiol, estradiol acetate, estradiol hemihydrates, estriol, estrone, and estropipate, administered orally, transdermally, or vaginally) with/without progestin.

Table C. Characteristics of Included Systematic Reviews and/or Meta-Analyses of Observational Epidemiological Studies on Menopausal Hormone Therapy and Multiple Outcomes

Outcome[a]	Reference	Study Design	No. of Studies	Population	Exposure[b]	Comments
**Neoplasms**						
Cutaneous melanoma	Gandini 2011[36]	CO/CC	9	PPM	Any MHT	We excluded 2 studies
Head and neck cancer	McCarthy 2017[37]	CO/CC	3	PPM	Any MHT	No meta-analysis conducted
Glioma	Qi 2013[38]	CO/CC	10	PPM	Any MHT	The review only included CCs; we excluded 2 studies; added 4 COs that were not included for glioma in Qi 2013 review; additionally included 2 CCs from Benson 2015[39]
Meningioma	Qi 2013[40]	CO/CC	14	PPM	Any MHT	We excluded 1 CS; additionally included 1 CC from Benson 2015[39]
Thyroid cancer	Cao 2015[41]	CO/CC	12	PPM	Any MHT	We excluded 1 study
Esophageal cancer	Zhu 2017[42]	CO/CC	5	PPM	Any MHT	We excluded 1 study
Gastric cancer	Camargo 2012[43]	CO/CC	6	PPM	Any MHT	We excluded 1 study
Colorectal cancer	Green 2012[44]	CO/CC	27	PPM	Any MHT	We excluded 2 studies
Pancreatic cancer	Tang 2015[45]	CO/CC	11	PPM	Any MHT	We excluded 4 studies
Primary liver cancer	Zhong 2016[46]	CO/CC	5	PM	Any MHT	We excluded 2 studies
Lung cancer						
Incidence	Yao 2013[47]	CO/CC	20	PPM	Any MHT	The review pooled observational studies with RCTs using unadjusted effect estimates; we excluded 6 studies; additionally included 3 COs from Bae 2015[48]
Overall survival	Li 2017[49]	CO	5	PM with lung cancer	Any MHT before diagnosis	The review pooled lung cancer mortality and lung cancer-specific survival with overall survival; we excluded 3 studies
Breast cancer						
Incidence	Anothaisintawee 2013[50]	CO/CC	86	PPM	Any MHT	The review combined unadjusted effect estimates; we excluded 9 studies; added 7 studies that were not included for incidence in Anothaisintawee 2013 review; additionally included 10 studies from Wang 2017[51]

Table C. Characteristics of Included Systematic Reviews and/or Meta-Analyses of Observational Epidemiological Studies on Menopausal Hormone Therapy and Multiple Outcomes (continued)

Outcome[a]	Reference	Study Design	No. of Studies	Population	Exposure[b]	Comments
Breast cancer						
Recurrence	Col 2005[5]	CO	7	PPM with breast cancer	Any MHT after diagnosis	
Mortality	Yu 2017[52]	CO/CC	11	PPM	Any MHT	The review pooled breast cancer survival with breast cancer mortality; we excluded 2 studies
Specific survival	Yu 2017[52]	CO	21	PPM with breast cancer	Any MHT before/after diagnosis	The review pooled breast cancer-specific survival with breast cancer mortality; we added 2 COs that were not included for specific survival in Yu 2017 review; additionally included 1 CO from Col 2005[5]
Overall survival	Yu 2017[52]	CO	29	PPM with breast cancer	Any MHT before/after diagnosis	We excluded 1 study; added 2 COs that were not included for overall survival in Yu 2017 review; additionally included 1 CO from Col 2005[5]
Endometrial cancer						
Incidence	Grady 1995[53]	CO/CC	32	PPM	Any MHT	We excluded 16 studies; additionally included 11 COs from Sjogren 2016[54]
Recurrence	Shim 2014[55]	CO	4	PPM with endometrial cancer	Any MHT after diagnosis	The review combined cohort studies with RCTs; included only patients who had received surgical treatment for endometrial cancer; the included studies did not report adjusted effect estimates
Mortality	Grady 1995[53]	CO	3	PPM	Estrogen	
Ovarian cancer						
Incidence	Greiser 2007[56]	CO/CC	38	PPM	Any MHT	We excluded 5 studies; additionally included 6 COs from Zhou 2008[57] and Shi 2016[58]
Recurrence	Li 2015[6]	CO	3	PPM with ovarian cancer	Any MHT after diagnosis	The review included only patients who had received surgical treatment for ovarian cancer; we added 1 CO that was not included for recurrence in Li 2015 review
Overall survival	Li 2015[6]	CO	3	PPM with ovarian cancer	Any MHT after diagnosis	The review included only patients who had received surgical treatment for ovarian cancer; we excluded 1 study

Table C. Characteristics of Included Systematic Reviews and/or Meta-Analyses of Observational Epidemiological Studies on Menopausal Hormone Therapy and Multiple Outcomes (continued)

Outcome[a]	Reference	Study Design	No. of Studies	Population	Exposure[b]	Comments
**Diseases of the immune system**						
Systemic lupus erythematosus	Rojas-Villarraga 2014[59]	CO/CC	3	PPM	Any MHT	We excluded 1 study
**Endocrine, nutritional or metabolic diseases**						
Diabetes mellitus	Xu 2014[10]	CO	2	PM	Any MHT	We excluded 6 CSs
**Mental or behavioural disorders**						
Dementia	O'Brien 2014[19]	CO	5	PM	Any MHT	
**Diseases of the nervous system**						
Parkinson disease	Wang 2015[60]	CO/CC	9	PM	Any MHT	We excluded 5 studies
Alzheimer disease	LeBlanc 2001[61]	CO/CC	14	Healthy PM	Any MHT	We excluded 7 studies; additionally included 8 studies from O'Brien 2014[19] and Hogervorst 2000[62]
**Diseases of the visual system**						
Cataract	Lai 2013[63]	CO/CC	6	PM	Any MHT	We excluded 3 studies
**Diseases of the circulatory system**						
Coronary heart disease						
Incidence	Humphrey 2002[64]	CO/CC	12	PPM	Any MHT	The review only included high-quality studies
Mortality	Humphrey 2002[64]	CO/CC	5	PM	Any MHT	The review only included high-quality studies
Venous thromboembolism	Canonico 2008[21]	CO/CC	8	PPM	Any MHT	The review only included high-quality studies
Deep vein thrombosis	Canonico 2008[21]	CC	3	PM	Any MHT	The review only included high-quality studies
Pulmonary embolism	Canonico 2008[21]	CO/CC	3	PM	Any MHT	The review only included high-quality studies
**Diseases of the respiratory system**						
Asthma	McCleary 2018[65]	CO	5	PM	Any MHT	We excluded 3 studies
**Diseases of the digestive system**						
Cholelithiasis	Wang 2017[66]	CO/CC	12	PPM	Any MHT	The review combined observational studies with RCTs; we excluded 4 studies; added 2 COs that were not included for cholelithiasis in Wang 2017 review

Table C. Characteristics of Included Systematic Reviews and/or Meta-Analyses of Observational Epidemiological Studies on Menopausal Hormone Therapy and Multiple Outcomes (continued)

Outcome[a]	Reference	Study Design	No. of Studies	Population	Exposure[b]	Comments
**Diseases of the musculoskeletal system or connective tissue**						
Osteoarthritis	de Klerk 2009[67]	RCT/CO/CC/CS	19	PPM	Any MHT	No meta-analysis conducted
**Others, not elsewhere classified**						
All-cause mortality	Salpeter 2009[68]	CO/CC	8	PPM	Any MHT	
Cardiovascular disease						
Incidence	Humphrey 2002[64]	CO/CC	4	PPM	Any MHT	The review only included high-quality studies; we added 1 CO that was not included for incidence in Humphrey 2002 review
Mortality	Humphrey 2002[64]	CO	7	PPM	Any MHT	The review only included high-quality studies; we excluded 1 study

Abbreviations: CC, case-control study; CO, cohort study; CS, cross-sectional study; MHT, menopausal hormone therapy; PM, postmenopausal women; PPM, peri-/post-menopausal women; RCT, randomized controlled trial.

[a] Incidence unless otherwise indicated.

[b] “Any MHT”: any type of menopausal hormone therapy, such as estrogen alone, estrogen plus progestin, tibolone, selective estrogen receptor modulators, or unspecified; the comparator group is never use of hormone therapy, unless otherwise indicated.

Table D. Quality Assessment of Included Systematic Reviews and/or Meta-Analyses of Randomized Controlled Trials on Menopausal Hormone Therapy and Multiple Outcomes

		AMSTAR 2 Items[b]															
**Outcome[a]**	**Reference**	**1**	**2**	**3**	**4**	**5**	**6**	**7**	**8**	**9**	**10**	**11**	**12**	**13**	**14**	**15**	**16**
**Neoplasms**																	
All cancer																	
Incidence	Zhu 2016[1]	Y	N	Y	PY	Y	Y	N	PY	N	N	N	N	N	Y	N	Y
Mortality	Marjoribanks 2017[3]	Y	Y	Y	PY	Y	Y	Y	Y	Y	Y	Y	Y	Y	Y	Y	Y
Colorectal cancer																	
Incidence	Marjoribanks 2017[3]	Y	Y	Y	PY	Y	Y	Y	Y	Y	Y	Y	Y	Y	Y	Y	Y
Mortality	Marjoribanks 2017[3]	Y	Y	Y	PY	Y	Y	Y	Y	Y	Y	Y	Y	Y	Y	Y	Y
Lung cancer																	
Incidence	Marjoribanks 2017[3]	Y	Y	Y	PY	Y	Y	Y	Y	Y	Y	Y	Y	Y	Y	Y	Y
Mortality	Marjoribanks 2017[3]	Y	Y	Y	PY	Y	Y	Y	Y	Y	Y	Y	Y	Y	Y	Y	Y
Breast cancer																	
Incidence	Marjoribanks 2017[3]	Y	Y	Y	PY	Y	Y	Y	Y	Y	Y	Y	Y	Y	Y	Y	Y
Recurrence	Col 2005[5]	Y	N	Y	N	N	Y	Y	PY	N	N	N	N	Y	Y	N	Y
Mortality	Benkhadra 2015[4]	Y	PY	Y	PY	Y	Y	N	Y	Y	Y	N	N	Y	N	N	Y
Endometrial cancer	Marjoribanks 2017[3]	Y	Y	Y	PY	Y	Y	Y	Y	Y	Y	Y	Y	Y	Y	Y	Y
Ovarian cancer																	
Incidence	Marjoribanks 2017[3]	Y	Y	Y	PY	Y	Y	Y	Y	Y	Y	Y	Y	Y	Y	Y	Y
Overall survival	Li 2015[6]	Y	PY	Y	PY	Y	Y	N	PY	PY	N	N	Y	Y	Y	N	Y
**Symptoms, signs or clinical findings of blood, blood-forming organs, or the immune system**																	
C-reactive protein	Salpeter 2006[7]	Y	N	Y	N	N	N	N	PY	PY	N	N	Y	N	N	N	Y
C-reactive protein	Casanova 2015[8]	Y	PY	Y	N	Y	Y	N	PY	Y	N	N	N	N	Y	N	Y
Immunological factors	Abdi 2016[9]	N	N	N	N	N	N	N	N	N	N	NM	NM	N	N	NM	Y
PAI-1 antigen	Salpeter 2006[7]	Y	N	Y	N	N	N	N	PY	PY	N	N	Y	N	N	N	Y
Protein C	Salpeter 2006[7]	Y	N	Y	N	N	N	N	PY	PY	N	N	Y	N	Y	N	Y
Protein S	Salpeter 2006[7]	Y	N	Y	N	N	N	N	PY	PY	N	N	Y	N	N	N	Y
Fibrinogen	Salpeter 2006[7]	Y	N	Y	N	N	N	N	PY	PY	N	N	Y	N	Y	N	Y
E-selectin	Salpeter 2006[7]	Y	N	Y	N	N	N	N	PY	PY	N	N	Y	N	Y	N	Y

Table D. Quality Assessment of Included Systematic Reviews and/or Meta-Analyses of Randomized Controlled Trials on Menopausal Hormone Therapy and Multiple Outcomes (continued)

		AMSTAR 2 Items[b]															
**Outcome[a]**	**Reference**	**1**	**2**	**3**	**4**	**5**	**6**	**7**	**8**	**9**	**10**	**11**	**12**	**13**	**14**	**15**	**16**
**Endocrine, nutritional or metabolic diseases**																	
Diabetes mellitus	Salpeter 2006[7]	Y	N	Y	N	N	N	N	PY	PY	N	N	Y	N	Y	N	Y
**Symptoms, signs or clinical findings of endocrine, nutritional or metabolic diseases**																	
Fasting glucose[c]	Salpeter 2006[7]	Y	N	Y	N	N	N	N	PY	PY	N	N	Y	N	Y	N	Y
Fasting glucose	Xu 2014[10]	Y	N	Y	PY	Y	Y	N	Y	N	N	N	N	N	N	N	Y
Fasting glucose[d]	Salpeter 2006[7]	Y	N	Y	N	N	N	N	PY	PY	N	N	Y	N	Y	N	Y
Fasting insulin[c]	Salpeter 2006[7]	Y	N	Y	N	N	N	N	PY	PY	N	N	Y	N	Y	N	Y
Fasting insulin[d]	Salpeter 2006[7]	Y	N	Y	N	N	N	N	PY	PY	N	N	Y	N	Y	N	Y
HbA1c	Xu 2014[10]	Y	N	Y	PY	Y	Y	N	Y	N	N	N	N	N	N	N	Y
Insulin resistance[c]	Salpeter 2006[7]	Y	N	Y	N	N	N	N	PY	PY	N	N	Y	N	Y	N	Y
Insulin resistance[d]	Salpeter 2006[7]	Y	N	Y	N	N	N	N	PY	PY	N	N	Y	N	Y	N	Y
Total cholesterol	Casanova 2015[8]	Y	PY	Y	N	Y	Y	N	PY	Y	N	Y	N	N	N	N	Y
Total cholesterol	Ramesh 2016[11]	Y	Y	Y	Y	Y	Y	N	Y	PY	N	Y	N	N	Y	Y	Y
Total cholesterol	Xu 2014[10]	Y	N	Y	PY	Y	Y	N	Y	N	N	N	N	N	Y	N	Y
HDL cholesterol	Casanova 2015[8]	Y	PY	Y	N	Y	Y	N	PY	Y	N	Y	N	N	Y	N	Y
HDL cholesterol	Ramesh 2016[11]	Y	Y	Y	Y	Y	Y	N	Y	PY	N	Y	N	N	Y	Y	Y
HDL cholesterol	Xu 2014[10]	Y	N	Y	PY	Y	Y	N	Y	N	N	N	N	N	N	N	Y
HDL cholesterol	Salpeter 2006[7]	Y	N	Y	N	N	N	N	PY	PY	N	N	Y	N	Y	N	Y
LDL cholesterol	Casanova 2015[8]	Y	PY	Y	N	Y	Y	N	PY	Y	N	Y	N	N	Y	N	Y
LDL cholesterol	Ramesh 2016[11]	Y	Y	Y	Y	Y	Y	N	Y	PY	N	Y	N	N	Y	Y	Y
LDL cholesterol	Xu 2014[10]	Y	N	Y	PY	Y	Y	N	Y	N	N	N	N	N	N	N	Y
LDL cholesterol	Salpeter 2006[7]	Y	N	Y	N	N	N	N	PY	PY	N	N	Y	N	Y	N	Y
LDL/HDL ratio	Salpeter 2006[7]	Y	N	Y	N	N	N	N	PY	PY	N	N	Y	N	N	N	Y
Lipoprotein (a)	Anagnostis 2017[12]	Y	N	Y	N	Y	Y	N	PY	N	N	Y	N	N	N	N	Y
Triglyceride	Casanova 2015[8]	Y	PY	Y	N	Y	Y	N	PY	Y	N	Y	N	N	N	N	Y
Triglyceride	Ramesh 2016[11]	Y	Y	Y	Y	Y	Y	N	Y	PY	N	Y	N	N	Y	Y	Y
Triglyceride	Xu 2014[10]	Y	N	Y	PY	Y	Y	N	Y	N	N	N	N	N	Y	N	Y
Triglyceride	Salpeter 2006[7]	Y	N	Y	N	N	N	N	PY	PY	N	N	Y	N	N	N	Y

Table D. Quality Assessment of Included Systematic Reviews and/or Meta-Analyses of Randomized Controlled Trials on Menopausal Hormone Therapy and Multiple Outcomes (continued)

		AMSTAR 2 Items[b]															
**Outcome[a]**	**Reference**	**1**	**2**	**3**	**4**	**5**	**6**	**7**	**8**	**9**	**10**	**11**	**12**	**13**	**14**	**15**	**16**
Body mass index	Kongnyuy 1999[13]	Y	Y	Y	PY	N	Y	Y	Y	Y	Y	N	Y	Y	Y	N	Y
Body mass index	Casanova 2015[8]	Y	PY	Y	N	Y	Y	N	PY	Y	N	N	N	N	Y	N	Y
Body mass index	Xu 2014[10]	Y	N	Y	PY	Y	Y	N	Y	N	N	N	N	N	Y	N	Y
Body weight	Kongnyuy 1999[13]	Y	Y	Y	PY	N	Y	Y	Y	Y	Y	N	Y	Y	Y	N	Y
Lean body mass	Salpeter 2006[7]	Y	N	Y	N	N	N	N	PY	PY	N	N	Y	N	Y	N	Y
Abdominal fat	Salpeter 2006[7]	Y	N	Y	N	N	N	N	PY	PY	N	N	Y	N	Y	N	Y
Waist circumference	Salpeter 2006[7]	Y	N	Y	N	N	N	N	PY	PY	N	N	Y	N	Y	N	Y
Waist/hip ratio	Kongnyuy 1999[13]	Y	Y	Y	PY	N	Y	Y	Y	Y	Y	N	Y	Y	Y	N	Y
BMD of lumbar spine	Wells 2002[14]	Y	N	Y	PY	Y	Y	Y	PY	PY	N	N	N	N	N	Y	Y
BMD of lumbar spine	Rudic 2011[15]	Y	Y	Y	PY	Y	Y	Y	Y	Y	Y	Y	Y	Y	Y	Y	Y
BMD of forearm	Wells 2002[14]	Y	N	Y	PY	Y	Y	Y	PY	PY	N	N	N	N	N	Y	Y
BMD of femoral neck	Wells 2002[14]	Y	N	Y	PY	Y	Y	Y	PY	PY	N	N	N	N	N	Y	Y
BMD of proximal femur	Rudic 2011[15]	Y	Y	Y	PY	Y	Y	Y	Y	Y	Y	Y	Y	Y	Y	Y	Y
**Mental or behavioural disorders**																	
Dementia (probable)	Marjoribanks 2017[3]	Y	Y	Y	PY	Y	Y	Y	Y	Y	Y	Y	Y	Y	Y	Y	Y
**Mental or behavioural symptoms, signs or clinical findings**																	
Depressive symptom	Whedon 2017[16]	Y	PY	Y	PY	Y	Y	N	PY	Y	N	Y	N	Y	Y	Y	Y
**Diseases of the nervous system**																	
CeVD	Sare 2008[17]	Y	N	Y	N	N	Y	N	PY	PY	N	Y	Y	Y	Y	N	Y
Stroke	Sare 2008[17]	Y	N	Y	N	N	Y	N	PY	PY	N	Y	Y	Y	Y	N	Y
Fatal stroke	Sare 2008[17]	Y	N	Y	N	N	Y	N	PY	PY	N	Y	Y	Y	Y	N	Y
Non-fatal stroke	Sare 2008[17]	Y	N	Y	N	N	Y	N	PY	PY	N	Y	Y	Y	Y	N	Y
TIA	Marjoribanks 2017[3]	Y	Y	Y	PY	Y	Y	Y	Y	Y	Y	Y	Y	Y	Y	Y	Y
Alzheimer disease	O'Brien 2014[19]	Y	N	Y	PY	N	N	N	PY	N	N	N	Y	Y	Y	N	Y
**Diseases of the circulatory system**																	
CHD																	
Incidence	Sare 2008[17]	Y	N	Y	N	N	Y	N	PY	PY	N	Y	Y	N	Y	N	Y
Mortality	Marjoribanks 2017[3]	Y	Y	Y	PY	Y	Y	Y	Y	Y	Y	Y	Y	Y	Y	Y	Y

Table D. Quality Assessment of Included Systematic Reviews and/or Meta-Analyses of Randomized Controlled Trials on Menopausal Hormone Therapy and Multiple Outcomes (continued)

		AMSTAR 2 Items[b]															
**Outcome[a]**	**Reference**	**1**	**2**	**3**	**4**	**5**	**6**	**7**	**8**	**9**	**10**	**11**	**12**	**13**	**14**	**15**	**16**
MI	Sare 2008[17]	Y	N	Y	N	N	Y	N	PY	PY	N	Y	Y	N	Y	N	Y
Fatal MI	Sare 2008[17]	Y	N	Y	N	N	Y	N	PY	PY	N	Y	Y	N	Y	N	Y
Non-fatal MI	Sare 2008[17]	Y	N	Y	N	N	Y	N	PY	PY	N	Y	Y	N	Y	N	Y
Angina pectoris																	
Any angina	Boardman 2015[20]	Y	Y	Y	PY	Y	Y	Y	Y	Y	Y	Y	N	Y	Y	Y	Y
Unstable angina	Sare 2008[17]	Y	N	Y	N	N	Y	N	PY	PY	N	Y	Y	N	Y	N	Y
VTE	Sare 2008[17]	Y	N	Y	N	N	Y	N	PY	PY	N	Y	Y	N	Y	N	Y
Deep vein thrombosis	Sare 2008[17]	Y	N	Y	N	N	Y	N	PY	PY	N	Y	Y	N	Y	N	Y
Pulmonary embolism	Sare 2008[17]	Y	N	Y	N	N	Y	N	PY	PY	N	Y	Y	N	Y	N	Y
**Symptoms, signs or clinical findings of the circulatory system**																	
Cardiac death	Yang 2013[18]	Y	N	Y	N	Y	Y	N	Y	PY	N	Y	N	N	Y	N	Y
CR	Boardman 2015[20]	Y	Y	Y	PY	Y	Y	Y	Y	Y	Y	Y	N	Y	Y	Y	Y
Mean blood pressure	Salpeter 2006[7]	Y	N	Y	N	N	N	N	PY	PY	N	N	Y	N	N	N	Y
Systolic blood pressure	Casanova 2015[8]	Y	PY	Y	N	Y	Y	N	PY	Y	N	Y	N	N	N	N	Y
Diastolic blood pressure	Casanova 2015[8]	Y	PY	Y	N	Y	Y	N	PY	Y	N	Y	N	N	Y	N	Y
**Diseases of the digestive system**																	
Gallbladder disease[e]	Marjoribanks 2017[3]	Y	Y	Y	PY	Y	Y	Y	Y	Y	Y	Y	Y	Y	Y	Y	Y
**Diseases of the genitourinary system**																	
RUTI	Perrotta 2008[22]	Y	Y	Y	PY	Y	Y	Y	Y	PY	N	Y	N	N	Y	N	Y
Endometrial hyperplasia	Furness 2012[23]	Y	Y	Y	PY	Y	Y	Y	Y	Y	Y	N	Y	Y	Y	Y	Y
Vaginal atrophy	Lethaby 2016[24]	Y	Y	Y	Y	Y	Y	Y	Y	Y	Y	Y	Y	Y	Y	Y	Y
Irregular vaginal bleeding	Lethaby 1999[25]	Y	Y	Y	PY	N	Y	Y	Y	PY	Y	N	Y	Y	Y	N	Y
**Symptoms, signs or clinical findings of the genitourinary system**																	
VMS	Maclennan 2004[26]	Y	Y	Y	Y	N	Y	Y	Y	PY	Y	Y	Y	Y	N	Y	Y
VMS severity	Maclennan 2004[26]	Y	Y	Y	Y	N	Y	Y	Y	PY	Y	Y	Y	Y	Y	Y	Y
Urinary incontinence	Cody 2012[27]	Y	Y	Y	PY	Y	Y	Y	PY	PY	N	Y	N	Y	Y	N	Y
**Injury, poisoning or certain other consequences of external causes**																	
All fracture	Zhu 2016[1]	Y	N	Y	PY	Y	Y	N	PY	N	N	N	N	N	Y	N	Y

Table D. Quality Assessment of Included Systematic Reviews and/or Meta-Analyses of Randomized Controlled Trials on Menopausal Hormone Therapy and Multiple Outcomes (continued)

		AMSTAR 2 Items[b]															
**Outcome[a]**	**Reference**	**1**	**2**	**3**	**4**	**5**	**6**	**7**	**8**	**9**	**10**	**11**	**12**	**13**	**14**	**15**	**16**
Vertebral fracture	Torgerson 2001[28]	Y	N	Y	PY	N	Y	N	PY	PY	N	Y	N	N	Y	N	Y
Nonvertebral fracture	Torgerson 2001[29]	Y	N	Y	PY	N	Y	N	PY	PY	N	Y	Y	Y	Y	Y	Y
Hip fracture	Marjoribanks 2017[3]	Y	Y	Y	PY	Y	Y	Y	Y	Y	Y	Y	Y	Y	Y	Y	Y
**Functioning assessment**																	
Cognitive function	Lethaby 2008[30]	Y	Y	Y	N	Y	Y	Y	Y	PY	N	N	Y	Y	Y	N	Y
Cognitive function	Marjoribanks 2017[3]	Y	Y	Y	PY	Y	Y	Y	Y	Y	Y	Y	Y	Y	Y	Y	Y
Cognitive function	Hogervorst 2009[31]	Y	Y	Y	Y	Y	N	Y	Y	PY	N	N	Y	Y	Y	Y	Y
Sleep quality	Cintron 2017[32]	Y	PY	Y	PY	Y	Y	N	Y	Y	N	Y	Y	Y	Y	N	Y
Sexual function	Nastri 2013[33]	Y	Y	Y	PY	Y	Y	Y	Y	Y	Y	N	Y	Y	N	Y	Y
Skeletal muscle strength	Greising 2009[34]	Y	N	Y	N	N	N	N	PY	N	N	N	Y	N	Y	Y	Y
**Others, not elsewhere classified**																	
All-cause mortality	Benkhadra 2015[4]	Y	PY	Y	PY	Y	Y	N	Y	Y	Y	N	N	Y	Y	N	Y
CaVD																	
Incidence	Sare 2008[17]	Y	N	Y	N	N	Y	N	PY	PY	N	Y	Y	N	Y	N	Y
Mortality	Salpeter 2004[35]	Y	N	Y	PY	Y	Y	N	PY	PY	N	Y	Y	N	Y	N	Y

Abbreviations: AMSTAR, A MeaSurement Tool to Assess systematic Reviews; BMD, bone mineral density; CaVD, cardiovascular disease; CeVD, cerebrovascular disease; CHD, coronary heart disease; CR, coronary revascularization; HbA1c, hemoglobin A1c; HDL, high-density lipoprotein; LDL, low-density lipoprotein; MI, myocardial infarction; PAI-1, plasminogen activator inhibitor-1; RUTI, recurrent urinary tract infection; TIA, transient ischaemic attack; VMS, vasomotor symptom; VTE, venous thromboembolism.

[a] Incidence unless otherwise indicated.

[b] The revised AMSTAR (AMSTAR 2) has 13 items for systematic reviews and 16 items for meta-analyses.

[c] Postmenopausal women with diabetes.

[d] Postmenopausal women without diabetes.

[e] Gallbladder disease requiring surgery.

1 – Did the research questions and inclusion criteria for the review include the components of PICO? (yes/no)
2 – Did the report of the review contain an explicit statement that the review methods were established prior to the conduct of the review and did the report justify any significant deviations from the protocol? (yes/partial yes/no)
3 – Did the review authors explain their selection of the study designs for inclusion in the review? (yes/no)
4 – Did the review authors use a comprehensive literature search strategy? (yes/partial yes/no)
5 – Did the review authors perform study selection in duplicate? (yes/no)
6 – Did the review authors perform data extraction in duplicate? (yes/no)
7 – Did the review authors provide a list of excluded studies and justify the exclusions? (yes/partial yes/no)
8 – Did the review authors describe the included studies in adequate detail? (yes/partial yes/no)

9 – Did the review authors use a satisfactory technique for assessing the risk of bias (RoB) in individual studies that were included in the review? (yes/partial yes/no)
10 – Did the review authors report on the sources of funding for the studies included in the review? (yes/no)
11 – If meta-analysis was performed, did the review authors use appropriate methods for statistical combination of results? (yes/no/no meta-analysis conducted)
12 – If meta-analysis was performed, did the review authors assess the potential impact of RoB in individual studies on the results of the meta-analysis or other evidence synthesis? (yes/no/no meta-analysis conducted)
13 – Did the review authors account for RoB in individual studies when interpreting/discussing the results of the review? (yes/no)
14 – Did the review authors provide a satisfactory explanation for, and discussion of, any heterogeneity observed in the results of the review? (yes/no)
15 – If they performed quantitative synthesis, did the review authors carry out an adequate investigation of publication bias (small study bias) and discuss its likely impact on the results of the review? (yes/no/no meta-analysis conducted)
16 – Did the review authors report any potential sources of conflict of interest, including any funding they received for conducting the review? (yes/no)

(green)	Yes (Y)
(yellow)	Partial Yes (PY)
(red)	No (N)
(grey)	No meta-analysis conducted (NM)

Table E. Quality Assessment of Included Systematic Reviews and/or Meta-Analyses of Observational Epidemiological Studies on Menopausal Hormone Therapy and Multiple Outcomes

		AMSTAR 2 Items[b]															
**Outcome[a]**	**Reference**	**1**	**2**	**3**	**4**	**5**	**6**	**7**	**8**	**9**	**10**	**11**	**12**	**13**	**14**	**15**	**16**
**Neoplasms**																	
Cutaneous melanoma	Gandini 2011[36]	Y	N	Y	PY	N	Y	N	PY	N	N	Y	N	N	Y	N	Y
Head and neck cancer	McCarthy 2017[37]	Y	N	Y	N	Y	N	N	PY	PY	N	NM	NM	Y	Y	NM	Y
Glioma	Qi 2013[38]	Y	N	N	PY	N	Y	Y	PY	PY	N	Y	N	Y	Y	N	Y
Meningioma	Qi 2013[40]	Y	N	Y	PY	N	Y	Y	PY	N	N	Y	N	Y	Y	Y	Y
Thyroid cancer	Cao 2015[41]	Y	N	Y	PY	N	Y	N	PY	N	N	N	N	N	Y	N	Y
Esophageal cancer	Zhu 2017[42]	Y	N	N	PY	N	N	N	PY	PY	N	N	N	N	Y	N	Y
Gastric cancer	Camargo 2012[43]	Y	N	N	N	Y	Y	Y	PY	N	N	Y	N	Y	Y	N	Y
Colorectal cancer	Green 2012[44]	Y	N	Y	PY	N	N	N	N	N	N	N	N	N	N	N	Y
Pancreatic cancer	Tang 2015[45]	Y	N	N	PY	N	Y	Y	PY	N	N	Y	Y	Y	Y	Y	Y
Primary liver cancer	Zhong 2016[46]	Y	N	N	PY	N	Y	Y	PY	PY	N	Y	N	N	N	N	Y
Lung cancer																	
Incidence	Yao 2013[47]	Y	N	Y	N	Y	Y	N	PY	PY	N	N	N	N	Y	N	Y
Overall survival	Li 2017[49]	Y	N	Y	N	Y	N	N	PY	PY	N	N	N	Y	Y	N	Y
Breast cancer																	
Incidence	Anothaisintawee 2013[50]	Y	N	Y	PY	N	N	N	PY	N	N	N	N	N	N	N	Y
Recurrence	Col 2005[5]	Y	N	Y	N	N	Y	Y	PY	N	N	N	N	Y	Y	N	Y
Mortality	Yu 2017[52]	Y	N	Y	PY	Y	Y	Y	PY	PY	N	Y	Y	N	N	Y	Y
Specific survival	Yu 2017[52]	Y	N	Y	PY	Y	Y	Y	PY	PY	N	Y	Y	N	N	Y	Y
Overall survival	Yu 2017[52]	Y	N	Y	PY	Y	Y	Y	PY	PY	N	Y	Y	N	N	Y	Y
Endometrial cancer																	
Incidence	Grady 1995[53]	Y	N	Y	N	N	N	Y	PY	N	N	N	N	Y	N	N	N
Recurrence	Shim 2014[55]	Y	N	Y	PY	N	Y	Y	Y	N	N	N	N	Y	Y	Y	Y
Mortality	Grady 1995[53]	Y	N	Y	N	N	N	Y	PY	N	N	N	N	Y	N	N	N
Ovarian cancer																	
Incidence	Greiser 2007[56]	Y	N	Y	PY	Y	Y	Y	Y	N	N	N	N	N	N	N	Y
Recurrence	Li 2015[6]	Y	PY	Y	PY	Y	Y	N	PY	PY	N	N	Y	Y	Y	N	Y
Overall survival	Li 2015[6]	Y	PY	Y	PY	Y	Y	N	PY	PY	N	N	Y	Y	Y	N	Y

Table E. Quality Assessment of Included Systematic Reviews and/or Meta-Analyses of Observational Epidemiological Studies on Menopausal Hormone Therapy and Multiple Outcomes (continued)

		AMSTAR 2 Items[b]															
**Outcome[a]**	**Reference**	**1**	**2**	**3**	**4**	**5**	**6**	**7**	**8**	**9**	**10**	**11**	**12**	**13**	**14**	**15**	**16**
**Diseases of the immune system**																	
SLE	Rojas-Villarraga 2014[59]	Y	N	Y	PY	Y	Y	N	PY	N	N	Y	N	N	Y	N	Y
**Endocrine, nutritional or metabolic diseases**																	
Diabetes mellitus	Xu 2014[10]	Y	N	N	PY	Y	Y	N	PY	N	N	N	N	N	Y	N	Y
**Mental or behavioural disorders**																	
Dementia	O'Brien 2014[19]	Y	N	Y	PY	N	N	N	Y	N	N	N	N	Y	N	N	Y
**Diseases of the nervous system**																	
Parkinson disease	Wang 2015[60]	Y	N	N	PY	N	Y	N	N	PY	N	Y	Y	Y	N	Y	Y
Alzheimer disease	LeBlanc 2001[61]	Y	N	Y	PY	N	N	N	Y	PY	N	Y	Y	Y	Y	N	Y
**Diseases of the visual system**																	
Cataract	Lai 2013[63]	Y	N	Y	PY	N	Y	Y	PY	PY	N	N	N	Y	Y	N	Y
**Diseases of the circulatory system**																	
CHD																	
Incidence	Humphrey 2002[64]	Y	N	Y	PY	Y	N	N	PY	PY	N	N	Y	Y	Y	N	Y
Mortality	Humphrey 2002[64]	Y	N	Y	PY	Y	N	N	PY	PY	N	N	Y	Y	Y	N	Y
VTE	Canonico 2008[21]	Y	N	Y	N	N	Y	N	PY	PY	N	N	Y	Y	Y	N	Y
Deep vein thrombosis	Canonico 2008[21]	Y	N	Y	N	N	Y	N	PY	PY	N	N	Y	Y	Y	N	Y
Pulmonary embolism	Canonico 2008[21]	Y	N	Y	N	N	Y	N	PY	PY	N	N	Y	Y	Y	N	Y
**Diseases of the respiratory system**																	
Asthma	McCleary 2018[65]	Y	PY	Y	Y	Y	Y	N	PY	PY	N	Y	N	Y	N	Y	Y
**Diseases of the digestive system**																	
Cholelithiasis	Wang 2017[66]	Y	N	N	PY	N	Y	N	PY	PY	N	N	Y	N	Y	N	Y
**Diseases of the musculoskeletal system or connective tissue**																	
Osteoarthritis	de Klerk 2009[67]	Y	N	Y	PY	Y	Y	N	PY	N	Y	NM	NM	Y	N	NM	Y
**Others, not elsewhere classified**																	
All-cause mortality	Salpeter 2009[68]	Y	N	Y	N	Y	Y	N	N	N	N	Y	N	N	Y	N	Y

Table E. Quality Assessment of Included Systematic Reviews and/or Meta-Analyses of Observational Epidemiological Studies on Menopausal Hormone Therapy and Multiple Outcomes (continued)

		AMSTAR 2 Items[b]															
**Outcome[a]**	**Reference**	**1**	**2**	**3**	**4**	**5**	**6**	**7**	**8**	**9**	**10**	**11**	**12**	**13**	**14**	**15**	**16**
CaVD																	
Incidence	Humphrey 2002[64]	Y	N	Y	PY	Y	N	N	PY	PY	N	N	Y	Y	Y	N	Y
Mortality	Humphrey 2002[64]	Y	N	Y	PY	Y	N	N	PY	PY	N	N	Y	Y	Y	N	Y

Abbreviations: AMSTAR, A MeaSurement Tool to Assess systematic Reviews; CaVD, cardiovascular disease; CHD, coronary heart disease; SLE, systemic lupus erythematosus; VTE, venous thromboembolism.

[a] Incidence unless otherwise indicated.

[b] The revised AMSTAR (AMSTAR 2) has 13 items for systematic reviews and 16 items for meta-analyses.

1 – Did the research questions and inclusion criteria for the review include the components of PICO? (yes/no)
2 – Did the report of the review contain an explicit statement that the review methods were established prior to the conduct of the review and did the report justify any significant deviations from the protocol? (yes/partial yes/no)
3 – Did the review authors explain their selection of the study designs for inclusion in the review? (yes/no)
4 – Did the review authors use a comprehensive literature search strategy? (yes/partial yes/no)
5 – Did the review authors perform study selection in duplicate? (yes/no)
6 – Did the review authors perform data extraction in duplicate? (yes/no)
7 – Did the review authors provide a list of excluded studies and justify the exclusions? (yes/partial yes/no)
8 – Did the review authors describe the included studies in adequate detail? (yes/partial yes/no)
9 – Did the review authors use a satisfactory technique for assessing the risk of bias (RoB) in individual studies that were included in the review? (yes/partial yes/no)
10 – Did the review authors report on the sources of funding for the studies included in the review? (yes/no)
11 – If meta-analysis was performed, did the review authors use appropriate methods for statistical combination of results? (yes/no/no meta-analysis conducted)
12 – If meta-analysis was performed, did the review authors assess the potential impact of RoB in individual studies on the results of the meta-analysis or other evidence synthesis? (yes/no/no meta-analysis conducted)
13 – Did the review authors account for RoB in individual studies when interpreting/discussing the results of the review? (yes/no)
14 – Did the review authors provide a satisfactory explanation for, and discussion of, any heterogeneity observed in the results of the review? (yes/no)
15 – If they performed quantitative synthesis, did the review authors carry out an adequate investigation of publication bias (small study bias) and discuss its likely impact on the results of the review? (yes/no/no meta-analysis conducted)
16 – Did the review authors report any potential sources of conflict of interest, including any funding they received for conducting the review? (yes/no)

Yes (Y)
Partial Yes (PY)
No (N)
No meta-analysis conducted (NM)

Table F. Any Menopausal Hormone Therapy for Primary Prevention of Multiple Outcomes in Included Systematic Reviews and Meta-Analyses of Randomized Controlled Trials[a]

Outcome[b]	No. of Trials	No. of Events/Total No.		Metric (unit)	Most Precise Study[c]	Summary Effect[d]	*P*-Value[e]	T[f]	95% PI[g]	Proportion of True Effects (θ) Below/Above a Threshold (%)[h]			
		Any MHT	Control							$\widehat{P}(\theta < q)$	$\widehat{P}(\theta <$ null$)$	$\widehat{P}(\theta >$ null$)$	$\widehat{P}(\theta > q^{*})$
**Neoplasms**													
All cancer													
Incidence	12	2,180/19,724	2,112/18,918	RR	1.04 (0.97, 1.12)	1.01 (0.70, 1.45)	8.9×10^{-1}	0	N/A	N/A	N/A	N/A	N/A
Mortality	5	1,185/17,796	1,124/17,476	RR	1.05 (0.95, 1.17)	1.03 (0.80, 1.34)	4.4×10^{-1}	0	N/A	N/A	N/A	N/A	N/A
Colorectal cancer													
Incidence	8	268/18,687	289/17,867	RR	0.80 (0.63, 1.01)	0.91 (0.46, 1.77)	5.4×10^{-1}	0	N/A	N/A	N/A	N/A	N/A
Mortality	2	100/13,816	90/13,531	RR	1.01 (0.69, 1.48)	1.09 (0.36, 3.28)	4.9×10^{-1}	0	N/A	N/A	N/A	N/A	N/A
Lung cancer													
Incidence	4	366/15,266	325/14,986	RR	1.13 (0.93, 1.38)	1.10 (0.74, 1.63)	2.9×10^{-1}	0	N/A	N/A	N/A	N/A	N/A
Mortality	4	208/15,266	184/14,986	RR	1.10 (0.88, 1.39)	1.09 (0.98, 1.22)	6.2×10^{-2}	0	N/A	N/A	N/A	N/A	N/A
Breast cancer													
Incidence	15	910/20,742	813/19,430	RR	1.24 (1.10, 1.40)	1.05 (0.71, 1.57)	7.0×10^{-1}	0.18	0.75 to 1.29	13 (0, 100)	33 (0, 100)	67 (0, 100)	47 (0, 100)
Mortality	3	104/15,196	99/14,914	RR	1.28 (0.90, 1.82)	1.03 (0.06, 17.26)	9.5×10^{-1}	0.44	N/A	N/A	N/A	N/A	N/A
Endometrial cancer	6	76/11,278	106/10,026	RR	0.67 (0.50, 0.92)	0.65 (0.17, 2.44)	1.7×10^{-1}	0	N/A	N/A	N/A	N/A	N/A
Ovarian cancer	2	53/8,829	42/8,422	RR	1.23 (0.82, 1.85)	1.21 (0.15, 9.77)	4.6×10^{-1}	0	N/A	N/A	N/A	N/A	N/A
**Symptoms, signs or clinical findings of blood, blood-forming organs, or the immune system**													
C-reactive protein[i]	12	1,444	627	MD (% change)	27.10 (6.20, 48.00)	42.28 (18.14, 66.41)	3.9×10^{-3}	20.99	-1.98 to 77.31	N/A	0 (N/A, N/A)	100 (50, 100)	N/A
C-reactive protein[j]	4	142	156	MD (mg/L)	0.50 (-0.13, 1.13)	0.35 (-1.27, 1.96)	3.3×10^{-1}	0	N/A	N/A	N/A	N/A	N/A
				RR	0.96 (0.71, 1.30)	1.06 (0.71, 1.59)	5.9×10^{-1}	0.13	N/A	N/A	N/A	N/A	N/A
PAI-1 antigen	13	1,323	655	MD (% change)	-19.10 (-35.20, -3.00)	-32.19 (-44.38, -20.01)	4.7×10^{-4}	6.52	-51.53 to -20.47	N/A	100 (N/A, N/A)	0 (N/A, N/A)	N/A
Fibrinogen	20	2,303	905	MD (% change)	-4.40 (-7.00, -1.80)	-5.43 (-7.36, -3.49)	9.3×10^{-4}	0	N/A	N/A	N/A	N/A	N/A
E-selectin	7	985	352	MD (% change)	-19.00 (-25.30, -12.70)	-17.63 (-26.74, -8.51)	1.6×10^{-2}	0	N/A	N/A	N/A	N/A	N/A
**Symptoms, signs or clinical findings of endocrine, nutritional or metabolic diseases**													
Insulin resistance	18	2,430	1,207	MD (% change)	-12.40 (-20.90, -3.90)	-14.17 (-21.94, -6.40)	3.9×10^{-3}	1.87	-20.15 to -11.12	N/A	100 (N/A, N/A)	0 (N/A, N/A)	N/A
Total cholesterol[j]	11	599	565	MD (mg/dL)	-18.53 (-27.17, -9.89)	-13.20 (-19.69, -6.72)	1.4×10^{-3}	5.99	-25.38 to 3.38	N/A	100 (55, 100)	0 (N/A, N/A)	N/A
				RR	1.06 (0.81, 1.38)	0.68 (0.56, 0.83)	1.7×10^{-3}	0.20	0.46 to 1.14	91 (0, 100)	100 (55, 100)	0 (N/A, N/A)	0 (N/A, N/A)
Total cholesterol[k]	2	44	43	MD (mg/dL)	9.00 (-12.39, 30.39)	8.59 (-4.45, 21.64)	7.6×10^{-2}	0	N/A	N/A	N/A	N/A	N/A
				RR	1.20 (0.77, 1.87)	1.17 (0.67, 2.05)	1.7×10^{-1}	0	N/A	N/A	N/A	N/A	N/A
Total cholesterol[l]	5	125	132	MD (mmol/L)	-0.46 (-0.84, -0.08)	-0.39 (-0.78, -0.01)	4.8×10^{-2}	0.09	N/A	N/A	N/A	N/A	N/A
HDL cholesterol[j]	12	631	620	MD (mg/dL)	1.54 (-2.62, 5.70)	1.41 (-3.56, 6.37)	5.4×10^{-1}	7.26	-10.19 to 24.18	N/A	42 (0, 67)	58 (17, 83)	N/A
				RR	1.17 (0.90, 1.53)	1.10 (0.78, 1.54)	5.5×10^{-1}	0.39	0.53 to 8.51	33 (0, 58)	42 (0, 67)	58 (11, 83)	50 (17, 83)
HDL cholesterol[k]	2	44	43	MD (mg/dL)	12.30 (5.22, 19.38)	11.68 (-6.43, 29.79)	7.7×10^{-2}	0	N/A	N/A	N/A	N/A	N/A
				RR	2.15 (1.36, 3.40)	1.95 (0.24, 15.88)	1.5×10^{-1}	0	N/A	N/A	N/A	N/A	N/A
HDL cholesterol[l]	5	125	132	MD (mmol/L)	0.01 (-0.14, 0.16)	-0.05 (-0.17, 0.07)	3.3×10^{-1}	0.01	N/A	N/A	N/A	N/A	N/A
LDL cholesterol[j]	11	600	566	MD (mg/dL)	-3.60 (-12.75, 5.55)	-13.16 (-18.66, -7.67)	6.1×10^{-4}	2.88	-19.93 to -6.05	N/A	100 (N/A, N/A)	0 (N/A, N/A)	N/A
				RR	0.90 (0.69, 1.17)	0.70 (0.60, 0.82)	8.0×10^{-4}	0.14	0.45 to 0.97	100 (55, 100)	100 (N/A, N/A)	0 (N/A, N/A)	0 (N/A, N/A)
LDL cholesterol[k]	2	44	43	MD (mg/dL)	-8.00 (-26.48, 10.48)	-7.10 (-34.28, 20.07)	1.9×10^{-1}	0	N/A	N/A	N/A	N/A	N/A
				RR	0.83 (0.53, 1.29)	0.86 (0.36, 2.04)	2.7×10^{-1}	0	N/A	N/A	N/A	N/A	N/A
LDL cholesterol[l]	5	124	128	MD (mmol/L)	-0.18 (-0.53, 0.17)	-0.22 (-0.42, -0.01)	4.2×10^{-2}	0	N/A	N/A	N/A	N/A	N/A
LDL/HDL ratio	55	6,362	4,377	MD (% change)	-17.00 (-19.30, -14.70)	-16.25 (-18.83, -13.68)	4.0×10^{-15}	5.57	-30.46 to -4.67	N/A	100 (93, 98)	0 (N/A, N/A)	N/A
Lipoprotein (a)	39	1,989	1,970	MD (% change)	-2.71 (-3.06, -2.36)	-21.20 (-28.78, -13.62)	1.1×10^{-5}	18.23	-77.87 to 2.47	N/A	95 (79, 97)	5 (0, 15)	N/A
Triglyceride[j]	10	551	514	MD (mg/dL)	3.40 (-12.97, 19.77)	-4.49 (-18.84, 9.87)	4.9×10^{-1}	17.07	-51.80 to 24.98	N/A	60 (0, 90)	40 (0, 100)	N/A
				RR	1.06 (0.81, 1.38)	0.91 (0.68, 1.20)	4.5×10^{-1}	0.31	0.33 to 1.54	30 (0, 70)	60 (0, 90)	40 (0, 100)	20 (0, 100)
Triglyceride[k]	2	44	43	MD (mg/dL)	20.00 (-19.01, 59.01)	21.08 (-8.20, 50.36)	6.9×10^{-2}	0	N/A	N/A	N/A	N/A	N/A
				RR	1.25 (0.80, 1.95)	1.26 (1.17, 1.34)	1.5×10^{-2}	0	N/A	N/A	N/A	N/A	N/A
Triglyceride[l]	4	106	110	MD (mmol/L)	-0.23 (-0.62, 0.16)	-0.11 (-0.65, 0.43)	4.0×10^{-1}	0	N/A	N/A	N/A	N/A	N/A
Triglyceride[i]	52	6,008	4,214	MD (% change)	9.20 (7.30, 11.10)	2.39 (-0.69, 5.48)	1.2×10^{-1}	4.87	-12.67 to 10.94	N/A	25 (0, 77)	75 (0, 100)	N/A
Body mass index[m]	12	12,862	13,363	MD (kg/m^2)	0.00 (-0.20, 0.20)	-0.11 (-0.48, 0.26)	3.2×10^{-1}	0	N/A	N/A	N/A	N/A	N/A
				RR	1.00 (0.97, 1.03)	0.99 (0.93, 1.05)	3.7×10^{-1}	0	N/A	N/A	N/A	N/A	N/A
Body mass index[j]	2	84	77	MD (kg/m^2)	0.00 (-1.23, 1.23)	0.17 (-7.00, 7.35)	8.1×10^{-1}	0	N/A	N/A	N/A	N/A	N/A

				RR	1.00 (0.74, 1.36)	1.08 (0.13, 9.27)	7.3×10^{-1}	0.10	N/A	N/A	N/A	N/A	N/A
Body mass index[l]	2	73	74	MD (kg/m^2)	-0.79 (-2.81, 1.23)	-1.05 (-6.67, 4.57)	2.5×10^{-1}	0	N/A	N/A	N/A	N/A	N/A
Body weight	22	14,486	14,073	MD (kg)	0.30 (-0.27, 0.87)	-0.03 (-0.67, 0.60)	9.0×10^{-1}	0.36	-1.56 to 1.18	N/A	59 (0, 100)	41 (0, 100)	N/A
				RR	1.02 (0.99, 1.05)	1.00 (0.95, 1.05)	1.0×10^{-0}	0.01	0.92 to 1.08	0 (N/A, N/A)	59 (0, 100)	41 (0, 100)	0 (N/A, N/A)
Abdominal fat	4	136	85	MD (% change)	-5.80 (-11.50, -0.10)	-6.89 (-25.53, 11.75)	1.7×10^{-1}	0	N/A	N/A	N/A	N/A	N/A
Waist circumference	5	3,873	3,164	MD (% change)	-0.70 (-1.10, -0.30)	-0.82 (-4.04, 2.39)	2.0×10^{-1}	0	N/A	N/A	N/A	N/A	N/A
Waist/hip ratio	2	209	195	MD	0.00 (-0.02, 0.02)	0.00 (0.00, 0.00)	N/A	0	N/A	N/A	N/A	N/A	N/A
				RR	1.00 (0.82, 1.21)	1.00 (1.00, 1.00)	N/A	0	N/A	N/A	N/A	N/A	N/A
BMD of lumbar spine[n]	2	13	23	MD (g/cm^2/year)	2.10 (-0.73, 4.93)	1.26 (-11.48, 14.00)	4.3×10^{-1}	0	N/A	N/A	N/A	N/A	N/A
				RR	1.02 (0.46, 2.23)	1.42 (0.01, 231.76)	5.4×10^{-1}	0.33	N/A	N/A	N/A	N/A	N/A
BMD of proximal femur[n]	2	13	23	MD (g/cm^2/year)	2.30 (0.67, 3.93)	2.24 (0.60, 3.89)	3.7×10^{-2}	0	N/A	N/A	N/A	N/A	N/A
				RR	1.45 (0.66, 3.22)	2.16 (0.01, 845.14)	3.5×10^{-1}	0.46	N/A	N/A	N/A	N/A	N/A
**Mental or behavioural disorders**													
Dementia (probable)	2	68/3,693	40/3,786	RR	1.97 (1.16, 3.33)	1.74 (0.30, 9.97)	1.6×10^{-1}	0	N/A	N/A	N/A	N/A	N/A
**Mental or behavioural symptoms, signs or clinical findings**													
Depressive symptom	5	437	389	SMD	0.10 (-0.09, 0.29)	-0.13 (-0.64, 0.38)	5.1×10^{-1}	0.37	N/A	N/A	N/A	N/A	N/A
				RR	1.09 (0.92, 1.30)	0.89 (0.56, 1.41)	5.1×10^{-1}	0.34	N/A	N/A	N/A	N/A	N/A
**Diseases of the nervous system**													
Cerebrovascular disease	21	553/21,664	430/20,617	RR	1.37 (1.08, 1.73)	1.25 (1.04, 1.50)	3.0×10^{-2}	0	N/A	N/A	N/A	N/A	N/A
Stroke	17	785/19,090	661/18,182	RR	1.15 (0.99, 1.33)	1.17 (1.05, 1.29)	2.7×10^{-2}	0	N/A	N/A	N/A	N/A	N/A
Fatal stroke	6	333/17,633	303/17,268	RR	1.11 (0.90, 1.37)	1.08 (0.63, 1.83)	4.2×10^{-1}	0	N/A	N/A	N/A	N/A	N/A
Non-fatal stroke	11	299/16,732	217/15,924	RR	1.37 (1.04, 1.81)	1.35 (1.08, 1.69)	2.5×10^{-2}	0	N/A	N/A	N/A	N/A	N/A
Transient ischaemic attack	6	54/3,098	60/2,564	RR	0.80 (0.51, 1.23)	0.88 (0.30, 2.60)	5.0×10^{-1}	0	N/A	N/A	N/A	N/A	N/A
Alzheimer disease	2	33/3,693	21/3,786	RR	1.72 (0.84, 3.51)	1.61 (0.58, 4.46)	1.1×10^{-1}	0	N/A	N/A	N/A	N/A	N/A
**Diseases of the circulatory system**													
Coronary heart disease													
Incidence	17	1,063/20,261	1,021/19,187	RR	1.08 (0.95, 1.22)	1.02 (0.82, 1.26)	7.5×10^{-1}	0	N/A	N/A	N/A	N/A	N/A
Mortality	6	566/14,952	581/14,659	RR	1.04 (0.88, 1.21)	0.96 (0.46, 2.00)	6.7×10^{-1}	0	N/A	N/A	N/A	N/A	N/A
MI	15	714/19,486	658/18,592	RR	1.14 (0.99, 1.32)	1.06 (0.65, 1.75)	4.8×10^{-1}	0	N/A	N/A	N/A	N/A	N/A
Fatal MI	6	3/1,029	8/1,018	RR	0.19 (0.02, 1.65)	0.52 (0.12, 2.14)	2.7×10^{-1}	0	N/A	N/A	N/A	N/A	N/A
Non-fatal MI	8	327/17,635	300/16,805	RR	0.91 (0.73, 1.13)	1.06 (0.34, 3.30)	7.4×10^{-1}	0	N/A	N/A	N/A	N/A	N/A
Angina pectoris													
Any angina	2	269/13,816	297/13,531	RR	0.97 (0.79, 1.20)	0.90 (0.26, 3.07)	4.6×10^{-1}	0.07	N/A	N/A	N/A	N/A	N/A
Unstable angina	1	3/2,196	0/2,189	RR	6.98 (0.36, 135.01)	N/A	N/A	N/A	N/A	N/A	N/A	N/A	N/A
Venous thromboembolism	23	479/21,830	321/20,462	RR	1.04 (0.84, 1.29)	1.60 (0.99, 2.58)	5.2×10^{-2}	0.24	1.03 to 2.99	0 (N/A, N/A)	0 (N/A, N/A)	100 (78, 100)	96 (0, 100)
Deep vein thrombosis	14	405/19,906	310/19,017	RR	1.25 (1.02, 1.53)	1.39 (0.68, 2.84)	1.9×10^{-1}	0.19	1.01 to 2.38	0 (N/A, N/A)	0 (N/A, N/A)	100 (N/A, N/A)	93 (0, 100)
Pulmonary embolism	9	309/19,465	236/18,426	RR	1.28 (1.02, 1.61)	1.26 (0.81, 1.94)	1.1×10^{-1}	0	N/A	N/A	N/A	N/A	N/A
**Symptoms, signs or clinical findings of the circulatory system**													
Cardiac death	4	104/14,264	107/13,969	RR	0.94 (0.65, 1.35)	0.96 (0.67, 1.39)	6.3×10^{-1}	0	N/A	N/A	N/A	N/A	N/A
Coronary revascularization	3	912/13,927	869/13,642	RR	1.02 (0.91, 1.16)	1.03 (0.90, 1.18)	2.0×10^{-1}	0	N/A	N/A	N/A	N/A	N/A
Systolic blood pressure	6	341	306	MD (mmHg)	-0.63 (-3.55, 2.29)	0.84 (-5.67, 7.36)	7.5×10^{-1}	5.19	N/A	N/A	N/A	N/A	N/A
				RR	0.95 (0.75, 1.21)	1.07 (0.65, 1.75)	7.4×10^{-1}	0.40	N/A	N/A	N/A	N/A	N/A
Diastolic blood pressure	6	341	306	MD (mmHg)	1.90 (-0.45, 4.25)	-0.31 (-2.94, 2.33)	7.6×10^{-1}	1.18	N/A	N/A	N/A	N/A	N/A
				RR	1.05 (0.82, 1.33)	0.95 (0.75, 1.22)	6.3×10^{-1}	0.12	N/A	N/A	N/A	N/A	N/A
**Diseases of the digestive system**													
Gallbladder disease[o]	5	536/13,734	316/12,792	RR	1.78 (1.42, 2.24)	1.63 (1.31, 2.04)	1.1×10^{-2}	0	N/A	N/A	N/A	N/A	N/A
**Diseases of the genitourinary system**													
Endometrial hyperplasia	23	405/6,038	17/2,089	RR	37.00 (9.30, 147.27)	2.70 (1.15, 6.38)	2.8×10^{-2}	1.09	0.38 to 44.62	13 (0, 26)	13 (0, 26)	87 (61, 100)	87 (63, 96)
Irregular vaginal bleeding	9	151/558	39/289	RR	1.73 (1.10, 2.71)	1.78 (0.77, 4.10)	1.4×10^{-1}	0.54	N/A	N/A	N/A	N/A	N/A
**Injury, poisoning or certain other consequences of external causes**													
All fracture	30	2,024/22,226	2,546/20,962	RR	0.82 (0.76, 0.89)	0.72 (0.62, 0.84)	1.7×10^{-3}	0.10	0.58 to 0.87	100 (83, 100)	100 (N/A, N/A)	0 (N/A, N/A)	0 (N/A, N/A)
Vertebral fracture	16	206/17,478	284/16,858	RR	0.78 (0.60, 1.01)	0.69 (0.50, 0.94)	3.3×10^{-2}	0	N/A	N/A	N/A	N/A	N/A
Nonvertebral fracture	26	965/13,728	1,164/12,193	RR	0.79 (0.72, 0.87)	0.76 (0.62, 0.94)	2.5×10^{-2}	0.11	0.60 to 1.02	96 (0, 100)	100 (62, 100)	0 (N/A, N/A)	0 (N/A, N/A)

Hip fracture	6	392/17,799	449/17,502	RR	0.82 (0.69, 0.97)	0.85 (0.57, 1.29)	1.6×10^{-1}	0	N/A	N/A	N/A	N/A	N/A
**Functioning assessment**													
Sleep quality	8	7,964	7,484	SMD	-0.09 (-0.12, -0.06)	-0.13 (-0.42, 0.16)	2.5×10^{-1}	0.18	N/A	N/A	N/A	N/A	N/A
				RR	0.92 (0.89, 0.95)	0.89 (0.67, 1.17)	2.6×10^{-1}	0.17	N/A	N/A	N/A	N/A	N/A
Sexual function	10	1,531	1,295	SMD	-0.12 (-0.23, 0.00)	-0.21 (-0.37, -0.05)	1.6×10^{-2}	0.16	-0.61 to 0.23	40 (0, 60)	80 (0, 100)	20 (0, 50)	0 (N/A, N/A)
				RR	0.90 (0.81, 1.00)	0.82 (0.71, 0.96)	1.7×10^{-2}	0.15	0.57 to 1.28	70 (0, 90)	80 (30, 100)	20 (0, 50)	10 (0, 70)
Skeletal muscle strength	5	158	164	SMD	0.10 (-0.28, 0.48)	-0.46 (-1.13, 0.22)	1.3×10^{-1}	0.50	N/A	N/A	N/A	N/A	N/A
				RR	1.10 (0.78, 1.55)	0.66 (0.36, 1.22)	1.3×10^{-1}	0.45	N/A	N/A	N/A	N/A	N/A
**Others, not elsewhere classified**													
All-cause mortality	38	4,274/24,707	4,234/23,050	RR	1.01 (0.96, 1.07)	0.99 (0.83, 1.18)	7.2×10^{-1}	0	N/A	N/A	N/A	N/A	N/A
Cardiovascular disease													
Incidence	22	1,219/20,144	939/18,936	RR	1.13 (0.99, 1.29)	1.29 (0.99, 1.68)	5.6×10^{-2}	0.14	1.02 to 1.61	0 (N/A, N/A)	0 (N/A, N/A)	100 (41, 100)	86 (0, 100)
Mortality	9	1,249/17,142	1,246/16,703	RR	1.02 (0.92, 1.13)	0.96 (0.59, 1.57)	6.0×10^{-1}	0.11	N/A	N/A	N/A	N/A	N/A

Abbreviations: BMD, bone mineral density; HDL, high-density lipoprotein; LDL, low-density lipoprotein; MD, mean difference; MHT, menopausal hormone therapy; MI, myocardial infarction; N/A, not available or not applicable; PAI-1, plasminogen activator inhibitor-1; PI, prediction interval; RR, risk ratio; SMD, standardized mean difference.

[a] Primary prevention refers to reducing the risk of occurrence of a disease among individuals who do not have that disease.

[b] Incidence unless otherwise indicated.

[c] Point estimate with 95% confidence interval of the study with the smallest standard error in each meta-analysis.

[d] Point estimate with 95% confidence interval of robust random-effects meta-analysis.

[e] *P*-value of robust random-effects meta-analysis.

[f] The estimated standard deviation of true effects (for MD and SMD, T is on its original scale; for RR, T is on log RR scale).

[g] The middle 95% area of the estimated effect distribution. 95% PI is reported only in meta-analyses of ≥ 10 studies. More information about how to interpret 95% PI can be found in S3 Text (section 3).

[h] The proportion with 95% confidence interval of true effects (θ) below or above a threshold of scientific importance; complementary to 95% PI, these metrics estimate the area of the lower and upper tails of the effect distribution. For MD, null = 0; for SMD, q = -0.2, null = 0, q^* = 0.2; for RR, q = 0.9, null = 1.0, q^* = 1.1. These metrics are reported only in meta-analyses of ≥ 10 studies. More information about how to interpret them can be found in S3 Text (section 3).

[i] In postmenopausal women without diabetes.

[j] In healthy postmenopausal women who used low-dose menopausal hormone therapy.

[k] In postmenopausal women with chronic kidney disease.

[l] In postmenopausal women with diabetes.

[m] In postmenopausal women who used menopausal hormone therapy for at least 3 months.

[n] In postmenopausal women with primary biliary cirrhosis.

[o] Gallbladder disease requiring surgery.

Table G. Estrogen-Alone Therapy for Primary Prevention of Multiple Outcomes in Included Systematic Reviews and Meta-Analyses of Randomized Controlled Trials[a]

Outcome[b]	No. of Trials	No. of Events/Total No.		Metric (unit)	Most Precise Study[c]	Summary Effect[d]	*P*-Value[e]	T[f]	95% PI[g]	Proportion of True Effects (θ) Below/Above a Threshold (%)[h]			
		ET	Control							$\hat{P}\ (\theta < q)$	$\hat{P}\ (\theta < \text{null})$	$\hat{P}\ (\theta > \text{null})$	$\hat{P}\ (\theta > q^{*})$
**Neoplasms**													
All cancer													
Incidence	5	693/6,494	753/6,228	RR	0.94 (0.85, 1.03)	0.94 (0.83, 1.07)	1.0×10^{-1}	0	N/A	N/A	N/A	N/A	N/A
Mortality	1	424/5,310	439/5,429	RR	0.99 (0.87, 1.12)	N/A	N/A	N/A	N/A	N/A	N/A	N/A	N/A
Colorectal cancer													
Incidence	2	100/5,485	91/5,603	RR	1.14 (0.86, 1.51)	1.13 (0.29, 4.44)	4.7×10^{-1}	0	N/A	N/A	N/A	N/A	N/A
Mortality	1	47/5,310	40/5,429	RR	1.20 (0.79, 1.83)	N/A	N/A	N/A	N/A	N/A	N/A	N/A	N/A
Lung cancer													
Incidence	1	109/5,310	114/5,429	RR	0.98 (0.75, 1.27)	N/A	N/A	N/A	N/A	N/A	N/A	N/A	N/A
Mortality	1	34/5,310	33/5,429	RR	1.05 (0.65, 1.70)	N/A	N/A	N/A	N/A	N/A	N/A	N/A	N/A
Breast cancer													
Incidence	7	247/6,537	302/6,472	RR	0.82 (0.70, 0.97)	0.83 (0.43, 1.61)	1.8×10^{-1}	0	N/A	N/A	N/A	N/A	N/A
Mortality	1	30/5,310	46/5,429	RR	0.67 (0.42, 1.05)	N/A	N/A	N/A	N/A	N/A	N/A	N/A	N/A
Endometrial cancer	1	0/119	1/119	RR	0.33 (0.01, 8.10)	N/A	N/A	N/A	N/A	N/A	N/A	N/A	N/A
**Symptoms, signs or clinical findings of blood, blood-forming organs, or the immune system**													
C-reactive protein[i]	2	45	27	MD (% change)	62.60 (-10.80, 136.00)	70.90 (-135.24, 277.05)	1.4×10^{-1}	0	N/A	N/A	N/A	N/A	N/A
C-reactive protein[j]	1	8	15	MD (mg/L)	0.50 (-0.13, 1.13)	N/A	N/A	N/A	N/A	N/A	N/A	N/A	N/A
				RR	2.30 (1.02, 5.19)	N/A	N/A	N/A	N/A	N/A	N/A	N/A	N/A
PAI-1 antigen	2	28	25	MD (% change)	-28.00 (-78.00, 22.00)	-39.96 (-197.42, 117.50)	1.9×10^{-1}	0	N/A	N/A	N/A	N/A	N/A
Fibrinogen	2	30	28	MD (% change)	-15.80 (-32.00, 0.40)	-5.53 (-140.76, 129.71)	7.0×10^{-1}	12.27	N/A	N/A	N/A	N/A	N/A
E-selectin	1	30	14	MD (% change)	-4.50 (-38.00, 29.00)	N/A	N/A	N/A	N/A	N/A	N/A	N/A	N/A
**Symptoms, signs or clinical findings of endocrine, nutritional or metabolic diseases**													
Insulin resistance	6	170	151	MD (% change)	-12.40 (-20.90, -3.90)	-18.97 (-44.88, 6.95)	9.0×10^{-2}	7.51	N/A	N/A	N/A	N/A	N/A
Total cholesterol[j]	3	106	119	MD (mg/dL)	2.00 (-7.74, 11.74)	0.44 (-40.97, 41.85)	9.3×10^{-1}	0	N/A	N/A	N/A	N/A	N/A
				RR	1.06 (0.81, 1.38)	1.00 (0.41, 2.45)	9.9×10^{-1}	0	N/A	N/A	N/A	N/A	N/A
Total cholesterol[k]	1	11	11	MD (mg/dL)	6.00 (-48.03, 60.03)	N/A	N/A	N/A	N/A	N/A	N/A	N/A	N/A
				RR	1.09 (0.51, 2.32)	N/A	N/A	N/A	N/A	N/A	N/A	N/A	N/A
HDL cholesterol[j]	3	107	120	MD (mg/dL)	2.70 (-1.75, 7.15)	1.70 (-13.21, 16.62)	5.0×10^{-1}	0	N/A	N/A	N/A	N/A	N/A
				RR	1.17 (0.90, 1.53)	1.12 (0.54, 2.33)	3.8×10^{-1}	0	N/A	N/A	N/A	N/A	N/A
HDL cholesterol[k]	1	11	11	MD (mg/dL)	8.40 (-7.90, 24.70)	N/A	N/A	N/A	N/A	N/A	N/A	N/A	N/A
				RR	1.48 (0.69, 3.18)	N/A	N/A	N/A	N/A	N/A	N/A	N/A	N/A
LDL cholesterol[j]	3	107	120	MD (mg/dL)	-3.60 (-12.75, 5.55)	-5.24 (-48.31, 37.82)	4.3×10^{-1}	0	N/A	N/A	N/A	N/A	N/A
				RR	0.90 (0.69, 1.17)	0.86 (0.37, 1.98)	3.3×10^{-1}	0	N/A	N/A	N/A	N/A	N/A
LDL cholesterol[k]	1	11	11	MD (mg/dL)	-2.00 (-46.10, 42.10)	N/A	N/A	N/A	N/A	N/A	N/A	N/A	N/A
				RR	0.97 (0.45, 2.06)	N/A	N/A	N/A	N/A	N/A	N/A	N/A	N/A
LDL/HDL ratio	14	711	512	MD (% change)	-11.60 (-15.90, -7.30)	-14.66 (-19.77, -9.55)	2.6×10^{-4}	4.54	-28.38 to -1.68	N/A	100 (57, 100)	0 (N/A, N/A)	N/A
Lipoprotein (a)	10	569	576	MD (% change)	-7.01 (-7.57, -6.45)	-14.62 (-20.66, -8.58)	1.4×10^{-3}	7.92	-33.92 to 0.83	N/A	100 (N/A, N/A)	0 (N/A, N/A)	N/A
Triglyceride[j]	3	106	120	MD (mg/dL)	3.40 (-12.97, 19.77)	2.50 (-7.81, 12.82)	2.4×10^{-1}	0	N/A	N/A	N/A	N/A	N/A
				RR	1.06 (0.81, 1.38)	1.04 (0.83, 1.31)	3.3×10^{-1}	0	N/A	N/A	N/A	N/A	N/A
Triglyceride[k]	1	11	11	MD (mg/dL)	26.00 (-57.29, 109.29)	N/A	N/A	N/A	N/A	N/A	N/A	N/A	N/A
				RR	1.27 (0.59, 2.71)	N/A	N/A	N/A	N/A	N/A	N/A	N/A	N/A
Triglyceride[i]	14	642	445	MD (% change)	7.90 (2.20, 13.60)	3.57 (-3.58, 10.72)	2.4×10^{-1}	0	N/A	N/A	N/A	N/A	N/A
Body mass index[l]	2	4,430	4,503	MD (kg/m^2)	-0.10 (-0.37, 0.17)	-0.19 (-2.92, 2.54)	5.4×10^{-1}	0.19	N/A	N/A	N/A	N/A	N/A
				RR	0.99 (0.95, 1.02)	0.97 (0.55, 1.70)	5.8×10^{-1}	0.04	N/A	N/A	N/A	N/A	N/A
Body mass index[j]	1	8	16	MD (kg/m^2)	2.00 (-1.98, 5.98)	N/A	N/A	N/A	N/A	N/A	N/A	N/A	N/A
				RR	1.57 (0.72, 3.42)	N/A	N/A	N/A	N/A	N/A	N/A	N/A	N/A
Body weight	9	5,282	4,912	MD (kg)	-0.20 (-0.95, 0.55)	0.19 (-1.35, 1.74)	7.3×10^{-1}	0.74	N/A	N/A	N/A	N/A	N/A
				RR	0.99 (0.95, 1.03)	1.02 (0.88, 1.18)	6.8×10^{-1}	0.05	N/A	N/A	N/A	N/A	N/A
Abdominal fat	1	14	14	MD (% change)	-5.70 (-52.00, 40.60)	N/A	N/A	N/A	N/A	N/A	N/A	N/A	N/A
Waist circumference	1	16	14	MD (% change)	-0.60 (-10.60, 9.40)	N/A	N/A	N/A	N/A	N/A	N/A	N/A	N/A

Waist/hip ratio	1	170	166	MD	0.00 (-0.02, 0.02)	N/A	N/A	N/A	N/A	N/A	N/A	N/A	N/A
				RR	1.00 (0.82, 1.21)	N/A	N/A	N/A	N/A	N/A	N/A	N/A	N/A
**Mental or behavioural disorders**													
Dementia (probable)	1	28/1,464	19/1,483	RR	1.49 (0.84, 2.66)	N/A	N/A	N/A	N/A	N/A	N/A	N/A	N/A
**Mental or behavioural symptoms, signs or clinical findings**													
Depressive symptom	3	337	327	SMD	0.10 (-0.09, 0.29)	-0.32 (-1.35, 0.71)	3.1×10^{-1}	0.43	N/A	N/A	N/A	N/A	N/A
				RR	1.09 (0.92, 1.30)	0.75 (0.29, 1.90)	3.1×10^{-1}	0.39	N/A	N/A	N/A	N/A	N/A
**Diseases of the nervous system**													
Cerebrovascular disease	4	190/6,034	143/6,149	RR	1.37 (1.08, 1.73)	1.35 (0.94, 1.92)	6.1×10^{-2}	0	N/A	N/A	N/A	N/A	N/A
Stroke	3	289/5,934	259/6,044	RR	1.12 (0.95, 1.33)	1.14 (0.46, 2.79)	3.2×10^{-1}	0	N/A	N/A	N/A	N/A	N/A
Fatal stroke	1	126/5,310	132/5,429	RR	0.98 (0.77, 1.24)	N/A	N/A	N/A	N/A	N/A	N/A	N/A	N/A
Non-fatal stroke	2	115/5,421	85/5,540	RR	1.37 (1.04, 1.81)	1.38 (0.58, 3.26)	1.3×10^{-1}	0	N/A	N/A	N/A	N/A	N/A
Transient ischaemic attack	2	16/624	13/615	RR	1.13 (0.54, 2.36)	1.19 (0.08, 17.66)	5.6×10^{-1}	0	N/A	N/A	N/A	N/A	N/A
Alzheimer disease	1	13/1,464	9/1,483	RR	1.46 (0.63, 3.41)	N/A	N/A	N/A	N/A	N/A	N/A	N/A	N/A
**Diseases of the circulatory system**													
Coronary heart disease													
Incidence	4	378/5,933	409/6,041	RR	0.94 (0.82, 1.08)	0.94 (0.85, 1.04)	8.6×10^{-2}	0	N/A	N/A	N/A	N/A	N/A
Mortality	3	245/6,039	282/6,158	RR	0.89 (0.75, 1.05)	0.89 (0.68, 1.16)	1.1×10^{-1}	0	N/A	N/A	N/A	N/A	N/A
MI	3	299/5,758	304/5,867	RR	1.01 (0.86, 1.19)	1.00 (0.65, 1.55)	9.4×10^{-1}	0	N/A	N/A	N/A	N/A	N/A
Fatal MI	2	1/448	6/438	RR	0.19 (0.02, 1.65)	0.23 (0.01, 5.53)	1.1×10^{-1}	0	N/A	N/A	N/A	N/A	N/A
Non-fatal MI	3	162/5,758	178/5,867	RR	0.91 (0.73, 1.13)	0.93 (0.34, 2.53)	5.3×10^{-1}	0	N/A	N/A	N/A	N/A	N/A
Any angina pectoris	1	163/5,310	171/5,429	RR	0.97 (0.79, 1.20)	N/A	N/A	N/A	N/A	N/A	N/A	N/A	N/A
Venous thromboembolism	7	183/6,819	169/6,681	RR	1.04 (0.84, 1.29)	1.07 (0.41, 2.79)	6.0×10^{-1}	0	N/A	N/A	N/A	N/A	N/A
Deep vein thrombosis	4	142/6,241	136/6,299	RR	1.04 (0.82, 1.31)	1.05 (0.57, 1.92)	5.4×10^{-1}	0	N/A	N/A	N/A	N/A	N/A
Pulmonary embolism	4	113/6,463	101/6,363	RR	1.14 (0.87, 1.50)	1.13 (0.79, 1.61)	1.5×10^{-1}	0	N/A	N/A	N/A	N/A	N/A
**Symptoms, signs or clinical findings of the circulatory system**													
Cardiac death	3	65/5,758	73/5,867	RR	0.94 (0.65, 1.35)	0.90 (0.41, 1.98)	3.7×10^{-1}	0	N/A	N/A	N/A	N/A	N/A
Coronary revascularization	2	406/5,421	398/5,540	RR	1.05 (0.92, 1.19)	1.04 (0.62, 1.76)	4.9×10^{-1}	0	N/A	N/A	N/A	N/A	N/A
Systolic blood pressure	1	111	111	MD (mmHg)	-0.63 (-3.55, 2.29)	N/A	N/A	N/A	N/A	N/A	N/A	N/A	N/A
				RR	0.95 (0.75, 1.21)	N/A	N/A	N/A	N/A	N/A	N/A	N/A	N/A
Diastolic blood pressure	1	111	111	MD (mmHg)	0.48 (-2.04, 3.00)	N/A	N/A	N/A	N/A	N/A	N/A	N/A	N/A
				RR	1.05 (0.82, 1.33)	N/A	N/A	N/A	N/A	N/A	N/A	N/A	N/A
**Diseases of the digestive system**													
Gallbladder disease[m]	3	200/4,416	117/4,514	RR	1.78 (1.42, 2.24)	1.75 (0.40, 7.57)	1.3×10^{-1}	0	N/A	N/A	N/A	N/A	N/A
**Diseases of the genitourinary system**													
Endometrial hyperplasia	13	371/2,742	9/1,142	RR	37.00 (9.30, 147.27)	6.93 (2.07, 23.23)	7.3×10^{-3}	0.79	1.18 to 50.68	0 (N/A, N/A)	0 (N/A, N/A)	100 (N/A, N/A)	100 (N/A, N/A)
Irregular vaginal bleeding	3	113/311	24/99	RR	1.73 (1.10, 2.71)	1.34 (0.07, 24.94)	5.1×10^{-1}	0.32	N/A	N/A	N/A	N/A	N/A
**Injury, poisoning or certain other consequences of external causes**													
All fracture	9	602/7,109	845/6,706	RR	0.73 (0.65, 0.80)	0.70 (0.45, 1.09)	6.7×10^{-2}	0.08	N/A	N/A	N/A	N/A	N/A
Vertebral fracture	2	45/5,367	75/5,471	RR	0.64 (0.44, 0.94)	0.45 (0.00, 1351.77)	4.3×10^{-1}	0.70	N/A	N/A	N/A	N/A	N/A
Nonvertebral fracture	6	25/839	23/510	RR	0.42 (0.17, 1.04)	0.86 (0.20, 3.67)	7.9×10^{-1}	0.80	N/A	N/A	N/A	N/A	N/A
Hip fracture	1	134/5,310	148/5,429	RR	0.93 (0.74, 1.17)	N/A	N/A	N/A	N/A	N/A	N/A	N/A	N/A
**Functioning assessment**													
Sleep quality	2	26	31	SMD	0.67 (-0.05, 1.39)	0.02 (-8.42, 8.46)	9.8×10^{-1}	0.86	N/A	N/A	N/A	N/A	N/A
				RR	1.87 (0.97, 3.58)	1.02 (0.00, 2697.80)	9.8×10^{-1}	0.80	N/A	N/A	N/A	N/A	N/A
Sexual function	6	646	531	SMD	-0.39 (-0.62, -0.15)	-0.26 (-0.51, -0.02)	3.9×10^{-2}	0.17	N/A	N/A	N/A	N/A	N/A
				RR	0.70 (0.57, 0.87)	0.79 (0.63, 0.99)	4.3×10^{-2}	0.17	N/A	N/A	N/A	N/A	N/A
**Others, not elsewhere classified**													
All-cause mortality	15	1,661/7,805	1,785/7,608	RR	0.94 (0.89, 1.00)	0.95 (0.86, 1.04)	9.2×10^{-2}	0	N/A	N/A	N/A	N/A	N/A
Cardiovascular disease													
Incidence	4	444/5,805	397/5,682	RR	1.13 (0.99, 1.29)	1.13 (0.66, 1.93)	2.1×10^{-1}	0	N/A	N/A	N/A	N/A	N/A
Mortality	3	549/5,502	578/5,579	RR	0.97 (0.87, 1.08)	0.97 (0.95, 0.99)	3.9×10^{-2}	0	N/A	N/A	N/A	N/A	N/A

Abbreviations: ET, estrogen-alone therapy; HDL, high-density lipoprotein; LDL, low-density lipoprotein; MD, mean difference; MI, myocardial infarction; N/A, not available or not applicable; PAI-1, plasminogen activator inhibitor-1; PI, prediction interval; RR, risk ratio; SMD, standardized mean difference.

[a] Primary prevention refers to reducing the risk of occurrence of a disease among individuals who do not have that disease.

[b] Incidence unless otherwise indicated.
[c] Point estimate with 95% confidence interval of the study with the smallest standard error in each meta-analysis.
[d] Point estimate with 95% confidence interval of robust random-effects meta-analysis.
[e] *P*-value of robust random-effects meta-analysis.
[f] The estimated standard deviation of true effects (for MD and SMD, T is on its original scale; for RR, T is on log RR scale).
[g] The middle 95% area of the estimated effect distribution. 95% PI is reported only in meta-analyses of ≥ 10 studies. More information about how to interpret 95% PI can be found in S3 Text (section 3).
[h] The proportion with 95% confidence interval of true effects (θ) below or above a threshold of scientific importance; complementary to 95% PI, these metrics estimate the area of the lower and upper tails of the effect distribution. For MD, null = 0; for SMD, q = -0.2, null = 0, q^* = 0.2; for RR, q = 0.9, null = 1.0, q^* = 1.1. These metrics are reported only in meta-analyses of ≥ 10 studies. More information about how to interpret them can be found in S3 Text (section 3).
[i] In postmenopausal women without diabetes.
[j] In healthy postmenopausal women who used low-dose menopausal hormone therapy.
[k] In postmenopausal women with chronic kidney disease.
[l] In postmenopausal women who used menopausal hormone therapy for at least 3 months.
[m] Gallbladder disease requiring surgery.

Table H. Estrogen Plus Progestin Therapy for Primary Prevention of Multiple Outcomes in Included Systematic Reviews and Meta-Analyses of Randomized Controlled Trials[a]

Outcome[b]	No. of Trials	No. of Events/Total No.		Metric (unit)	Most Precise Study[c]	Summary Effect[d]	*P*-Value[e]	T[f]	95% PI[g]	Proportion of True Effects (θ) Below/Above a Threshold (%)[h]			
		EPT	Control							$\hat{P}(\theta < q)$	$\hat{P}(\theta < \text{null})$	$\hat{P}(\theta > \text{null})$	$\hat{P}(\theta > q^*)$
**Neoplasms**													
All cancer													
Incidence	6	1,480/12,660	1,354/12,224	RR	1.04 (0.97, 1.12)	1.05 (0.97, 1.13)	8.9×10^{-2}	0	N/A	N/A	N/A	N/A	N/A
Mortality	4	761/12,486	685/12,047	RR	1.05 (0.95, 1.17)	1.06 (0.78, 1.44)	2.5×10^{-1}	0	N/A	N/A	N/A	N/A	N/A
Colorectal cancer													
Incidence	5	162/12,692	196/11,932	RR	0.80 (0.63, 1.01)	0.79 (0.64, 0.98)	4.5×10^{-2}	0	N/A	N/A	N/A	N/A	N/A
Mortality	1	53/8,506	50/8,102	RR	1.01 (0.69, 1.48)	N/A	N/A	N/A	N/A	N/A	N/A	N/A	N/A
Lung cancer													
Incidence	3	257/9,956	211/9,557	RR	1.13 (0.93, 1.38)	1.17 (0.48, 2.82)	2.7×10^{-1}	0	N/A	N/A	N/A	N/A	N/A
Mortality	3	174/9,956	151/9,557	RR	1.10 (0.88, 1.39)	1.10 (1.00, 1.21)	4.7×10^{-2}	0	N/A	N/A	N/A	N/A	N/A
Breast cancer													
Incidence	7	651/13,548	502/12,580	RR	1.24 (1.10, 1.40)	1.24 (0.97, 1.57)	5.7×10^{-2}	0	N/A	N/A	N/A	N/A	N/A
Mortality	2	74/9,886	53/9,485	RR	1.28 (0.90, 1.82)	1.55 (0.00, 1446.26)	5.7×10^{-1}	0.54	N/A	N/A	N/A	N/A	N/A
Endometrial cancer	6	76/11,159	106/10,026	RR	0.67 (0.50, 0.92)	0.65 (0.19, 2.26)	1.6×10^{-1}	0	N/A	N/A	N/A	N/A	N/A
Ovarian cancer	1	53/8,506	41/8,102	RR	1.23 (0.82, 1.85)	N/A	N/A	N/A	N/A	N/A	N/A	N/A	N/A
**Symptoms, signs or clinical findings of blood, blood-forming organs, or the immune system**													
C-reactive protein[i]	5	450	253	MD (% change)	27.10 (6.20, 48.00)	27.96 (-8.78, 64.70)	8.9×10^{-2}	16.50	N/A	N/A	N/A	N/A	N/A
C-reactive protein[j]	3	134	141	MD (mg/L)	-0.18 (-1.66, 1.30)	-0.08 (-0.88, 0.72)	5.3×10^{-1}	0	N/A	N/A	N/A	N/A	N/A
				RR	0.96 (0.71, 1.30)	0.98 (0.87, 1.12)	5.0×10^{-1}	0	N/A	N/A	N/A	N/A	N/A
PAI-1 antigen	7	329	217	MD (% change)	-67.20 (-95.40, -39.00)	-32.43 (-65.21, 0.35)	5.2×10^{-2}	17.75	N/A	N/A	N/A	N/A	N/A
Fibrinogen	12	671	469	MD (% change)	-7.60 (-11.90, -3.30)	-7.11 (-9.79, -4.43)	2.0×10^{-3}	0	N/A	N/A	N/A	N/A	N/A
E-selectin	4	203	145	MD (% change)	-19.00 (-25.30, -12.70)	-18.74 (-28.10, -9.37)	2.2×10^{-2}	0	N/A	N/A	N/A	N/A	N/A
**Symptoms, signs or clinical findings of endocrine, nutritional or metabolic diseases**													
Insulin resistance	5	643	566	MD (% change)	-4.80 (-19.30, 9.70)	-10.70 (-32.99, 11.58)	1.8×10^{-1}	0	N/A	N/A	N/A	N/A	N/A
Total cholesterol[j]	8	493	446	MD (mg/dL)	-18.53 (-27.17, -9.89)	-15.31 (-20.93, -9.68)	4.5×10^{-4}	3.03	N/A	N/A	N/A	N/A	N/A
				RR	0.58 (0.44, 0.77)	0.63 (0.53, 0.75)	6.2×10^{-4}	0.11	N/A	N/A	N/A	N/A	N/A
Total cholesterol[k]	1	33	32	MD (mg/dL)	9.00 (-12.39, 30.39)	N/A	N/A	N/A	N/A	N/A	N/A	N/A	N/A
				RR	1.20 (0.77, 1.87)	N/A	N/A	N/A	N/A	N/A	N/A	N/A	N/A
Total cholesterol[l]	5	125	132	MD (mmol/L)	-0.46 (-0.84, -0.08)	-0.39 (-0.78, -0.01)	4.8×10^{-2}	0.09	N/A	N/A	N/A	N/A	N/A
HDL cholesterol[j]	9	524	500	MD (mg/dL)	1.54 (-2.62, 5.70)	1.66 (-4.89, 8.21)	5.7×10^{-1}	8.15	N/A	N/A	N/A	N/A	N/A
				RR	1.10 (0.84, 1.45)	1.13 (0.70, 1.82)	5.6×10^{-1}	0.45	N/A	N/A	N/A	N/A	N/A
HDL cholesterol[k]	1	33	32	MD (mg/dL)	12.30 (5.22, 19.38)	N/A	N/A	N/A	N/A	N/A	N/A	N/A	N/A
				RR	2.15 (1.36, 3.40)	N/A	N/A	N/A	N/A	N/A	N/A	N/A	N/A
HDL cholesterol[l]	5	125	132	MD (mmol/L)	0.01 (-0.14, 0.16)	-0.05 (-0.17, 0.07)	3.3×10^{-1}	0.01	N/A	N/A	N/A	N/A	N/A
LDL cholesterol[j]	8	493	446	MD (mg/dL)	-14.29 (-23.77, -4.81)	-14.96 (-19.32, -10.59)	1.4×10^{-4}	0	N/A	N/A	N/A	N/A	N/A
				RR	0.66 (0.50, 0.87)	0.67 (0.56, 0.80)	1.3×10^{-3}	0.15	N/A	N/A	N/A	N/A	N/A
LDL cholesterol[k]	1	33	32	MD (mg/dL)	-8.00 (-26.48, 10.48)	N/A	N/A	N/A	N/A	N/A	N/A	N/A	N/A
				RR	0.83 (0.53, 1.29)	N/A	N/A	N/A	N/A	N/A	N/A	N/A	N/A
LDL cholesterol[l]	5	124	128	MD (mmol/L)	-0.18 (-0.53, 0.17)	-0.22 (-0.42, -0.01)	4.2×10^{-2}	0	N/A	N/A	N/A	N/A	N/A
LDL/HDL ratio	30	3,490	3,103	MD (% change)	-20.00 (-22.70, -17.30)	-14.85 (-18.15, -11.54)	2.0×10^{-8}	5.15	-28.94 to -5.00	N/A	100 (N/A, N/A)	0 (N/A, N/A)	N/A
Lipoprotein (a)	27	1,342	1,228	MD (% change)	-2.71 (-3.06, -2.36)	-20.63 (-29.75, -11.51)	2.1×10^{-4}	14.78	-60.05 to 4.27	N/A	93 (74, 96)	7 (0, 22)	N/A
Triglyceride[j]	7	445	394	MD (mg/dL)	-24.80 (-41.43, -8.17)	-6.29 (-26.23, 13.66)	4.7×10^{-1}	20.19	N/A	N/A	N/A	N/A	N/A
				RR	0.64 (0.49, 0.85)	0.87 (0.58, 1.30)	4.3×10^{-1}	0.38	N/A	N/A	N/A	N/A	N/A
Triglyceride[k]	1	33	32	MD (mg/dL)	20.00 (-19.01, 59.01)	N/A	N/A	N/A	N/A	N/A	N/A	N/A	N/A
				RR	1.25 (0.80, 1.95)	N/A	N/A	N/A	N/A	N/A	N/A	N/A	N/A
Triglyceride[l]	4	106	110	MD (mmol/L)	-0.23 (-0.62, 0.16)	-0.11 (-0.65, 0.43)	4.0×10^{-1}	0	N/A	N/A	N/A	N/A	N/A
Triglyceride[i]	29	3,429	3,087	MD (% change)	6.90 (2.50, 11.30)	-0.99 (-6.25, 4.27)	6.9×10^{-1}	6.36	-19.85 to 7.53	N/A	62 (0, 100)	38 (0, 100)	N/A
Body mass index[m]	10	8,432	8,860	MD (kg/m^2)	0.00 (-0.20, 0.20)	-0.10 (-1.10, 0.90)	5.9×10^{-1}	0	N/A	N/A	N/A	N/A	N/A
				RR	1.00 (0.97, 1.03)	0.99 (0.80, 1.22)	6.8×10^{-1}	0	N/A	N/A	N/A	N/A	N/A
Body mass index[j]	1	76	61	MD (kg/m^2)	0.00 (-1.23, 1.23)	N/A	N/A	N/A	N/A	N/A	N/A	N/A	N/A

				RR	1.00 (0.74, 1.36)	1.00 (0.74, 1.36)	1.0×10^{-0}	0	N/A	N/A	N/A	N/A	N/A
Body mass index[l]	2	73	74	MD (kg/m^2)	-0.79 (-2.81, 1.23)	-1.05 (-6.67, 4.57)	2.5×10^{-1}	0	N/A	N/A	N/A	N/A	N/A
Body weight	13	9,204	9,161	MD (kg)	0.30 (-0.27, 0.87)	-0.23 (-1.35, 0.89)	6.2×10^{-1}	0.78	-2.50 to 1.50	N/A	69 (0, 100)	31 (0, 100)	N/A
				RR	1.02 (0.99, 1.05)	0.98 (0.90, 1.07)	6.1×10^{-1}	0.05	0.85 to 1.12	8 (0, 92)	69 (0, 100)	31 (0, 100)	0 (N/A, N/A)
Abdominal fat	3	122	71	MD (% change)	-5.80 (-11.50, -0.10)	-7.45 (-27.64, 12.75)	1.9×10^{-1}	3.16	N/A	N/A	N/A	N/A	N/A
Waist circumference	2	3,067	2,870	MD (% change)	-0.70 (-1.10, -0.30)	-0.70 (-0.74, -0.66)	2.8×10^{-3}	0	N/A	N/A	N/A	N/A	N/A
Waist/hip ratio	1	39	29	MD	0.00 (-0.03, 0.03)	N/A	N/A	N/A	N/A	N/A	N/A	N/A	N/A
				RR	1.00 (0.65, 1.55)	N/A	N/A	N/A	N/A	N/A	N/A	N/A	N/A
BMD of lumbar spine[n]	2	13	23	MD (g/cm^2/year)	2.10 (-0.73, 4.93)	1.26 (-11.48, 14.00)	4.3×10^{-1}	0	N/A	N/A	N/A	N/A	N/A
				RR	1.02 (0.46, 2.23)	1.42 (0.01, 231.76)	5.4×10^{-1}	0.33	N/A	N/A	N/A	N/A	N/A
BMD of proximal femur[n]	2	13	23	MD (g/cm^2/year)	2.30 (0.67, 3.93)	2.24 (0.60, 3.89)	3.7×10^{-2}	0	N/A	N/A	N/A	N/A	N/A
				RR	1.45 (0.66, 3.22)	2.16 (0.01, 845.14)	3.5×10^{-1}	0.46	N/A	N/A	N/A	N/A	N/A
**Mental or behavioural disorders**													
Dementia (probable)	1	40/2,229	21/2,303	RR	1.97 (1.16, 3.33)	N/A	N/A	N/A	N/A	N/A	N/A	N/A	N/A
**Mental or behavioural symptoms, signs or clinical findings**													
Depressive symptom	1	68	37	SMD	0.19 (-0.28, 0.66)	N/A	N/A	N/A	N/A	N/A	N/A	N/A	N/A
				RR	1.19 (0.78, 1.82)	N/A	N/A	N/A	N/A	N/A	N/A	N/A	N/A
**Diseases of the nervous system**													
Cerebrovascular disease	13	337/13,840	277/13,315	RR	1.06 (0.82, 1.35)	1.18 (0.83, 1.66)	2.1×10^{-1}	0	N/A	N/A	N/A	N/A	N/A
Stroke	11	477/12,026	389/11,175	RR	1.15 (0.99, 1.33)	1.17 (0.86, 1.60)	1.1×10^{-1}	0	N/A	N/A	N/A	N/A	N/A
Fatal stroke	5	207/12,323	171/11,839	RR	1.11 (0.90, 1.37)	1.15 (0.31, 4.21)	4.3×10^{-1}	0	N/A	N/A	N/A	N/A	N/A
Non-fatal stroke	7	173/10,367	125/9,964	RR	1.52 (1.10, 2.10)	1.34 (0.49, 3.65)	2.1×10^{-1}	0	N/A	N/A	N/A	N/A	N/A
Transient ischaemic attack	3	37/1,624	45/1,629	RR	0.80 (0.51, 1.23)	0.82 (0.16, 4.22)	3.9×10^{-1}	0	N/A	N/A	N/A	N/A	N/A
Alzheimer disease	1	20/2,229	12/2,303	RR	1.72 (0.84, 3.51)	N/A	N/A	N/A	N/A	N/A	N/A	N/A	N/A
**Diseases of the circulatory system**													
Coronary heart disease													
Incidence	10	671/13,073	596/12,064	RR	1.08 (0.95, 1.22)	1.07 (0.92, 1.25)	1.2×10^{-1}	0	N/A	N/A	N/A	N/A	N/A
Mortality	2	310/8,576	286/8,174	RR	1.04 (0.88, 1.21)	1.03 (0.52, 2.07)	6.6×10^{-1}	0	N/A	N/A	N/A	N/A	N/A
MI	8	399/12,095	333/11,615	RR	1.14 (0.99, 1.32)	1.14 (0.70, 1.83)	1.9×10^{-1}	0	N/A	N/A	N/A	N/A	N/A
Fatal MI	3	1/338	2/334	RR	0.32 (0.01, 7.50)	0.68 (0.03, 15.56)	6.5×10^{-1}	0	N/A	N/A	N/A	N/A	N/A
Non-fatal MI	3	156/10,933	115/10,518	RR	1.26 (0.99, 1.61)	1.28 (0.40, 4.07)	2.3×10^{-1}	0	N/A	N/A	N/A	N/A	N/A
Angina pectoris													
Any angina	1	106/8,506	126/8,102	RR	0.80 (0.62, 1.04)	N/A	N/A	N/A	N/A	N/A	N/A	N/A	N/A
Unstable angina	1	3/2,196	0/2,189	RR	6.98 (0.36, 135.01)	N/A	N/A	N/A	N/A	N/A	N/A	N/A	N/A
Venous thromboembolism	11	278/13,627	143/12,692	RR	1.63 (1.31, 2.03)	2.01 (0.60, 6.73)	1.2×10^{-1}	0.18	1.51 to 3.37	0 (N/A, N/A)	0 (N/A, N/A)	100 (N/A, N/A)	100 (N/A, N/A)
Deep vein thrombosis	5	252/12,181	171/11,776	RR	1.25 (1.02, 1.53)	2.51 (0.42, 14.81)	1.7×10^{-1}	0.65	N/A	N/A	N/A	N/A	N/A
Pulmonary embolism	3	193/12,082	134/11,674	RR	1.28 (1.02, 1.61)	2.02 (0.22, 18.91)	2.7×10^{-1}	0.54	N/A	N/A	N/A	N/A	N/A
**Symptoms, signs or clinical findings of the circulatory system**													
Cardiac death	1	39/8,506	34/8,102	RR	1.09 (0.69, 1.73)	N/A	N/A	N/A	N/A	N/A	N/A	N/A	N/A
Coronary revascularization	1	506/8,506	471/8,102	RR	1.02 (0.91, 1.16)	N/A	N/A	N/A	N/A	N/A	N/A	N/A	N/A
Systolic blood pressure	5	230	195	MD (mmHg)	4.00 (0.26, 7.74)	1.09 (-7.61, 9.79)	7.4×10^{-1}	6.42	N/A	N/A	N/A	N/A	N/A
				RR	1.39 (1.02, 1.89)	1.09 (0.57, 2.11)	7.3×10^{-1}	0.49	N/A	N/A	N/A	N/A	N/A
Diastolic blood pressure	5	230	195	MD (mmHg)	1.90 (-0.45, 4.25)	-0.69 (-4.56, 3.19)	6.2×10^{-1}	1.65	N/A	N/A	N/A	N/A	N/A
				RR	0.83 (0.61, 1.12)	0.92 (0.66, 1.30)	5.4×10^{-1}	0.15	N/A	N/A	N/A	N/A	N/A
**Diseases of the digestive system**													
Gallbladder disease[o]	4	336/9,318	203/8,557	RR	1.64 (1.30, 2.06)	1.56 (0.89, 2.75)	6.4×10^{-2}	0	N/A	N/A	N/A	N/A	N/A
**Diseases of the genitourinary system**													
Endometrial hyperplasia	10	34/3,296	8/947	RR	2.16 (0.49, 9.44)	0.69 (0.22, 2.13)	4.5×10^{-1}	0	N/A	N/A	N/A	N/A	N/A
Irregular vaginal bleeding	6	38/247	15/190	RR	0.50 (0.17, 1.49)	2.73 (0.60, 12.31)	1.4×10^{-1}	0.96	N/A	N/A	N/A	N/A	N/A
**Injury, poisoning or certain other consequences of external causes**													
All fracture	17	1,353/13,634	1,645/13,405	RR	0.82 (0.76, 0.89)	0.69 (0.53, 0.89)	1.1×10^{-2}	0.17	0.49 to 0.96	100 (41, 100)	100 (N/A, N/A)	0 (N/A, N/A)	0 (N/A, N/A)
Vertebral fracture	10	147/10,461	200/10,358	RR	0.78 (0.60, 1.01)	0.68 (0.34, 1.37)	1.4×10^{-1}	0	N/A	N/A	N/A	N/A	N/A
Nonvertebral fracture	17	891/11,593	1,108/11,018	RR	0.79 (0.72, 0.87)	0.76 (0.56, 1.02)	5.8×10^{-2}	0.11	0.57 to 0.91	100 (0, 100)	100 (82, 82)	0 (N/A, N/A)	0 (N/A, N/A)

Hip fracture	4	249/12,152	287/11,746	RR	0.82 (0.69, 0.97)	0.83 (0.45, 1.54)	1.7×10^{-1}	0	N/A	N/A	N/A	N/A	N/A
**Functioning assessment**													
Sleep quality	3	7,660	7,328	SMD	-0.09 (-0.12, -0.06)	-0.12 (-1.39, 1.15)	4.7×10^{-1}	0.09	N/A	N/A	N/A	N/A	N/A
				RR	0.92 (0.89, 0.95)	0.86 (0.21, 3.50)	4.9×10^{-1}	0.13	N/A	N/A	N/A	N/A	N/A
Sexual function	3	668	646	SMD	-0.12 (-0.23, 0.00)	-0.09 (-0.71, 0.53)	3.8×10^{-1}	0.02	N/A	N/A	N/A	N/A	N/A
				RR	0.90 (0.81, 1.00)	0.92 (0.52, 1.64)	4.0×10^{-1}	0.03	N/A	N/A	N/A	N/A	N/A
Skeletal muscle strength	4	104	110	SMD	-0.48 (-0.87, -0.08)	-0.63 (-1.40, 0.15)	8.2×10^{-2}	0.39	N/A	N/A	N/A	N/A	N/A
				RR	0.65 (0.46, 0.93)	0.57 (0.28, 1.15)	8.2×10^{-2}	0.36	N/A	N/A	N/A	N/A	N/A
**Others, not elsewhere classified**													
All-cause mortality	16	2,536/14,949	2,380/13,891	RR	1.01 (0.96, 1.07)	1.02 (0.82, 1.26)	4.9×10^{-1}	0	N/A	N/A	N/A	N/A	N/A
Cardiovascular disease													
Incidence	12	735/12,419	516/11,862	RR	1.54 (1.33, 1.78)	1.38 (0.87, 2.21)	1.1×10^{-1}	0.21	0.89 to 2.04	0 (N/A, N/A)	0 (N/A, N/A)	100 (0, 100)	83 (0, 100)
Mortality	3	692/10,917	644/10,397	RR	1.02 (0.92, 1.13)	1.02 (0.49, 2.10)	7.8×10^{-1}	0	N/A	N/A	N/A	N/A	N/A

Abbreviations: BMD, bone mineral density; EPT, estrogen plus progestin therapy; HDL, high-density lipoprotein; LDL, low-density lipoprotein; MD, mean difference; MI, myocardial infarction; N/A, not available or not applicable; PAI-1, plasminogen activator inhibitor-1; PI, prediction interval; RR, risk ratio; SMD, standardized mean difference.

[a] Primary prevention refers to reducing the risk of occurrence of a disease among individuals who do not have that disease.

[b] Incidence unless otherwise indicated.

[c] Point estimate with 95% confidence interval of the study with the smallest standard error in each meta-analysis.

[d] Point estimate with 95% confidence interval of robust random-effects meta-analysis.

[e] *P*-value of robust random-effects meta-analysis.

[f] The estimated standard deviation of true effects (for MD and SMD, T is on its original scale; for RR, T is on log RR scale).

[g] The middle 95% area of the estimated effect distribution. 95% PI is reported only in meta-analyses of ≥ 10 studies. More information about how to interpret 95% PI can be found in S3 Text (section 3).

[h] The proportion with 95% confidence interval of true effects (θ) below or above a threshold of scientific importance; complementary to 95% PI, these metrics estimate the area of the lower and upper tails of the effect distribution. For MD, null = 0; for SMD, q = -0.2, null = 0, q^* = 0.2; for RR, q = 0.9, null = 1.0, q^* = 1.1. These metrics are reported only in meta-analyses of ≥ 10 studies. More information about how to interpret them can be found in S3 Text (section 3).

[i] In postmenopausal women without diabetes.

[j] In healthy postmenopausal women who used low-dose menopausal hormone therapy.

[k] In postmenopausal women with chronic kidney disease.

[l] In postmenopausal women with diabetes.

[m] In postmenopausal women who used menopausal hormone therapy for at least 3 months.

[n] In postmenopausal women with primary biliary cirrhosis.

[o] Gallbladder disease requiring surgery.

Table I. Any Menopausal Hormone Therapy for Secondary Prevention of Multiple Outcomes in Included Systematic Reviews and Meta-Analyses of Randomized Controlled Trials[a]

Outcome[b]	No. of Trials	No. of Events/Total No.		Metric (unit)	Most Precise Study[c]	Summary Effect[d]	*P*-Value[e]	T[f]	95% PI[g]	Proportion of True Effects (θ) Below/Above a Threshold (%)[h]			
		Any MHT	Control							$\widehat{P}$ ($\theta < q$)	$\widehat{P}$ ($\theta <$ null)	$\widehat{P}$ ($\theta >$ null)	$\widehat{P}$ ($\theta > q^*$)
**Neoplasms**													
Breast cancer recurrence	4	103/494	70/503	RR	1.26 (0.92, 1.74)	1.51 (0.32, 7.12)	2.8×10^{-1}	0.26	N/A	N/A	N/A	N/A	N/A
Ovarian cancer OS	1	32/59	41/66	RR	0.87 (0.65, 1.18)	N/A	N/A	N/A	N/A	N/A	N/A	N/A	N/A
**Symptoms, signs or clinical findings of endocrine, nutritional or metabolic diseases**													
Fasting glucose	5	465	431	MD (mmol/L)	-0.36 (-0.85, 0.13)	-0.63 (-2.22, 0.97)	2.1×10^{-1}	0.17	N/A	N/A	N/A	N/A	N/A
Hemoglobin A1c	4	84	78	MD (%)	-0.51 (-1.01, -0.01)	-0.47 (-0.72, -0.23)	1.4×10^{-2}	0	N/A	N/A	N/A	N/A	N/A
**Mental or behavioural symptoms, signs or clinical findings**													
Depressive symptom	5	139	133	SMD	-0.19 (-0.64, 0.26)	0.16 (-1.62, 1.94)	8.1×10^{-1}	1.08	N/A	N/A	N/A	N/A	N/A
				RR	0.84 (0.56, 1.27)	1.16 (0.23, 5.82)	8.1×10^{-1}	0.98	N/A	N/A	N/A	N/A	N/A
**Diseases of the nervous system**													
Cerebrovascular disease	2	89/371	76/366	RR	1.20 (0.91, 1.57)	0.63 (0.00, ∞)	7.3×10^{-1}	1.19	N/A	N/A	N/A	N/A	N/A
Stroke	2	63/371	60/366	RR	1.09 (0.79, 1.51)	0.61 (0.00, ∞)	7.0×10^{-1}	1.10	N/A	N/A	N/A	N/A	N/A
Fatal stroke	2	12/371	8/366	RR	2.91 (0.95, 8.93)	0.82 (0.00, ∞)	9.2×10^{-1}	1.91	N/A	N/A	N/A	N/A	N/A
Non-fatal stroke	1	51/337	52/327	RR	0.95 (0.67, 1.36)	N/A	N/A	N/A	N/A	N/A	N/A	N/A	N/A
Transient ischaemic attack	1	30/337	25/327	RR	1.16 (0.70, 1.94)	N/A	N/A	N/A	N/A	N/A	N/A	N/A	N/A
**Diseases of the circulatory system**													
Coronary heart disease													
Incidence	8	459/2,590	443/2,455	RR	0.94 (0.81, 1.09)	0.97 (0.76, 1.24)	6.5×10^{-1}	0	N/A	N/A	N/A	N/A	N/A
Mortality	4	161/2,200	156/2,099	RR	1.08 (0.86, 1.37)	1.02 (0.26, 4.05)	9.3×10^{-1}	0	N/A	N/A	N/A	N/A	N/A
MI	9	207/2,630	215/2,498	RR	0.93 (0.75, 1.17)	0.93 (0.76, 1.15)	2.2×10^{-1}	0	N/A	N/A	N/A	N/A	N/A
Fatal MI	5	28/2,197	32/2,070	RR	1.19 (0.61, 2.30)	0.84 (0.08, 9.49)	7.2×10^{-1}	0	N/A	N/A	N/A	N/A	N/A
Non-fatal MI	5	174/2,441	175/2,326	RR	0.90 (0.71, 1.14)	0.96 (0.43, 2.15)	7.7×10^{-1}	0	N/A	N/A	N/A	N/A	N/A
Angina pectoris													
Any angina	3	154/1,624	152/1,531	RR	0.91 (0.71, 1.17)	0.91 (0.64, 1.29)	2.4×10^{-1}	0	N/A	N/A	N/A	N/A	N/A
Unstable angina	4	184/1,758	171/1,629	RR	0.88 (0.68, 1.14)	0.97 (0.51, 1.83)	8.5×10^{-1}	0.13	N/A	N/A	N/A	N/A	N/A
Venous thromboembolism	1	8/71	1/69	RR	7.77 (1.00, 60.53)	N/A	N/A	N/A	N/A	N/A	N/A	N/A	N/A
Deep vein thrombosis	1	4/71	0/69	RR	8.75 (0.48, 159.53)	N/A	N/A	N/A	N/A	N/A	N/A	N/A	N/A
Pulmonary embolism	1	3/71	1/69	RR	2.92 (0.31, 27.35)	N/A	N/A	N/A	N/A	N/A	N/A	N/A	N/A
**Symptoms, signs or clinical findings of the circulatory system**													
Cardiac death	4	105/2,307	98/2,205	RR	1.19 (0.85, 1.67)	1.04 (0.33, 3.30)	8.7×10^{-1}	0.05	N/A	N/A	N/A	N/A	N/A
Coronary revascularization	3	313/1,624	312/1,531	RR	0.93 (0.80, 1.08)	0.98 (0.29, 3.33)	8.9×10^{-1}	0.29	N/A	N/A	N/A	N/A	N/A
**Diseases of the genitourinary system**													
RUTI	3	54/139	89/134	RR	0.64 (0.47, 0.86)	0.58 (0.11, 2.99)	2.9×10^{-1}	0.51	N/A	N/A	N/A	N/A	N/A
Vaginal atrophy	5	151/999	605/904	RR	0.22 (0.19, 0.26)	0.31 (0.12, 0.81)	3.0×10^{-2}	0.55	N/A	N/A	N/A	N/A	N/A
**Symptoms, signs or clinical findings of the genitourinary system**													
Vasomotor symptom	9	791	313	MD (freq/week)	-11.79 (-19.57, -4.01)	-17.92 (-23.80, -12.04)	1.4×10^{-4}	5.43	N/A	N/A	N/A	N/A	N/A
				RR	0.44 (0.34, 0.57)	0.43 (0.33, 0.57)	1.3×10^{-4}	0.26	N/A	N/A	N/A	N/A	N/A
Vasomotor symptom severity	7	299	204	SMD	-1.10 (-1.53, -0.68)	-1.36 (-1.95, -0.76)	1.5×10^{-3}	0.54	N/A	N/A	N/A	N/A	N/A
				RR	0.36 (0.25, 0.54)	0.29 (0.17, 0.50)	1.5×10^{-3}	0.50	N/A	N/A	N/A	N/A	N/A
Urinary incontinence	13	N/A/8,500	N/A/8,499	RR	1.09 (1.02, 1.17)	0.82 (0.62, 1.09)	1.5×10^{-1}	0.28	0.36 to 1.94	77 (0, 92)	77 (38, 92)	23 (0, 46)	15 (0, 31)
**Functioning assessment**													
Sleep quality	2	33	32	SMD	0.27 (-0.41, 0.96)	0.00 (-3.49, 3.50)	9.9×10^{-1}	0.16	N/A	N/A	N/A	N/A	N/A
				RR	1.29 (0.69, 2.40)	1.00 (0.04, 25.90)	9.9×10^{-1}	0.17	N/A	N/A	N/A	N/A	N/A
**Others, not elsewhere classified**													
Cardiovascular disease													
Incidence	11	791/3,032	701/2,890	RR	1.03 (0.92, 1.15)	1.08 (0.94, 1.25)	1.8×10^{-1}	0.07	0.94 to 1.27	0 (N/A, N/A)	18 (0, 100)	82 (0, 100)	45 (0, 100)
Mortality	9	220/2,811	203/2,669	RR	1.11 (0.89, 1.37)	1.06 (0.62, 1.80)	6.0×10^{-1}	0	N/A	N/A	N/A	N/A	N/A

Abbreviations: freq, frequency; MD, mean difference; MHT, menopausal hormone therapy; MI, myocardial infarction; N/A, not available or not applicable; OS, overall survival; PI, prediction interval; RR, risk ratio; RUTI, recurrent urinary tract infection; SMD, standardized mean difference.

[a] Secondary prevention refers to reducing the risk of recurrence of a disease among individuals who already have that disease.

[b] Incidence unless otherwise indicated.

[c] Point estimate with 95% confidence interval of the study with the smallest standard error in each meta-analysis.

[d] Point estimate with 95% confidence interval of robust random-effects meta-analysis.
[e] *P*-value of robust random-effects meta-analysis.
[f] The estimated standard deviation of true effects (for MD and SMD, T is on its original scale; for RR, T is on log RR scale).
[g] The middle 95% area of the estimated effect distribution. 95% PI is reported only in meta-analyses of ≥ 10 studies. More information about how to interpret 95% PI can be found in S3 Text (section 3).
[h] The proportion with 95% confidence interval of true effects (θ) below or above a threshold of scientific importance; complementary to 95% PI, these metrics estimate the area of the lower and upper tails of the effect distribution. For MD, null = 0; for SMD, q = -0.2, null = 0, q^* = 0.2; for RR, q = 0.9, null = 1.0, q^* = 1.1. These metrics are reported only in meta-analyses of ≥ 10 studies. More information about how to interpret them can be found in S3 Text (section 3).

Table J. Estrogen-Alone Therapy for Secondary Prevention of Multiple Outcomes in Included Systematic Reviews and Meta-Analyses of Randomized Controlled Trials[a]

Outcome[b]	No. of Trials	No. of Events/Total No.		Metric (unit)	Most Precise Study[c]	Summary Effect[d]	*P*-Value[e]	T[f]	95% PI[g]	Proportion of True Effects (θ) Below/Above a Threshold (%)[h]			
		ET	Control							$\hat{P}\ (\theta < q)$	$\hat{P}\ (\theta <$ null)	$\hat{P}\ (\theta >$ null)	$\hat{P}\ (\theta > q^{*})$
**Neoplasms**													
Breast cancer recurrence	1	2/34	4/43	RR	0.63 (0.12, 3.25)	N/A	N/A	N/A	N/A	N/A	N/A	N/A	N/A
Ovarian cancer OS	1	32/59	41/66	RR	0.87 (0.65, 1.18)	N/A	N/A	N/A	N/A	N/A	N/A	N/A	N/A
**Mental or behavioural symptoms, signs or clinical findings**													
Depressive symptom	4	100	94	SMD	0.38 (-0.15, 0.91)	0.27 (-2.42, 2.95)	7.7×10^{-1}	1.36	N/A	N/A	N/A	N/A	N/A
				RR	1.41 (0.87, 2.28)	1.27 (0.11, 14.49)	7.7×10^{-1}	1.23	N/A	N/A	N/A	N/A	N/A
**Diseases of the nervous system**													
Cerebrovascular disease	2	89/371	76/366	RR	1.20 (0.91, 1.57)	0.63 (0.00, ∞)	7.3×10^{-1}	1.19	N/A	N/A	N/A	N/A	N/A
Stroke	2	63/371	60/366	RR	1.09 (0.79, 1.51)	0.61 (0.00, ∞)	7.0×10^{-1}	1.10	N/A	N/A	N/A	N/A	N/A
Fatal stroke	2	12/371	8/366	RR	2.91 (0.95, 8.93)	0.82 (0.00, ∞)	9.2×10^{-1}	1.91	N/A	N/A	N/A	N/A	N/A
Non-fatal stroke	1	51/337	52/327	RR	0.95 (0.67, 1.36)	N/A	N/A	N/A	N/A	N/A	N/A	N/A	N/A
**Diseases of the circulatory system**													
Coronary heart disease													
Incidence	2	87/613	91/609	RR	1.00 (0.72, 1.39)	0.95 (0.43, 2.12)	5.9×10^{-1}	0	N/A	N/A	N/A	N/A	N/A
Mortality	3	26/673	34/671	RR	0.69 (0.40, 1.18)	0.76 (0.08, 7.17)	4.2×10^{-1}	0	N/A	N/A	N/A	N/A	N/A
MI	2	54/613	52/609	RR	1.05 (0.71, 1.55)	1.03 (0.57, 1.85)	6.3×10^{-1}	0	N/A	N/A	N/A	N/A	N/A
Fatal MI	2	7/613	14/609	RR	0.45 (0.17, 1.18)	0.50 (0.02, 13.61)	2.3×10^{-1}	0	N/A	N/A	N/A	N/A	N/A
Non-fatal MI	2	47/613	38/609	RR	1.30 (0.83, 2.04)	1.23 (0.23, 6.61)	3.6×10^{-1}	0	N/A	N/A	N/A	N/A	N/A
Angina pectoris													
Any angina	1	18/100	22/105	RR	0.86 (0.49, 1.50)	N/A	N/A	N/A	N/A	N/A	N/A	N/A	N/A
Unstable angina	1	18/100	22/105	RR	0.86 (0.49, 1.50)	N/A	N/A	N/A	N/A	N/A	N/A	N/A	N/A
**Symptoms, signs or clinical findings of the circulatory system**													
Cardiac death	2	25/613	33/609	RR	0.69 (0.40, 1.18)	0.75 (0.04, 14.13)	4.3×10^{-1}	0	N/A	N/A	N/A	N/A	N/A
Coronary revascularization	1	18/100	24/105	RR	0.79 (0.46, 1.36)	N/A	N/A	N/A	N/A	N/A	N/A	N/A	N/A
**Diseases of the genitourinary system**													
RUTI	3	54/139	89/134	RR	0.64 (0.47, 0.86)	0.58 (0.11, 2.99)	2.9×10^{-1}	0.51	N/A	N/A	N/A	N/A	N/A
Vaginal atrophy	5	151/999	605/904	RR	0.22 (0.19, 0.26)	0.31 (0.12, 0.81)	3.0×10^{-2}	0.55	N/A	N/A	N/A	N/A	N/A
**Symptoms, signs or clinical findings of the genitourinary system**													
Vasomotor symptom	3	269	96	MD (freq/week)	-15.00 (-23.28, -6.72)	-14.78 (-22.11, -7.46)	1.6×10^{-2}	0	N/A	N/A	N/A	N/A	N/A
				RR	0.58 (0.44, 0.76)	0.57 (0.40, 0.80)	2.6×10^{-2}	0	N/A	N/A	N/A	N/A	N/A
Vasomotor symptom severity	1	43	40	SMD	-0.55 (-0.99, -0.11)	N/A	N/A	N/A	N/A	N/A	N/A	N/A	N/A
				RR	0.60 (0.41, 0.90)	N/A	N/A	N/A	N/A	N/A	N/A	N/A	N/A
Urinary incontinence	10	N/A/3,173	N/A/3,191	RR	1.59 (1.39, 1.82)	0.68 (0.47, 0.99)	4.4×10^{-2}	0.49	0.23 to 2.34	90 (20, 100)	90 (50, 100)	10 (0, 30)	10 (0, 30)
**Functioning assessment**													
Sleep quality	1	17	16	SMD	0.27 (-0.41, 0.96)	N/A	N/A	N/A	N/A	N/A	N/A	N/A	N/A
				RR	1.29 (0.69, 2.40)	N/A	N/A	N/A	N/A	N/A	N/A	N/A	N/A
**Others, not elsewhere classified**													
Cardiovascular disease													
Incidence	4	229/984	211/975	RR	1.15 (0.90, 1.47)	1.08 (0.89, 1.32)	2.0×10^{-1}	0	N/A	N/A	N/A	N/A	N/A
Mortality	3	48/950	50/936	RR	0.69 (0.40, 1.18)	0.97 (0.15, 6.20)	9.2×10^{-1}	0.22	N/A	N/A	N/A	N/A	N/A

Abbreviations: ET, estrogen-alone therapy; freq, frequency; MD, mean difference; MI, myocardial infarction; N/A, not available or not applicable; OS, overall survival; PI, prediction interval; RR, risk ratio; RUTI, recurrent urinary tract infection; SMD, standardized mean difference.

[a] Secondary prevention refers to reducing the risk of recurrence of a disease among individuals who already have that disease.

[b] Incidence unless otherwise indicated.

[c] Point estimate with 95% confidence interval of the study with the smallest standard error in each meta-analysis.

[d] Point estimate with 95% confidence interval of robust random-effects meta-analysis.

[e] *P*-value of robust random-effects meta-analysis.

[f] The estimated standard deviation of true effects (for MD and SMD, T is on its original scale; for RR, T is on log RR scale).

[g] The middle 95% area of the estimated effect distribution. 95% PI is reported only in meta-analyses of ≥ 10 studies. More information about how to interpret 95% PI can be found in S3 Text (section 3).

[h] The proportion with 95% confidence interval of true effects (θ) below or above a threshold of scientific importance; complementary to 95% PI, these metrics estimate the area of the lower and upper tails of the effect distribution. For MD, null = 0; for SMD, q = -0.2, null = 0, q^{*} = 0.2; for RR, q = 0.9, null = 1.0, q^{*} = 1.1. These metrics are reported only in meta-analyses of ≥ 10 studies. More information about how to interpret them can be found in S3 Text (section 3).

Table K. Estrogen Plus Progestin Therapy for Secondary Prevention of Multiple Outcomes in Included Systematic Reviews and Meta-Analyses of Randomized Controlled Trials[a]

Outcome[b]	No. of Trials	No. of Events/Total No.		Metric (unit)	Most Precise Study[c]	Summary Effect[d]	*P*-Value[e]	T[f]	95% PI[g]	Proportion of True Effects (θ) Below/Above a Threshold (%)[h]			
		EPT	Control							$\hat{P}\ (\theta < q)$	$\hat{P}\ (\theta <$ null$)$	$\hat{P}\ (\theta >$ null$)$	$\hat{P}\ (\theta > q^*)$
**Symptoms, signs or clinical findings of endocrine, nutritional or metabolic diseases**													
Fasting glucose	5	465	431	MD (mmol/L)	-0.36 (-0.85, 0.13)	-0.63 (-2.22, 0.97)	$2.1×10^{-1}$	0.17	N/A	N/A	N/A	N/A	N/A
Hemoglobin A1c	4	84	78	MD (%)	-0.51 (-1.01, -0.01)	-0.47 (-0.72, -0.23)	$1.4×10^{-2}$	0	N/A	N/A	N/A	N/A	N/A
**Mental or behavioural symptoms, signs or clinical findings**													
Depressive symptom	1	39	39	SMD	-0.19 (-0.64, 0.26)	N/A	N/A	N/A	N/A	N/A	N/A	N/A	N/A
				RR	0.84 (0.56, 1.27)	N/A	N/A	N/A	N/A	N/A	N/A	N/A	N/A
**Diseases of the circulatory system**													
Coronary heart disease													
Incidence	4	302/1,573	330/1,559	RR	0.94 (0.81, 1.09)	0.92 (0.38, 2.22)	$4.6×10^{-1}$	0	N/A	N/A	N/A	N/A	N/A
Mortality	3	135/1,527	125/1,533	RR	1.08 (0.86, 1.37)	1.08 (0.85, 1.37)	$1.5×10^{-1}$	0	N/A	N/A	N/A	N/A	N/A
MI	4	145/1,573	160/1,559	RR	0.93 (0.75, 1.17)	0.91 (0.37, 2.24)	$4.6×10^{-1}$	0	N/A	N/A	N/A	N/A	N/A
Fatal MI	3	20/1,524	18/1,508	RR	1.19 (0.61, 2.30)	1.09 (0.07, 17.24)	$7.9×10^{-1}$	0	N/A	N/A	N/A	N/A	N/A
Non-fatal MI	2	122/1,484	136/1,488	RR	0.90 (0.71, 1.14)	0.90 (0.80, 1.01)	$5.2×10^{-2}$	0	N/A	N/A	N/A	N/A	N/A
Angina pectoris													
Any angina	2	124/1,484	142/1,488	RR	0.91 (0.71, 1.17)	0.87 (0.25, 3.07)	$4.0×10^{-1}$	0	N/A	N/A	N/A	N/A	N/A
Unstable angina	3	120/1,524	139/1,508	RR	0.88 (0.68, 1.14)	0.86 (0.34, 2.12)	$2.8×10^{-1}$	0	N/A	N/A	N/A	N/A	N/A
Venous thromboembolism	1	8/71	1/69	RR	7.77 (1.00, 60.53)	N/A	N/A	N/A	N/A	N/A	N/A	N/A	N/A
Deep vein thrombosis	1	4/71	0/69	RR	8.75 (0.48, 159.53)	N/A	N/A	N/A	N/A	N/A	N/A	N/A	N/A
Pulmonary embolism	1	3/71	1/69	RR	2.92 (0.31, 27.35)	N/A	N/A	N/A	N/A	N/A	N/A	N/A	N/A
**Symptoms, signs or clinical findings of the circulatory system**													
Cardiac death	2	72/1,484	62/1,488	RR	1.19 (0.85, 1.67)	1.17 (0.31, 4.42)	$3.8×10^{-1}$	0	N/A	N/A	N/A	N/A	N/A
Coronary revascularization	2	287/1,484	311/1,488	RR	0.93 (0.80, 1.08)	0.93 (0.66, 1.30)	$2.1×10^{-1}$	0	N/A	N/A	N/A	N/A	N/A
**Symptoms, signs or clinical findings of the genitourinary system**													
Vasomotor symptom	6	522	217	MD (freq/week)	-11.79 (-19.57, -4.01)	-19.64 (-29.14, -10.15)	$3.4×10^{-3}$	7.17	N/A	N/A	N/A	N/A	N/A
				RR	0.44 (0.34, 0.57)	0.39 (0.26, 0.57)	$1.6×10^{-3}$	0.27	N/A	N/A	N/A	N/A	N/A
Vasomotor symptom severity	6	256	164	SMD	-1.10 (-1.53, -0.68)	-1.50 (-2.10, -0.90)	$1.5×10^{-3}$	0.44	N/A	N/A	N/A	N/A	N/A
				RR	0.36 (0.25, 0.54)	0.25 (0.14, 0.44)	$1.5×10^{-3}$	0.41	N/A	N/A	N/A	N/A	N/A
Urinary incontinence	3	N/A/5,327	N/A/5,308	RR	1.09 (1.02, 1.17)	1.11 (0.78, 1.59)	$2.0×10^{-1}$	0.05	N/A	N/A	N/A	N/A	N/A
**Functioning assessment**													
Sleep quality	1	16	16	SMD	-0.28 (-0.97, 0.42)	N/A	N/A	N/A	N/A	N/A	N/A	N/A	N/A
				RR	0.77 (0.41, 1.46)	N/A	N/A	N/A	N/A	N/A	N/A	N/A	N/A
**Others, not elsewhere classified**													
Cardiovascular disease													
Incidence	5	472/1,644	464/1,628	RR	1.03 (0.92, 1.15)	0.98 (0.54, 1.78)	$8.5×10^{-1}$	0.19	N/A	N/A	N/A	N/A	N/A
Mortality	3	161/1,524	148/1,508	RR	1.11 (0.89, 1.37)	1.09 (0.28, 4.28)	$5.8×10^{-1}$	0	N/A	N/A	N/A	N/A	N/A

Abbreviations: EPT, estrogen plus progestin therapy; freq, frequency; MD, mean difference; MI, myocardial infarction; N/A, not available or not applicable; PI, prediction interval; RR, risk ratio; SMD, standardized mean difference.

[a] Secondary prevention refers to reducing the risk of recurrence of a disease among individuals who already have that disease.

[b] Incidence unless otherwise indicated.

[c] Point estimate with 95% confidence interval of the study with the smallest standard error in each meta-analysis.

[d] Point estimate with 95% confidence interval of robust random-effects meta-analysis.

[e] *P*-value of robust random-effects meta-analysis.

[f] The estimated standard deviation of true effects (for MD and SMD, T is on its original scale; for RR, T is on log RR scale).

[g] The middle 95% area of the estimated effect distribution. 95% PI is reported only in meta-analyses of ≥ 10 studies. More information about how to interpret 95% PI can be found in S3 Text (section 3).

[h] The proportion with 95% confidence interval of true effects (θ) below or above a threshold of scientific importance; complementary to 95% PI, these metrics estimate the area of the lower and upper tails of the effect distribution. For MD, null = 0; for SMD, q = -0.2, null = 0, q^* = 0.2; for RR, q = 0.9, null = 1.0, q^* = 1.1. These metrics are reported only in meta-analyses of ≥ 10 studies. More information about how to interpret them can be found in S3 Text (section 3).

Table L. Any Menopausal Hormone Therapy for Primary Prevention of Multiple Outcomes in Included Systematic Reviews and Meta-Analyses of Observational Epidemiological Studies[a]

Outcome[b]	Timing of MHT	No. of Studies (Cc/Co)	No. of Cases/Population	Metric	Most Precise Study[c]	Summary Effect[d]	*P*-Value[e]	T[f]	95% PI[g]	Proportion of True Effects (θ) Below/Above a Threshold (%)[h] $\hat{P}$ ($\theta < 0.9$)	$\hat{P}$ ($\theta < 1.0$)	$\hat{P}$ ($\theta > 1.0$)	$\hat{P}$ ($\theta > 1.1$)
**Neoplasms**													
Cutaneous melanoma	Ever	7 (6/1)	1,875/61,114	RR	1.20 (0.80, 1.80)	1.23 (0.90, 1.68)	1.4×10^{-1}	0	N/A	N/A	N/A	N/A	N/A
Glioma	Ever	10 (6/4)	3,002/1,580,830	RR	1.05 (0.88, 1.25)	0.87 (0.72, 1.04)	1.1×10^{-1}	0.16	0.57 to 1.21	80 (0, 100)	80 (0, 100)	20 (0, 70)	0 (N/A, N/A)
	Current	7 (4/3)	2,632/1,541,011	RR	1.09 (0.90, 1.32)	0.82 (0.60, 1.13)	1.9×10^{-1}	0.25	N/A	N/A	N/A	N/A	N/A
	Past	7 (4/3)	2,632/1,541,011	RR	1.02 (0.82, 1.27)	0.94 (0.75, 1.19)	5.5×10^{-1}	0.13	N/A	N/A	N/A	N/A	N/A
Meningioma	Ever	13 (9/4)	4,618/1,479,871	RR	1.30 (1.13, 1.50)	1.14 (0.98, 1.33)	7.6×10^{-2}	0.17	0.61 to 1.59	8 (0, 38)	31 (0, 62)	69 (20, 100)	54 (15, 77)
	Current	10 (7/3)	4,131/1,451,024	RR	1.30 (1.13, 1.50)	1.22 (1.02, 1.46)	3.5×10^{-2}	0.19	0.53 to 1.77	10 (0, 30)	10 (0, 50)	90 (10, 100)	70 (10, 100)
	Past	10 (7/3)	4,131/1,451,024	RR	1.33 (1.10, 1.61)	1.15 (0.98, 1.37)	8.0×10^{-2}	0.01	1.13 to 1.18	0 (N/A, N/A)	0 (N/A, N/A)	100 (0, 100)	100 (0, 100)
Thyroid cancer	Ever	12 (4/8)	1,957/802,681	RR	1.40 (1.15, 1.70)	1.09 (0.88, 1.34)	3.6×10^{-1}	0.14	0.82 to 1.44	17 (0, 100)	25 (0, 100)	75 (0, 100)	50 (0, 100)
	Current	4 (0/4)	1,197/692,663	RR	1.22 (0.95, 1.57)	1.11 (0.76, 1.61)	4.5×10^{-1}	0.17	N/A	N/A	N/A	N/A	N/A
	Past	3 (0/3)	734/402,492	RR	0.88 (0.61, 1.26)	1.01 (0.51, 1.99)	9.6×10^{-1}	0.08	N/A	N/A	N/A	N/A	N/A
Esophageal cancer	Ever	5 (4/1)	1,432/203,548	RR	0.68 (0.53, 0.88)	0.70 (0.60, 0.81)	8.6×10^{-3}	0	N/A	N/A	N/A	N/A	N/A
	Current	3 (1/2)	1,093/132,186	RR	0.68 (0.49, 0.94)	0.68 (0.62, 0.74)	1.0×10^{-2}	0	N/A	N/A	N/A	N/A	N/A
	Past	3 (1/2)	1,093/132,186	RR	0.70 (0.52, 0.95)	0.72 (0.35, 1.50)	1.1×10^{-1}	0	N/A	N/A	N/A	N/A	N/A
Gastric cancer	Ever	6 (3/3)	1,555/616,630	RR	0.75 (0.54, 1.05)	0.78 (0.70, 0.86)	3.3×10^{-3}	0	N/A	N/A	N/A	N/A	N/A
	Current	3 (1/2)	799/130,359	RR	0.69 (0.43, 1.10)	0.75 (0.27, 2.06)	1.7×10^{-1}	0	N/A	N/A	N/A	N/A	N/A
	Past	3 (1/2)	799/130,359	RR	0.82 (0.55, 1.22)	0.84 (0.61, 1.16)	9.2×10^{-2}	0	N/A	N/A	N/A	N/A	N/A
Colorectal cancer	Ever	25 (17/8)	22,690/527,776	RR	0.81 (0.73, 0.90)	0.83 (0.77, 0.89)	2.2×10^{-5}	0.12	0.57 to 1.06	64 (36, 92)	92 (68, 100)	8 (0, 24)	0 (N/A, N/A)
	Current	22 (12/10)	18,166/582,956	RR	0.80 (0.71, 0.90)	0.77 (0.71, 0.83)	3.6×10^{-6}	0.10	0.63 to 0.92	100 (50, 100)	100 (73, 73)	0 (N/A, N/A)	0 (N/A, N/A)
	Past	18 (12/6)	16,011/415,854	RR	0.84 (0.74, 0.96)	0.88 (0.81, 0.97)	1.1×10^{-2}	0.11	0.64 to 1.10	56 (0, 94)	89 (11, 100)	11 (0, 50)	0 (N/A, N/A)
Pancreatic cancer	Ever	11 (6/5)	2,211/505,673	RR	0.70 (0.54, 0.91)	0.96 (0.82, 1.13)	6.0×10^{-1}	0.14	0.70 to 1.31	36 (0, 100)	45 (0, 100)	55 (0, 100)	27 (0, 100)
	Current	3 (0/3)	548/134,847	RR	0.71 (0.53, 0.95)	0.86 (0.40, 1.87)	4.9×10^{-1}	0.23	N/A	N/A	N/A	N/A	N/A
	Past	3 (0/3)	548/134,847	RR	0.70 (0.49, 1.01)	0.89 (0.43, 1.83)	5.7×10^{-1}	0.20	N/A	N/A	N/A	N/A	N/A
Primary liver cancer	Ever	5 (4/1)	887/596,239	RR	1.15 (0.81, 1.63)	0.65 (0.30, 1.39)	1.8×10^{-1}	0.47	N/A	N/A	N/A	N/A	N/A
	Current	2 (1/1)	539/588,128	RR	1.28 (0.92, 1.79)	1.03 (0.03, 35.31)	9.2×10^{-1}	0.31	N/A	N/A	N/A	N/A	N/A
	Past	2 (1/1)	539/588,128	RR	1.41 (0.95, 2.09)	0.88 (0.00, 437.84)	8.3×10^{-1}	0.65	N/A	N/A	N/A	N/A	N/A
Lung cancer	Ever	18 (9/9)	13,102/773,738	RR	0.94 (0.87, 1.01)	0.95 (0.85, 1.05)	2.5×10^{-1}	0.11	0.73 to 1.23	28 (0, 50)	67 (0, 100)	33 (0, 72)	6 (0, 33)
	Current	8 (2/6)	7,575/464,343	RR	0.94 (0.87, 1.02)	0.93 (0.78, 1.12)	3.8×10^{-1}	0.13	N/A	N/A	N/A	N/A	N/A
	Past	6 (1/5)	6,725/460,852	RR	0.92 (0.81, 1.04)	0.93 (0.88, 0.99)	3.5×10^{-2}	0	N/A	N/A	N/A	N/A	N/A
Breast cancer													
Incidence	Ever	71 (39/32)	108,678/3,331,883	RR	1.52 (1.48, 1.56)	1.25 (1.19, 1.31)	6.5×10^{-13}	0.14	0.93 to 1.74	0 (N/A, N/A)	8 (1, 17)	92 (79, 97)	77 (62, 89)
	Current	52 (22/30)	82,830/2,958,526	RR	1.68 (1.64, 1.72)	1.43 (1.33, 1.55)	1.2×10^{-11}	0.19	0.87 to 2.33	2 (0, 10)	10 (0, 23)	90 (73, 100)	83 (67, 92)
	Past	46 (20/26)	81,327/2,857,047	RR	1.08 (1.04, 1.12)	1.04 (1.00, 1.08)	4.4×10^{-2}	0.06	0.89 to 1.15	2 (0, 17)	22 (0, 57)	78 (41, 100)	17 (0, 54)
Mortality	Ever	10 (3/7)	4,281/1,368,552	RR	0.84 (0.75, 0.94)	0.95 (0.79, 1.13)	4.8×10^{-1}	0.16	0.70 to 1.49	40 (0, 80)	60 (0, 100)	40 (0, 70)	30 (0, 70)
	Current	8 (3/5)	4,283/1,373,628	RR	1.22 (1.06, 1.41)	1.01 (0.79, 1.29)	9.2×10^{-1}	0.23	N/A	N/A	N/A	N/A	N/A
	Past	7 (3/4)	4,144/1,332,179	RR	0.78 (0.68, 0.89)	0.90 (0.76, 1.07)	1.6×10^{-1}	0.08	N/A	N/A	N/A	N/A	N/A
Endometrial cancer													
Incidence	Ever	23 (10/13)	12,119/1,803,931	RR	1.79 (1.69, 1.90)	2.10 (1.70, 2.59)	4.1×10^{-7}	0.35	0.89 to 5.98	0 (N/A, N/A)	4 (0, 13)	96 (78, 100)	96 (70, 100)
	Current	15 (5/10)	10,877/1,765,059	RR	2.03 (1.89, 2.18)	3.18 (1.84, 5.48)	5.3×10^{-4}	0.67	0.71 to 25.57	0 (N/A, N/A)	7 (0, 20)	93 (67, 100)	87 (47, 93)
	Past	14 (4/10)	10,705/1,763,167	RR	1.54 (1.43, 1.66)	1.56 (1.14, 2.15)	1.0×10^{-2}	0.35	0.67 to 5.40	7 (0, 21)	14 (0, 36)	86 (50, 93)	86 (50, 100)
Mortality	Ever	1 (0/1)	6/6,093	RR	2.60 (0.44, 15.50)	N/A	N/A	N/A	N/A	N/A	N/A	N/A	N/A
Ovarian cancer	Ever	37 (26/11)	16,290/2,389,636	RR	1.23 (1.14, 1.33)	1.16 (1.06, 1.26)	1.9×10^{-3}	0.15	0.71 to 1.54	11 (0, 34)	19 (0, 35)	81 (59, 95)	76 (51, 89)
	Current	15 (4/11)	9,295/2,328,057	RR	1.38 (1.26, 1.51)	1.24 (1.08, 1.44)	6.3×10^{-3}	0.17	0.66 to 2.10	7 (0, 27)	20 (0, 53)	80 (33, 100)	73 (23, 87)
	Past	14 (4/10)	8,980/2,283,816	RR	1.00 (0.89, 1.12)	1.06 (0.95, 1.17)	2.6×10^{-1}	0.11	0.75 to 1.54	7 (0, 36)	29 (0, 100)	71 (0, 100)	36 (0, 93)
**Diseases of the immune system**													
SLE	Ever	3 (2/1)	204/71,061	RR	1.90 (1.16, 3.10)	1.62 (0.67, 3.91)	1.3×10^{-1}	0	N/A	N/A	N/A	N/A	N/A
	Current	3 (2/1)	204/71,061	RR	1.70 (1.00, 2.90)	1.53 (0.80, 2.94)	9.9×10^{-2}	0	N/A	N/A	N/A	N/A	N/A
	Past	3 (2/1)	204/71,061	RR	2.20 (1.24, 3.90)	1.77 (0.29, 10.88)	2.3×10^{-1}	0	N/A	N/A	N/A	N/A	N/A
**Endocrine, nutritional or metabolic diseases**													
Diabetes mellitus	Ever	2 (0/2)	265/9,308	RR	0.63 (0.46, 0.87)	0.81 (0.02, 28.81)	5.9×10^{-1}	0.34	N/A	N/A	N/A	N/A	N/A

	Current	2 (0/2)	265/9,308	RR	0.37 (0.21, 0.64)	0.61 (0.00, 606.60)	5.3×10^{-1}	0.68	N/A	N/A	N/A	N/A	N/A
	Past	2 (0/2)	265/9,308	RR	0.81 (0.57, 1.16)	0.90 (0.14, 5.96)	6.1×10^{-1}	0	N/A	N/A	N/A	N/A	N/A
**Mental or behavioural disorders**													
Dementia	Ever	3 (0/3)	1,799/8,268	RR	0.96 (0.84, 1.09)	0.94 (0.69, 1.28)	2.9×10^{-1}	0	N/A	N/A	N/A	N/A	N/A
	Current	2 (0/2)	362/6,036	RR	1.26 (0.99, 1.61)	1.23 (0.34, 4.37)	2.9×10^{-1}	0	N/A	N/A	N/A	N/A	N/A
	Past	1 (0/1)	79/3,130	RR	0.74 (0.35, 1.55)	N/A	N/A	N/A	N/A	N/A	N/A	N/A	N/A
**Diseases of the nervous system**													
Parkinson disease	Ever	8 (5/3)	1,839/270,396	RR	1.24 (1.00, 1.53)	1.04 (0.79, 1.36)	7.4×10^{-1}	0.21	N/A	N/A	N/A	N/A	N/A
	Current	4 (1/3)	904/268,462	RR	1.29 (1.03, 1.61)	1.23 (0.97, 1.57)	6.3×10^{-2}	0	N/A	N/A	N/A	N/A	N/A
	Past	4 (1/3)	904/268,462	RR	1.08 (0.78, 1.50)	1.23 (0.81, 1.87)	1.9×10^{-1}	0.11	N/A	N/A	N/A	N/A	N/A
Alzheimer disease	Ever	12 (7/5)	1,597/10,568	RR	0.65 (0.48, 0.88)	0.78 (0.63, 0.95)	2.1×10^{-2}	0.17	0.58 to 1.01	67 (0, 100)	100 (0, 100)	0 (N/A, N/A)	0 (N/A, N/A)
	Current	6 (2/4)	685/11,315	RR	1.22 (0.89, 1.68)	1.12 (0.68, 1.83)	4.9×10^{-1}	0.04	N/A	N/A	N/A	N/A	N/A
	Past	3 (1/2)	244/5,223	RR	1.70 (0.90, 3.20)	0.64 (0.04, 9.40)	5.4×10^{-1}	0.99	N/A	N/A	N/A	N/A	N/A
**Diseases of the visual system**													
Cataract	Ever	7 (3/4)	21,354/43,082	RR	0.90 (0.84, 0.97)	0.87 (0.79, 0.97)	2.2×10^{-2}	0.07	N/A	N/A	N/A	N/A	N/A
	Current	4 (2/2)	20,578/41,179	RR	0.88 (0.80, 0.97)	0.88 (0.79, 0.98)	4.0×10^{-2}	0	N/A	N/A	N/A	N/A	N/A
	Past	4 (2/2)	20,578/41,179	RR	0.96 (0.87, 1.06)	0.99 (0.94, 1.05)	5.3×10^{-1}	0	N/A	N/A	N/A	N/A	N/A
**Diseases of the circulatory system**													
Coronary heart disease													
Incidence	Ever	10 (8/2)	5,215/91,067	RR	0.72 (0.64, 0.81)	0.82 (0.69, 0.96)	2.1×10^{-2}	0.12	0.69 to 1.14	80 (0, 100)	90 (0, 100)	10 (0, 50)	0 (N/A, N/A)
	Current	9 (7/2)	6,641/98,395	RR	0.61 (0.52, 0.71)	0.74 (0.62, 0.89)	6.9×10^{-3}	0.11	N/A	N/A	N/A	N/A	N/A
	Past	7 (5/2)	4,924/90,074	RR	0.82 (0.72, 0.94)	0.87 (0.71, 1.05)	1.1×10^{-1}	0.10	N/A	N/A	N/A	N/A	N/A
Mortality	Ever	3 (0/3)	512/49,929	RR	0.67 (0.53, 0.85)	0.67 (0.25, 1.81)	2.0×10^{-1}	0.28	N/A	N/A	N/A	N/A	N/A
	Current	5 (1/4)	1,238/64,273	RR	0.47 (0.32, 0.69)	0.60 (0.33, 1.08)	7.0×10^{-2}	0.34	N/A	N/A	N/A	N/A	N/A
	Past	5 (1/4)	1,188/63,947	RR	0.99 (0.75, 1.30)	0.81 (0.53, 1.26)	2.3×10^{-1}	0.18	N/A	N/A	N/A	N/A	N/A
Venous thromboembolism	Ever	4 (3/1)	505/123,376	RR	1.96 (1.33, 2.89)	1.99 (1.53, 2.58)	5.6×10^{-3}	0	N/A	N/A	N/A	N/A	N/A
	Current	8 (7/1)	1,695/128,625	RR	1.21 (0.97, 1.51)	2.08 (1.40, 3.07)	4.3×10^{-3}	0.39	N/A	N/A	N/A	N/A	N/A
	Past	5 (4/1)	763/124,237	RR	1.10 (0.71, 1.70)	1.17 (0.96, 1.43)	7.7×10^{-2}	0	N/A	N/A	N/A	N/A	N/A
Deep vein thrombosis	Current	3 (3/0)	321/11,055	RR	2.20 (1.34, 3.60)	2.26 (1.14, 4.49)	3.8×10^{-2}	0	N/A	N/A	N/A	N/A	N/A
Pulmonary embolism	Ever	1 (0/1)	68/112,593	RR	1.65 (0.99, 2.74)	N/A	N/A	N/A	N/A	N/A	N/A	N/A	N/A
	Current	3 (2/1)	176/122,745	RR	2.10 (1.16, 3.80)	2.05 (1.49, 2.83)	1.7×10^{-2}	0	N/A	N/A	N/A	N/A	N/A
	Past	1 (0/1)	68/112,593	RR	1.30 (0.70, 2.40)	N/A	N/A	N/A	N/A	N/A	N/A	N/A	N/A
**Diseases of the respiratory system**													
Asthma	Ever	5 (0/5)	1,646/163,161	RR	1.46 (1.21, 1.76)	1.41 (1.09, 1.81)	2.3×10^{-2}	0	N/A	N/A	N/A	N/A	N/A
	Current	5 (0/5)	1,646/163,161	RR	1.20 (0.99, 1.46)	1.48 (1.02, 2.13)	4.3×10^{-2}	0	N/A	N/A	N/A	N/A	N/A
	Past	5 (0/5)	1,646/163,161	RR	1.16 (0.86, 1.57)	1.37 (1.08, 1.73)	2.4×10^{-2}	0	N/A	N/A	N/A	N/A	N/A
**Diseases of the digestive system**													
Cholelithiasis	Ever	8 (4/4)	19,909/277,380	RR	1.48 (1.41, 1.55)	1.63 (1.41, 1.88)	9.4×10^{-4}	0.08	N/A	N/A	N/A	N/A	N/A
	Current	7 (2/5)	20,572/368,310	RR	1.74 (1.64, 1.85)	1.89 (1.58, 2.27)	3.0×10^{-4}	0.14	N/A	N/A	N/A	N/A	N/A
	Past	7 (2/5)	19,719/278,615	RR	1.35 (1.28, 1.42)	1.40 (1.23, 1.59)	5.2×10^{-3}	0.06	N/A	N/A	N/A	N/A	N/A
**Others, not elsewhere classified**													
All-cause mortality	Ever	6 (1/5)	13,770/106,642	RR	0.90 (0.86, 0.94)	0.89 (0.82, 0.97)	2.9×10^{-2}	0	N/A	N/A	N/A	N/A	N/A
	Current	6 (1/5)	14,837/109,822	RR	0.84 (0.77, 0.92)	0.79 (0.66, 0.95)	2.1×10^{-2}	0.16	N/A	N/A	N/A	N/A	N/A
	Past	6 (1/5)	14,605/109,496	RR	0.90 (0.85, 0.95)	0.92 (0.82, 1.03)	1.1×10^{-1}	0.07	N/A	N/A	N/A	N/A	N/A
Cardiovascular disease													
Incidence	Ever	2 (1/1)	1,530/8,954	RR	1.09 (0.93, 1.28)	1.11 (0.56, 2.22)	3.0×10^{-1}	0	N/A	N/A	N/A	N/A	N/A
	Current	2 (0/2)	3,114/77,850	RR	0.77 (0.68, 0.87)	0.90 (0.11, 7.22)	6.3×10^{-1}	0.22	N/A	N/A	N/A	N/A	N/A
	Past	1 (0/1)	1,089/7,317	RR	1.11 (0.89, 1.39)	N/A	N/A	N/A	N/A	N/A	N/A	N/A	N/A
Mortality	Ever	4 (0/4)	604/16,896	RR	0.66 (0.48, 0.90)	0.65 (0.40, 1.06)	6.7×10^{-2}	0.19	N/A	N/A	N/A	N/A	N/A
	Current	4 (0/4)	776/20,402	RR	0.96 (0.64, 1.43)	0.50 (0.18, 1.40)	1.2×10^{-1}	0.51	N/A	N/A	N/A	N/A	N/A
	Past	3 (0/3)	632/17,806	RR	0.86 (0.64, 1.15)	0.88 (0.59, 1.31)	2.1×10^{-1}	0	N/A	N/A	N/A	N/A	N/A

Abbreviations: Cc, case-control study; Co, cohort study; MHT, menopausal hormone therapy; N/A, not available or not applicable; PI, prediction interval; RR, risk ratio; SLE, systemic lupus erythematosus.

[a] Primary prevention refers to reducing the risk of occurrence of a disease among individuals who do not have that disease.

[b] Incidence unless otherwise indicated.

[c] Point estimate with 95% confidence interval of the study with the smallest standard error in each meta-analysis.
[d] Point estimate with 95% confidence interval of robust random-effects meta-analysis.
[e] *P*-value of robust random-effects meta-analysis.
[f] The estimated standard deviation (on log RR scale) of true effects.
[g] The middle 95% area of the estimated effect distribution. 95% PI is reported only in meta-analyses of ≥ 10 studies. More information about how to interpret 95% PI can be found in S3 Text (section 3).
[h] The proportion with 95% confidence interval of true effects (θ) below or above a threshold of scientific importance; complementary to 95% PI, these metrics estimate the area of the lower and upper tails of the effect distribution. They are reported only in meta-analyses of ≥ 10 studies. More information about how to interpret them can be found in S3 Text (section 3).

Table M. Estrogen-Alone Therapy for Primary Prevention of Multiple Outcomes in Included Systematic Reviews and Meta-Analyses of Observational Epidemiological Studies[a]

Outcome[b]	Timing of ET	No. of Studies (Cc/Co)	No. of Cases/Population	Metric	Most Precise Study[c]	Summary Effect[d]	*P*-Value[e]	T[f]	95% PI[g]	Proportion of True Effects (θ) Below/Above a Threshold (%)[h]			
										$\hat{P}$ ($\theta < 0.9$)	$\hat{P}$ ($\theta < 1.0$)	$\hat{P}$ ($\theta > 1.0$)	$\hat{P}$ ($\theta > 1.1$)
**Neoplasms**													
Cutaneous melanoma	Ever	3 (3/0)	995/5,473	RR	2.08 (1.38, 3.14)	1.78 (0.74, 4.28)	9.8×10^{-2}	0.15	N/A	N/A	N/A	N/A	N/A
Glioma	Ever	2 (2/0)	1,347/8,453	RR	1.10 (0.93, 1.30)	1.14 (0.50, 2.58)	2.9×10^{-1}	0	N/A	N/A	N/A	N/A	N/A
	Current	1 (0/1)	557/1,147,894	RR	1.34 (1.04, 1.72)	N/A	N/A	N/A	N/A	N/A	N/A	N/A	N/A
Meningioma	Ever	4 (4/0)	2,292/13,488	RR	1.30 (1.06, 1.60)	1.02 (0.48, 2.17)	9.4×10^{-1}	0.31	N/A	N/A	N/A	N/A	N/A
	Current	4 (3/1)	1,854/1,163,020	RR	1.30 (1.06, 1.60)	1.04 (0.47, 2.31)	8.7×10^{-1}	0.34	N/A	N/A	N/A	N/A	N/A
	Past	2 (2/0)	994/7,230	RR	1.20 (0.76, 1.90)	1.10 (0.20, 5.92)	6.1×10^{-1}	0	N/A	N/A	N/A	N/A	N/A
Thyroid cancer	Ever	5 (1/4)	942/461,912	RR	0.84 (0.63, 1.12)	1.05 (0.68, 1.60)	7.7×10^{-1}	0.23	N/A	N/A	N/A	N/A	N/A
	Current	1 (0/1)	463/290,171	RR	1.34 (0.85, 2.11)	N/A	N/A	N/A	N/A	N/A	N/A	N/A	N/A
Esophageal cancer	Ever	3 (1/2)	1,093/132,186	RR	0.65 (0.45, 0.93)	0.75 (0.13, 4.26)	2.8×10^{-1}	0	N/A	N/A	N/A	N/A	N/A
	Current	1 (0/1)	14/51,515	RR	1.44 (0.28, 7.44)	N/A	N/A	N/A	N/A	N/A	N/A	N/A	N/A
	Past	1 (0/1)	14/51,515	RR	1.72 (0.24, 12.49)	N/A	N/A	N/A	N/A	N/A	N/A	N/A	N/A
Gastric cancer	Ever	3 (1/2)	799/130,359	RR	0.70 (0.44, 1.12)	0.77 (0.32, 1.86)	1.6×10^{-1}	0	N/A	N/A	N/A	N/A	N/A
	Current	1 (0/1)	19/51,515	RR	1.68 (0.44, 6.38)	N/A	N/A	N/A	N/A	N/A	N/A	N/A	N/A
	Past	1 (0/1)	19/51,515	RR	1.80 (0.36, 9.02)	N/A	N/A	N/A	N/A	N/A	N/A	N/A	N/A
Colorectal cancer	Ever	13 (9/4)	17,166/249,282	RR	0.85 (0.76, 0.95)	0.79 (0.71, 0.88)	6.9×10^{-4}	0.12	0.59 to 0.97	85 (31, 100)	100 (N/A, N/A)	0 (N/A, N/A)	0 (N/A, N/A)
	Current	10 (4/6)	6,745/353,397	RR	0.90 (0.74, 1.10)	0.72 (0.61, 0.86)	2.3×10^{-3}	0.17	0.54 to 1.08	90 (50, 100)	100 (N/A, N/A)	0 (N/A, N/A)	0 (N/A, N/A)
	Past	4 (2/2)	3,074/79,127	RR	1.05 (0.86, 1.28)	0.96 (0.57, 1.59)	8.0×10^{-1}	0.26	N/A	N/A	N/A	N/A	N/A
Pancreatic cancer	Ever	4 (3/1)	815/62,973	RR	0.84 (0.64, 1.10)	0.81 (0.53, 1.23)	1.8×10^{-1}	0.10	N/A	N/A	N/A	N/A	N/A
	Current	1 (0/1)	263/60,878	RR	0.59 (0.41, 0.84)	N/A	N/A	N/A	N/A	N/A	N/A	N/A	N/A
Primary liver cancer	Ever	4 (3/1)	646/588,661	RR	1.09 (0.63, 1.88)	0.70 (0.20, 2.39)	3.7×10^{-1}	0.46	N/A	N/A	N/A	N/A	N/A
Lung cancer	Ever	8 (4/4)	4,761/337,385	RR	0.97 (0.86, 1.09)	0.97 (0.83, 1.13)	5.8×10^{-1}	0.11	N/A	N/A	N/A	N/A	N/A
	Current	4 (1/3)	3,389/252,390	RR	0.96 (0.84, 1.10)	0.85 (0.64, 1.13)	1.6×10^{-1}	0.13	N/A	N/A	N/A	N/A	N/A
	Past	2 (0/2)	2,163/190,780	RR	0.98 (0.84, 1.14)	0.93 (0.36, 2.37)	5.0×10^{-1}	0.05	N/A	N/A	N/A	N/A	N/A
Breast cancer													
Incidence	Ever	34 (24/10)	62,837/675,959	RR	1.01 (0.94, 1.09)	1.06 (0.99, 1.14)	7.6×10^{-2}	0	N/A	N/A	N/A	N/A	N/A
	Current	27 (12/15)	64,429/2,486,628	RR	1.38 (1.32, 1.44)	1.16 (1.08, 1.25)	4.0×10^{-4}	0.14	0.80 to 1.50	4 (0, 19)	19 (4, 45)	81 (44, 89)	59 (30, 78)
	Past	19 (10/9)	42,782/1,344,182	RR	1.06 (0.94, 1.19)	1.03 (0.97, 1.10)	3.1×10^{-1}	0.09	0.89 to 1.46	0 (N/A, N/A)	32 (0, 100)	68 (0, 100)	21 (0, 49)
Mortality	Ever	5 (2/3)	3,183/488,161	RR	0.84 (0.75, 0.94)	0.87 (0.68, 1.11)	1.4×10^{-1}	0.07	N/A	N/A	N/A	N/A	N/A
	Current	4 (2/2)	3,131/487,707	RR	1.10 (0.93, 1.30)	0.86 (0.48, 1.54)	3.9×10^{-1}	0.24	N/A	N/A	N/A	N/A	N/A
	Past	4 (2/2)	3,131/487,707	RR	1.00 (0.91, 1.10)	0.90 (0.64, 1.27)	3.3×10^{-1}	0.13	N/A	N/A	N/A	N/A	N/A
Endometrial cancer													
Incidence	Ever	18 (10/8)	5,872/836,988	RR	2.70 (2.14, 3.40)	2.55 (2.05, 3.18)	1.3×10^{-7}	0.36	1.01 to 6.99	0 (N/A, N/A)	0 (N/A, N/A)	100 (N/A, N/A)	100 (N/A, N/A)
	Current	13 (4/9)	10,663/1,756,930	RR	2.70 (2.41, 3.02)	4.75 (3.02, 7.46)	7.9×10^{-6}	0.65	1.31 to 26.91	0 (N/A, N/A)	0 (N/A, N/A)	100 (N/A, N/A)	100 (N/A, N/A)
	Past	10 (4/6)	4,524/804,662	RR	1.88 (1.44, 2.46)	1.84 (1.18, 2.88)	1.3×10^{-2}	0.50	0.67 to 8.47	0 (N/A, N/A)	10 (0, 30)	90 (50, 100)	80 (19, 100)
Mortality	Ever	1 (0/1)	6/6,093	RR	2.60 (0.44, 15.50)	N/A	N/A	N/A	N/A	N/A	N/A	N/A	N/A
Ovarian cancer	Ever	18 (15/3)	6,002/215,733	RR	1.60 (1.28, 2.00)	1.21 (1.01, 1.45)	3.6×10^{-2}	0.30	0.53 to 2.24	17 (0, 33)	17 (0, 33)	83 (44, 94)	78 (39, 94)
	Current	9 (2/7)	7,092/2,161,898	RR	1.31 (1.11, 1.54)	1.36 (1.02, 1.82)	4.1×10^{-2}	0.24	N/A	N/A	N/A	N/A	N/A
	Past	4 (2/2)	1,429/154,169	RR	1.04 (0.81, 1.34)	0.94 (0.60, 1.46)	6.6×10^{-1}	0.18	N/A	N/A	N/A	N/A	N/A
**Mental or behavioural disorders**													
Dementia	Ever	1 (0/1)	248/1,768	RR	0.79 (0.58, 1.07)	N/A	N/A	N/A	N/A	N/A	N/A	N/A	N/A
	Current	1 (0/1)	283/2,906	RR	1.23 (0.95, 1.59)	N/A	N/A	N/A	N/A	N/A	N/A	N/A	N/A
**Diseases of the nervous system**													
Parkinson disease	Ever	6 (4/2)	856/207,200	RR	1.28 (0.92, 1.78)	0.97 (0.46, 2.03)	9.2×10^{-1}	0.44	N/A	N/A	N/A	N/A	N/A
	Current	4 (2/2)	541/112,661	RR	1.47 (0.98, 2.20)	1.23 (0.44, 3.42)	4.5×10^{-1}	0	N/A	N/A	N/A	N/A	N/A
	Past	2 (1/1)	354/84,971	RR	0.81 (0.47, 1.41)	1.44 (0.00, ∞)	6.7×10^{-1}	0.82	N/A	N/A	N/A	N/A	N/A
Alzheimer disease	Ever	10 (6/4)	1,451/8,419	RR	0.65 (0.48, 0.88)	0.76 (0.60, 0.96)	2.8×10^{-2}	0.17	0.56 to 1.03	70 (0, 100)	100 (0, 100)	0 (N/A, N/A)	0 (N/A, N/A)
	Current	3 (2/1)	357/3,413	RR	1.22 (0.89, 1.68)	0.95 (0.25, 3.60)	8.6×10^{-1}	0.27	N/A	N/A	N/A	N/A	N/A
	Past	1 (1/0)	107/227	RR	1.70 (0.90, 3.20)	N/A	N/A	N/A	N/A	N/A	N/A	N/A	N/A
**Diseases of the visual system**													

Cataract	Ever	5 (2/3)	11,086/21,950	RR	0.90 (0.84, 0.97)	0.84 (0.70, 1.02)	6.8×10^{-2}	0.10	N/A	N/A	N/A	N/A	N/A
	Current	1 (1/0)	10,000/20,000	RR	0.88 (0.80, 0.97)	N/A	N/A	N/A	N/A	N/A	N/A	N/A	N/A
	Past	1 (1/0)	10,000/20,000	RR	0.96 (0.87, 1.06)	N/A	N/A	N/A	N/A	N/A	N/A	N/A	N/A
**Diseases of the circulatory system**													
Coronary heart disease													
Incidence	Ever	4 (4/0)	1,231/3,463	RR	0.90 (0.68, 1.20)	0.83 (0.58, 1.19)	1.5×10^{-1}	0	N/A	N/A	N/A	N/A	N/A
	Current	7 (6/1)	5,375/88,254	RR	0.55 (0.44, 0.68)	0.63 (0.45, 0.90)	2.5×10^{-2}	0.07	N/A	N/A	N/A	N/A	N/A
	Past	2 (2/0)	949/2,511	RR	0.90 (0.62, 1.30)	0.87 (0.22, 3.51)	4.3×10^{-1}	0	N/A	N/A	N/A	N/A	N/A
Mortality	Ever	1 (0/1)	37/1,542	RR	1.14 (0.53, 2.44)	N/A	N/A	N/A	N/A	N/A	N/A	N/A	N/A
	Current	1 (0/1)	87/1,868	RR	0.99 (0.59, 1.67)	N/A	N/A	N/A	N/A	N/A	N/A	N/A	N/A
	Past	1 (0/1)	37/1,542	RR	1.24 (0.55, 2.78)	N/A	N/A	N/A	N/A	N/A	N/A	N/A	N/A
Venous thromboembolism	Ever	1 (0/1)	68/112,593	RR	1.65 (0.99, 2.74)	N/A	N/A	N/A	N/A	N/A	N/A	N/A	N/A
	Current	6 (5/1)	1,179/126,903	RR	1.01 (0.76, 1.34)	1.88 (1.04, 3.40)	4.1×10^{-2}	0.49	N/A	N/A	N/A	N/A	N/A
	Past	1 (0/1)	68/112,593	RR	1.30 (0.70, 2.40)	N/A	N/A	N/A	N/A	N/A	N/A	N/A	N/A
Deep vein thrombosis	Current	1 (1/0)	95/705	RR	1.22 (0.57, 2.61)	N/A	N/A	N/A	N/A	N/A	N/A	N/A	N/A
Pulmonary embolism	Ever	1 (0/1)	68/112,593	RR	1.65 (0.99, 2.74)	N/A	N/A	N/A	N/A	N/A	N/A	N/A	N/A
	Current	1 (0/1)	68/112,593	RR	2.10 (1.16, 3.80)	N/A	N/A	N/A	N/A	N/A	N/A	N/A	N/A
	Past	1 (0/1)	68/112,593	RR	1.30 (0.70, 2.40)	N/A	N/A	N/A	N/A	N/A	N/A	N/A	N/A
**Diseases of the respiratory system**													
Asthma	Ever	1 (0/1)	569/57,664	RR	1.54 (1.13, 2.09)	N/A	N/A	N/A	N/A	N/A	N/A	N/A	N/A
	Current	4 (0/4)	1,433/158,667	RR	1.88 (1.44, 2.45)	1.86 (1.44, 2.39)	6.9×10^{-3}	0	N/A	N/A	N/A	N/A	N/A
	Past	1 (0/1)	569/57,664	RR	1.04 (0.51, 2.12)	N/A	N/A	N/A	N/A	N/A	N/A	N/A	N/A
**Diseases of the digestive system**													
Cholelithiasis	Ever	4 (3/1)	16,883/187,128	RR	1.65 (1.56, 1.75)	1.66 (1.28, 2.14)	2.5×10^{-2}	0	N/A	N/A	N/A	N/A	N/A
	Current	4 (1/3)	19,219/332,903	RR	1.92 (1.76, 2.10)	2.01 (1.69, 2.40)	2.8×10^{-3}	0.06	N/A	N/A	N/A	N/A	N/A
	Past	3 (1/2)	16,693/188,363	RR	1.49 (1.38, 1.61)	1.37 (0.73, 2.58)	1.5×10^{-1}	0.17	N/A	N/A	N/A	N/A	N/A
**Others, not elsewhere classified**													
All-cause mortality	Ever	2 (0/2)	351/7,635	RR	0.80 (0.64, 1.00)	0.81 (0.59, 1.12)	7.7×10^{-2}	0	N/A	N/A	N/A	N/A	N/A
	Current	3 (1/2)	5,055/49,077	RR	0.69 (0.60, 0.80)	0.72 (0.57, 0.90)	2.7×10^{-2}	0	N/A	N/A	N/A	N/A	N/A
	Past	1 (0/1)	132/1,542	RR	0.88 (0.57, 1.36)	N/A	N/A	N/A	N/A	N/A	N/A	N/A	N/A
Cardiovascular disease													
Incidence	Ever	1 (1/0)	441/1,637	RR	1.09 (0.65, 1.82)	N/A	N/A	N/A	N/A	N/A	N/A	N/A	N/A
	Current	1 (0/1)	2,025/70,533	RR	0.75 (0.65, 0.87)	N/A	N/A	N/A	N/A	N/A	N/A	N/A	N/A
Mortality	Ever	2 (0/2)	93/7,635	RR	0.60 (0.33, 1.10)	0.81 (0.02, 40.26)	6.2×10^{-1}	0.30	N/A	N/A	N/A	N/A	N/A
	Current	2 (0/2)	197/4,138	RR	0.96 (0.64, 1.43)	0.72 (0.00, 108.73)	5.5×10^{-1}	0.45	N/A	N/A	N/A	N/A	N/A
	Past	1 (0/1)	53/1,542	RR	1.20 (0.61, 2.35)	N/A	N/A	N/A	N/A	N/A	N/A	N/A	N/A

Abbreviations: Cc, case-control study; Co, cohort study; ET, estrogen-alone therapy; N/A, not available or not applicable; PI, prediction interval; RR, risk ratio.

[a] Primary prevention refers to reducing the risk of occurrence of a disease among individuals who do not have that disease.

[b] Incidence unless otherwise indicated.

[c] Point estimate with 95% confidence interval of the study with the smallest standard error in each meta-analysis.

[d] Point estimate with 95% confidence interval of robust random-effects meta-analysis.

[e] *P*-value of robust random-effects meta-analysis.

[f] The estimated standard deviation (on log RR scale) of true effects.

[g] The middle 95% area of the estimated effect distribution. 95% PI is reported only in meta-analyses of ≥ 10 studies. More information about how to interpret 95% PI can be found in S3 Text (section 3).

[h] The proportion with 95% confidence interval of true effects (θ) below or above a threshold of scientific importance; complementary to 95% PI, these metrics estimate the area of the lower and upper tails of the effect distribution. They are reported only in meta-analyses of ≥ 10 studies. More information about how to interpret them can be found in S3 Text (section 3).

Table N. Estrogen Plus Progestin Therapy for Primary Prevention of Multiple Outcomes in Included Systematic Reviews and Meta-Analyses of Observational Epidemiological Studies[a]

Outcome[b]	Timing of EPT	No. of Studies (Cc/Co)	No. of Cases/Population	Metric	Most Precise Study[c]	Summary Effect[d]	*P*-Value[e]	T[f]	95% PI[g]	Proportion of True Effects (θ) Below/Above a Threshold (%)[h]			
										$\hat{P}$ ($\theta < 0.9$)	$\hat{P}$ ($\theta < 1.0$)	$\hat{P}$ ($\theta > 1.0$)	$\hat{P}$ ($\theta > 1.1$)
**Neoplasms**													
Cutaneous melanoma	Ever	1 (1/0)	151/448	RR	1.50 (0.80, 2.80)	N/A	N/A	N/A	N/A	N/A	N/A	N/A	N/A
Glioma	Ever	2 (2/0)	1,347/8,453	RR	0.96 (0.71, 1.29)	0.90 (0.29, 2.75)	4.3×10^{-1}	0	N/A	N/A	N/A	N/A	N/A
	Current	1 (0/1)	557/1,147,894	RR	0.94 (0.72, 1.23)	N/A	N/A	N/A	N/A	N/A	N/A	N/A	N/A
Meningioma	Ever	3 (3/0)	2,222/13,304	RR	1.16 (0.92, 1.46)	1.21 (0.82, 1.78)	1.2×10^{-1}	0	N/A	N/A	N/A	N/A	N/A
	Current	3 (2/1)	1,784/1,162,836	RR	1.21 (0.86, 1.71)	1.14 (0.96, 1.34)	8.1×10^{-2}	0	N/A	N/A	N/A	N/A	N/A
	Past	1 (1/0)	924/7,046	RR	1.29 (0.79, 2.10)	N/A	N/A	N/A	N/A	N/A	N/A	N/A	N/A
Thyroid cancer	Ever	5 (1/4)	942/461,912	RR	0.97 (0.71, 1.32)	1.09 (0.79, 1.50)	4.7×10^{-1}	0	N/A	N/A	N/A	N/A	N/A
Esophageal cancer	Ever	3 (1/2)	1,093/132,186	RR	0.77 (0.57, 1.04)	0.75 (0.62, 0.91)	3.3×10^{-2}	0	N/A	N/A	N/A	N/A	N/A
	Current	1 (0/1)	25/74,372	RR	0.43 (0.14, 1.31)	N/A	N/A	N/A	N/A	N/A	N/A	N/A	N/A
	Past	1 (0/1)	25/74,372	RR	0.36 (0.05, 2.74)	N/A	N/A	N/A	N/A	N/A	N/A	N/A	N/A
Gastric cancer	Ever	3 (1/2)	799/130,359	RR	0.83 (0.56, 1.24)	0.70 (0.11, 4.30)	2.4×10^{-1}	0	N/A	N/A	N/A	N/A	N/A
	Current	1 (0/1)	30/74,372	RR	0.43 (0.14, 1.30)	N/A	N/A	N/A	N/A	N/A	N/A	N/A	N/A
	Past	1 (0/1)	30/74,372	RR	0.34 (0.05, 2.51)	N/A	N/A	N/A	N/A	N/A	N/A	N/A	N/A
Colorectal cancer	Ever	10 (7/3)	15,238/236,085	RR	0.83 (0.73, 0.94)	0.79 (0.71, 0.87)	3.1×10^{-3}	0	N/A	N/A	N/A	N/A	N/A
	Current	9 (4/5)	6,503/356,228	RR	0.94 (0.78, 1.14)	0.86 (0.73, 1.00)	5.1×10^{-2}	0.06	N/A	N/A	N/A	N/A	N/A
	Past	3 (2/1)	2,825/71,426	RR	0.88 (0.60, 1.29)	0.78 (0.38, 1.62)	2.5×10^{-1}	0	N/A	N/A	N/A	N/A	N/A
Pancreatic cancer	Ever	1 (0/1)	263/60,878	RR	0.81 (0.58, 1.14)	N/A	N/A	N/A	N/A	N/A	N/A	N/A	N/A
	Current	1 (0/1)	263/60,878	RR	0.84 (0.60, 1.17)	N/A	N/A	N/A	N/A	N/A	N/A	N/A	N/A
Primary liver cancer	Ever	2 (1/1)	539/588,128	RR	0.63 (0.36, 1.09)	0.68 (0.20, 2.35)	1.6×10^{-1}	0	N/A	N/A	N/A	N/A	N/A
Lung cancer	Ever	6 (2/4)	4,394/286,895	RR	1.03 (0.91, 1.17)	1.03 (0.85, 1.25)	7.0×10^{-1}	0.09	N/A	N/A	N/A	N/A	N/A
	Current	4 (1/3)	2,922/201,963	RR	0.97 (0.83, 1.13)	0.86 (0.64, 1.15)	1.7×10^{-1}	0.11	N/A	N/A	N/A	N/A	N/A
	Past	2 (0/2)	1,696/140,353	RR	1.19 (0.98, 1.45)	1.14 (0.50, 2.59)	2.8×10^{-1}	0	N/A	N/A	N/A	N/A	N/A
Breast cancer													
Incidence	Ever	34 (22/12)	58,983/624,400	RR	1.44 (1.35, 1.54)	1.40 (1.30, 1.51)	1.1×10^{-9}	0.14	1.03 to 2.12	0 (N/A, N/A)	0 (N/A, N/A)	100 (82, 100)	91 (68, 100)
	Current	26 (10/16)	61,010/2,438,210	RR	1.96 (1.90, 2.02)	1.75 (1.55, 1.98)	1.3×10^{-9}	0.21	0.96 to 2.84	0 (N/A, N/A)	0 (N/A, N/A)	100 (77, 100)	92 (62, 100)
	Past	16 (8/8)	37,921/1,217,291	RR	1.04 (0.93, 1.16)	1.07 (0.97, 1.18)	1.4×10^{-1}	0.11	0.81 to 1.40	6 (0, 31)	25 (0, 69)	75 (0, 94)	44 (0, 75)
Mortality	Ever	2 (2/0)	1,566/16,317	RR	0.90 (0.74, 1.10)	0.87 (0.36, 2.09)	2.9×10^{-1}	0	N/A	N/A	N/A	N/A	N/A
	Current	3 (2/1)	1,705/57,766	RR	0.90 (0.62, 1.30)	0.98 (0.49, 1.94)	9.1×10^{-1}	0.19	N/A	N/A	N/A	N/A	N/A
	Past	2 (2/0)	1,566/16,317	RR	0.80 (0.58, 1.10)	0.79 (0.51, 1.22)	9.1×10^{-2}	0	N/A	N/A	N/A	N/A	N/A
Endometrial cancer	Ever	8 (2/6)	4,210/824,680	RR	0.94 (0.82, 1.08)	1.30 (0.93, 1.83)	1.0×10^{-1}	0.31	N/A	N/A	N/A	N/A	N/A
	Current	8 (1/7)	9,103/1,735,287	RR	1.71 (1.57, 1.86)	1.30 (0.93, 1.82)	1.0×10^{-1}	0.35	N/A	N/A	N/A	N/A	N/A
	Past	5 (1/4)	2,964/783,019	RR	0.97 (0.79, 1.19)	1.07 (0.62, 1.83)	7.3×10^{-1}	0.27	N/A	N/A	N/A	N/A	N/A
Ovarian cancer	Ever	11 (8/3)	4,804/188,492	RR	1.39 (1.07, 1.81)	1.18 (1.05, 1.32)	1.2×10^{-2}	0	N/A	N/A	N/A	N/A	N/A
	Current	8 (1/7)	6,903/2,137,296	RR	1.50 (1.34, 1.68)	1.28 (1.07, 1.53)	1.7×10^{-2}	0.14	N/A	N/A	N/A	N/A	N/A
	Past	4 (1/3)	1,555/173,808	RR	1.40 (0.86, 2.28)	1.71 (0.96, 3.05)	6.0×10^{-2}	0.10	N/A	N/A	N/A	N/A	N/A
**Mental or behavioural disorders**													
Dementia	Ever	1 (0/1)	248/1,768	RR	0.93 (0.64, 1.35)	N/A	N/A	N/A	N/A	N/A	N/A	N/A	N/A
	Current	1 (0/1)	283/2,906	RR	1.34 (0.95, 1.89)	N/A	N/A	N/A	N/A	N/A	N/A	N/A	N/A
**Diseases of the nervous system**													
Parkinson disease	Ever	2 (0/2)	527/206,535	RR	1.47 (1.09, 1.98)	1.22 (0.09, 16.88)	5.1×10^{-1}	0.23	N/A	N/A	N/A	N/A	N/A
	Current	3 (1/2)	474/112,524	RR	1.63 (1.15, 2.31)	1.27 (0.41, 3.97)	4.2×10^{-1}	0.31	N/A	N/A	N/A	N/A	N/A
	Past	1 (0/1)	287/84,834	RR	1.10 (0.71, 1.70)	N/A	N/A	N/A	N/A	N/A	N/A	N/A	N/A
Alzheimer disease	Ever	1 (0/1)	176/1,768	RR	0.93 (0.60, 1.43)	N/A	N/A	N/A	N/A	N/A	N/A	N/A	N/A
	Current	2 (1/1)	250/3,186	RR	1.41 (0.94, 2.12)	1.42 (1.24, 1.62)	2.0×10^{-2}	0	N/A	N/A	N/A	N/A	N/A
**Diseases of the visual system**													
Cataract	Ever	2 (1/1)	10,236/20,699	RR	0.93 (0.85, 1.01)	0.93 (0.82, 1.04)	7.8×10^{-2}	0	N/A	N/A	N/A	N/A	N/A
	Current	1 (1/0)	10,000/20,000	RR	0.85 (0.76, 0.96)	N/A	N/A	N/A	N/A	N/A	N/A	N/A	N/A
	Past	1 (1/0)	10,000/20,000	RR	1.04 (0.92, 1.17)	N/A	N/A	N/A	N/A	N/A	N/A	N/A	N/A
**Diseases of the circulatory system**													

Coronary heart disease	Ever	1 (1/0)	846/1,687	RR	1.20 (0.60, 2.40)	N/A	N/A	N/A	N/A	N/A	N/A	N/A	N/A
	Current	4 (3/1)	4,230/85,027	RR	0.64 (0.48, 0.85)	0.71 (0.56, 0.91)	2.4×10^{-2}	0	N/A	N/A	N/A	N/A	N/A
Venous thromboembolism	Current	5 (5/0)	1,111/14,310	RR	1.48 (1.12, 1.95)	2.22 (1.19, 4.14)	2.6×10^{-2}	0.31	N/A	N/A	N/A	N/A	N/A
Deep vein thrombosis	Current	1 (1/0)	95/705	RR	2.70 (1.44, 5.07)	N/A	N/A	N/A	N/A	N/A	N/A	N/A	N/A
**Diseases of the respiratory system**													
Asthma	Ever	1 (0/1)	569/57,664	RR	1.12 (0.92, 1.36)	N/A	N/A	N/A	N/A	N/A	N/A	N/A	N/A
	Current	4 (0/4)	1,433/158,667	RR	1.14 (0.93, 1.40)	1.46 (0.92, 2.31)	7.9×10^{-2}	0.20	N/A	N/A	N/A	N/A	N/A
	Past	1 (0/1)	569/57,664	RR	0.97 (0.64, 1.46)	N/A	N/A	N/A	N/A	N/A	N/A	N/A	N/A
**Diseases of the digestive system**													
Cholelithiasis	Ever	1 (1/0)	16,386/180,246	RR	1.40 (1.33, 1.47)	N/A	N/A	N/A	N/A	N/A	N/A	N/A	N/A
	Current	2 (1/1)	18,059/235,091	RR	1.66 (1.53, 1.80)	1.67 (1.28, 2.18)	2.6×10^{-2}	0	N/A	N/A	N/A	N/A	N/A
	Past	1 (1/0)	16,386/180,246	RR	1.28 (1.20, 1.36)	N/A	N/A	N/A	N/A	N/A	N/A	N/A	N/A
**Others, not elsewhere classified**													
All-cause mortality	Current	2 (1/1)	4,691/47,209	RR	0.46 (0.36, 0.58)	0.59 (0.02, 21.95)	3.1×10^{-1}	0.35	N/A	N/A	N/A	N/A	N/A
Cardiovascular disease	Ever	1 (1/0)	441/1,637	RR	1.16 (0.43, 3.12)	N/A	N/A	N/A	N/A	N/A	N/A	N/A	N/A
	Current	1 (0/1)	2,025/70,533	RR	0.91 (0.75, 1.11)	N/A	N/A	N/A	N/A	N/A	N/A	N/A	N/A

Abbreviations: Cc, case-control study; Co, cohort study; EPT, estrogen plus progestin therapy; N/A, not available or not applicable; PI, prediction interval; RR, risk ratio.
[a] Primary prevention refers to reducing the risk of occurrence of a disease among individuals who do not have that disease.
[b] Incidence unless otherwise indicated.
[c] Point estimate with 95% confidence interval of the study with the smallest standard error in each meta-analysis.
[d] Point estimate with 95% confidence interval of robust random-effects meta-analysis.
[e] *P*-value of robust random-effects meta-analysis.
[f] The estimated standard deviation (on log RR scale) of true effects.
[g] The middle 95% area of the estimated effect distribution. 95% PI is reported only in meta-analyses of ≥ 10 studies. More information about how to interpret 95% PI can be found in S3 Text (section 3).
[h] The proportion with 95% confidence interval of true effects (θ) below or above a threshold of scientific importance; complementary to 95% PI, these metrics estimate the area of the lower and upper tails of the effect distribution. They are reported only in meta-analyses of ≥ 10 studies. More information about how to interpret them can be found in S3 Text (section 3).

Table O. Any Menopausal Hormone Therapy for Secondary Prevention of Multiple Outcomes in Included Systematic Reviews and Meta-Analyses of Observational Epidemiological Studies[a]

Outcome[b]	Timing of MHT	No. of Studies (Cc/Co)	No. of Cases/Population	Metric	Most Precise Study[c]	Summary Effect[d]	*P*-Value[e]	T[f]	95% PI[g]	Proportion of True Effects (θ) Below/Above a Threshold (%)[h] $\hat{P}$ ($\theta < 0.9$)	$\hat{P}$ ($\theta < 1.0$)	$\hat{P}$ ($\theta > 1.0$)	$\hat{P}$ ($\theta > 1.1$)
**Neoplasms**													
Lung cancer overall survival	Ever	3 (0/3)	N/A/1,972	RR	0.77 (0.65, 0.92)	1.06 (0.45, 2.51)	8.0×10^{-1}	0.29	N/A	N/A	N/A	N/A	N/A
	Current	1 (0/1)	N/A/454	RR	0.91 (0.76, 1.09)	N/A	N/A	N/A	N/A	N/A	N/A	N/A	N/A
Breast cancer													
Recurrence	Ever	2 (0/2)	408/1,991	RR	0.72 (0.57, 0.91)	0.69 (0.29, 1.61)	1.1×10^{-1}	0	N/A	N/A	N/A	N/A	N/A
Specific survival	Ever	11 (0/11)	3,174/24,753	RR	0.87 (0.77, 0.98)	0.72 (0.59, 0.88)	6.1×10^{-3}	0.17	0.48 to 0.93	100 (N/A, N/A)	100 (N/A, N/A)	0 (N/A, N/A)	0 (N/A, N/A)
	Current	11 (0/11)	3,561/34,022	RR	0.85 (0.74, 0.98)	0.74 (0.62, 0.88)	6.0×10^{-3}	0.08	0.62 to 0.86	100 (N/A, N/A)	100 (N/A, N/A)	0 (N/A, N/A)	0 (N/A, N/A)
	Past	7 (0/7)	2,850/22,070	RR	0.92 (0.78, 1.08)	0.91 (0.70, 1.18)	3.6×10^{-1}	0.12	N/A	N/A	N/A	N/A	N/A
Overall survival	Ever	16 (0/16)	12,969/39,593	RR	0.86 (0.82, 0.91)	0.82 (0.75, 0.89)	2.9×10^{-4}	0.10	0.59 to 1.06	81 (38, 94)	94 (69, 100)	6 (0, 31)	0 (N/A, N/A)
	Current	7 (0/7)	7,763/33,414	RR	0.82 (0.76, 0.88)	0.79 (0.73, 0.86)	1.7×10^{-3}	0	N/A	N/A	N/A	N/A	N/A
	Past	4 (0/4)	6,652/22,526	RR	0.91 (0.85, 0.97)	0.92 (0.73, 1.16)	1.7×10^{-1}	0	N/A	N/A	N/A	N/A	N/A
Ovarian cancer													
Recurrence	Ever	2 (0/2)	40/483	RR	0.93 (0.64, 1.35)	0.87 (0.33, 2.29)	3.2×10^{-1}	0	N/A	N/A	N/A	N/A	N/A
Overall survival	Ever	3 (0/3)	187/599	RR	0.80 (0.57, 1.13)	0.81 (0.71, 0.91)	2.5×10^{-2}	0	N/A	N/A	N/A	N/A	N/A

Abbreviations: Cc, case-control study; Co, cohort study; MHT, menopausal hormone therapy; N/A, not available or not applicable; PI, prediction interval; RR, risk ratio.
[a] Secondary prevention refers to reducing the risk of recurrence of a disease among individuals who already have that disease.
[b] Incidence unless otherwise indicated.
[c] Point estimate with 95% confidence interval of the study with the smallest standard error in each meta-analysis.
[d] Point estimate with 95% confidence interval of robust random-effects meta-analysis.
[e] *P*-value of robust random-effects meta-analysis.
[f] The estimated standard deviation (on log RR scale) of true effects.
[g] The middle 95% area of the estimated effect distribution. 95% PI is reported only in meta-analyses of ≥ 10 studies. More information about how to interpret 95% PI can be found in S3 Text (section 3).
[h] The proportion with 95% confidence interval of true effects (θ) below or above a threshold of scientific importance; complementary to 95% PI, these metrics estimate the area of the lower and upper tails of the effect distribution. They are reported only in meta-analyses of ≥ 10 studies. More information about how to interpret them can be found in S3 Text (section 3).

Table P. Estrogen-Alone Therapy for Secondary Prevention of Multiple Outcomes in Included Systematic Reviews and Meta-Analyses of Observational Epidemiological Studies[a]

Outcome[b]	Timing of ET	No. of Studies (Cc/Co)	No. of Cases/Population	Metric	Most Precise Study[c]	Summary Effect[d]	*P*-Value[e]	T[f]	95% PI[g]	Proportion of True Effects (θ) Below/Above a Threshold (%)[h]			
										$\widehat{P}$ ($\theta < 0.9$)	$\widehat{P}$ ($\theta < 1.0$)	$\widehat{P}$ ($\theta > 1.0$)	$\widehat{P}$ ($\theta > 1.1$)
**Neoplasms**													
Lung cancer overall survival	Ever	1 (0/1)	N/A/484	RR	0.85 (0.70, 1.03)	N/A	N/A	N/A	N/A	N/A	N/A	N/A	N/A
Breast cancer													
Recurrence	Ever	2 (0/2)	386/1,790	RR	0.63 (0.42, 0.95)	0.59 (0.18, 1.95)	1.1×10^{-1}	0	N/A	N/A	N/A	N/A	N/A
Specific survival	Ever	4 (0/4)	2,161/15,970	RR	0.89 (0.78, 1.02)	0.86 (0.56, 1.31)	1.8×10^{-1}	0.11	N/A	N/A	N/A	N/A	N/A
	Current	6 (0/6)	2,990/25,988	RR	0.91 (0.76, 1.09)	0.94 (0.80, 1.11)	2.6×10^{-1}	0	N/A	N/A	N/A	N/A	N/A
	Past	2 (0/2)	1,973/14,180	RR	0.86 (0.70, 1.05)	0.87 (0.65, 1.16)	1.0×10^{-1}	0	N/A	N/A	N/A	N/A	N/A
Overall survival	Ever	4 (0/4)	6,508/20,937	RR	0.87 (0.82, 0.93)	0.83 (0.59, 1.16)	1.3×10^{-1}	0.09	N/A	N/A	N/A	N/A	N/A
	Current	4 (0/4)	5,154/21,890	RR	0.86 (0.78, 0.94)	0.88 (0.65, 1.18)	1.3×10^{-1}	0	N/A	N/A	N/A	N/A	N/A
	Past	1 (0/1)	3,953/12,269	RR	0.89 (0.82, 0.96)	N/A	N/A	N/A	N/A	N/A	N/A	N/A	N/A

Abbreviations: Cc, case-control study; Co, cohort study; ET, estrogen-alone therapy; N/A, not available or not applicable; PI, prediction interval; RR, risk ratio.

[a] Secondary prevention refers to reducing the risk of recurrence of a disease among individuals who already have that disease.

[b] Incidence unless otherwise indicated.

[c] Point estimate with 95% confidence interval of the study with the smallest standard error in each meta-analysis.

[d] Point estimate with 95% confidence interval of robust random-effects meta-analysis.

[e] *P*-value of robust random-effects meta-analysis.

[f] The estimated standard deviation (on log RR scale) of true effects.

[g] The middle 95% area of the estimated effect distribution. 95% PI is reported only in meta-analyses of ≥ 10 studies. More information about how to interpret 95% PI can be found in S3 Text (section 3).

[h] The proportion with 95% confidence interval of true effects (θ) below or above a threshold of scientific importance; complementary to 95% PI, these metrics estimate the area of the lower and upper tails of the effect distribution. They are reported only in meta-analyses of ≥ 10 studies. More information about how to interpret them can be found in S3 Text (section 3).

Table Q. Estrogen Plus Progestin Therapy for Secondary Prevention of Multiple Outcomes in Included Systematic Reviews and Meta-Analyses of Observational Epidemiological Studies[a]

Outcome[b]	Timing of EPT	No. of Studies (Cc/Co)	No. of Cases/Population	Metric	Most Precise Study[c]	Summary Effect[d]	*P*-Value[e]	T[f]	95% PI[g]	Proportion of True Effects (θ) Below/Above a Threshold (%)[h]			
										$\hat{P}$ ($\theta < 0.9$)	$\hat{P}$ ($\theta < 1.0$)	$\hat{P}$ ($\theta > 1.0$)	$\hat{P}$ ($\theta > 1.1$)
**Neoplasms**													
Lung cancer overall survival	Ever	1 (0/1)	N/A/484	RR	0.73 (0.60, 0.90)	N/A	N/A	N/A	N/A	N/A	N/A	N/A	N/A
Breast cancer													
Recurrence	Ever	2 (0/2)	308/1,277	RR	0.86 (0.63, 1.18)	0.78 (0.07, 8.26)	4.1×10^{-1}	0.14	N/A	N/A	N/A	N/A	N/A
Specific survival	Ever	4 (0/4)	2,116/15,457	RR	0.73 (0.59, 0.91)	0.71 (0.45, 1.11)	6.8×10^{-2}	0	N/A	N/A	N/A	N/A	N/A
	Current	6 (0/6)	2,990/25,988	RR	0.69 (0.54, 0.88)	0.66 (0.62, 0.72)	3.0×10^{-4}	0	N/A	N/A	N/A	N/A	N/A
	Past	2 (0/2)	1,973/14,180	RR	0.96 (0.61, 1.50)	0.87 (0.20, 3.77)	4.4×10^{-1}	0	N/A	N/A	N/A	N/A	N/A
Overall survival	Ever	6 (0/6)	8,180/22,932	RR	0.77 (0.68, 0.86)	0.73 (0.46, 1.16)	1.2×10^{-1}	0.21	N/A	N/A	N/A	N/A	N/A
	Current	4 (0/4)	5,154/21,890	RR	0.71 (0.63, 0.80)	0.71 (0.68, 0.74)	9.4×10^{-4}	0	N/A	N/A	N/A	N/A	N/A
	Past	1 (0/1)	3,953/12,269	RR	1.01 (0.82, 1.24)	N/A	N/A	N/A	N/A	N/A	N/A	N/A	N/A

Abbreviations: Cc, case-control study; Co, cohort study; EPT, estrogen plus progestin therapy; N/A, not available or not applicable; PI, prediction interval; RR, risk ratio.

[a] Secondary prevention refers to reducing the risk of recurrence of a disease among individuals who already have that disease.

[b] Incidence unless otherwise indicated.

[c] Point estimate with 95% confidence interval of the study with the smallest standard error in each meta-analysis.

[d] Point estimate with 95% confidence interval of robust random-effects meta-analysis.

[e] *P*-value of robust random-effects meta-analysis.

[f] The estimated standard deviation (on log RR scale) of true effects.

[g] The middle 95% area of the estimated effect distribution. 95% PI is reported only in meta-analyses of ≥ 10 studies. More information about how to interpret 95% PI can be found in S3 Text (section 3).

[h] The proportion with 95% confidence interval of true effects (θ) below or above a threshold of scientific importance; complementary to 95% PI, these metrics estimate the area of the lower and upper tails of the effect distribution. They are reported only in meta-analyses of ≥ 10 studies. More information about how to interpret them can be found in S3 Text (section 3).

Table R. Assessment of Small-Study Effects and Publication Bias: Any Menopausal Hormone Therapy for Primary Prevention of Multiple Outcomes in Included Systematic Reviews and Meta-Analyses of Randomized Controlled Trials

Outcome[a]	Metric (unit)	Small-Study Effects[b]	Uncorrected[c]	Vevea & Hedges Selection Model[d]		Severity of Publication Bias (η) Required to "Explain Away" Results[e]				
				Corrected	LRT *P*-Value	Worst-Case	$\eta(\hat{\mu}, \text{null})$	$\eta(\hat{\mu}^{ci}, \text{null})$	$\eta(\hat{\mu}, q)$	$\eta(\hat{\mu}^{ci}, q)$
**Neoplasms**										
All cancer										
Incidence	RR	No evidence	1.01 (0.70, 1.45)	N/A	N/A	1.01 (0.70, 1.45)	Not possible	1	N/A	N/A
Mortality	RR	N/A	1.03 (0.80, 1.34)	N/A	N/A	1.03 (0.80, 1.34)	Not possible	1	N/A	N/A
Colorectal cancer										
Incidence	RR	N/A	0.91 (0.46, 1.77)	N/A	N/A	0.91 (0.46, 1.77)	Not possible	1	N/A	N/A
Mortality	RR	N/A	1.09 (0.36, 3.28)	N/A	N/A	1.09 (0.36, 3.28)	Not possible	1	N/A	N/A
Lung cancer										
Incidence	RR	N/A	1.10 (0.74, 1.63)	N/A	N/A	1.10 (0.74, 1.63)	Not possible	1	1	1
Mortality	RR	N/A	1.09 (0.98, 1.22)	N/A	N/A	1.09 (0.98, 1.22)	Not possible	1	N/A	N/A
Breast cancer										
Incidence	RR	No evidence	1.05 (0.71, 1.57)	N/A	N/A	0.88 (0.34, 2.28)	3	1	N/A	N/A
Mortality	RR	N/A	1.03 (0.06, 17.26)	N/A	N/A	1.03 (0.06, 17.26)	Not possible	1	N/A	N/A
Endometrial cancer	RR	N/A	0.65 (0.17, 2.44)	N/A	N/A	0.43 (0.07, 2.74)	Not possible	1	Not possible	1
Ovarian cancer	RR	N/A	1.21 (0.15, 9.77)	N/A	N/A	1.21 (0.15, 9.77)	Not possible	1	Not possible	1
**Symptoms, signs or clinical findings of blood, blood-forming organs, or the immune system**										
C-reactive protein[f]	MD (% change)	Likely	42.28 (18.14, 66.41)	N/A	N/A	17.81 (-18.60, 54.22)	Not possible	2	N/A	N/A
C-reactive protein[g]	MD (mg/L)	N/A	0.35 (-1.27, 1.96)	N/A	N/A	0.35 (-1.27, 1.96)	Not possible	1	N/A	N/A
PAI-1 antigen	MD (% change)	No evidence	-32.19 (-44.38, -20.01)	N/A	N/A	-19.77 (-53.07, 13.54)	Not possible	9	N/A	N/A
Fibrinogen	MD (% change)	No evidence	-5.43 (-7.36, -3.49)	N/A	N/A	-4.52 (-6.05, -3.00)	Not possible	Not possible	N/A	N/A
E-selectin	MD (% change)	N/A	-17.63 (-26.74, -8.51)	N/A	N/A	-9.73 (-14.15, -5.32)	Not possible	Not possible	N/A	N/A
**Symptoms, signs or clinical findings of endocrine, nutritional or metabolic diseases**										
Insulin resistance	MD (% change)	No evidence	-14.17 (-21.94, -6.40)	N/A	N/A	-7.33 (-10.85, -3.82)	Not possible	Not possible	N/A	N/A
Total cholesterol[g]	MD (mg/dL)	No evidence	-13.20 (-19.69, -6.72)	N/A	N/A	-4.39 (-16.70, 7.91)	Not possible	3	N/A	N/A
Total cholesterol[h]	MD (mg/dL)	N/A	8.59 (-4.45, 21.64)	N/A	N/A	8.59 (-4.45, 21.64)	Not possible	1	N/A	N/A
Total cholesterol[i]	MD (mmol/L)	N/A	-0.39 (-0.78, -0.01)	N/A	N/A	-0.20 (-1.01, 0.60)	Not possible	1	N/A	N/A
HDL cholesterol[g]	MD (mg/dL)	No evidence	1.41 (-3.56, 6.37)	N/A	N/A	-1.15 (-4.83, 2.53)	2	1	N/A	N/A
HDL cholesterol[h]	MD (mg/dL)	N/A	11.68 (-6.43, 29.79)	N/A	N/A	8.40 (-7.90, 24.70)	Not possible	1	N/A	N/A
HDL cholesterol[i]	MD (mmol/L)	N/A	-0.05 (-0.17, 0.07)	N/A	N/A	0.00 (-0.04, 0.03)	Not possible	1	N/A	N/A
LDL cholesterol[g]	MD (mg/dL)	No evidence	-13.16 (-18.66, -7.67)	N/A	N/A	-5.63 (-16.91, 5.65)	Not possible	3	N/A	N/A
LDL cholesterol[h]	MD (mg/dL)	N/A	-7.10 (-34.28, 20.07)	N/A	N/A	-7.10 (-34.28, 20.07)	Not possible	1	N/A	N/A
LDL cholesterol[i]	MD (mmol/L)	N/A	-0.22 (-0.42, -0.01)	N/A	N/A	-0.22 (-0.42, -0.01)	Not possible	Not possible	N/A	N/A
LDL/HDL ratio	MD (% change)	No evidence	-16.25 (-18.83, -13.68)	-15.43 (-18.80, -12.06)	4.3×10^{-1}	-7.52 (-10.93, -4.10)	Not possible	Not possible	N/A	N/A
Lipoprotein (a)	MD (% change)	No evidence	-21.20 (-28.78, -13.62)	-16.60 (-25.54, -7.65)	6.2×10^{-1}	-2.68 (-10.29, 4.94)	Not possible	>200	N/A	N/A
Triglyceride[g]	MD (mg/dL)	No evidence	-4.49 (-18.84, 9.87)	N/A	N/A	5.01 (-3.80, 13.82)	2	1	N/A	N/A
Triglyceride[h]	MD (mg/dL)	N/A	21.08 (-8.20, 50.36)	N/A	N/A	21.08 (-8.20, 50.36)	Not possible	1	N/A	N/A
Triglyceride[i]	MD (mmol/L)	N/A	-0.11 (-0.65, 0.43)	N/A	N/A	-0.11 (-0.65, 0.43)	Not possible	1	N/A	N/A
Triglyceride[f]	MD (% change)	No evidence	2.39 (-0.69, 5.48)	2.37 (-1.35, 6.09)	8.6×10^{-1}	0.00 (-3.41, 3.41)	16	1	N/A	N/A
Body mass index[j]	MD (kg/m^2)	Likely	-0.11 (-0.48, 0.26)	N/A	N/A	-0.11 (-0.48, 0.26)	Not possible	1	N/A	N/A
Body mass index[g]	MD (kg/m^2)	N/A	0.17 (-7.00, 7.35)	N/A	N/A	0.17 (-7.00, 7.35)	Not possible	1	N/A	N/A
Body mass index[i]	MD (kg/m^2)	N/A	-1.05 (-6.67, 4.57)	N/A	N/A	-1.05 (-6.67, 4.57)	Not possible	1	N/A	N/A
Body weight	MD (kg)	No evidence	-0.03 (-0.67, 0.60)	N/A	N/A	-0.01 (-0.66, 0.65)	1	1	N/A	N/A
Abdominal fat	MD (% change)	N/A	-6.89 (-25.53, 11.75)	N/A	N/A	-1.11 (-25.56, 23.35)	Not possible	1	N/A	N/A
Waist circumference	MD (% change)	N/A	-0.82 (-4.04, 2.39)	N/A	N/A	-0.87 (-1.89, 0.14)	Not possible	1	N/A	N/A
Waist/hip ratio	MD	N/A	0.00 (0.00, 0.00)	N/A	N/A	0.00 (0.00, 0.00)	1	1	N/A	N/A

BMD of lumbar spine[k]	MD (g/cm^2/year)	N/A	1.26 (-11.48, 14.00)	N/A	N/A	1.26 (-11.48, 14.00)	Not possible	1	N/A	N/A
BMD of proximal femur[k]	MD (g/cm^2/year)	N/A	2.24 (0.60, 3.89)	N/A	N/A	1.95 (-1.83, 5.72)	Not possible	4	N/A	N/A
**Mental or behavioural disorders**										
Dementia (probable)	RR	N/A	1.74 (0.30, 9.97)	N/A	N/A	1.49 (0.84, 2.66)	Not possible	1	Not possible	1
**Mental or behavioural symptoms, signs or clinical findings**										
Depressive symptom	SMD	N/A	-0.13 (-0.64, 0.38)	N/A	N/A	0.12 (-0.19, 0.43)	2	1	N/A	N/A
**Diseases of the nervous system**										
Cerebrovascular disease	RR	No evidence	1.25 (1.04, 1.50)	N/A	N/A	1.12 (0.93, 1.36)	Not possible	1	Not possible	1
Stroke	RR	No evidence	1.17 (1.05, 1.29)	N/A	N/A	1.17 (1.05, 1.29)	Not possible	Not possible	Not possible	1
Fatal stroke	RR	N/A	1.08 (0.63, 1.83)	N/A	N/A	1.08 (0.63, 1.83)	Not possible	1	N/A	N/A
Non-fatal stroke	RR	No evidence	1.35 (1.08, 1.69)	N/A	N/A	1.21 (0.75, 1.94)	Not possible	>200	Not possible	1
Transient ischaemic attack	RR	N/A	0.88 (0.30, 2.60)	N/A	N/A	0.88 (0.30, 2.60)	Not possible	1	Not possible	1
Alzheimer disease	RR	N/A	1.61 (0.58, 4.46)	N/A	N/A	1.61 (0.58, 4.46)	Not possible	1	Not possible	1
**Diseases of the circulatory system**										
Coronary heart disease										
Incidence	RR	No evidence	1.02 (0.82, 1.26)	N/A	N/A	1.02 (0.82, 1.26)	Not possible	1	N/A	N/A
Mortality	RR	N/A	0.96 (0.46, 2.00)	N/A	N/A	0.96 (0.46, 2.00)	Not possible	1	N/A	N/A
MI	RR	No evidence	1.06 (0.65, 1.75)	N/A	N/A	1.06 (0.65, 1.75)	Not possible	1	N/A	N/A
Fatal MI	RR	N/A	0.52 (0.12, 2.14)	N/A	N/A	0.52 (0.12, 2.14)	Not possible	1	Not possible	1
Non-fatal MI	RR	N/A	1.06 (0.34, 3.30)	N/A	N/A	1.06 (0.34, 3.30)	Not possible	1	N/A	N/A
Angina pectoris										
Any angina	RR	N/A	0.90 (0.26, 3.07)	N/A	N/A	0.90 (0.26, 3.07)	Not possible	1	1	1
Unstable angina	RR	N/A	6.98 (0.36, 135.01)	N/A	N/A	6.98 (0.36, 135.01)	Not possible	1	Not possible	1
Venous thromboembolism	RR	No evidence	1.60 (0.99, 2.58)	N/A	N/A	1.10 (0.47, 2.55)	Not possible	6	Not possible	1
Deep vein thrombosis	RR	Likely	1.39 (0.68, 2.84)	N/A	N/A	1.09 (0.22, 5.43)	Not possible	1	Not possible	1
Pulmonary embolism	RR	N/A	1.26 (0.81, 1.94)	N/A	N/A	1.18 (0.47, 2.96)	Not possible	1	Not possible	1
**Symptoms, signs or clinical findings of the circulatory system**										
Cardiac death	RR	N/A	0.96 (0.67, 1.39)	N/A	N/A	0.96 (0.67, 1.39)	Not possible	1	N/A	N/A
Coronary revascularization	RR	N/A	1.03 (0.90, 1.18)	N/A	N/A	1.03 (0.90, 1.18)	Not possible	1	N/A	N/A
Systolic blood pressure	MD (mmHg)	N/A	0.84 (-5.67, 7.36)	N/A	N/A	-2.24 (-11.09, 6.62)	1	1	N/A	N/A
Diastolic blood pressure	MD (mmHg)	N/A	-0.31 (-2.94, 2.33)	N/A	N/A	-0.31 (-2.94, 2.33)	Not possible	1	N/A	N/A
**Diseases of the digestive system**										
Gallbladder disease[l]	RR	N/A	1.63 (1.31, 2.04)	N/A	N/A	1.51 (1.20, 1.90)	Not possible	Not possible	Not possible	Not possible
**Diseases of the genitourinary system**										
Endometrial hyperplasia	RR	No evidence	2.70 (1.15, 6.38)	N/A	N/A	1.25 (0.70, 2.21)	Not possible	4	Not possible	2
Irregular vaginal bleeding	RR	N/A	1.78 (0.77, 4.10)	N/A	N/A	1.07 (0.31, 3.72)	Not possible	1	Not possible	1
**Injury, poisoning or certain other consequences of external causes**										
All fracture	RR	Likely	0.72 (0.62, 0.84)	0.74 (0.62, 0.89)	5.8×10^{-1}	0.78 (0.58, 1.05)	Not possible	Not possible	Not possible	12
Vertebral fracture	RR	No evidence	0.69 (0.50, 0.94)	N/A	N/A	0.74 (0.51, 1.07)	Not possible	1	Not possible	1
Nonvertebral fracture	RR	No evidence	0.76 (0.62, 0.94)	N/A	N/A	0.85 (0.58, 1.26)	Not possible	1	Not possible	1
Hip fracture	RR	N/A	0.85 (0.57, 1.29)	N/A	N/A	0.91 (0.62, 1.35)	Not possible	1	12	1
**Functioning assessment**										
Sleep quality	SMD	N/A	-0.13 (-0.42, 0.16)	N/A	N/A	-0.06 (-0.57, 0.45)	Not possible	1	N/A	N/A
Sexual function	SMD	Likely	-0.21 (-0.37, -0.05)	N/A	N/A	-0.09 (-0.28, 0.11)	Not possible	2	1	1
Skeletal muscle strength	SMD	N/A	-0.46 (-1.13, 0.22)	N/A	N/A	-0.06 (-1.08, 0.95)	Not possible	1	10	1
**Others, not elsewhere classified**										
All-cause mortality	RR	No evidence	0.99 (0.83, 1.18)	N/A	N/A	0.99 (0.83, 1.18)	Not possible	1	N/A	N/A
Cardiovascular disease										
Incidence	RR	No evidence	1.29 (0.99, 1.68)	N/A	N/A	1.12 (0.96, 1.31)	Not possible	1	Not possible	1

Mortality	RR	N/A	0.96 (0.59, 1.57)	N/A	N/A	1.00 (0.74, 1.34)	Not possible	1	N/A	N/A

Abbreviations: BMD, bone mineral density; HDL, high-density lipoprotein; LDL, low-density lipoprotein; LRT, likelihood-ratio test; MD, mean difference; MI, myocardial infarction; N/A, not available or not applicable; PAI-1, plasminogen activator inhibitor-1; RR, risk ratio; SMD, standardized mean difference.

[a] Incidence unless otherwise indicated.

[b] Egger's regression test is used to examine whether smaller studies tend to show more pronounced effects than larger studies. It is applied only in meta-analyses of ≥ 10 studies. More information about how to interpret small-study effects can be found in S3 Text (section 4).

[c] Robust random-effects meta-analysis of all studies.

[d] This model provides: (1) a summary effect estimate corrected for suspected publication bias; (2) a likelihood-ratio test for the presence of publication bias. This model is applied only in meta-analyses of ≥ 30 studies. More information can be found in S3 Text (section 5).

[e] This model provides: (1) a summary effect estimate corrected for worst-case publication bias; (2) severity of publication bias (η) required to attenuate $\hat{\mu}$ (pooled point estimate) or $\hat{\mu}^{ci}$ (the limit of 95% confidence interval) to the null or to a non-null value q (for MD, null = 0; for SMD, null = 0, q = -0.2 or 0.2; for RR, null = 1.0, q = 0.9 or 1.1; q is the value in the same direction of the pooled estimate). A large η would indicate that the meta-analysis result is relatively robust to publication bias, whereas a small η would indicate that the meta-analysis result is relatively sensitive to publication bias. η is conservatively rounded down to the nearest integer; a η of ≥ 4 would represent implausibly severe or extreme publication bias; "Not possible" indicates that no value of η could sufficiently attenuate the statistic; "1" indicates that the statistic is already ≤ null or q. More information on this model can be found in S3 Text (section 5).

[f] In postmenopausal women without diabetes.

[g] In healthy postmenopausal women who used low-dose menopausal hormone therapy.

[h] In postmenopausal women with chronic kidney disease.

[i] In postmenopausal women with diabetes.

[j] In postmenopausal women who used menopausal hormone therapy for at least 3 months.

[k] In postmenopausal women with primary biliary cirrhosis.

[l] Gallbladder disease requiring surgery.

Table S. Assessment of Small-Study Effects and Publication Bias: Any Menopausal Hormone Therapy for Secondary Prevention of Multiple Outcomes in Included Systematic Reviews and Meta-Analyses of Randomized Controlled Trials

				Vevea & Hedges Selection Model[d]		Severity of Publication Bias (η) Required to "Explain Away" Results[e]				
Outcome[a]	Metric (unit)	Small-Study Effects[b]	Uncorrected[c]	Corrected	LRT *P*-Value	Worst-Case	$\eta(\hat{\mu}$, null)	$\eta(\hat{\mu}^{ci}$, null)	$\eta(\hat{\mu}, q)$	$\eta(\hat{\mu}^{ci}, q)$
**Neoplasms**										
Breast cancer recurrence	RR	N/A	1.51 (0.32, 7.12)	N/A	N/A	1.24 (0.51, 3.00)	Not possible	1	Not possible	1
Ovarian cancer OS	RR	N/A	0.87 (0.65, 1.18)	N/A	N/A	0.87 (0.65, 1.18)	Not possible	1	Not possible	1
**Symptoms, signs or clinical findings of endocrine, nutritional or metabolic diseases**										
Fasting glucose	MD (mmol/L)	N/A	-0.63 (-2.22, 0.97)	N/A	N/A	-0.63 (-2.22, 0.97)	Not possible	1	N/A	N/A
Hemoglobin A1c	MD (%)	N/A	-0.47 (-0.72, -0.23)	N/A	N/A	-0.43 (-0.99, 0.13)	Not possible	8	N/A	N/A
**Mental or behavioural symptoms, signs or clinical findings**										
Depressive symptom	SMD	N/A	0.16 (-1.62, 1.94)	N/A	N/A	-0.34 (-1.82, 1.13)	1	1	N/A	N/A
**Diseases of the nervous system**										
Cerebrovascular disease	RR	N/A	0.63 (0.00, ∞)	N/A	N/A	0.63 (0.00, ∞)	Not possible	1	Not possible	1
Stroke	RR	N/A	0.61 (0.00, ∞)	N/A	N/A	0.61 (0.00, ∞)	Not possible	1	Not possible	1
Fatal stroke	RR	N/A	0.82 (0.00, ∞)	N/A	N/A	0.82 (0.00, ∞)	Not possible	1	Not possible	1
Non-fatal stroke	RR	N/A	0.95 (0.67, 1.36)	N/A	N/A	0.95 (0.67, 1.36)	Not possible	1	N/A	N/A
Transient ischaemic attack	RR	N/A	1.16 (0.70, 1.94)	N/A	N/A	1.16 (0.70, 1.94)	Not possible	1	Not possible	1
**Diseases of the circulatory system**										
Coronary heart disease										
Incidence	RR	N/A	0.97 (0.76, 1.24)	N/A	N/A	0.97 (0.76, 1.24)	Not possible	1	N/A	N/A
Mortality	RR	N/A	1.02 (0.26, 4.05)	N/A	N/A	1.02 (0.26, 4.05)	Not possible	1	N/A	N/A
MI	RR	N/A	0.93 (0.76, 1.15)	N/A	N/A	0.93 (0.76, 1.15)	Not possible	1	N/A	N/A
Fatal MI	RR	N/A	0.84 (0.08, 9.49)	N/A	N/A	0.84 (0.08, 9.49)	Not possible	1	Not possible	1
Non-fatal MI	RR	N/A	0.96 (0.43, 2.15)	N/A	N/A	0.96 (0.43, 2.15)	Not possible	1	N/A	N/A
Angina pectoris										
Any angina	RR	N/A	0.91 (0.64, 1.29)	N/A	N/A	0.91 (0.64, 1.29)	Not possible	1	N/A	N/A
Unstable angina	RR	N/A	0.97 (0.51, 1.83)	N/A	N/A	0.97 (0.51, 1.83)	Not possible	1	N/A	N/A
Venous thromboembolism	RR	N/A	7.77 (1.00, 60.53)	N/A	N/A	7.77 (1.00, 60.53)	Not possible	1	Not possible	1
Deep vein thrombosis	RR	N/A	8.75 (0.48, 159.53)	N/A	N/A	8.75 (0.48, 159.53)	Not possible	1	Not possible	1
Pulmonary embolism	RR	N/A	2.92 (0.31, 27.35)	N/A	N/A	2.92 (0.31, 27.35)	Not possible	1	Not possible	1
**Symptoms, signs or clinical findings of the circulatory system**										
Cardiac death	RR	N/A	1.04 (0.33, 3.30)	N/A	N/A	1.04 (0.33, 3.30)	Not possible	1	N/A	N/A
Coronary revascularization	RR	N/A	0.98 (0.29, 3.33)	N/A	N/A	0.98 (0.29, 3.33)	Not possible	1	N/A	N/A
**Diseases of the genitourinary system**										
RUTI	RR	N/A	0.58 (0.11, 2.99)	N/A	N/A	1.06 (0.67, 1.65)	30	1	9	1
Vaginal atrophy	RR	N/A	0.31 (0.12, 0.81)	N/A	N/A	0.32 (0.00, ∞)	Not possible	1	Not possible	1
**Symptoms, signs or clinical findings of the genitourinary system**										
Vasomotor symptom	MD (freq/week)	N/A	-17.92 (-23.80, -12.04)	N/A	N/A	-11.40 (-22.99, 0.19)	Not possible	5	N/A	N/A
Vasomotor symptom severity	SMD	N/A	-1.36 (-1.95, -0.76)	N/A	N/A	N/A	N/A	N/A	N/A	N/A
Urinary incontinence	RR	Likely	0.82 (0.62, 1.09)	N/A	N/A	1.02 (0.81, 1.29)	Not possible	1	3	1
**Functioning assessment**										
Sleep quality	SMD	N/A	0.00 (-3.49, 3.50)	N/A	N/A	0.00 (-3.49, 3.50)	1	1	N/A	N/A
**Others, not elsewhere classified**										
Cardiovascular disease										
Incidence	RR	No evidence	1.08 (0.94, 1.25)	N/A	N/A	1.08 (0.94, 1.25)	Not possible	1	N/A	N/A
Mortality	RR	N/A	1.06 (0.62, 1.80)	N/A	N/A	1.06 (0.62, 1.80)	Not possible	1	N/A	N/A

Abbreviations: freq, frequency; LRT, likelihood-ratio test; MD, mean difference; MI, myocardial infarction; N/A, not available or not applicable; OS, overall survival; RR, risk ratio; RUTI, recurrent urinary tract infection; SMD, standardized mean difference.

[a] Incidence unless otherwise indicated.

[b] Egger's regression test is used to examine whether smaller studies tend to show more pronounced effects than larger studies. It is applied only in meta-analyses of ≥ 10 studies. More information about how to interpret small-study effects can be found in S3 Text (section 4).

[c] Robust random-effects meta-analysis of all studies.

[d] This model provides: (1) a summary effect estimate corrected for suspected publication bias; (2) a likelihood-ratio test for the presence of publication bias. This model is applied only in meta-analyses of ≥ 30 studies. More information can be found in S3 Text (section 5).

[e] This model provides: (1) a summary effect estimate corrected for worst-case publication bias; (2) severity of publication bias (η) required to attenuate $\hat{\mu}$ (pooled point estimate) or $\hat{\mu}^{ci}$ (the limit of 95% confidence interval) to the null or to a non-null value q (for MD, null = 0; for SMD, null = 0, q = -0.2 or 0.2; for RR, null = 1.0, q = 0.9 or 1.1; q is the value in the same direction of the pooled estimate). A large η would indicate that the meta-analysis result is relatively robust to publication bias, whereas a small η would indicate that the meta-analysis result is relatively sensitive to publication bias. η is conservatively rounded down to the nearest integer; a η of ≥ 4 would represent implausibly severe or extreme publication bias; “Not possible” indicates that no value of η could sufficiently attenuate the statistic; “1” indicates that the statistic is already ≤ null or q. More information on this model can be found in S3 Text (section 5).

Table T. Assessment of Small-Study Effects and Publication Bias: Any Menopausal Hormone Therapy for Primary Prevention of Multiple Outcomes in Included Systematic Reviews and Meta-Analyses of Observational Epidemiological Studies

Outcome[a]	Timing of MHT	Metric	Small-Study Effects[b]	Uncorrected[c]	Vevea & Hedges Selection Model[d]		Severity of Publication Bias (η) Required to "Explain Away" Results[e]				
					Corrected	LRT *P*-Value	Worst-Case	$\eta(\hat{\mu}, 1.0)$	$\eta(\hat{\mu}^{ci}, 1.0)$	$\eta(\hat{\mu}, q)$	$\eta(\hat{\mu}^{ci}, q)$
**Neoplasms**											
Cutaneous melanoma	Ever	RR	N/A	1.23 (0.90, 1.68)	N/A	N/A	1.03 (0.81, 1.32)	Not possible	1	3	1
Glioma	Ever	RR	Likely	0.87 (0.72, 1.04)	N/A	N/A	0.94 (0.80, 1.10)	Not possible	1	6	1
	Current	RR	N/A	0.82 (0.60, 1.13)	N/A	N/A	0.96 (0.78, 1.17)	Not possible	1	11	1
	Past	RR	N/A	0.94 (0.75, 1.19)	N/A	N/A	0.94 (0.75, 1.19)	Not possible	1	N/A	N/A
Meningioma	Ever	RR	No evidence	1.14 (0.98, 1.33)	N/A	N/A	0.96 (0.79, 1.17)	6	1	1	1
	Current	RR	No evidence	1.22 (1.02, 1.46)	N/A	N/A	0.88 (0.54, 1.42)	25	1	6	1
	Past	RR	No evidence	1.15 (0.98, 1.37)	N/A	N/A	1.09 (0.94, 1.27)	Not possible	1	5	1
Thyroid cancer	Ever	RR	No evidence	1.09 (0.88, 1.34)	N/A	N/A	1.01 (0.81, 1.27)	Not possible	1	N/A	N/A
	Current	RR	N/A	1.11 (0.76, 1.61)	N/A	N/A	1.02 (0.57, 1.81)	Not possible	1	1	1
	Past	RR	N/A	1.01 (0.51, 1.99)	N/A	N/A	1.01 (0.51, 1.99)	Not possible	1	N/A	N/A
Esophageal cancer	Ever	RR	N/A	0.70 (0.60, 0.81)	N/A	N/A	0.72 (0.58, 0.90)	Not possible	Not possible	Not possible	19
	Current	RR	N/A	0.68 (0.62, 0.74)	N/A	N/A	0.67 (0.00, ∞)	Not possible	Not possible	Not possible	Not possible
	Past	RR	N/A	0.72 (0.35, 1.50)	N/A	N/A	0.80 (0.00, ∞)	Not possible	1	Not possible	1
Gastric cancer	Ever	RR	N/A	0.78 (0.70, 0.86)	N/A	N/A	0.78 (0.70, 0.86)	Not possible	Not possible	Not possible	Not possible
	Current	RR	N/A	0.75 (0.27, 2.06)	N/A	N/A	0.75 (0.27, 2.06)	Not possible	1	Not possible	1
	Past	RR	N/A	0.84 (0.61, 1.16)	N/A	N/A	0.84 (0.61, 1.16)	Not possible	1	Not possible	1
Colorectal cancer	Ever	RR	No evidence	0.83 (0.77, 0.89)	N/A	N/A	0.92 (0.88, 0.97)	Not possible	Not possible	5	1
	Current	RR	No evidence	0.77 (0.71, 0.83)	N/A	N/A	0.89 (0.83, 0.95)	Not possible	Not possible	Not possible	4
	Past	RR	No evidence	0.88 (0.81, 0.97)	N/A	N/A	0.94 (0.88, 1.01)	Not possible	12	1	1
Pancreatic cancer	Ever	RR	No evidence	0.96 (0.82, 1.13)	N/A	N/A	1.00 (0.87, 1.15)	4	1	N/A	N/A
	Current	RR	N/A	0.86 (0.40, 1.87)	N/A	N/A	0.98 (0.06, 17.50)	Not possible	1	1	1
	Past	RR	N/A	0.89 (0.43, 1.83)	N/A	N/A	0.89 (0.43, 1.83)	Not possible	1	Not possible	1
Primary liver cancer	Ever	RR	N/A	0.65 (0.30, 1.39)	N/A	N/A	1.15 (0.98, 1.34)	9	1	4	1
	Current	RR	N/A	1.03 (0.03, 35.31)	N/A	N/A	1.03 (0.03, 35.31)	Not possible	1	N/A	N/A
	Past	RR	N/A	0.88 (0.00, 437.84)	N/A	N/A	1.41 (0.95, 2.09)	1	1	1	1
Lung cancer	Ever	RR	No evidence	0.95 (0.85, 1.05)	N/A	N/A	1.00 (0.92, 1.09)	5	1	N/A	N/A
	Current	RR	N/A	0.93 (0.78, 1.12)	N/A	N/A	1.00 (0.86, 1.17)	2	1	N/A	N/A
	Past	RR	N/A	0.93 (0.88, 0.99)	N/A	N/A	0.93 (0.88, 0.99)	Not possible	Not possible	N/A	N/A
Breast cancer											
Incidence	Ever	RR	No evidence	1.25 (1.19, 1.31)	1.26 (1.17, 1.34)	8.8×10^{-1}	1.07 (1.03, 1.11)	Not possible	Not possible	10	3
	Current	RR	No evidence	1.43 (1.33, 1.55)	1.48 (1.33, 1.65)	3.9×10^{-1}	1.10 (1.01, 1.19)	Not possible	43	47	5
	Past	RR	No evidence	1.04 (1.00, 1.08)	1.04 (1.00, 1.08)	9.4×10^{-1}	1.02 (0.98, 1.05)	Not possible	1	N/A	N/A
Mortality	Ever	RR	No evidence	0.95 (0.79, 1.13)	N/A	N/A	1.00 (0.79, 1.26)	Not possible	1	N/A	N/A
	Current	RR	N/A	1.01 (0.79, 1.29)	N/A	N/A	0.90 (0.71, 1.15)	1	1	N/A	N/A
	Past	RR	N/A	0.90 (0.76, 1.07)	N/A	N/A	0.96 (0.84, 1.09)	Not possible	1	1	1
Endometrial cancer											
Incidence	Ever	RR	Likely	2.10 (1.70, 2.59)	N/A	N/A	1.12 (0.47, 2.66)	Not possible	11	Not possible	7
	Current	RR	Likely	3.18 (1.84, 5.48)	N/A	N/A	1.05 (0.85, 1.30)	Not possible	17	Not possible	5
	Past	RR	No evidence	1.56 (1.14, 2.15)	N/A	N/A	1.04 (0.83, 1.29)	Not possible	5	63	1
Mortality	Ever	RR	N/A	2.60 (0.44, 15.50)	N/A	N/A	2.60 (0.44, 15.50)	Not possible	1	Not possible	1
Ovarian cancer	Ever	RR	No evidence	1.16 (1.06, 1.26)	1.13 (1.00, 1.27)	5.9×10^{-1}	1.07 (0.97, 1.19)	Not possible	3	3	1
	Current	RR	No evidence	1.24 (1.08, 1.44)	N/A	N/A	1.02 (0.79, 1.31)	Not possible	1	4	1
	Past	RR	No evidence	1.06 (0.95, 1.17)	N/A	N/A	1.01 (0.95, 1.08)	Not possible	1	N/A	N/A
**Diseases of the immune system**											

Systemic lupus erythematosus	Ever	RR	N/A	1.62 (0.67, 3.91)	N/A	N/A	1.34 (0.08, 23.30)	Not possible	1	Not possible	1
	Current	RR	N/A	1.53 (0.80, 2.94)	N/A	N/A	1.53 (0.80, 2.94)	Not possible	1	Not possible	1
	Past	RR	N/A	1.77 (0.29, 10.88)	N/A	N/A	1.14 (0.66, 1.94)	Not possible	1	Not possible	1
**Endocrine, nutritional or metabolic diseases**											
Diabetes mellitus	Ever	RR	N/A	0.81 (0.02, 28.81)	N/A	N/A	1.11 (0.67, 1.83)	5	1	2	1
	Current	RR	N/A	0.61 (0.00, 606.60)	N/A	N/A	1.10 (0.49, 2.48)	12	1	5	1
	Past	RR	N/A	0.90 (0.14, 5.96)	N/A	N/A	0.90 (0.14, 5.96)	Not possible	1	1	1
**Mental or behavioural disorders**											
Dementia	Ever	RR	N/A	0.94 (0.69, 1.28)	N/A	N/A	0.94 (0.69, 1.28)	Not possible	1	N/A	N/A
	Current	RR	N/A	1.23 (0.34, 4.37)	N/A	N/A	1.23 (0.34, 4.37)	Not possible	1	Not possible	1
	Past	RR	N/A	0.74 (0.35, 1.55)	N/A	N/A	0.74 (0.35, 1.55)	Not possible	1	Not possible	1
**Diseases of the nervous system**											
Parkinson disease	Ever	RR	N/A	1.04 (0.79, 1.36)	N/A	N/A	0.97 (0.66, 1.42)	Not possible	1	N/A	N/A
	Current	RR	N/A	1.23 (0.97, 1.57)	N/A	N/A	1.17 (0.90, 1.51)	Not possible	1	Not possible	1
	Past	RR	N/A	1.23 (0.81, 1.87)	N/A	N/A	1.23 (0.81, 1.87)	Not possible	1	Not possible	1
Alzheimer disease	Ever	RR	No evidence	0.78 (0.63, 0.95)	N/A	N/A	0.89 (0.71, 1.11)	Not possible	1	Not possible	1
	Current	RR	N/A	1.12 (0.68, 1.83)	N/A	N/A	1.12 (0.68, 1.83)	Not possible	1	Not possible	1
	Past	RR	N/A	0.64 (0.04, 9.40)	N/A	N/A	1.03 (0.00, ∞)	> 200	1	5	1
**Diseases of the visual system**											
Cataract	Ever	RR	N/A	0.87 (0.79, 0.97)	N/A	N/A	0.92 (0.83, 1.01)	Not possible	1	1	1
	Current	RR	N/A	0.88 (0.79, 0.98)	N/A	N/A	0.90 (0.80, 1.02)	Not possible	1	45	1
	Past	RR	N/A	0.99 (0.94, 1.05)	N/A	N/A	0.99 (0.94, 1.05)	Not possible	1	N/A	N/A
**Diseases of the circulatory system**											
Coronary heart disease											
Incidence	Ever	RR	No evidence	0.82 (0.69, 0.96)	N/A	N/A	0.96 (0.78, 1.18)	Not possible	1	6	1
	Current	RR	N/A	0.74 (0.62, 0.89)	N/A	N/A	0.86 (0.66, 1.13)	Not possible	4	Not possible	1
	Past	RR	N/A	0.87 (0.71, 1.05)	N/A	N/A	0.95 (0.66, 1.37)	Not possible	1	2	1
Mortality	Ever	RR	N/A	0.67 (0.25, 1.81)	N/A	N/A	1.14 (0.53, 2.44)	15	1	7	1
	Current	RR	N/A	0.60 (0.33, 1.08)	N/A	N/A	0.91 (0.27, 2.99)	Not possible	1	> 200	1
	Past	RR	N/A	0.81 (0.53, 1.26)	N/A	N/A	0.93 (0.60, 1.45)	Not possible	1	10	1
Venous thromboembolism	Ever	RR	N/A	1.99 (1.53, 2.58)	N/A	N/A	1.65 (0.99, 2.74)	Not possible	2	Not possible	1
	Current	RR	N/A	2.08 (1.40, 3.07)	N/A	N/A	1.19 (0.47, 2.98)	Not possible	33	Not possible	33
	Past	RR	N/A	1.17 (0.96, 1.43)	N/A	N/A	1.17 (0.96, 1.43)	Not possible	1	Not possible	1
Deep vein thrombosis	Current	RR	N/A	2.26 (1.14, 4.49)	N/A	N/A	N/A	N/A	N/A	N/A	N/A
Pulmonary embolism	Ever	RR	N/A	1.65 (0.99, 2.74)	N/A	N/A	1.65 (0.99, 2.74)	Not possible	1	Not possible	1
	Current	RR	N/A	2.05 (1.49, 2.83)	N/A	N/A	1.99 (0.54, 7.39)	Not possible	3	Not possible	2
	Past	RR	N/A	1.30 (0.70, 2.40)	N/A	N/A	1.30 (0.70, 2.40)	Not possible	1	Not possible	1
**Diseases of the respiratory system**											
Asthma	Ever	RR	N/A	1.41 (1.09, 1.81)	N/A	N/A	1.08 (0.71, 1.65)	Not possible	2	129	1
	Current	RR	N/A	1.48 (1.02, 2.13)	N/A	N/A	1.23 (0.70, 2.17)	Not possible	1	Not possible	1
	Past	RR	N/A	1.37 (1.08, 1.73)	N/A	N/A	1.12 (0.56, 2.23)	Not possible	12	Not possible	1
**Diseases of the digestive system**											
Cholelithiasis	Ever	RR	N/A	1.63 (1.41, 1.88)	N/A	N/A	1.22 (0.67, 2.23)	Not possible	14	Not possible	13
	Current	RR	N/A	1.89 (1.58, 2.27)	N/A	N/A	N/A	N/A	N/A	N/A	N/A
	Past	RR	N/A	1.40 (1.23, 1.59)	N/A	N/A	0.97 (0.64, 1.46)	> 200	7	133	1
**Others, not elsewhere classified**											
All-cause mortality	Ever	RR	N/A	0.89 (0.82, 0.97)	N/A	N/A	0.89 (0.78, 1.02)	Not possible	1	Not possible	1
	Current	RR	N/A	0.79 (0.66, 0.95)	N/A	N/A	0.89 (0.73, 1.10)	Not possible	2	Not possible	1
	Past	RR	N/A	0.92 (0.82, 1.03)	N/A	N/A	0.96 (0.76, 1.22)	Not possible	1	N/A	N/A

Cardiovascular disease											
Incidence	Ever	RR	N/A	1.11 (0.56, 2.22)	N/A	N/A	1.11 (0.56, 2.22)	Not possible	1	Not possible	1
	Current	RR	N/A	0.90 (0.11, 7.22)	N/A	N/A	1.07 (0.87, 1.32)	4	1	1	1
	Past	RR	N/A	1.11 (0.89, 1.39)	N/A	N/A	1.11 (0.89, 1.39)	Not possible	1	Not possible	1
Mortality	Ever	RR	N/A	0.65 (0.40, 1.06)	N/A	N/A	0.81 (0.02, 40.26)	Not possible	1	Not possible	1
	Current	RR	N/A	0.50 (0.18, 1.40)	N/A	N/A	0.72 (0.00, ∞)	Not possible	1	Not possible	1
	Past	RR	N/A	0.88 (0.59, 1.31)	N/A	N/A	0.88 (0.59, 1.31)	Not possible	1	Not possible	1

Abbreviations: LRT, likelihood-ratio test; MHT, menopausal hormone therapy; N/A, not available or not applicable; RR, risk ratio.

[a] Incidence unless otherwise indicated.

[b] Egger's regression test is used to examine whether smaller studies tend to show more pronounced effects than larger studies. It is applied only in meta-analyses of ≥ 10 studies. More information about how to interpret small-study effects can be found in S3 Text (section 4).

[c] Robust random-effects meta-analysis of all studies.

[d] This model provides: (1) a summary effect estimate corrected for suspected publication bias; (2) a likelihood-ratio test for the presence of publication bias. This model is applied only in meta-analyses of ≥ 30 studies. More information can be found in S3 Text (section 5).

[e] This model provides: (1) a summary effect estimate corrected for worst-case publication bias; (2) severity of publication bias (η) required to attenuate $\hat{\mu}$ (pooled point estimate) or $\hat{\mu}^{ci}$ (the limit of 95% confidence interval) to the null or to a non-null value q (for RR, null = 1.0, q = 0.9 or 1.1; q is the value in the same direction of the pooled estimate). A large η would indicate that the meta-analysis result is relatively robust to publication bias, whereas a small η would indicate that the meta-analysis result is relatively sensitive to publication bias. η is conservatively rounded down to the nearest integer; a η of ≥ 4 would represent implausibly severe or extreme publication bias; "Not possible" indicates that no value of η could sufficiently attenuate the statistic; "1" indicates that the statistic is already ≤ null or q. More information on this model can be found in S3 Text (section 5).

Table U. Sensitivity Analysis for Residual Confounding: Any Menopausal Hormone Therapy for Primary Prevention of Multiple Outcomes in Included Systematic Reviews and Meta-Analyses of Observational Epidemiological Studies

Outcome[a]	Timing of MHT	Metric	Summary Effect[b]	Severity of Residual Confounding Required to "Explain Away" Results[c]					
				$E(\hat{\mu}, 1.0)$	$E(\hat{\mu}^{ci}, 1.0)$	$E(\hat{\mu}, q)$	$E(\hat{\mu}^{ci}, q)$	$\hat{T}/\hat{G}(r, 1.0)$	$\hat{T}/\hat{G}(r, q)$
**Neoplasms**									
Cutaneous melanoma	Ever	RR	1.23 (0.90, 1.68)	1.77	1	1.49	1	N/A	N/A
Glioma	Ever	RR	0.87 (0.72, 1.04)	N/A	N/A	N/A	N/A	1.28/1.88	1.15/1.57
	Current	RR	0.82 (0.60, 1.13)	1.73	1	1.41	1	N/A	N/A
	Past	RR	0.94 (0.75, 1.19)	1.31	1	N/A	N/A	N/A	N/A
Meningioma	Ever	RR	1.14 (0.98, 1.33)	N/A	N/A	N/A	N/A	1.29/1.90	1.18/1.64
	Current	RR	1.22 (1.02, 1.46)	N/A	N/A	N/A	N/A	1.32/1.97	1.20/1.69
	Past	RR	1.15 (0.98, 1.37)	N/A	N/A	N/A	N/A	1.16/1.59	1.06/1.31
Thyroid cancer	Ever	RR	1.09 (0.88, 1.34)	N/A	N/A	N/A	N/A	1.14/1.54	1.04/1.24
	Current	RR	1.11 (0.76, 1.61)	1.45	1	1.08	1	N/A	N/A
	Past	RR	1.01 (0.51, 1.99)	1.09	1	N/A	N/A	N/A	N/A
Esophageal cancer	Ever	RR	0.70 (0.60, 0.81)	2.22	1.78	1.90	1.47	N/A	N/A
	Current	RR	0.68 (0.62, 0.74)	2.31	2.06	1.99	1.75	N/A	N/A
	Past	RR	0.72 (0.35, 1.50)	2.11	1	1.79	1	N/A	N/A
Gastric cancer	Ever	RR	0.78 (0.70, 0.86)	1.90	1.60	1.59	1.27	N/A	N/A
	Current	RR	0.75 (0.27, 2.06)	2.00	1	1.69	1	N/A	N/A
	Past	RR	0.84 (0.61, 1.16)	1.66	1	1.34	1	N/A	N/A
Colorectal cancer	Ever	RR	0.83 (0.77, 0.89)	N/A	N/A	N/A	N/A	1.48/2.32	1.33/1.99
	Current	RR	0.77 (0.71, 0.83)	N/A	N/A	N/A	N/A	1.47/2.30	1.32/1.97
	Past	RR	0.88 (0.81, 0.97)	N/A	N/A	N/A	N/A	1.29/1.90	1.16/1.59
Pancreatic cancer	Ever	RR	0.96 (0.82, 1.13)	N/A	N/A	N/A	N/A	1.15/1.57	1.03/1.21
	Current	RR	0.86 (0.40, 1.87)	1.59	1	1.26	1	N/A	N/A
	Past	RR	0.89 (0.43, 1.83)	1.49	1	1.10	1	N/A	N/A
Primary liver cancer	Ever	RR	0.65 (0.30, 1.39)	2.46	1	2.13	1	N/A	N/A
	Current	RR	1.03 (0.03, 35.31)	1.22	1	N/A	N/A	N/A	N/A
	Past	RR	0.88 (0.00, 437.84)	1.54	1	1.19	1	N/A	N/A
Lung cancer	Ever	RR	0.95 (0.85, 1.05)	N/A	N/A	N/A	N/A	1.19/1.67	1.07/1.34
	Current	RR	0.93 (0.78, 1.12)	1.34	1	N/A	N/A	N/A	N/A
	Past	RR	0.93 (0.88, 0.99)	1.36	1.11	N/A	N/A	N/A	N/A
Breast cancer									
Incidence	Ever	RR	1.25 (1.19, 1.31)	N/A	N/A	N/A	N/A	1.57/2.52	1.42/2.19
	Current	RR	1.43 (1.33, 1.55)	N/A	N/A	N/A	N/A	1.93/3.27	1.76/2.92

		Past	RR	1.04 (1.00, 1.08)	N/A	N/A	N/A	N/A	1.11/1.46	1.01/1.11
	Mortality	Ever	RR	0.95 (0.79, 1.13)	N/A	N/A	N/A	N/A	1.19/1.67	1.07/1.34
		Current	RR	1.01 (0.79, 1.29)	1.11	1	N/A	N/A	N/A	N/A
		Past	RR	0.90 (0.76, 1.07)	1.47	1	1.03	1	N/A	N/A
Endometrial cancer										
	Incidence	Ever	RR	2.10 (1.70, 2.59)	N/A	N/A	N/A	N/A	3.98/7.42	3.61/6.68
		Current	RR	3.18 (1.84, 5.48)	N/A	N/A	N/A	N/A	2.04/3.50	1.85/3.10
		Past	RR	1.56 (1.14, 2.15)	N/A	N/A	N/A	N/A	1.90/3.21	1.73/2.85
	Mortality	Ever	RR	2.60 (0.44, 15.50)	4.64	1	4.16	1	N/A	N/A
Ovarian cancer		Ever	RR	1.16 (1.06, 1.26)	N/A	N/A	N/A	N/A	1.35/2.04	1.23/1.76
		Current	RR	1.24 (1.08, 1.44)	N/A	N/A	N/A	N/A	1.52/2.41	1.38/2.10
		Past	RR	1.06 (0.95, 1.17)	N/A	N/A	N/A	N/A	1.13/1.51	1.03/1.21
**Diseases of the immune system**										
Systemic lupus erythematosus		Ever	RR	1.62 (0.67, 3.91)	2.62	1	2.30	1	N/A	N/A
		Current	RR	1.53 (0.80, 2.94)	2.44	1	2.14	1	N/A	N/A
		Past	RR	1.77 (0.29, 10.88)	2.94	1	2.60	1	N/A	N/A
**Endocrine, nutritional or metabolic diseases**										
Diabetes mellitus		Ever	RR	0.81 (0.02, 28.81)	1.78	1	1.47	1	N/A	N/A
		Current	RR	0.61 (0.00, 606.60)	2.66	1	2.31	1	N/A	N/A
		Past	RR	0.90 (0.14, 5.96)	1.46	1	1.02	1	N/A	N/A
**Mental or behavioural disorders**										
Dementia		Ever	RR	0.94 (0.69, 1.28)	1.32	1	N/A	N/A	N/A	N/A
		Current	RR	1.23 (0.34, 4.37)	1.76	1	1.48	1	N/A	N/A
		Past	RR	0.74 (0.35, 1.55)	2.04	1	1.73	1	N/A	N/A
**Diseases of the nervous system**										
Parkinson disease		Ever	RR	1.04 (0.79, 1.36)	1.24	1	N/A	N/A	N/A	N/A
		Current	RR	1.23 (0.97, 1.57)	1.77	1	1.49	1	N/A	N/A
		Past	RR	1.23 (0.81, 1.87)	1.76	1	1.48	1	N/A	N/A
Alzheimer disease		Ever	RR	0.78 (0.63, 0.95)	N/A	N/A	N/A	N/A	1.57/2.52	1.41/2.17
		Current	RR	1.12 (0.68, 1.83)	1.48	1	1.15	1	N/A	N/A
		Past	RR	0.64 (0.04, 9.40)	2.47	1	2.14	1	N/A	N/A
**Diseases of the visual system**										
Cataract		Ever	RR	0.87 (0.79, 0.97)	1.56	1.22	1.22	1	N/A	N/A
		Current	RR	0.88 (0.79, 0.98)	1.53	1.16	1.18	1	N/A	N/A
		Past	RR	0.99 (0.94, 1.05)	1.09	1	N/A	N/A	N/A	N/A
**Diseases of the circulatory system**										

Coronary heart disease									
Incidence	Ever	RR	0.82 (0.69, 0.96)	N/A	N/A	N/A	N/A	1.36/2.06	1.22/1.74
	Current	RR	0.74 (0.62, 0.89)	2.04	1.51	1.73	1.15	N/A	N/A
	Past	RR	0.87 (0.71, 1.05)	1.58	1	1.24	1	N/A	N/A
Mortality	Ever	RR	0.67 (0.25, 1.81)	2.37	1	2.04	1	N/A	N/A
	Current	RR	0.60 (0.33, 1.08)	2.73	1	2.37	1	N/A	N/A
	Past	RR	0.81 (0.53, 1.26)	1.76	1	1.45	1	N/A	N/A
Venous thromboembolism	Ever	RR	1.99 (1.53, 2.58)	3.39	2.43	3.02	2.13	N/A	N/A
	Current	RR	2.08 (1.40, 3.07)	3.57	2.15	3.18	1.86	N/A	N/A
	Past	RR	1.17 (0.96, 1.43)	1.62	1	1.33	1	N/A	N/A
Deep vein thrombosis	Current	RR	2.26 (1.14, 4.49)	3.95	1.54	3.53	1.24	N/A	N/A
Pulmonary embolism	Ever	RR	1.65 (0.99, 2.74)	2.69	1	2.37	1	N/A	N/A
	Current	RR	2.05 (1.49, 2.83)	3.52	2.34	3.13	2.04	N/A	N/A
	Past	RR	1.30 (0.70, 2.40)	1.92	1	1.65	1	N/A	N/A
**Diseases of the respiratory system**									
Asthma	Ever	RR	1.41 (1.09, 1.81)	2.16	1.42	1.88	1	N/A	N/A
	Current	RR	1.48 (1.02, 2.13)	2.31	1.17	2.02	1	N/A	N/A
	Past	RR	1.37 (1.08, 1.73)	2.08	1.39	1.80	1	N/A	N/A
**Diseases of the digestive system**									
Cholelithiasis	Ever	RR	1.63 (1.41, 1.88)	2.64	2.17	2.32	1.88	N/A	N/A
	Current	RR	1.89 (1.58, 2.27)	3.20	2.54	2.84	2.24	N/A	N/A
	Past	RR	1.40 (1.23, 1.59)	2.14	1.75	1.85	1.47	N/A	N/A
**Others, not elsewhere classified**									
All-cause mortality	Ever	RR	0.89 (0.82, 0.97)	1.50	1.22	1.12	1	N/A	N/A
	Current	RR	0.79 (0.66, 0.95)	1.84	1.30	1.54	1	N/A	N/A
	Past	RR	0.92 (0.82, 1.03)	1.40	1	N/A	N/A	N/A	N/A
Cardiovascular disease									
Incidence	Ever	RR	1.11 (0.56, 2.22)	1.46	1	1.12	1	N/A	N/A
	Current	RR	0.90 (0.11, 7.22)	1.47	1	1.06	1	N/A	N/A
	Past	RR	1.11 (0.89, 1.39)	1.46	1	1.10	1	N/A	N/A
Mortality	Ever	RR	0.65 (0.40, 1.06)	2.44	1	2.11	1	N/A	N/A
	Current	RR	0.50 (0.18, 1.40)	3.44	1	3.02	1	N/A	N/A
	Past	RR	0.88 (0.59, 1.31)	1.53	1	1.18	1	N/A	N/A

Abbreviations: MHT, menopausal hormone therapy; N/A, not available or not applicable; RR, risk ratio.

[a] Incidence unless otherwise indicated.

[b] Point estimate with 95% confidence interval of robust random-effects meta-analysis.

[c] E-value: the minimum strength of association, on RR scale, that residual confounding would need to have with both the exposure and outcome, conditional on the measured covariates, to attenuate $\hat{\mu}$ (pooled point estimate) or $\hat{\mu}^{ci}$ (the limit of 95% confidence interval) to the null or to a non-null value q; $\hat{T}/\hat{G}(r, q)$: the minimum bias factor on RR scale, $\hat{T}(r, q)$, or the minimum confounding association strength, $\hat{G}(r, q)$, in all studies that would be required to reduce to less than r the proportion of studies with true effect sizes exceeding the null or a non-null value q, conditional on the measured covariates. null = 1.0, q = 0.9 or 1.1 (q is the value in the same direction of the pooled estimate); r = 0.1 and 0.2 for meta-analyses of ≥ 16 studies and of 10–15 studies, respectively. A large E-value or $\hat{T}/\hat{G}(r, q)$ would indicate that the meta-analysis result is relatively robust to residual confounding, whereas a small E-value or $\hat{T}/\hat{G}(r, q)$ would indicate that the meta-analysis result is relatively sensitive to residual confounding. The E-value is reported in meta-analyses of < 10 studies, whereas $\hat{T}/\hat{G}(r, q)$ are reported in meta-analyses of ≥ 10 studies. More information on these metrics can be found in S3 Text (section 6).

Table V. Assessment of Small-Study Effects and Publication Bias: Any Menopausal Hormone Therapy for Secondary Prevention of Multiple Outcomes in Included Systematic Reviews and Meta-Analyses of Observational Epidemiological Studies

					Vevea & Hedges Selection Model[d]		Severity of Publication Bias (η) Required to “Explain Away” Results[e]				
**Outcome[a]**	**Timing of MHT**	**Metric**	**Small-Study Effects[b]**	**Uncorrected[c]**	**Corrected**	**LRT *P*-Value**	**Worst-Case**	$\eta(\hat{\mu}, 1.0)$	$\eta(\hat{\mu}^{ci}, 1.0)$	$\eta(\hat{\mu}, q)$	$\eta(\hat{\mu}^{ci}, q)$
**Neoplasms**											
Lung cancer overall survival	Ever	RR	N/A	1.06 (0.45, 2.51)	N/A	N/A	0.90 (0.12, 6.74)	1	1	N/A	N/A
	Current	RR	N/A	0.91 (0.76, 1.09)	N/A	N/A	0.91 (0.76, 1.09)	Not possible	1	N/A	N/A
Breast cancer											
Recurrence	Ever	RR	N/A	0.69 (0.29, 1.61)	N/A	N/A	N/A	N/A	N/A	N/A	N/A
Specific survival	Ever	RR	Likely	0.72 (0.59, 0.88)	N/A	N/A	0.82 (0.73, 0.93)	Not possible	Not possible	Not possible	2
	Current	RR	Likely	0.74 (0.62, 0.88)	N/A	N/A	0.80 (0.71, 0.91)	Not possible	Not possible	Not possible	> 200
	Past	RR	N/A	0.91 (0.70, 1.18)	N/A	N/A	0.94 (0.78, 1.13)	Not possible	1	N/A	N/A
Overall survival	Ever	RR	Likely	0.82 (0.75, 0.89)	N/A	N/A	0.93 (0.80, 1.08)	Not possible	5	13	1
	Current	RR	N/A	0.79 (0.73, 0.86)	N/A	N/A	0.86 (0.62, 1.18)	Not possible	10	Not possible	1
	Past	RR	N/A	0.92 (0.73, 1.16)	N/A	N/A	0.98 (0.58, 1.67)	Not possible	1	N/A	N/A
Ovarian cancer											
Recurrence	Ever	RR	N/A	0.87 (0.33, 2.29)	N/A	N/A	0.87 (0.33, 2.29)	Not possible	1	Not possible	1
Overall survival	Ever	RR	N/A	0.81 (0.71, 0.91)	N/A	N/A	0.81 (0.71, 0.91)	Not possible	Not possible	Not possible	1

Abbreviations: LRT, likelihood-ratio test; MHT, menopausal hormone therapy; N/A, not available or not applicable; RR, risk ratio.

[a] Incidence unless otherwise indicated.

[b] Egger’s regression test is used to examine whether smaller studies tend to show more pronounced effects than larger studies. It is applied only in meta-analyses of ≥ 10 studies. More information about how to interpret small-study effects can be found in S3 Text (section 4).

[c] Robust random-effects meta-analysis of all studies.

[d] This model provides: (1) a summary effect estimate corrected for suspected publication bias; (2) a likelihood-ratio test for the presence of publication bias. This model is applied only in meta-analyses of ≥ 30 studies. More information can be found in S3 Text (section 5).

[e] This model provides: (1) a summary effect estimate corrected for worst-case publication bias; (2) severity of publication bias (η) required to attenuate $\hat{\mu}$ (pooled point estimate) or $\hat{\mu}^{ci}$ (the limit of 95% confidence interval) to the null or to a non-null value q (for RR, null = 1.0, q = 0.9 or 1.1; q is the value in the same direction of the pooled estimate). A large η would indicate that the meta-analysis result is relatively robust to publication bias, whereas a small η would indicate that the meta-analysis result is relatively sensitive to publication bias. η is conservatively rounded down to the nearest integer; a η of ≥ 4 would represent implausibly severe or extreme publication bias; “Not possible” indicates that no value of η could sufficiently attenuate the statistic; “1” indicates that the statistic is already ≤ null or q. More information on this model can be found in S3 Text (section 5).

Table W. Sensitivity Analysis for Residual Confounding: Any Menopausal Hormone Therapy for Secondary Prevention of Multiple Outcomes in Included Systematic Reviews and Meta-Analyses of Observational Epidemiological Studies

Outcome[a]	Timing of MHT	Metric	Summary Effect[b]	Severity of Residual Confounding Required to "Explain Away" Results[c]					
				$E(\hat{\mu}, 1.0)$	$E(\hat{\mu}^{ci}, 1.0)$	$E(\hat{\mu}, q)$	$E(\hat{\mu}^{ci}, q)$	$\hat{T}/\hat{G}(r, 1.0)$	$\hat{T}/\hat{G}(r, q)$
**Neoplasms**									
Lung cancer overall survival	Ever	RR	1.06 (0.45, 2.51)	1.31	1	N/A	N/A	N/A	N/A
	Current	RR	0.91 (0.76, 1.09)	1.43	1	N/A	N/A	N/A	N/A
Breast cancer									
Recurrence	Ever	RR	0.69 (0.29, 1.61)	2.26	1	1.94	1	N/A	N/A
Specific survival	Ever	RR	0.72 (0.59, 0.88)	N/A	N/A	N/A	N/A	1.75/2.90	1.58/2.54
	Current	RR	0.74 (0.62, 0.88)	N/A	N/A	N/A	N/A	1.51/2.39	1.36/2.06
	Past	RR	0.91 (0.70, 1.18)	1.43	1	N/A	N/A	N/A	N/A
Overall survival	Ever	RR	0.82 (0.75, 0.89)	N/A	N/A	N/A	N/A	1.43/2.21	1.29/1.90
	Current	RR	0.79 (0.73, 0.86)	1.84	1.60	1.53	1.27	N/A	N/A
	Past	RR	0.92 (0.73, 1.16)	1.39	1	N/A	N/A	N/A	N/A
Ovarian cancer									
Recurrence	Ever	RR	0.87 (0.33, 2.29)	1.56	1	1.22	1	N/A	N/A
Overall survival	Ever	RR	0.81 (0.71, 0.91)	1.78	1.42	1.47	1	N/A	N/A

Abbreviations: MHT, menopausal hormone therapy; N/A, not available or not applicable; RR, risk ratio.

[a] Incidence unless otherwise indicated.

[b] Point estimate with 95% confidence interval of robust random-effects meta-analysis.

[c] E-value: the minimum strength of association, on RR scale, that residual confounding would need to have with both the exposure and outcome, conditional on the measured covariates, to attenuate $\hat{\mu}$ (pooled point estimate) or $\hat{\mu}^{ci}$ (the limit of 95% confidence interval) to the null or to a non-null value q; $\hat{T}/\hat{G}(r, q)$: the minimum bias factor on RR scale, $\hat{T}(r, q)$, or the minimum confounding association strength, $\hat{G}(r, q)$, in all studies that would be required to reduce to less than r the proportion of studies with true effect sizes exceeding the null or a non-null value q, conditional on the measured covariates. null = 1.0, q = 0.9 or 1.1 (q is the value in the same direction of the pooled estimate); r = 0.1 and 0.2 for meta-analyses of ≥ 16 studies and of 10–15 studies, respectively. A large E-value or $\hat{T}/\hat{G}(r, q)$ would indicate that the meta-analysis result is relatively robust to residual confounding, whereas a small E-value or $\hat{T}/\hat{G}(r, q)$ would indicate that the meta-analysis result is relatively sensitive to residual confounding. The E-value is reported in meta-analyses of < 10 studies, whereas $\hat{T}/\hat{G}(r, q)$ are reported in meta-analyses of ≥ 10 studies. More information on these metrics can be found in S3 Text (section 6).

Table X. Summary of Results for Outcomes with No Available Data for Meta-Analysis

Outcome[a]	Reference	Study Design	No. of Studies	Population	Intervention /Exposure[b]	Key Findings/Conclusions
**Neoplasms**						
Head and neck cancer	McCarthy 2017[37]	CO/CC	3	PPM	Any MHT	MHT was associated with a reduced risk of head and neck cancer, but the evidence was inconclusive
**Diseases of the musculoskeletal system or connective tissue**						
Osteoarthritis	de Klerk 2009[67]	RCT/CO /CC/CS	19	PPM	Any MHT	Most evidence pointed in the direction of no relation between osteoarthritis and exogenous hormone use; there was some evidence for a protective effect of unopposed estrogen use for hip osteoarthritis
**Symptoms, signs or clinical findings of blood, blood-forming organs, or the immune system**						
Immunological factors	Abdi 2016[9]	Unclear	13	PM	Unclear	MHT induced significant changes in immunological mediators, such as reduced levels of IL-2, IL-6, and IGF-1 as well as increased levels of IL-1 and IL-4
Protein C	Salpeter 2006[7]	RCT	Unclear	PM without diabetes	E/EP ≥2 mo[c]	There was no evidence that MHT has effect on protein C: MD (% change) -0.80, 95% CI: -4.20 to 2.60
Protein S	Salpeter 2006[7]	RCT	Unclear	PM without diabetes	E/EP ≥2 mo[c]	Oral MHT reduced protein S: MD (% change) -8.60, 95% CI: -13.10 to -4.10; no significant difference between opposed and unopposed estrogen
**Endocrine, nutritional or metabolic diseases**						
Diabetes mellitus	Salpeter 2006[7]	RCT	Unclear	PM	E/EP ≥2 mo[c]	MHT reduced the risk of diabetes mellitus: RR 0.70, 95% CI: 0.60 to 0.90
**Symptoms, signs or clinical findings of endocrine, nutritional or metabolic diseases**						
Fasting glucose	Salpeter 2006[7]	RCT	Unclear	PM with diabetes	E/EP ≥2 mo[c]	MHT reduced fasting glucose: MD (% change) -11.50, 95% CI: -18.00 to -5.10
Fasting glucose	Salpeter 2006[7]	RCT	Unclear	PM without diabetes	E/EP ≥2 mo[c]	MHT reduced fasting glucose: MD (% change) -2.50, 95% CI: -3.50 to -1.50

Fasting insulin	Salpeter 2006[7]	RCT	Unclear	PM with diabetes	E/EP ≥2 mo[c]	MHT reduced fasting insulin: MD (% change) -20.20, 95% CI: -36.30 to -4.20
Fasting insulin	Salpeter 2006[7]	RCT	Unclear	PM without diabetes	E/EP ≥2 mo[c]	MHT reduced fasting insulin: MD (% change) -9.30, 95% CI: -13.70 to -4.90
Insulin resistance	Salpeter 2006[7]	RCT	Unclear	PM with diabetes	E/EP ≥2 mo[c]	MHT reduced insulin resistance: MD (% change) -35.80, 95% CI: -51.70 to -19.80
HDL cholesterol	Salpeter 2006[7]	RCT	Unclear	PM without diabetes	E/EP ≥2 mo[c]	MHT increased HDL cholesterol: MD (% change) 5.10, 95% CI: 3.60 to 6.70
LDL cholesterol	Salpeter 2006[7]	RCT	Unclear	PM without diabetes	E/EP ≥2 mo[c]	MHT reduced LDL cholesterol: MD (% change) -11.00, 95% CI: -12.30 to -9.60
Lean body mass	Salpeter 2006[7]	RCT	Unclear	PM without diabetes	E/EP ≥2 mo[c]	MHT increased lean body mass: MD (% change) 3.30, 95% CI: 0.02 to 6.60
Bone mineral density of lumbar spine	Wells 2002[14]	RCT	21	PM	E/EP	MHT increased bone mineral density of lumbar spine at 2 yr: MD (% change) 6.76, 95% CI: 5.63 to 7.89; no significant difference between opposed and unopposed estrogen
Bone mineral density of forearm	Wells 2002[14]	RCT	14	PM	E/EP	MHT increased bone mineral density of forearm at 2 yr: MD (% change) 4.53, 95% CI: 3.68 to 5.36; no significant difference between opposed and unopposed estrogen
Bone mineral density of femoral neck	Wells 2002[14]	RCT	9	PM	E/EP	MHT increased bone mineral density of femoral neck at 2 yr: MD (% change) 4.12, 95% CI: 3.45 to 4.80; no significant difference between opposed and unopposed estrogen
**Symptoms, signs or clinical findings of the circulatory system**						
Mean blood pressure	Salpeter 2006[7]	RCT	Unclear	PM without diabetes	E/EP ≥2 mo[c]	MHT produced a small reduction in mean blood pressure: MD (% change) -1.70, 95% CI: -2.90 to -0.50
**Functioning assessment**						
Cognitive function	Lethaby 2008[30]	DBRCT	16	Healthy PM	E/EP ≥2 wks	There was good evidence that estrogen or combined estrogen and progestin therapy does not protect against a decline in overall cognitive functioning of older

						postmenopausal women with normal intellectual ability
Cognitive function	Marjoribanks 2017[3]	DBRCT	5	PPM without major health problems	E/EP ≥12 mo[d]	MHT was not indicated for prevention of deterioration of cognitive function in postmenopausal women
Cognitive function	Hogervorst 2009[31]	DBRCT	7	PM with dementia	E/EP ≥2 wks	Estrogen or estrogen combined with progestin for cognitive improvement or maintenance was not indicated for women with Alzheimer's disease

Abbreviations: CC, case-control study; CI, confidence interval; CO, cohort study; CS, cross-sectional study; DBRCT, double-blinded randomized controlled trial; E, estrogen alone; EP, estrogen plus progestin; HDL, high-density lipoprotein; IGF, insulin-like growth factor; IL, interleukin; LDL, low-density lipoprotein; MD, mean difference; MHT, menopausal hormone therapy; PM, postmenopausal women; PPM, peri-/post-menopausal women; RCT, randomized controlled trial; RR, risk ratio.

[a] Incidence unless otherwise indicated.

[b] "Any MHT": any type of menopausal hormone therapy, such as estrogen alone, estrogen plus progestin, tibolone, selective estrogen receptor modulators, or unspecified; the comparator group is placebo or no treatment, unless otherwise indicated.

[c] Conjugated equine estrogen, oral esterified estrogen or transdermal estrogen, alone or in combination with progestin for at least 2 months.

[d] Estrogen with/without progestin (oral/transdermal/subcutaneous/intranasal) for at least 12 months.

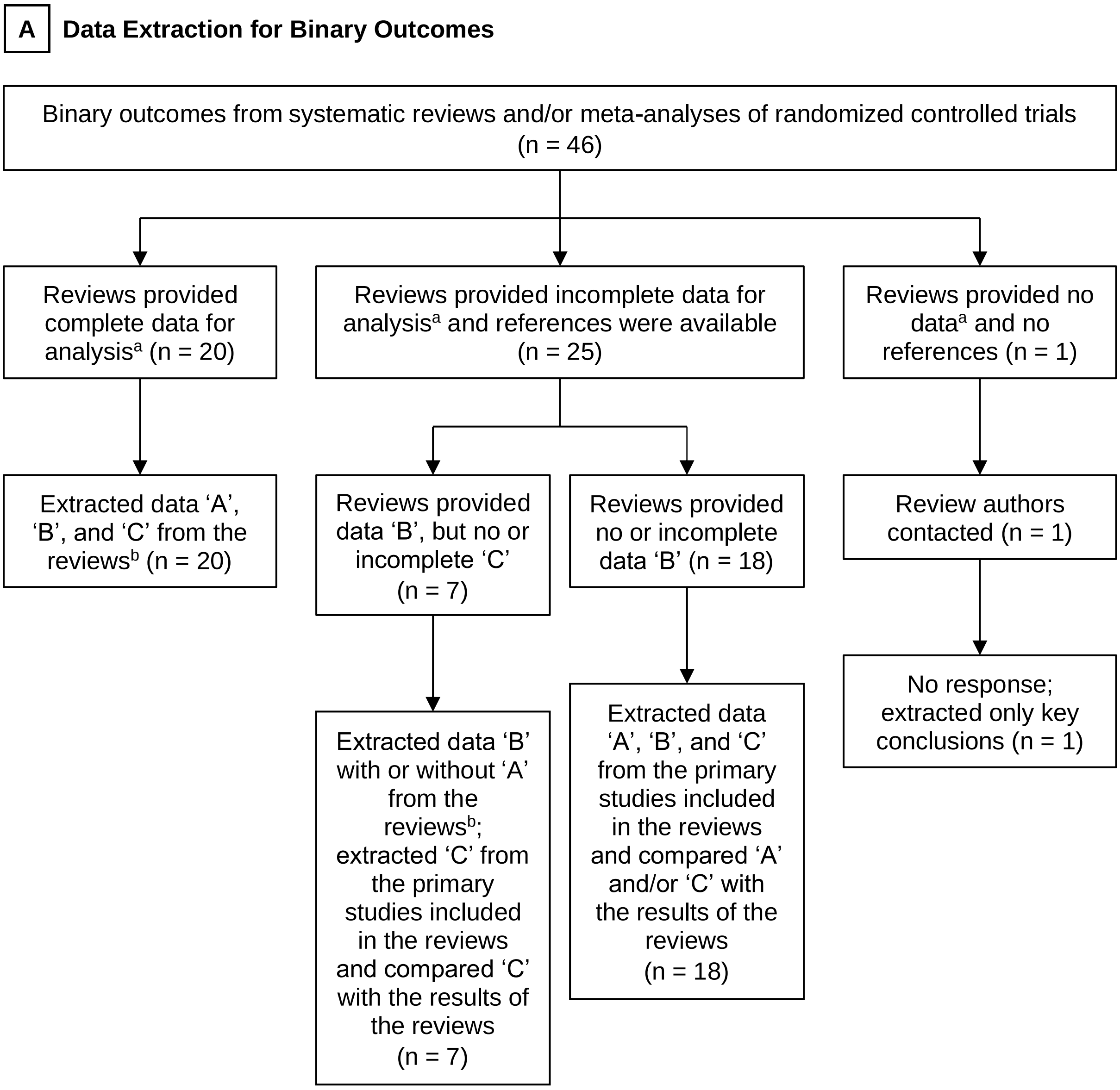

n, number of outcomes. The same outcome was counted more than once if investigated in different systematic reviews and/or meta-analyses with different population/intervention characteristics.

[a] 'Data for analysis' include study-specific effect estimates with 95% confidence intervals (denoted as 'A'), number of events and participants in intervention and control groups (as 'B'), and trial characteristics (as 'C').

[b] Data 'A', 'B', or 'C' were revised when we happened to find any errors in them.

Fig A. Pre-specified Protocol for Extracting Data from Included Systematic Reviews and/or Meta-Analyses of Randomized Controlled Trials on Menopausal Hormone Therapy and Multiple Outcomes

B Data Extraction for Continuous Outcomes

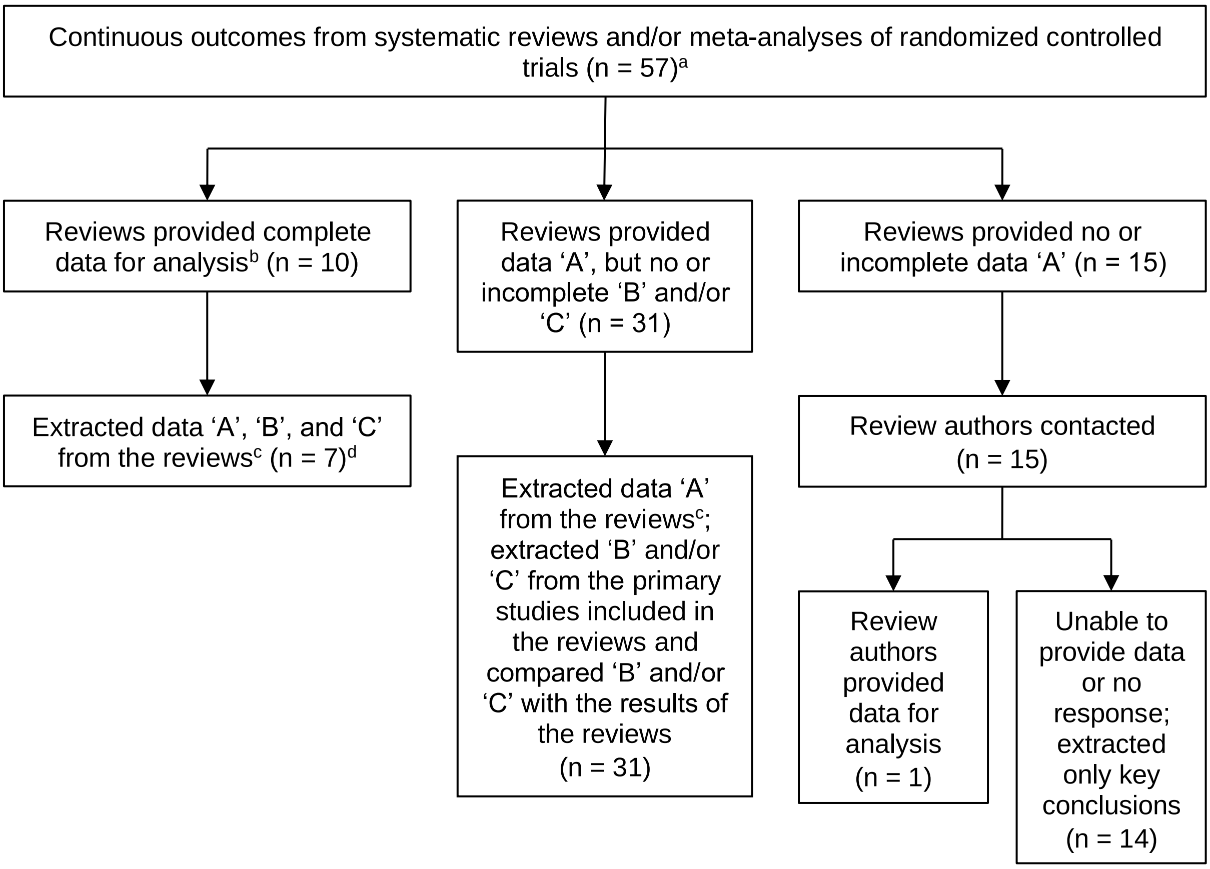

n, number of outcomes. The same outcome was counted more than once if investigated in different systematic reviews and/or meta-analyses with different population/intervention characteristics.

[a] For systematic reviews without meta-analysis (n = 1), only key findings or conclusions on each outcome were extracted from the reviews.

[b] 'Data for analysis' include study-specific effect estimates with 95% confidence intervals (denoted as 'A'), number of participants in intervention and control groups (as 'B'), and trial characteristics (as 'C').

[c] Data 'A', 'B', or 'C' were revised when we happened to find any errors in them.

[d] For outcome 'cognitive function', only key conclusions were extracted from the reviews (n = 3).

Fig A. Continued

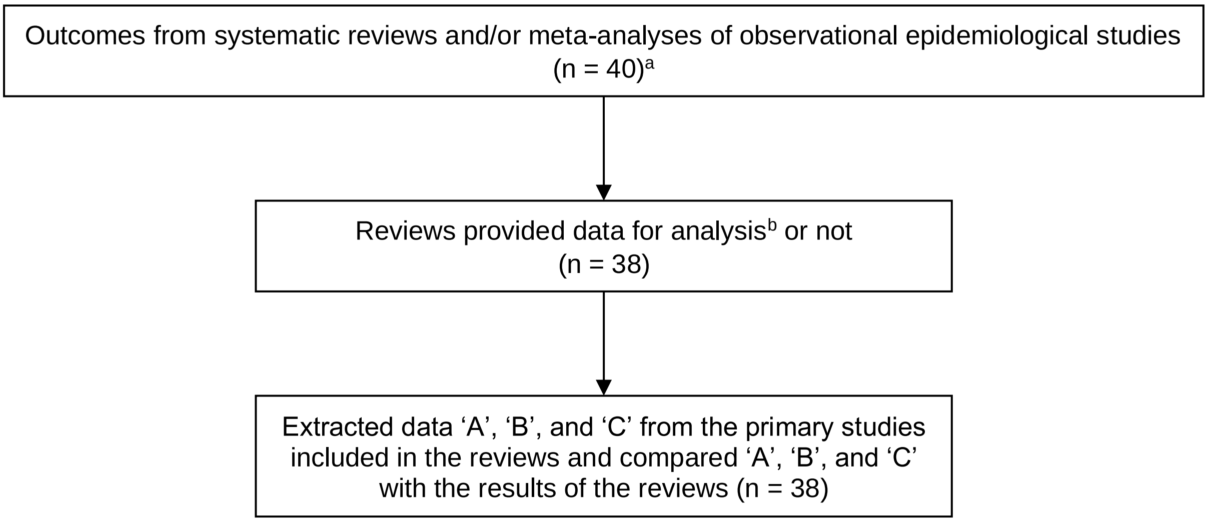

n, number of outcomes.

[a] For systematic reviews without meta-analysis (n = 2), only key findings or conclusions on each outcome were extracted from the reviews.

[b] ‘Data for analysis’ include study-specific effect estimates with 95% confidence intervals (denoted as ‘A’), number of cases and controls for case-control studies, or number of cases and population for cohort studies (as ‘B’), and study characteristics (as ‘C’).

Fig B. Pre-specified Protocol for Extracting Data from Included Systematic Reviews and/or Meta-Analyses of Observational Epidemiological Studies on Menopausal Hormone Therapy and Multiple Outcomes

Outcome	No. of Trials	No. of Participants: Menopausal Hormone Therapy	No. of Participants: Control	Unit	Mean Difference (95% CI)	Tau	95% Prediction Interval
**Symptoms, signs or clinical findings of blood, blood-forming organs, or the immune system**							
C-reactive protein	12	1,444	627	% change	42.28 (18.14, 66.41)	20.99	-1.98 to 77.31
PAI-1 antigen	13	1,323	655	% change	-32.19 (-44.38, -20.01)	6.52	-51.53 to -20.47
Fibrinogen	20	2,303	905	% change	-5.43 (-7.36, -3.49)	0	NA
E-selectin	7	985	352	% change	-17.63 (-26.74, -8.51)	0	NA
Protein S	NA	NA	NA	% change	-8.60 (-13.10, -4.10)	NA	NA
**Symptoms, signs or clinical findings of endocrine, nutritional or metabolic diseases**							
BMD of lumbar spine	21	NA	NA	% change	6.76 (5.63, 7.89)	NA	NA
BMD of forearm	14	NA	NA	% change	4.53 (3.68, 5.36)	NA	NA
BMD of femoral neck	9	NA	NA	% change	4.12 (3.45, 4.80)	NA	NA
BMD of proximal femur (EPT)	2	13	23	$g/cm^2/year$	2.24 (0.60, 3.89)	0	NA
Insulin resistance[a]	18	2,430	1,207	% change	-14.17 (-21.94, -6.40)	1.87	-20.15 to -11.12
Insulin resistance[b]	NA	NA	NA	% change	-35.80 (-51.70, -19.80)	NA	NA
Hemoglobin A1c (EPT)[b]	4	84	78	%	-0.47 (-0.72, -0.23)	0	NA
Fasting glucose[a]	NA	NA	NA	% change	-2.50 (-3.50, -1.50)	NA	NA
Fasting glucose[b]	NA	NA	NA	% change	-11.50 (-18.00, -5.10)	NA	NA
Fasting insulin[a]	NA	NA	NA	% change	-9.30 (-13.70, -4.90)	NA	NA
Fasting insulin[b]	NA	NA	NA	% change	-20.20 (-36.30, -4.20)	NA	NA
Total cholesterol[c]	11	599	565	mg/dL	-13.20 (-19.69, -6.72)	5.99	-25.38 to 3.38
Total cholesterol (EPT)[b]	5	125	132	mmol/L	-0.39 (-0.78, -0.01)	0.09	NA
HDL cholesterol	NA	NA	NA	% change	5.10 (3.60, 6.70)	NA	NA
LDL cholesterol[a]	NA	NA	NA	% change	-11.00 (-12.30, -9.60)	NA	NA
LDL cholesterol[c]	11	600	566	mg/dL	-13.16 (-18.66, -7.67)	2.88	-19.93 to -6.05
LDL cholesterol (EPT)[b]	5	124	128	mmol/L	-0.22 (-0.42, -0.01)	0	NA
LDL/HDL ratio	55	6,362	4,377	% change	-16.25 (-18.83, -13.68)	5.57	-30.46 to -4.67
Lipoprotein (a)	39	1,989	1,970	% change	-21.20 (-28.78, -13.62)	18.23	-77.87 to 2.47
Triglyceride	52	6,008	4,214	% change	2.39 (-0.69, 5.48)	4.87	-12.67 to 10.94
Waist circumference (EPT)	2	3,067	2,870	% change	-0.70 (-0.74, -0.66)	0	NA
Lean body mass	NA	NA	NA	% change	3.30 (0.02, 6.60)	NA	NA
**Symptoms, signs or clinical findings of the circulatory system**							
Mean blood pressure	NA	NA	NA	% change	-1.70 (-2.90, -0.50)	NA	NA

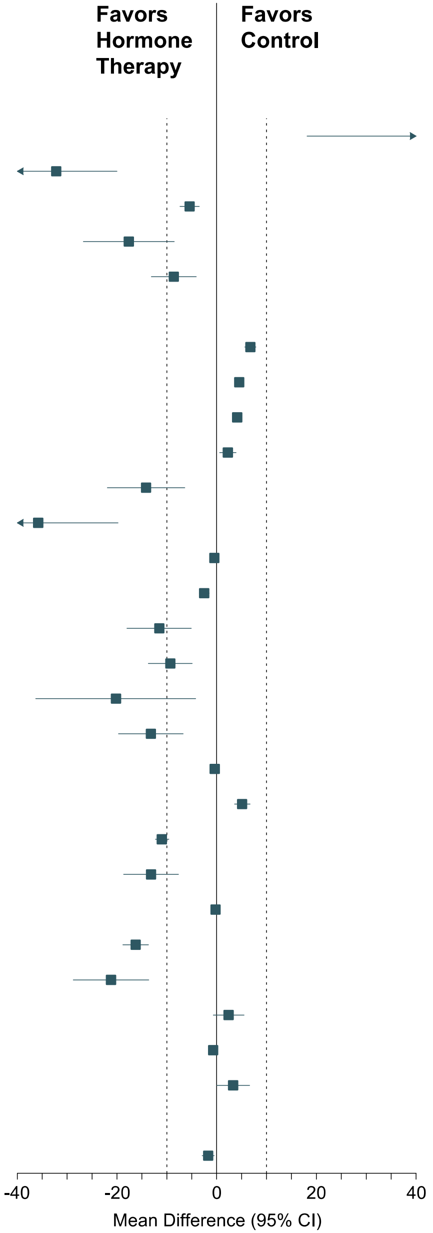

The average effects of any menopausal hormone therapy (estrogen-alone therapy or estrogen plus progestin therapy) in perimenopausal or postmenopausal women, unless otherwise stated. All estimates were from our own analysis apart from protein S, BMD of lumbar spine, BMD of forearm, BMD of femoral neck, insulin resistance in women with diabetes, fasting glucose, fasting insulin, HDL cholesterol, LDL cholesterol in women without diabetes, lean body mass and mean blood pressure. The center of each square represents the summary average effect for each outcome, and the horizontal lines represent the corresponding 95% confidence intervals. Abbreviations: BMD, bone mineral density; CI, confidence interval; EPT, estrogen plus progestin therapy; ET, estrogen-alone therapy; HDL, high-density lipoprotein; LDL, low-density lipoprotein; NA, not available or not applicable; PAI-1, plasminogen activator inhibitor-1.

[a] In women without diabetes.

[b] In women with diabetes.

[c] Low-dose menopausal hormone therapy.

Fig C. Consistent or Highly Suggestive Evidence from Meta-Analyses of Randomized Controlled Trials on Menopausal Hormone Therapy and Multiple Surrogate Outcomes

Outcome	No. of Trials	No. of Events/Total No.: Menopausal Hormone Therapy	No. of Events/Total No.: Control	Risk Ratio (95% CI)	Tau	Most Precise[a]
**Neoplasms**						
Breast cancer (ET)	7	247/6,537	302/6,472	0.83 (0.43, 1.61)	0	0.82 (0.70, 0.97)
Breast cancer (EPT)	7	651/13,548	502/12,580	1.24 (0.97, 1.57)	0	1.24 (1.10, 1.40)
Endometrial cancer (EPT)	6	76/11,159	106/10,026	0.65 (0.19, 2.26)	0	0.67 (0.50, 0.92)
**Diseases of the circulatory system**						
Pulmonary embolism (EPT)	3	193/12,082	134/11,674	2.02 (0.22, 18.91)	0.54	1.28 (1.02, 1.61)
**Mental or behavioural disorders**						
Dementia (EPT)	1	40/2,229	21/2,303	NA	NA	1.97 (1.16, 3.33)
**Diseases of the genitourinary system**						
Irregular vaginal bleeding (ET)	3	113/311	24/99	1.34 (0.07, 24.94)	0.32	1.73 (1.10, 2.71)
Recurrent urinary tract infection (ET)[b]	3	54/139	89/134	0.58 (0.11, 2.99)	0.51	0.64 (0.47, 0.86)
**Injury, poisoning or certain other consequences of external causes**						
Hip fracture (EPT)	4	249/12,152	287/11,746	0.83 (0.45, 1.54)	0	0.82 (0.69, 0.97)
**Functioning assessment**						
Sleep quality (EPT)[c]	3	NA/7,660	NA/7,328	0.86 (0.21, 3.50)	0.13	0.92 (0.89, 0.95)
Skeletal muscle strength (EPT)[c]	4	NA/104	NA/110	0.57 (0.28, 1.15)	0.36	0.65 (0.46, 0.93)

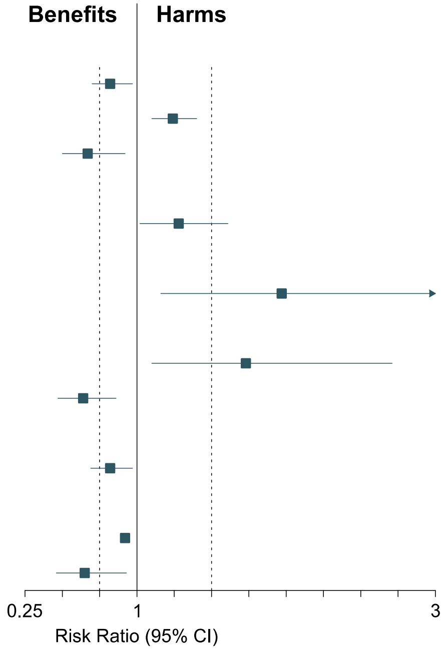

The effects of estrogen-alone therapy or estrogen plus progestin therapy in perimenopausal or postmenopausal women, unless otherwise stated. All estimates were from our own analysis. The center of each square represents the summary average effect for each outcome, and the horizontal lines represent the corresponding 95% confidence intervals. Abbreviations: CI, confidence interval; EPT, estrogen plus progestin therapy; ET, estrogen-alone therapy; NA, not available or not applicable.

[a] Risk ratio with 95% confidence interval of the study with the smallest standard error in each meta-analysis.

[b] In women who already have the outcomes of interest.

[c] The effect measures for continuous outcomes were converted into risk ratio scale for comparison. The results on its original scale can be found in Table H.

Fig D. Suggestive Evidence from Meta-Analyses of Randomized Controlled Trials on Menopausal Hormone Therapy and Incidence of Diseases and Other Health Outcomes

Outcome	No. of Studies (Cc/Co)	No. of Cases /Population	Risk Ratio (95% CI)	Tau	Most Precise[a]
**Neoplasms**					
Breast cancer recurrence	2 (0/2)	408/1,991	0.69 (0.29, 1.61)	0	0.72 (0.57, 0.91)
Lung cancer overall survival[b]	3 (0/3)	NA/1,972	1.06 (0.45, 2.51)	0.29	0.77 (0.65, 0.92)
Cutaneous melanoma (ET)	3 (3/0)	995/5,473	1.78 (0.74, 4.28)	0.15	2.08 (1.38, 3.14)
Endometrial cancer (EPT)[c]	8 (1/7)	9,103/1,735,287	1.30 (0.93, 1.82)	0.35	1.71 (1.57, 1.86)
**Diseases of the immune system**					
Systemic lupus erythematosus	3 (2/1)	204/71,061	1.62 (0.67, 3.91)	0	1.90 (1.16, 3.10)
**Diseases of the nervous system**					
Parkinson's disease	8 (5/3)	1,839/270,396	1.04 (0.79, 1.36)	0.21	1.24 (1.00, 1.53)
**Endocrine, nutritional or metabolic diseases**					
Diabetes mellitus	2 (0/2)	265/9,308	0.81 (0.02, 28.81)	0.34	0.63 (0.46, 0.87)
**Diseases of the circulatory system**					
Coronary heart disease mortality	3 (0/3)	512/49,929	0.67 (0.25, 1.81)	0.28	0.67 (0.53, 0.85)
**Others, not elsewhere classified**					
Cardiovascular disease incidence[c]	2 (0/2)	3,114/77,850	0.90 (0.11, 7.22)	0.22	0.77 (0.68, 0.87)
Cardiovascular disease mortality	4 (0/4)	604/16,896	0.65 (0.40, 1.06)	0.19	0.66 (0.48, 0.90)

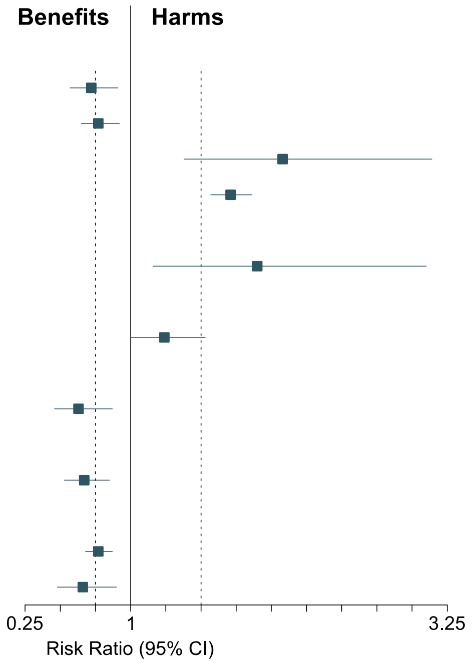

The average effects of any menopausal hormone therapy (estrogen-alone therapy or estrogen plus progestin therapy) in perimenopausal or postmenopausal women, unless otherwise stated. The estimates were for ever use of menopausal hormone therapy, unless otherwise stated. All estimates were from our own analysis. The center of each square represents the summary average effect for each outcome, and the horizontal lines represent the corresponding 95% confidence intervals. Abbreviations: Cc, case-control study; CI, confidence interval; Co, cohort study; EPT, estrogen plus progestin therapy; ET, estrogen-alone therapy; NA, not available.

[a] Risk ratio with 95% confidence interval of the study with the smallest standard error in each meta-analysis.

[b] Use of menopausal hormone therapy before diagnosis of cancer.

[c] Current use of menopausal hormone therapy.

Fig E. Suggestive Evidence from Meta-Analyses of Observational Epidemiological Studies on Menopausal Hormone Therapy and Incidence of Diseases and Other Health Outcomes

References

1. Zhu L, Jiang X, Sun Y, Shu W. Effect of hormone therapy on the risk of bone fractures: a systematic review and meta-analysis of randomized controlled trials. *Menopause* 2016;23(4):461-70.

2. Manson JE, Chlebowski RT, Stefanick ML, et al. Menopausal hormone therapy and health outcomes during the intervention and extended poststopping phases of the Women's Health Initiative randomized trials. *JAMA* 2013;310(13):1353-68.

3. Marjoribanks J, Farquhar C, Roberts H, Lethaby A, Lee J. Long-term hormone therapy for perimenopausal and postmenopausal women. *Cochrane Database Syst Rev* 2017;1(1):CD004143.

4. Benkhadra K, Mohammed K, Al Nofal A, et al. Menopausal Hormone Therapy and Mortality: A Systematic Review and Meta-Analysis. *J Clin Endocrinol Metab* 2015;100(11):4021-8.

5. Col NF, Kim JA, Chlebowski RT. Menopausal hormone therapy after breast cancer: a meta-analysis and critical appraisal of the evidence. *Breast Cancer Res* 2005;7(4):R535-40.

6. Li D, Ding CY, Qiu LH. Postoperative hormone replacement therapy for epithelial ovarian cancer patients: a systematic review and meta-analysis. *Gynecol Oncol* 2015;139(2):355-62.

7. Salpeter SR, Walsh JM, Ormiston TM, Greyber E, Buckley NS, Salpeter EE. Meta-analysis: effect of hormone-replacement therapy on components of the metabolic syndrome in postmenopausal women. *Diabetes Obes Metab* 2006;8(5):538-54.

8. Casanova G, Bossardi Ramos R, Ziegelmann P, Spritzer PM. Effects of low-dose versus placebo or conventional-dose postmenopausal hormone therapy on variables related to cardiovascular risk: a systematic review and meta-analyses of randomized clinical trials. *J Clin Endocrinol Metab* 2015;100(3):1028-37.

9. Abdi F, Mobedi H, Mosaffa N, Dolatian M, Ramezani Tehrani F. Effects of hormone replacement therapy on immunological factors in the postmenopausal period. *Climacteric* 2016;19(3):234-9.

10. Xu Y, Lin J, Wang S, Xiong J, Zhu Q. Combined estrogen replacement therapy on metabolic control in postmenopausal women with diabetes mellitus. *Kaohsiung J Med Sci* 2014;30(7):350-61.

11. Ramesh S, Mann MC, Holroyd-Leduc JM, et al. Hormone therapy and clinical and surrogate cardiovascular endpoints in women with chronic kidney disease: a systematic review and meta-analysis. *Menopause* 2016;23(9):1028-37.

12. Anagnostis P, Galanis P, Chatzistergiou V, et al. The effect of hormone replacement therapy and tibolone on lipoprotein (a) concentrations in postmenopausal women: A systematic review and meta-analysis. *Maturitas* 2017;99:27-36.

13. Kongnyuy EJ, Norman RJ, Flight IHK, Rees MC. Oestrogen and progestogen hormone replacement therapy for peri-menopausal and post-menopausal women: weight and body fat distribution. *Cochrane Database Syst Rev* 1999;1999(3):CD001018.

14. Wells G, Tugwell P, Shea B, et al. Meta-analyses of therapies for postmenopausal osteoporosis. V. Meta-analysis of the efficacy of hormone replacement therapy in treating and preventing osteoporosis in postmenopausal women. *Endocr Rev* 2002;23(4):529-39.

15. Rudic JS, Poropat G, Krstic MN, Bjelakovic G, Gluud C. Hormone replacement for osteoporosis in women with primary biliary cirrhosis. *Cochrane Database Syst Rev* 2011;2011(12):CD009146.

16. Whedon JM, KizhakkeVeettil A, Rugo NA, Kieffer KA. Bioidentical Estrogen for Menopausal Depressive Symptoms: A Systematic Review and Meta-Analysis. *J Womens Health* 2017;26(1):18-28.

17. Sare GM, Gray LJ, Bath PM. Association between hormone replacement therapy and subsequent arterial and venous vascular events: a meta-analysis. *Eur Heart J* 2008;29(16):2031-41.

18. Yang D, Li J, Yuan Z, Liu X. Effect of hormone replacement therapy on cardiovascular outcomes: a meta-analysis of randomized controlled trials. *PLoS One* 2013;8(5):e62329.

19. O'Brien J, Jackson JW, Grodstein F, Blacker D, Weuve J. Postmenopausal hormone therapy is not associated with risk of all-cause dementia and Alzheimer's disease. *Epidemiol Rev* 2014;36(1):83-103.

20. Boardman HM, Hartley L, Eisinga A, et al. Hormone therapy for preventing cardiovascular disease in post-menopausal women. *Cochrane Database Syst Rev* 2015;2015(3):CD002229.

21. Canonico M, Plu-Bureau G, Lowe GD, Scarabin PY. Hormone replacement therapy and risk of venous thromboembolism in postmenopausal women: systematic review and meta-analysis. *BMJ* 2008;336(7655):1227-31.

22. Perrotta C, Aznar M, Mejia R, Albert X, Ng CW. Oestrogens for preventing recurrent urinary tract infection in postmenopausal women. *Cochrane Database Syst Rev* 2008;2008(2):CD005131.

23. Furness S, Roberts H, Marjoribanks J, Lethaby A. Hormone therapy in postmenopausal women and risk of endometrial hyperplasia. *Cochrane Database Syst Rev* 2012;2012(8):CD000402.

24. Lethaby A, Ayeleke RO, Roberts H. Local oestrogen for vaginal atrophy in postmenopausal women. *Cochrane Database Syst Rev* 2016;2016(8):CD001500.

25. Lethaby AE, Farquhar C, Sarkis A, Roberts H, Jepson R, Barlow D. The association of oestrogen, oestrogen-progestogen and placebo with endometrial hyperplasia and irregular bleeding in the menopause. *Cochrane Database Syst Rev* 1999;1999(2):CD000402.

26. Maclennan AH, Broadbent JL, Lester S, Moore V. Oral oestrogen and combined oestrogen/progestogen therapy versus placebo for hot flushes. *Cochrane Database Syst Rev* 2004;2004(4):CD002978.

27. Cody JD, Jacobs ML, Richardson K, Moehrer B, Hextall A. Oestrogen therapy for urinary incontinence in post-menopausal women. *Cochrane Database Syst Rev* 2012;10(10):CD001405.

28. Torgerson DJ, Bell-Syer SE. Hormone replacement therapy and prevention of vertebral fractures: a meta-analysis of randomised trials. *BMC Musculoskelet Disord* 2001;2:7.

29. Torgerson DJ, Bell-Syer SE. Hormone replacement therapy and prevention of nonvertebral fractures: a meta-analysis of randomized trials. *JAMA* 2001;285(22):2891-7.

30. Lethaby A, Hogervorst E, Richards M, Yesufu A, Yaffe K. Hormone replacement therapy for cognitive function in postmenopausal women. *Cochrane Database Syst Rev* 2008;2008(1):CD003122.

31. Hogervorst E, Yaffe K, Richards M, Huppert FA. Hormone replacement therapy to maintain cognitive function in women with dementia. *Cochrane Database Syst Rev* 2009;2009(1):CD003799.

32. Cintron D, Lipford M, Larrea-Mantilla L, et al. Efficacy of menopausal hormone therapy on sleep quality: systematic review and meta-analysis. *Endocrine* 2017;55(3):702-11.

33. Nastri CO, Lara LA, Ferriani RA, Rosa ESAC, Figueiredo JB, Martins WP. Hormone therapy for sexual function in perimenopausal and postmenopausal women. *Cochrane Database Syst Rev* 2013;2013(6):CD009672.

34. Greising SM, Baltgalvis KA, Lowe DA, Warren GL. Hormone therapy and skeletal muscle strength: a meta-analysis. *J Gerontol A Biol Sci Med Sci* 2009;64(10):1071-81.

35. Salpeter SR, Walsh JM, Greyber E, Ormiston TM, Salpeter EE. Mortality associated with hormone replacement therapy in younger and older women: a meta-analysis. *J Gen Intern Med* 2004;19(7):791-804.

36. Gandini S, Iodice S, Koomen E, Di Pietro A, Sera F, Caini S. Hormonal and reproductive factors in relation to melanoma in women: current review and meta-analysis. *Eur J Cancer* 2011;47(17):2607-17.

37. McCarthy CE, Field JK, Marcus MW. Age at menopause and hormone replacement therapy as risk factors for head and neck and oesophageal cancer (Review). *Oncol Rep* 2017;38(4):1915-22.

38. Qi ZY, Shao C, Zhang X, Hui GZ, Wang Z. Exogenous and endogenous hormones in relation to glioma in women: a meta-analysis of 11 case-control studies. *PLoS One* 2013;8(7):e68695.

39. Benson VS, Kirichek O, Beral V, Green J. Menopausal hormone therapy and central nervous system tumor risk: large UK prospective study and meta-analysis. *Int J Cancer* 2015;136(10):2369-77.

40. Qi ZY, Shao C, Huang YL, Hui GZ, Zhou YX, Wang Z. Reproductive and exogenous hormone factors in relation to risk of meningioma in women: a meta-analysis. *PLoS One* 2013;8(12):e83261.

41. Cao Y, Wang Z, Gu J, et al. Reproductive Factors but Not Hormonal Factors Associated with Thyroid Cancer Risk: A Systematic Review and Meta-Analysis. *Biomed Res Int* 2015;2015:103515.

42. Zhu Y, Yue D, Yuan B, Zhu L, Lu M. Reproductive factors are associated with oesophageal cancer risk: results from a meta-analysis of observational studies. *Eur J Cancer Prev* 2017;26(1):1-9.

43. Camargo MC, Goto Y, Zabaleta J, Morgan DR, Correa P, Rabkin CS. Sex hormones, hormonal interventions, and gastric cancer risk: a meta-analysis. *Cancer Epidemiol Biomarkers Prev* 2012;21(1):20-38.

44. Green J, Czanner G, Reeves G, et al. Menopausal hormone therapy and risk of gastrointestinal cancer: nested case-control study within a prospective cohort, and meta-analysis. *Int J Cancer* 2012;130(10):2387-96.

45. Tang B, Lv J, Li Y, Yuan S, Wang Z, He S. Relationship between female hormonal and menstrual factors and pancreatic cancer: a meta-analysis of observational studies. *Medicine* 2015;94(7):e177.

46. Zhong GC, Liu Y, Chen N, et al. Reproductive factors, menopausal hormone therapies and primary liver cancer risk: a systematic review and dose-response meta-analysis of observational studies. *Hum Reprod Update* 2016;23(1):126-38.

47. Yao Y, Gu X, Zhu J, Yuan D, Song Y. Hormone replacement therapy in females can decrease the risk of lung cancer: a meta-analysis. *PLoS One* 2013;8(8):e71236.

48. Bae JM, Kim EH. Hormonal Replacement Therapy and the Risk of Lung Cancer in Women: An Adaptive Meta-analysis of Cohort Studies. *J Prev Med Public Health* 2015;48(6):280-6.

49. Li W, Lin X, Wang R, Wang F, Xie S, Tse LA. Hormone therapy and lung cancer mortality in women: Systematic review and meta-analysis. *Steroids* 2017;118:47-54.

50. Anothaisintawee T, Wiratkapun C, Lerdsitthichai P, et al. Risk factors of breast cancer: a systematic review and meta-analysis. *Asia Pac J Public Health* 2013;25(5):368-87.

51. Wang K, Li F, Chen L, Lai YM, Zhang X, Li HY. Change in risk of breast cancer after receiving hormone replacement therapy by considering effect-modifiers: a systematic review and dose-response meta-analysis of prospective studies. *Oncotarget* 2017;8(46):81109-24.

52. Yu X, Zhou S, Wang J, et al. Hormone replacement therapy and breast cancer survival: a systematic review and meta-analysis of observational studies. *Breast Cancer* 2017;24(5):643-57.
53. Grady D, Gebretsadik T, Kerlikowske K, Ernster V, Petitti D. Hormone replacement therapy and endometrial cancer risk: a meta-analysis. *Obstet Gynecol* 1995;85(2):304-13.
54. Sjogren LL, Morch LS, Lokkegaard E. Hormone replacement therapy and the risk of endometrial cancer: A systematic review. *Maturitas* 2016;91:25-35.
55. Shim SH, Lee SJ, Kim SN. Effects of hormone replacement therapy on the rate of recurrence in endometrial cancer survivors: a meta-analysis. *Eur J Cancer* 2014;50(9):1628-37.
56. Greiser CM, Greiser EM, Doren M. Menopausal hormone therapy and risk of ovarian cancer: systematic review and meta-analysis. *Hum Reprod Update* 2007;13(5):453-63.
57. Zhou B, Sun Q, Cong R, et al. Hormone replacement therapy and ovarian cancer risk: a meta-analysis. *Gynecol Oncol* 2008;108(3):641-51.
58. Shi LF, Wu Y, Li CY. Hormone therapy and risk of ovarian cancer in postmenopausal women: a systematic review and meta-analysis. *Menopause* 2016;23(4):417-24.
59. Rojas-Villarraga A, Torres-Gonzalez JV, Ruiz-Sternberg AM. Safety of hormonal replacement therapy and oral contraceptives in systemic lupus erythematosus: a systematic review and meta-analysis. *PLoS One* 2014;9(8):e104303.
60. Wang P, Li J, Qiu S, Wen H, Du J. Hormone replacement therapy and Parkinson's disease risk in women: a meta-analysis of 14 observational studies. *Neuropsychiatr Dis Treat* 2015;11:59-66.
61. LeBlanc ES, Janowsky J, Chan BK, Nelson HD. Hormone replacement therapy and cognition: systematic review and meta-analysis. *JAMA* 2001;285(11):1489-99.
62. Hogervorst E, Williams J, Budge M, Riedel W, Jolles J. The nature of the effect of female gonadal hormone replacement therapy on cognitive function in post-menopausal women: a meta-analysis. *Neuroscience* 2000;101(3):485-512.
63. Lai K, Cui J, Ni S, Zhang Y, He J, Yao K. The effects of postmenopausal hormone use on cataract: a meta-analysis. *PLoS One* 2013;8(10):e78647.
64. Humphrey LL, Chan BK, Sox HC. Postmenopausal hormone replacement therapy and the primary prevention of cardiovascular disease. *Ann Intern Med* 2002;137(4):273-84.
65. McCleary N, Nwaru BI, Nurmatov UB, Critchley H, Sheikh A. Endogenous and exogenous sex steroid hormones in asthma and allergy in females: A systematic review and meta-analysis. *J Allergy Clin Immunol* 2018;141(4):1510-3.e8.
66. Wang S, Wang Y, Xu J, Chen Y. Is the oral contraceptive or hormone replacement therapy a risk factor for cholelithiasis: A systematic review and meta-analysis. *Medicine* 2017;96(14):e6556.
67. de Klerk BM, Schiphof D, Groeneveld FP, et al. Limited evidence for a protective effect of unopposed oestrogen therapy for osteoarthritis of the hip: a systematic review. *Rheumatology* 2009;48(2):104-12.
68. Salpeter SR, Cheng J, Thabane L, Buckley NS, Salpeter EE. Bayesian meta-analysis of hormone therapy and mortality in younger postmenopausal women. *Am J Med* 2009;122(11):1016-22.e1.
