# Supplementary material for: Menopausal hormone therapy and women’s health: An umbrella review
Source: PLoS Med. 2021 Aug 2;18(8):e1003731. doi: 10.1371/journal.pmed.1003731 (PMC8366967; doi:10.1371/journal.pmed.1003731)
Supplement: Attachment — Submitted filename: Manuscript_commented.docx [file pmed.1003731.s007.docx]

**Menopausal Hormone Therapy and Women’s Health: An Umbrella Review of Systematic Reviews and Meta-Analyses of Randomized Controlled Trials and Observational Epidemiological Studies**

**Short Title:** Menopausal Hormone Therapy and Women’s Health

Guo-Qiang Zhang, MD; Jin-Liang Chen*, MD, PhD; Ying Luo*, MD; Maya B. Mathur*, PhD; Panagiotis Anagnostis, MD, PhD; Ulugbek Nurmatov, MD, PhD; Madar Talibov, PhD; Jing Zhang, MD; Catherine M. Hawrylowicz, PhD; Mary Ann Lumsden, MD; Hilary Critchley, MD; Aziz Sheikh, MD; Bo Lundbäck, MD, PhD; Cecilia Lässer, PhD; Hannu Kankaanranta, MD, PhD; Siew Hwa Lee, PhD; Bright I. Nwaru, PhD

*Authors contributed equally to this manuscript, and are listed in alphabetical order by last name.

**Author Affiliations:** Krefting Research Centre, Institute of Medicine, Sahlgrenska Academy, University of Gothenburg, Gothenburg, Sweden (G.Q. Zhang, Lundbäck, Lässer, Kankaanranta, Nwaru); Department of Geriatrics, The First Affiliated Hospital of Chongqing Medical University, Chongqing, China (Chen); Department of Gastroenterology, Children’s Hospital of Chongqing Medical University, Chongqing, China (Luo); Quantitative Sciences Unit, Stanford University, Palo Alto, CA, USA (Mathur); Unit of Reproductive Endocrinology, 1st Department of Obstetrics and Gynecology, Medical School, Aristotle University of Thessaloniki, Thessaloniki, Greece (Anagnostis); Division of Population Medicine, School of Medicine, Cardiff University, Cardiff, UK (Nurmatov); UMR1086 INSERM - Unité de Recherche Interdisciplinaire pour la Prévention et le Traitement des Cancers (ANTICIPE), Centre de Lutte Contre le Cancer François Baclesse, Caen, France (Talibov); Department of Intensive Care Unit, Chongqing General Hospital, University of Chinese Academy of Sciences, Chongqing, China (J. Zhang); MRC and Asthma UK Centre in Allergic Mechanisms of Asthma, King’s College London, Guy’s Hospital, London, UK (Hawrylowicz); Section of Reproductive and Maternal Medicine, Glasgow Royal Infirmary Campus of the School of Medicine, University of Glasgow, Glasgow, UK (Lumsden); MRC Centre for Reproductive Health, Queen’s Medical Research Institute, University of Edinburgh, Edinburgh, UK (Critchley); Asthma UK Centre for Applied Research, Centre for Medical Informatics, Usher Institute, University of Edinburgh, Edinburgh, UK (Sheikh, Nwaru); Department of Respiratory Medicine, Seinäjoki Central Hospital, Seinäjoki, Finland and Faculty of Medicine and Health Technology, University of Tampere, Tampere, Finland (Kankaanranta); School of Nursing, Midwifery and Paramedic Practice, Robert Gordon University, Aberdeen, UK (Lee); Wallenberg Centre for Molecular and Translational Medicine, University of Gothenburg, Gothenburg, Sweden (Nwaru)

**Correspondence to:** Guo-Qiang Zhang, Krefting Research Centre, Institute of Medicine, Sahlgrenska Academy, University of Gothenburg, P.O. Box 424, Gothenburg SE-405 30, Sweden. E-mail: [guo-qiang.zhang@gu.se](mailto:guo-qiang.zhang@gu.se). Tel.: +46 079 3364568.

**Data Availability Statement:** The statistical analysis protocol and R scripts are respectively available in Supplement 3 and Supplements 4 and 5. The datasets are publicly available at the Open Science Framework (<https://osf.io/dsy37/?view_only=a5b0bfea96694a54a1b5886e3fb03765>).

**Funding:** GQZ was supported by the VBG Group Herman Krefting Foundation on Asthma and Allergy, and Sahlgrenska Academy, University of Gothenburg, Sweden. MBM was supported in part by the Franklin E. Fetzer Memorial Trust (award R2020-16). BIN was supported by the VBG Group Herman Krefting Foundation on Asthma and Allergy, Knut and Alice Wallenberg Foundation, and the Wallenberg Centre for Molecular and Translational Medicine, University of Gothenburg, Sweden. The funders had no role in study design, data collection and analysis, decision to publish, or preparation of the manuscript.

**Competing Interests:** The authors have declared that no competing interests exist.

**Word Count:** 3,571 words

**What is already known on this topic**

- For several decades, the possible health effects of menopausal hormone therapy (MHT), beyond alleviation of menopausal symptoms, have remained an ongoing debate.
- In 2015-2017, leading menopause societies (e.g., the North American Menopause Society) provided clinical practice guidelines for the use of MHT in women, but these lacked consistency in some critical outcomes (e.g., coronary heart disease and all-cause mortality).
- The guidelines often incorporated systematic reviews and meta-analyses as a key evidence support for the recommendations, but with little attention to their quality and scientific validity.

**What this study adds**

- The overview of the evidence landscape based on an umbrella review can help guideline developers and decision-makers to better appreciate the trade-offs between the benefits and harms associated with MHT use in women.
- Given that current evidence supporting the beneficial effects of MHT on coronary heart disease and all-cause mortality in young women (known as “timing hypothesis”) is only tentative, a re-examination of current practice guidelines is needed.
- Although systematic reviews are a key component of evidence-based medicine, clinicians should evaluate their credibility prior to considering applying their results in clinical practice.

**ABSTRACT**

**OBJECTIVE:** To comprehensively summarize evidence on the benefits and harms of menopausal hormone therapy (MHT) across diverse health outcomes in women.

**DESIGN:** Umbrella review of systematic reviews and meta-analyses.

**DATA SOURCES:** MEDLINE, EMBASE, and 10 other databases from inception to November 26, 2017, updated December 17, 2020.

**ELIGIBILITY CRITERIA:** Systematic reviews or meta-analyses of randomized controlled trials (RCTs) and observational studies investigating effects of MHT, including estrogen-alone therapy (ET) and estrogen plus progestin therapy (EPT), in perimenopausal or postmenopausal women in all countries and settings. All health outcomes in previous systematic reviews were included, including menopausal symptoms, surrogate endpoints, biomarkers, various morbidity outcomes, and mortality.

**RESULTS:** 60 systematic reviews were included, involving 102 meta-analyses of RCTs and 38 of observational studies, with 121 unique outcomes. The overall quality of included systematic reviews was moderate to poor. In meta-analyses of RCTs, MHT was beneficial for vasomotor symptoms (risk ratio 0.29, 95% confidence interval 0.17 to 0.50) and vaginal atrophy (intravaginal ET: 0.31, 0.12 to 0.81), as well as sexual function, all fracture, vertebral and non-vertebral fracture, diabetes mellitus, cardiovascular mortality (ET) and colorectal cancer (EPT), but harmful for stroke (1.17, 1.05 to 1.29) and venous thromboembolism (1.60, 0.99 to 2.58), as well as cardiovascular disease, cerebrovascular disease, non-fatal stroke, deep vein thrombosis, gallbladder disease, and lung cancer mortality (EPT). In meta-analyses of observational studies, MHT was associated with decreased risks of cataract, glioma, and esophageal, gastric and colorectal cancer, but increased risks of pulmonary embolism, cholelithiasis, asthma, meningioma, and thyroid and ovarian cancer. ET and EPT had opposite effects for breast and endometrial cancer, endometrial hyperplasia and Alzheimer’s disease.

**CONCLUSIONS:** MHT has a complex balance of benefits and harms on multiple health outcomes. Some effects differ qualitatively between ET and EPT. The quality of available evidence is only moderate to poor.

**Key Words:** estrogen, menopausal hormone therapy, meta-analysis, progestin, umbrella review

**INTRODUCTION**

Longevity is increasing worldwide for women. By 2050, the world’s women aged 50 years and older are estimated to total 1.6 billion, up from one billion in 2020.^1^ Natural menopause occurs at a mean age of 49 years.^2^ Vasomotor symptoms, including hot flashes and night sweats, are the hallmark symptoms of menopause, affecting approximately 75% of perimenopausal women, and may persist for a decade or longer.^3^ Besides, up to 84% of postmenopausal women experience genitourinary symptoms, such as vulvovaginal atrophy and incontinence.^4^ The burden of menopausal symptoms can considerably affect the personal, social and work lives of women.^3^ Menopausal hormone therapy (MHT) is the most effective treatment for managing vasomotor and genitourinary symptoms.^5,6^ In Western countries, there have been about 600 million woman-years of MHT use in the period 1970-2019, and about 12 million current users in the 2010s, of which six million users are in the USA and UK alone.^7,8^

For several decades, the possible health effects of MHT, beyond alleviation of menopausal symptoms, have been debated. Nevertheless, a systematic, comprehensive assessment on this regard is lacking. More recently, leading medical societies provided clinical practice guidelines for the use of MHT in women.^5,6,9-11^ The guidelines lacked consistency in some outcomes, such as coronary heart disease (CHD) and all-cause mortality.^5,6,9,10^ Often, these guidelines incorporated systematic reviews and meta-analyses as a key evidence support for the recommendations, but with little attention to their quality and scientific validity. Numerous empirical evaluations have found that poor quality and major flaws impede many published systematic reviews in diverse disciplines.^12-18^ In light of the uncertainty surrounding the effects of MHT on numerous health outcomes in women, it is important that the quality of systematic reviews contributing to current guidelines and recommendations be critically appraised in order to provide the highest level and most reliable basis for recommendations in clinical practice. Consequently, we performed an outcome-wide umbrella review to summarize the evidence across existing systematic reviews and meta-analyses on the effects of MHT in perimenopausal and postmenopausal women.^19^ We sought to provide a comprehensive overview of the benefits and harms of MHT use, evaluate the validity of effects reported across systematic reviews, assess potential biases in the literature, and examine the credibility of the methods of existing systematic reviews.

**METHODS**

An umbrella review is a review of published systematic reviews, with or without meta-analyses, related to a given topic or question.^20-22^ It systematically integrates evidence from multiple systematic reviews to present a comprehensive view of the evidence landscape, readily providing guideline developers and decision-makers with currently available highest level of evidence relevant to the question posed.^21,22^ The protocol for the current umbrella review was registered in PROSPERO (CRD42017083412). Ethical approval was not required for this study.

**Literature Search and Eligibility Criteria**

We searched MEDLINE, EMBASE, Cochrane Database of Systematic Reviews, Database of Abstracts of Reviews of Effects, ISI Web of Science, CINAHL, Google Scholar, AMED, Global Health, PsycINFO, CAB International and WHO Global Health Library from inception to November 26, 2017. No language restriction was applied. eTable 1 in Supplement 1 presents detailed search strategies for each electronic database. Two investigators independently screened the titles and/or abstracts and reviewed full-text articles for eligibility. Any discrepancies were resolved by discussion. References of the included articles were also manually checked to identify additional eligible articles.

Articles were selected for inclusion if they met the following PI(E)COS criteria: 1) Population: perimenopausal or postmenopausal women of any ethnicity in any country or setting; 2) Intervention or Exposure: any type of MHT, including estrogen-alone therapy (ET) and estrogen plus progestin therapy (EPT), at any dose, duration, and route of administration; 3) Comparator: placebo or no treatment; 4) Outcome: any health outcome or indicator, including menopausal symptoms; 5) Study design: systematic reviews, with or without meta-analyses, of randomized controlled trials (RCTs) and observational epidemiological studies (cohort and case-control design). More details on study selection can be found in eMethods in Supplement 1. We categorized outcomes according to the International Classification of Diseases, 11th Revision (ICD-11).^23^ We performed an updated search on December 17, 2020 for studies published since November 27, 2017, but the literature obtained did not warrant a change in the conclusions reached based on the originally included studies. A summary of the literature obtained from the updated search is given in Supplement 2.

**Data Extraction and Quality Assessment**

Two investigators independently extracted data and assessed the methodological quality of included systematic reviews using the updated 16-item AMSTAR-2 (A MeaSurement Tool to Assess systematic Reviews) instrument.^24^ Quality appraisal of individual studies is beyond the scope of an umbrella review. Any discrepancies were resolved by discussion or arbitration by a third investigator. More details on data extraction are available in eMethods and eFigures 1 and 2 in Supplement 1.

**Data Synthesis and Analysis**

We developed *a priori* an analysis protocol including justification of the statistical methods applied to this umbrella review (Supplement 3). In brief, for each meta-analysis we calculated the summary average effect and its 95% confidence interval (CI) using the random-effects robust variance estimation method.^25^ We quantified the extent of heterogeneity by estimating the between-study standard deviation ($\tau$).^26,27^ To further account for heterogeneity, we estimated the predictive distribution for the true effect in a new study.^28-31^ Next, we examined small-study effects with a random-effects Egger’s regression.^32,33^ To assess publication bias, we used the Vevea and Hedges selection model,^34^ and the S-value, which represents the severity of publication bias that would hypothetically be required to shift the point estimate to the null.^35^ Lastly, for meta-analyses of observational studies, we assessed the robustness of meta-analysis results to potential residual confounding using the E-value^36,37^ and its equivalents for meta-analyses.^31,38^

The primary analyses focused on the average effects of any type of MHT (ET or EPT). We grouped the data according to whether the intervention or exposure was primary or secondary prevention. A subgroup analysis by MHT type was conducted to assess whether the effect varied qualitatively between ET and EPT. The analyses were conducted separately for RCTs and observational studies. For observational studies, the analyses were stratified by recency of MHT use (ever, current or past). Finally, based on the amount and the consistency of the body of evidence, we graded the evidence from meta-analyses as consistent, highly suggestive, suggestive, controversial, or insufficient, following the criteria described in Tables 1 and 2. All estimates were converted to the risk ratio (RR) scale,^36,39-41^ and results were presented on that scale, except where otherwise noted. All statistical analyses were performed using R software (version 4.0.1).^42^ The R scripts and datasets are respectively available in Supplements 4 and 5 and at the Open Science Framework (<https://osf.io/dsy37/?view_only=a5b0bfea96694a54a1b5886e3fb03765>).

**RESULTS**

**Characteristics of Included Studies**

Overall, 60 systematic reviews were included,^e1-e4,e6,e11-e65^ which involved 102 meta-analyses of RCTs and 38 meta-analyses of observational studies, with a total of 121 unique outcomes (Figure 1). Characteristics of the included systematic review(s) for each outcome are summarized in eTables 2 and 3 in Supplement 1. Figure 2 presents a summary of quality assessment by outcome across all included systematic reviews. Each AMSTAR-2 domain judgement for each outcome (eTables 4 and 5) and details of study characteristics and quality appraisal (eResults) are available in Supplement 1.

**Benefits and Harms of MHT**

In total, we included 936 individual study estimates from RCTs and 380 from observational studies (190 from case-control and 190 from cohort studies) for meta-analysis. The median number of study estimates per outcome in meta-analyses of RCTs and of observational studies was 5 (range 1-55) and 7 (range 1-71), respectively. Tables 1 and 2 summarize the evidence for all outcomes from meta-analyses of RCTs and of observational studies, respectively. More detailed results are available in eTables 6-24 in Supplement 1. Below we described the meta-analysis results for only outcomes with consistent or highly suggestive evidence (Figures 3A and B; eFigure 3 in Supplement 1). The meta-analysis results for other evidence levels, as well as small-study effects, publication bias, and sensitivity analysis for residual confounding in observational studies are described in eResults in Supplement 1.

**Consistent or highly suggestive beneficial effects of MHT**

***Genitourinary system***

In RCTs, MHT was associated with improved vasomotor symptoms (frequency: RR 0.43, 95%CI 0.33-0.57; severity: 0.29, 95%CI 0.17-0.50) and urinary incontinence (0.82, 95%CI 0.62-1.09). Intravaginal ET was associated with improved vaginal atrophy (0.31, 95%CI 0.12-0.81).

***Functioning assessment***

In RCTs, MHT was associated with improved sexual function (RR 0.82, 95%CI 0.71-0.96).

***Bone loss and fracture***

In RCTs, MHT was associated with increased bone mineral density at lumbar spine, forearm, femoral neck and proximal femur (eFigure 3 in Supplement 1), and decreased risks of all fracture (RR 0.72, 95%CI 0.62-0.84), vertebral fracture (0.69, 95%CI 0.50-0.94) and non-vertebral fracture (0.76, 95%CI 0.62-0.94).

***Neoplasms***

In RCTs, EPT was associated with a decreased risk of colorectal cancer (RR 0.79, 95%CI 0.64-0.98). In observational studies, MHT was associated with decreased risks of glioma (0.87, 95%CI 0.72-1.04), esophageal cancer (0.70, 95%CI 0.60-0.81), gastric cancer (0.78, 95%CI 0.70-0.86) and colorectal cancer (0.83, 95%CI 0.77-0.89). Among breast and ovarian cancer survivors, pre-/post-diagnosis MHT use was associated with improved breast cancer specific survival (0.72, 95%CI 0.59-0.88) and overall survival (0.82, 95%CI 0.75-0.89), and post-diagnosis MHT use was associated with improved ovarian cancer overall survival (0.81, 95%CI 0.71-0.91).

***Diseases of the nervous system***

In observational studies, ET was associated with a decreased risk of Alzheimer’s disease (RR 0.76, 95%CI 0.60-0.96).

***Diseases of the visual system***

In observational studies, MHT was associated with a decreased risk of cataract (RR 0.87, 95%CI 0.79-0.97).

***Endocrine, nutritional or metabolic diseases***

In RCTs, MHT was associated with lower levels of fasting glucose and fasting insulin and reduced insulin resistance in women with and without diabetes mellitus (eFigure 3 in Supplement 1), and a decreased risk of developing diabetes mellitus (RR 0.70, 95%CI 0.60-0.90). In addition, MHT was associated with lower levels of low-density lipoprotein cholesterol (LDL-C), lipoprotein (a) and plasminogen activator inhibitor-1 (PAI-1), but higher levels of triglycerides and C-reactive protein (eFigure 3 in Supplement 1).

***Diseases of the circulatory system***

In observational studies, MHT was associated with a decreased risk of CHD (RR 0.82, 95%CI 0.69-0.96). However, no evidence of effect was found in RCTs with regard to CHD incidence and mortality (17 trials, 1.02, 95%CI 0.82-1.26, and 6 trials, 0.96, 95%CI 0.46-2.00, respectively; eTable 6 in Supplement 1).

***Others, not elsewhere classified***

In RCTs, ET was associated with a small reduction in cardiovascular mortality (RR 0.97, 95%CI 0.95-0.99). In observational studies, MHT was associated with a decreased risk of all-cause mortality (0.89, 95%CI 0.82-0.97), but no evidence of effect was found in RCTs (38 trials, 0.99, 95%CI 0.83-1.18; eTable 6 in Supplement 1).

**Consistent or highly suggestive harmful effects of MHT**

***Genitourinary system***

In RCTs, oral ET was associated with an increased risk of endometrial hyperplasia (RR 6.93, 95%CI 2.07-23.23).

***Neoplasms***

In RCTs, EPT was associated with an increased risk of lung cancer mortality (RR 1.10, 95%CI 1.00-1.21). In observational studies, MHT was associated with increased risks of meningioma (1.14, 95%CI 0.98-1.33), thyroid cancer (1.09, 95%CI 0.88-1.34), ovarian cancer (1.16, 95%CI 1.06-1.26) and breast cancer (71 studies, 1.25, 95%CI 1.19-1.31; eTable 12 in Supplement 1). No qualitative difference on breast cancer risk between ET and EPT was found (Figure 3B). In addition, ET was associated with an increased risk of endometrial cancer (2.55, 95%CI 2.05-3.18).

***Diseases of the nervous system***

In RCTs, MHT was associated with increased risks of cerebrovascular disease (RR 1.25, 95%CI 1.04-1.50), stroke (1.17, 95%CI 1.05-1.29) and non-fatal stroke (1.35, 95%CI 1.08-1.69). In observational studies, EPT was associated with an increased risk of Alzheimer’s disease (1.42, 95%CI 1.24-1.62).

***Diseases of the respiratory system***

In observational studies, MHT was associated with an increased risk of asthma (RR 1.41, 95%CI 1.09-1.81).

***Diseases of the digestive system***

In RCTs, MHT was associated with an increased risk of gallbladder disease requiring surgery (RR 1.63, 95%CI 1.31-2.04). In observational studies, MHT was associated with an increased risk of cholelithiasis (1.63, 95%CI 1.41-1.88).

***Diseases of the circulatory system***

In RCTs, MHT was associated with increased risks of venous thromboembolism (RR 1.60, 95%CI 0.99-2.58) and deep vein thrombosis (1.39, 95%CI 0.68-2.84). In observational studies, MHT was associated with increased risks of venous thromboembolism (1.99, 95%CI 1.53-2.58), deep vein thrombosis (2.26, 95%CI 1.14-4.49) and pulmonary embolism (2.05, 95%CI 1.49-2.83).

***Others, not elsewhere classified***

In RCTs, MHT was associated with an increased risk of cardiovascular disease (RR 1.29, 95%CI 0.99-1.68).

**DISCUSSION**

**Summary of Key Findings**

In this umbrella review, consistent or highly suggestive evidence from RCTs found that MHT was beneficial for vasomotor symptoms, vaginal atrophy (intravaginal ET), sexual function, all fracture, vertebral and non-vertebral fracture, diabetes mellitus, cardiovascular mortality (ET) and colorectal cancer (EPT), but harmful for cardiovascular disease, cerebrovascular disease, stroke, non-fatal stroke, venous thromboembolism, deep vein thrombosis, gallbladder disease requiring surgery, and lung cancer mortality (EPT). Consistent or highly suggestive evidence from observational studies found that MHT was associated with decreased risks of cataract, glioma, and esophageal, gastric and colorectal cancer, but increased risks of venous thromboembolism, deep vein thrombosis, pulmonary embolism, cholelithiasis, asthma, meningioma, and thyroid and ovarian cancer. The effects of ET and EPT differed qualitatively for breast and endometrial cancer, endometrial hyperplasia and Alzheimer’s disease. The evidence levels for the other health outcomes ranged from suggestive to inconclusive.

**Breast and Ovarian Cancer**

In observational studies, we found that both ET and EPT were associated with increased incidence of breast and ovarian cancer. Our findings were consistent with the two recent individual participant meta-analyses of the worldwide epidemiological evidence.^7,8^ However, among breast and ovarian cancer survivors, pre- or post-diagnosis MHT use was associated with improved cancer-specific or overall survival. The findings were further replicated by two recent prospective cohort studies.^43,44^ One possible explanation could be that women who used MHT after diagnosis were likely to have used MHT before diagnosis. Therefore, despite increased incidence of breast and ovarian cancer with MHT use, MHT users with established breast and ovarian tumors may have better prognosis than non-users.^43,44^ Observational data on MHT and breast cancer mortality were controversial. Subgroup analysis by MHT type did not account for the divergent results. Data from the Million Women Study recently showed that both ET and EPT were associated with increased breast cancer mortality.^45^ It is therefore difficult to interpret the discrepancy in these results, but one speculation could be that any causal effect of MHT on breast cancer survival may reasonably result in such divergent results, since mortality reflects the net effects of MHT on both incidence and survival.^46^ Nevertheless, it is unclear whether the inverse association between MHT and breast cancer survival is causal, or due to residual confounding or other biases. In our analysis, small-study effects were also present. Further well-designed longitudinal studies of breast or ovarian cancer survivors across different settings, while controlling for different sources of bias, are warranted to assess causality.

In the Women’s Health Initiative (WHI) trials, a qualitative difference was found in the effects of ET and EPT on breast cancer incidence, with ET decreasing but EPT increasing the risk.^47^ Several explanations were proposed to reconcile the discordance between the WHI trials and observational studies: older women in the WHI trials, confounding in observational studies, different biological mechanisms of ET and EPT, or simply the play of chance in the WHI trials.^8,47^ Given the extensive amount of epidemiological evidence, replication of the findings from the WHI trials should be undertaken.

**CHD and All-Cause Mortality**

The effects of MHT on CHD and all-cause mortality have long been debated.^48-54^ In our analysis, observational data consistently showed up to 18% lower risk of CHD and up to 11% lower risk of all-cause mortality with ever use of MHT, while RCTs failed to support the presence of these beneficial effects. The distinction in populations of women between RCTs and observational studies, that is, observational studies generally included younger women who started MHT around the time of menopause, has led to the well-known “timing hypothesis”.^48,49^ According to this hypothesis, MHT reduces CHD and all-cause mortality only when initiated close to the onset of menopause, but does not or even increases when initiated many years later.^48,49^ Indeed, the age-stratification data from the WHI trials found that MHT reduced CHD and all-cause mortality in women aged 50-59 years, but increased CHD in older women.^55,56^ Four meta-analyses of RCTs assessed the timing hypothesis by stratifying trials according to mean time since menopause or mean age of participants at baseline, showing a highly significant reduction in CHD and all-cause mortality in women aged $<$ 60 years or those $<$ 10 years from menopause, but not in older women.^57-60^ Current guidelines and reviews widely adopted the WHI trials and the meta-analyses as part of the evidence supporting the beneficial effects of MHT in young group of women.^5,9,11,48-51^ However, it is worth noting that all these analyses are by nature *post hoc*, and such subgroup results are most appropriately regarded as hypothesis generation rather than hypothesis confirmation due to multiple comparisons.^10,53^ Particularly for the meta-analyses, because of issues of potential confounding and ecological fallacy, findings of such study-level analyses have to be interpreted cautiously.^61,62^ On the other hand, we did not find evidence that MHT caused additional events in women (mean baseline age $\geq$ 60 years) with established CHD.

The Early versus Late Intervention Trial with Estradiol (ELITE),^63^ the first trial specifically designed to test the timing hypothesis, found that oral estradiol reduced the progression of subclinical atherosclerosis (measured as carotid-artery intima–media thickness) when initiated within six years after menopause, but not when initiated $\geq$ 10 years. It is unclear whether this favorable effect on atherosclerosis will translate into reductions in risk of CHD.^53^ Mendelian randomization studies have demonstrated that several plasma biomarkers (e.g., LDL-C) have a causal role in CHD.^64-68^ Among these biomarkers, however, we found that the effects of MHT were mixed, with MHT reducing LDL-C, lipoprotein (a) and PAI-1, but increasing triglycerides. Overall, the available clinical data in support of the beneficial effects of MHT on CHD and all-cause mortality as well as the timing hypothesis are only suggestive.

**Urinary Incontinence**

The WHI trials found that oral ET or EPT increased the risk of urinary incontinence among continent women.^69^ The 2012 Cochrane review included 13 RCTs, comprising 10 small studies and three large studies, to assess the efficacy of MHT in treating urinary incontinence.^70^ Four small studies used intravaginal ET, while the rest used oral ET or EPT. Based on *post hoc* subgroup analysis, it concluded that intravaginal ET may improve incontinence, while oral ET or EPT may worsen incontinence. Though it seems rare to anticipate a qualitative difference between different routes of administration,^71^ this result was nevertheless carried into the guidelines without caveats.^5,9^ In our analysis, small-study effects were very prominent: all 10 small studies, regardless of route of administration, pointed in the direction of beneficial effects while the three large studies reported harmful effects. Taken together, current evidence shows that oral MHT increases the incidence and recurrence of urinary incontinence in postmenopausal women. The opposite effects of systemic and local MHT should be viewed at best as exploratory.

**Credibility of Meta-Analyses**

We found major methodological limitations in a substantial proportion of included meta-analyses, some of which warrant further discussion. First, a fixed-effect meta-analysis model, instead of a random-effects model, was often used even when heterogeneity was present or anticipated *a priori*. By gathering studies from the published literature, the random-effects assumption is the only plausible match to the underlying effect distribution.^72^ Second, although the random-effects model was used, interpretation of the summary effect as an estimate of a common effect under the fixed-effect model was often misplaced. Under the random-effects model, the summary effect is an estimate of the mean of a distribution of true effects across studies. Third, investigators often relied heavily on statistical methods (e.g., Egger’s test) to deal with publication bias. To address publication bias, there is need to obtain a representative sample of studies on a topic (e.g., through comprehensive literature searches). Fourth, the tools used for risk of bias assessment of individual studies were generally not comprehensive and risk of bias was often evaluated by studies across outcomes rather than by outcome across studies, which limited quality of evidence rating in existing meta-analyses.

**Limitations**

Certain limitations need to be taken into account in the interpretation of our findings. First, umbrella reviews focus on existing systematic reviews, and therefore outcomes not assessed in a systematic review cannot be included. Second, quality appraisal of individual studies is not the focus of umbrella reviews, as included systematic reviews are expected to perform this task. However, as discussed earlier, included systematic reviews are generally limited in their usefulness for accurate rating of quality of evidence. Third, it can be expected that the effects of MHT may vary by the participants (e.g., age) and the treatment (MHT type, dose, duration, and route of administration), and the range of effects can be estimated using the predictive distribution.^29-31^ Thus, the random-effects average effect should not be interpreted in isolation, but rather in conjunction with the predictive distribution and the effect from the subset of studies most relevant to the patients needed to treat.^73^

In conclusion, MHT has a complex balance of benefits and harms on various health outcomes. Some effects differ qualitatively between ET and EPT. Decisions regarding the use of MHT should consider the full range of effects, along with patients’ values and preferences. The overall quality of existing systematic reviews is moderate to poor. Clinicians should evaluate their credibility prior to considering applying their results in clinical practice.

**Author Contributions:** GQZ, SHL, and BIN contributed to the study concept and design. GQZ, JLC, YL, PA, UN, MT, JZ, SHL, and BIN contributed to the acquisition of data. All authors analyzed and interpreted the data. GQZ and BIN drafted the manuscript with critical revision for important intellectual content from all authors. GQZ, MBM, and BIN did the statistical analysis. JLC, YL, and MBM contributed equally to this manuscript, and are listed in alphabetical order by last name. All authors reviewed and approved the final version of the manuscript. GQZ is the guarantor. We thank the Biomedical Library at University of Gothenburg for retrieving the full-text articles. We also thank [WAITING FOR WRITTEN PERMISSION] for providing additional data or information. They were not compensated for their contribution.

**REFERENCES**

1.	United Nations, Department of Economic and Social Affairs, Population Division. World Population Prospects 2019. <https://population.un.org/wpp/DataQuery/>. Accessed November 27, 2020.

2.	Schoenaker DA, Jackson CA, Rowlands JV, Mishra GD. Socioeconomic position, lifestyle factors and age at natural menopause: a systematic review and meta-analyses of studies across six continents. *Int J Epidemiol* 2014;43:1542-62. doi:10.1093/ije/dyu094

3.	Monteleone P, Mascagni G, Giannini A, Genazzani AR, Simoncini T. Symptoms of menopause - global prevalence, physiology and implications. *Nat Rev Endocrinol* 2018;14:199-215. doi:10.1038/nrendo.2017.180

4.	The 2020 Genitourinary Syndrome of Menopause Position Statement of The North American Menopause Society. *Menopause* 2020;27:976-92. doi:10.1097/GME.0000000000001609

5.	The 2017 Hormone Therapy Position Statement of The North American Menopause Society. *Menopause* 2017;24:728-53. doi:10.1097/GME.0000000000000921

6.	Stuenkel CA, Davis SR, Gompel A, et al. Treatment of symptoms of the menopause: An Endocrine Society Clinical Practice Guideline. *J Clin Endocrinol Metab* 2015;100:3975-4011. doi:10.1210/jc.2015-2236

7.	Beral V, Gaitskell K, Hermon C, Moser K, Reeves G, Peto R. Menopausal hormone use and ovarian cancer risk: individual participant meta-analysis of 52 epidemiological studies. *Lancet* 2015;385:1835-42. doi:10.1016/S0140-6736(14)61687-1

8.	Type and timing of menopausal hormone therapy and breast cancer risk: individual participant meta-analysis of the worldwide epidemiological evidence. *Lancet* 2019;394:1159-68. doi:10.1016/S0140-6736(19)31709-X

9.	Baber RJ, Panay N, Fenton A. 2016 IMS Recommendations on women's midlife health and menopause hormone therapy. *Climacteric* 2016;19:109-50. doi:10.3109/13697137.2015.1129166

10.	Grossman DC, Curry SJ, Owens DK, et al. Hormone therapy for the primary prevention of chronic conditions in postmenopausal women: US Preventive Services Task Force Recommendation Statement. *JAMA* 2017;318:2224-33. doi:10.1001/jama.2017.18261

11.	de Villiers TJ, Hall JE, Pinkerton JV, et al. Revised global consensus statement on menopausal hormone therapy. *Maturitas* 2016;91:153-5. doi:10.1016/j.maturitas.2016.06.001

12.	Sathish M, Eswar R. Systematic reviews and meta-analysis in spine surgery-how good are they in methodological quality? a systematic review. *Global Spine J* 2020; published online February 26. doi:10.1177/2192568220906810

13.	Salim A, Mullassery D, Losty PD. Quality of systematic reviews and meta-analyses published in pediatric surgery. *J Pediatr Surg* 2017;52:1732-5. doi:10.1016/j.jpedsurg.2017.07.022

14.	Ioannidis JP. The mass production of redundant, misleading, and conflicted systematic reviews and meta-analyses. *Milbank Q* 2016;94:485-514. doi:10.1111/1468-0009.12210

15.	Rudmik LR, Walen SG, Dixon E, Dort J. Evaluation of meta-analyses in the otolaryngological literature. *Otolaryngol Head Neck Surg* 2008;139:187-94. doi:10.1016/j.otohns.2008.03.020

16.	Delaney A, Bagshaw SM, Ferland A, Manns B, Laupland KB, Doig CJ. A systematic evaluation of the quality of meta-analyses in the critical care literature. *Crit Care* 2005;9:R575-82. doi:10.1186/cc3803

17.	Jadad AR, Moher M, Browman GP, et al. Systematic reviews and meta-analyses on treatment of asthma: critical evaluation. *BMJ* 2000;320:537-40. doi:10.1136/bmj.320.7234.537

18.	Dijkman BG, Abouali JA, Kooistra BW, et al. Twenty years of meta-analyses in orthopaedic surgery: has quality kept up with quantity? *J Bone Joint Surg Am* 2010;92:48-57. doi:10.2106/JBJS.I.00251

19.	VanderWeele TJ, Mathur MB, Chen Y. Outcome-wide longitudinal designs for causal inference: a new template for empirical studies. *Stat Sci* 2020;35:437-66. doi:10.1214/19-STS728

20.	Ioannidis JP. Integration of evidence from multiple meta-analyses: a primer on umbrella reviews, treatment networks and multiple treatments meta-analyses. *CMAJ* 2009;181:488-93. doi:10.1503/cmaj.081086

21.	Aromataris E, Fernandez R, Godfrey CM, Holly C, Khalil H, Tungpunkom P. Summarizing systematic reviews: methodological development, conduct and reporting of an umbrella review approach. *Int J Evid Based Healthc* 2015;13:132-40. doi:10.1097/XEB.0000000000000055

22.	Fusar-Poli P, Radua J. Ten simple rules for conducting umbrella reviews. *Evid Based Ment Health* 2018;21:95-100. doi:10.1136/ebmental-2018-300014

23.	World Health Organization. International Classification of Diseases, 11th Revision. <https://icd.who.int/browse11/l-m/en>. Accessed March 16, 2020.

24.	Shea BJ, Reeves BC, Wells G, et al. AMSTAR 2: a critical appraisal tool for systematic reviews that include randomised or non-randomised studies of healthcare interventions, or both. *BMJ* 2017;358:j4008. doi:10.1136/bmj.j4008

25.	Hedges LV, Tipton E, Johnson MC. Robust variance estimation in meta-regression with dependent effect size estimates. *Res Synth Methods* 2010;1:39-65. doi:10.1002/jrsm.5

26.	Higgins JP, Thompson SG. Quantifying heterogeneity in a meta-analysis. *Stat Med* 2002;21:1539-58. doi:10.1002/sim.1186

27.	Rücker G, Schwarzer G, Carpenter JR, Schumacher M. Undue reliance on I^2^ in assessing heterogeneity may mislead. *BMC Med Res Methodol* 2008;8:79. doi:10.1186/1471-2288-8-79

28.	Higgins JP, Thompson SG, Spiegelhalter DJ. A re-evaluation of random-effects meta-analysis. *J R Stat Soc Ser A Stat Soc* 2009;172:137-59. doi:10.1111/j.1467-985X.2008.00552.x

29.	Wang CC, Lee WC. A simple method to estimate prediction intervals and predictive distributions: summarizing meta-analyses beyond means and confidence intervals. *Res Synth Methods* 2019;10:255-66. doi:10.1002/jrsm.1345

30.	Mathur MB, VanderWeele TJ. New metrics for meta-analyses of heterogeneous effects. *Stat Med* 2019;38:1336-42. doi:10.1002/sim.8057

31.	Mathur MB, VanderWeele TJ. Robust metrics and sensitivity analyses for meta-analyses of heterogeneous effects. *Epidemiology* 2020;31:356-8. doi:10.1097/EDE.0000000000001180

32.	Sterne JA, Gavaghan D, Egger M. Publication and related bias in meta-analysis: power of statistical tests and prevalence in the literature. *J Clin Epidemiol* 2000;53:1119-29. doi:10.1016/s0895-4356(00)00242-0

33.	Egger M, Davey Smith G, Schneider M, Minder C. Bias in meta-analysis detected by a simple, graphical test. *BMJ* 1997;315:629-34. doi:10.1136/bmj.315.7109.629

34.	Vevea JL, Hedges LV. A general linear model for estimating effect size in the presence of publication bias. *Psychometrika* 1995;60:419-35. doi:10.1007/bf02294384

35.	Mathur MB, VanderWeele TJ. Sensitivity analysis for publication bias in meta-analyses. *J R Stat Soc Ser C Appl Stat* 2020;69:1091-119. doi:10.1111/rssc.12440

36.	VanderWeele TJ, Ding P. Sensitivity analysis in observational research: introducing the E-value. *Ann Intern Med* 2017;167:268-74. doi:10.7326/M16-2607

37.	Mathur MB, Ding P, Riddell CA, VanderWeele TJ. Website and R package for computing E-values. *Epidemiology* 2018;29:e45-7. doi:10.1097/EDE.0000000000000864

38.	Mathur MB, VanderWeele TJ. Sensitivity analysis for unmeasured confounding in meta-analyses. *J Am Stat Assoc* 2020;115:163-72. doi:10.1080/01621459.2018.1529598

39.	Hasselblad V, Hedges LV. Meta-analysis of screening and diagnostic tests. *Psychol Bull* 1995;117:167-78. doi:10.1037/0033-2909.117.1.167

40.	VanderWeele TJ. Optimal approximate conversions of odds ratios and hazard ratios to risk ratios. *Biometrics* 2020;76:746-52. doi:10.1111/biom.13197

41.	Hedges LV. Distribution theory for Glass's estimator of effect size and related estimators. *J Educ Behav Stat* 1981;6:107-28. doi:10.2307/1164588

42.	R Core Team (2020). R: a language and environment for statistical computing. R Foundation for Statistical Computing, Vienna, Austria. URL https://www.R-project.org/.

43.	Wang T, Bradshaw PT, Moorman PG, et al. Menopausal hormone therapy use and long-term all-cause and cause-specific mortality in the Long Island Breast Cancer Study Project. *Int J Cancer* 2020;147:3404-15. doi:10.1002/ijc.33174

44.	Brieger KK, Peterson S, Lee AW, et al. Menopausal hormone therapy prior to the diagnosis of ovarian cancer is associated with improved survival. *Gynecol Oncol* 2020;158:702-9. doi:10.1016/j.ygyno.2020.06.481

45.	Beral V, Peto R, Pirie K, Reeves G. Menopausal hormone therapy and 20-year breast cancer mortality. *Lancet* 2019;394:1139. doi:10.1016/S0140-6736(19)32033-1

46.	Ellis L, Woods LM, Estève J, Eloranta S, Coleman MP, Rachet B. Cancer incidence, survival and mortality: explaining the concepts. *Int J Cancer* 2014;135:1774-82. doi:10.1002/ijc.28990

47.	Chlebowski RT, Anderson GL, Aragaki AK, et al. Association of menopausal hormone therapy with breast cancer incidence and mortality during long-term follow-up of the Women's Health Initiative randomized clinical trials. *JAMA* 2020;324:369-80. doi:10.1001/jama.2020.9482

48.	Hodis HN, Collins P, Mack WJ, Schierbeck LL. The timing hypothesis for coronary heart disease prevention with hormone therapy: past, present and future in perspective. *Climacteric* 2012;15:217-28. doi:10.3109/13697137.2012.656401

49.	Clarkson TB, Meléndez GC, Appt SE. Timing hypothesis for postmenopausal hormone therapy: its origin, current status, and future. *Menopause* 2013;20:342-53. doi:10.1097/GME.0b013e3182843aad

50.	Lobo RA. Hormone-replacement therapy: current thinking. *Nat Rev Endocrinol* 2017;13:220-31. doi:10.1038/nrendo.2016.164

51.	Manson JE, Bassuk SS, Kaunitz AM, Pinkerton JV. The Women's Health Initiative trials of menopausal hormone therapy: lessons learned. *Menopause* 2020;27:918-28. doi:10.1097/GME.0000000000001553

52.	Langer RD, Simon JA, Pines A, et al. Menopausal hormone therapy for primary prevention: why the USPSTF is wrong. *Climacteric* 2017;20:402-13. doi:10.1080/13697137.2017.1362156

53.	Keaney JF, Jr., Solomon CG. Postmenopausal hormone therapy and atherosclerosis--time is of the essence. *N Engl J Med* 2016;374:1279-80. doi:10.1056/NEJMe1602846

54.	Humphrey LL, Chan BK, Sox HC. Postmenopausal hormone replacement therapy and the primary prevention of cardiovascular disease. *Ann Intern Med* 2002;137:273-84. doi:10.7326/0003-4819-137-4-200208200-00012

55.	Manson JE, Chlebowski DRT, Stefanick ML, et al. The Women’s Health Initiative hormone therapy trials: update and overview of health outcomes during the intervention and post-stopping phases. *JAMA* 2013;310:1353-68. doi:10.1001/jama.2013.278040

56.	Manson JE, Aragaki AK, Rossouw JE, et al. Menopausal hormone therapy and long-term all-cause and cause-specific mortality: the Women's Health Initiative randomized trials. *JAMA* 2017;318:927-38. doi:10.1001/jama.2017.11217

57.	Salpeter SR, Cheng J, Thabane L, Buckley NS, Salpeter EE. Bayesian meta-analysis of hormone therapy and mortality in younger postmenopausal women. *Am J Med* 2009;122:1016-22. doi:10.1016/j.amjmed.2009.05.021

58.	Salpeter SR, Walsh JM, Greyber E, Ormiston TM, Salpeter EE. Mortality associated with hormone replacement therapy in younger and older women: a meta-analysis. *J Gen Intern Med* 2004;19:791-804. doi:10.1111/j.1525-1497.2004.30281.x

59.	Salpeter SR, Walsh JM, Greyber E, Salpeter EE. Brief report: coronary heart disease events associated with hormone therapy in younger and older women. A meta-analysis. *J Gen Intern Med* 2006;21:363-6. doi:10.1111/j.1525-1497.2006.00389.x

60.	Boardman HM, Hartley L, Eisinga A, et al. Hormone therapy for preventing cardiovascular disease in post-menopausal women. *Cochrane Database Syst Rev* 2015;(3):CD002229. doi:10.1002/14651858.CD002229.pub4

61.	Gartlehner G, Patel SV, Feltner C, et al. Hormone therapy for the primary prevention of chronic conditions in postmenopausal women: evidence report and systematic review for the US Preventive Services Task Force. *JAMA* 2017;318:2234-49. doi:10.1001/jama.2017.16952

62.	Richardson M, Garner P, Donegan S. Interpretation of subgroup analyses in systematic reviews: a tutorial. *Clin Epidemiol Glob Health* 2019;7:192-8. doi:10.1016/j.cegh.2018.05.005

63.	Hodis HN, Mack WJ, Henderson VW, et al. Vascular effects of early versus late postmenopausal treatment with estradiol. *N Engl J Med* 2016;374:1221-31. doi:10.1056/NEJMoa1505241

64.	Cohen JC, Boerwinkle E, Mosley TH, Jr., Hobbs HH. Sequence variations in PCSK9, low LDL, and protection against coronary heart disease. *N Engl J Med* 2006;354:1264-72. doi:10.1056/NEJMoa054013

65.	Holmes MV, Asselbergs FW, Palmer TM, et al. Mendelian randomization of blood lipids for coronary heart disease. *Eur Heart J* 2015;36:539-50. doi:10.1093/eurheartj/eht571

66.	Sarwar N, Sandhu MS, Ricketts SL, et al. Triglyceride-mediated pathways and coronary disease: collaborative analysis of 101 studies. *Lancet* 2010;375:1634-9. doi:10.1016/S0140-6736(10)60545-4

67.	Kamstrup PR, Tybjaerg-Hansen A, Steffensen R, Nordestgaard BG. Genetically elevated lipoprotein(a) and increased risk of myocardial infarction. *JAMA* 2009;301:2331-9. doi:10.1001/jama.2009.801

68.	Song C, Burgess S, Eicher JD, O'Donnell CJ, Johnson AD. Causal effect of plasminogen activator inhibitor type 1 on coronary heart disease. *J Am Heart Assoc* 2017;6:e004918. doi:10.1161/JAHA.116.004918

69.	Hendrix SL, Cochrane BB, Nygaard IE, et al. Effects of estrogen with and without progestin on urinary incontinence. *JAMA* 2005;293:935-48. doi:10.1001/jama.293.8.935

70.	Cody JD, Jacobs ML, Richardson K, Moehrer B, Hextall A. Oestrogen therapy for urinary incontinence in post-menopausal women. *Cochrane Database Syst Rev* 2012;10:CD001405. doi:10.1002/14651858.CD001405.pub3

71.	Yusuf S, Collins R, Peto R. Why do we need some large, simple randomized trials? *Stat Med* 1984;3:409-22. doi:10.1002/sim.4780030421

72.	Borenstein M, Hedges LV, Higgins JP, Rothstein HR. A basic introduction to fixed-effect and random-effects models for meta-analysis. *Res Synth Methods* 2010;1:97-111. doi:10.1002/jrsm.12

73.	Serghiou S, Goodman SN. Random-effects meta-analysis: summarizing evidence with caveats. *JAMA* 2019;321:301-2. doi:10.1001/jama.2018.19684

**Figure Legends**

**Figure 1. Flow Chart for Study Selection Process**

Abbreviations: MHT, menopausal hormone therapy; OE, observational epidemiological study; RCT, randomized controlled trial.

**Figure 2. Quality Assessment by Outcome Presented as Percentages across All Included Systematic Reviews**

Abbreviations: PI(E)CO, Population, Intervention or Exposure, Comparator, Outcome; RoB, risk of bias.

**Figure 3. Consistent or Highly Suggestive Evidence from Meta-Analyses of Randomized Controlled Trials and Observational Epidemiological Studies on Menopausal Hormone Therapy and Incidence of Diseases and Other Health Outcomes**

A, Meta-analyses of randomized controlled trials. B, Meta-analyses of observational epidemiological studies. The average effects of any menopausal hormone therapy (estrogen-alone therapy or estrogen plus progestin therapy) in perimenopausal or postmenopausal women, unless otherwise stated. For meta-analyses of observational studies, the estimates were for ever use of menopausal hormone therapy, unless otherwise stated. All estimates were from our own analysis apart from diabetes mellitus. Subgroup analysis results by type and recency of menopausal hormone therapy use can be found in eTables 6-17 in Supplement 1. Abbreviations: Cc, case-control study; CI, confidence interval; Co, cohort study; EPT, estrogen plus progestin therapy; ET, estrogen-alone therapy; NA, not available or not applicable.

^a^ In women who already have the outcomes of interest.

^b^ The effect measures for continuous outcomes were converted into risk ratio scale for comparison. The results on its original scale can be found in eTables 6 and 9 in Supplement 1.

^c^ Current use of menopausal hormone therapy.

^d^ Use of menopausal hormone therapy before or after diagnosis of cancer.

^e^ Use of menopausal hormone therapy after diagnosis of cancer.

**Table 1. Summary of Evidence Grading for Meta-Analyses of Randomized Controlled Trials on Menopausal Hormone Therapy and Incidence of Diseases and Other Health Outcomes**

| **Evidence** | **Criteria** | **Meta-Analyses of Randomized Controlled Trials^a^** | |
| --- | --- | --- | --- |
|  |  | **Outcomes with Beneficial Effects** | **Outcomes with Harmful Effects** |
| Consistent | 95% CI of the mean effect excludes null value with no heterogeneity; or predictive distribution^b^ contains an extreme proportion ($>$ 90%) of true effects in the direction of the mean effect | All fracture, vertebral fracture, non-vertebral fracture, colorectal cancer (EPT), cardiovascular mortality (ET) | Cardiovascular disease, cerebrovascular disease, stroke, non-fatal stroke, venous thromboembolism, deep vein thrombosis, gallbladder disease requiring surgery, endometrial hyperplasia (ET), lung cancer mortality (EPT) |
| Highly suggestive | 95% CI of the mean effect excludes null value, with heterogeneity present but predictive distribution not estimable^c^; or predictive distribution contains a substantial proportion (70%-90%) of true effects in the direction of the mean effect | Vasomotor symptom^d^, vasomotor symptom severity^d^, vaginal atrophy (intravaginal ET)^d^, sexual function, urinary incontinence^d^, diabetes mellitus | None |
| Suggestive | 95% CI of the mean effect includes null value; predictive distribution not estimable^c^; and 95% CI of the most precise study^e^ excludes null value | Breast cancer (ET), endometrial cancer (EPT), hip fracture (EPT), sleep quality (EPT), skeletal muscle strength (EPT), recurrent urinary tract infection (ET)^d^ | Breast cancer (EPT), dementia (EPT), pulmonary embolism (EPT), irregular vaginal bleeding (ET) |
| Controversial | Predictive distribution contains a non-negligible proportion ($>$ 30%) of true effects in both the same and the opposite direction of the mean effect | None | None |
| Insufficient | Insufficient evidence to draw conclusions | All-cause mortality, all-cancer incidence and mortality, colorectal cancer mortality, lung cancer, breast cancer recurrence and mortality, ovarian cancer incidence and overall survival, fatal stroke, transient ischaemic attack, coronary heart disease incidence and mortality, myocardial infarction, fatal myocardial infarction, non-fatal myocardial infarction, angina pectoris, cardiac death, coronary revascularization, depressive symptom, Alzheimer’s disease, cognitive function (in healthy women and in women with dementia) | |
| *Notes:* Small-study effects^f^ existed for all fracture, sexual function, urinary incontinence and deep vein thrombosis; meta-analysis results were robust to severe or extreme publication bias^g^ for all fracture, vasomotor symptom, stroke, non-fatal stroke, venous thromboembolism, gallbladder disease requiring surgery, and endometrial hyperplasia (ET). | | | |

Abbreviations: CI, confidence interval; EPT, estrogen plus progestin therapy; ET, estrogen-alone therapy.

^a^ The average effects of any menopausal hormone therapy (estrogen-alone therapy or estrogen plus progestin therapy) in perimenopausal or postmenopausal women. When subgroup analysis by type of hormone therapy indicated a qualitative difference, results were presented separately for them. When statistically significant results were found for only one type of hormone therapy, results were presented for only that type.

^b^ The predictive distribution describes how the true effect sizes across studies are distributed around the summary average effect. Predictive distribution was estimated only in meta-analyses of $\geq$ 10 studies. More information is available in Supplement 3.

^c^ Due to a small number of studies ($<$ 10) included in meta-analysis.

^d^ In women who already have the outcomes of interest.

^e^ The study with the smallest standard error in each meta-analysis.

^f^ Egger’s regression test was used to examine whether smaller studies tended to show more pronounced effects than larger studies (small-study effects). It was applied only in meta-analyses of $\geq$ 10 studies. More information is available in Supplement 3.

^g^ “Robust to severe or extreme publication bias”: The meta-analysis results cannot be explained away by hypothetical publication bias that greatly exceeds empirical estimates of publication bias severity in medicine (i.e., hypothetical publication bias in which statistically significant positive effects are more 4-fold likely to be published and meta-analyzed than nonsignificant or negative effects). More information is available in Supplement 3.

**Table 2. Summary of Evidence Grading for Meta-Analyses of Observational Epidemiological Studies on Menopausal Hormone Therapy and Incidence of Diseases and Other Health Outcomes**

| **Evidence** | **Criteria** | **Meta-Analyses of Observational Epidemiological Studies^a^** | |
| --- | --- | --- | --- |
|  |  | **Outcomes with Beneficial Effects** | **Outcomes with Harmful Effects** |
| Consistent | 95% CI of the mean effect excludes null value with no heterogeneity; or predictive distribution^b^ contains an extreme proportion ($>$ 90%) of true effects in the direction of the mean effect | Esophageal cancer, gastric cancer, colorectal cancer, breast cancer specific survival and overall survival^c^, ovarian cancer overall survival^d^, Alzheimer’s disease (ET), cataract, coronary heart disease, all-cause mortality | Breast cancer (EPT), endometrial cancer (ET), Alzheimer’s disease (EPT), venous thromboembolism, deep vein thrombosis, pulmonary embolism, cholelithiasis, asthma |
| Highly suggestive | 95% CI of the mean effect excludes null value, with heterogeneity present but predictive distribution not estimable^e^; or predictive distribution contains a substantial proportion (70%-90%) of true effects in the direction of the mean effect | Glioma | Breast cancer (ET), ovarian cancer, meningioma, thyroid cancer |
| Suggestive | 95% CI of the mean effect includes null value; predictive distribution not estimable^e^; and 95% CI of the most precise study^f^ excludes null value | Breast cancer recurrence^d^, lung cancer overall survival^g^, diabetes mellitus, coronary heart disease mortality, cardiovascular disease incidence and mortality | Cutaneous melanoma (ET), endometrial cancer (EPT), systemic lupus erythematosus, Parkinson’s disease |
| Controversial | Predictive distribution contains a non-negligible proportion ($>$ 30%) of true effects in both the same and the opposite direction of the mean effect | Breast cancer mortality, pancreatic cancer, lung cancer | |
| Insufficient | Insufficient evidence to draw conclusions | Primary liver cancer, endometrial cancer mortality, ovarian cancer recurrence, head and neck cancer, osteoarthritis, dementia | |
| *Notes:* Small-study effects^h^ existed for breast cancer specific survival and overall survival, and glioma; meta-analysis results were robust to severe or extreme publication bias^i^ for esophageal cancer, gastric cancer, colorectal cancer, breast cancer (EPT), breast cancer (ET), breast cancer specific survival and overall survival, endometrial cancer (ET), ovarian cancer incidence and overall survival, coronary heart disease, venous thromboembolism, asthma and cholelithiasis. | | | |

Abbreviations: CI, confidence interval; EPT, estrogen plus progestin therapy; ET, estrogen-alone therapy.

^a^ The average effects of any menopausal hormone therapy (estrogen-alone therapy or estrogen plus progestin therapy) in perimenopausal or postmenopausal women. When subgroup analysis by type of hormone therapy indicated a qualitative difference, results were presented separately for them. When statistically significant results were found for only one type of hormone therapy, results were presented for only that type.

^b^ The predictive distribution describes how the true effect sizes across studies are distributed around the summary average effect. Predictive distribution was estimated only in meta-analyses of $\geq$ 10 studies. More information is available in Supplement 3.

^c^ Use of menopausal hormone therapy before or after diagnosis of cancer.

^d^ Use of menopausal hormone therapy after diagnosis of cancer.

^e^ Due to a small number of studies ($<$ 10) included in meta-analysis.

^f^ The study with the smallest standard error in each meta-analysis.

^g^ Use of menopausal hormone therapy before diagnosis of cancer.

^h^ Egger’s regression test was used to examine whether smaller studies tended to show more pronounced effects than larger studies (small-study effects). It was applied only in meta-analyses of $\geq$ 10 studies. More information is available in Supplement 3.

^i^ “Robust to severe or extreme publication bias”: The meta-analysis results cannot be explained away by hypothetical publication bias that greatly exceeds empirical estimates of publication bias severity in medicine (i.e., hypothetical publication bias in which statistically significant positive effects are more 4-fold likely to be published and meta-analyzed than nonsignificant or negative effects). More information is available in Supplement 3.
