# Supplementary material for: Menopausal hormone therapy and women’s health: An umbrella review
Source: PLoS Med. 2021 Aug 2;18(8):e1003731. doi: 10.1371/journal.pmed.1003731 (PMC8366967; doi:10.1371/journal.pmed.1003731)
Supplement: Attachment — Submitted filename: Response to Reviewers.docx [file pmed.1003731.s008.docx]

*(The location of contents indicated in the ‘Response to Reviewers’ refers to the ‘Revised Article with Changes Highlighted’)*

**Comments from editors**

1. Please revise your title according to PLOS Medicine's style. Your title must be nondeclarative and not a question. It should begin with main concept if possible. "Effect of" should be used only if causality can be inferred, i.e., for an RCT. Please place the study design ("A randomized controlled trial," "A retrospective study," "A modelling study," etc.) in the subtitle (i.e., after a colon). For example, “Menopausal Hormone Therapy and Women’s Health: An Umbrella Review”.

**Answer:** The title of the manuscript has been revised to “Menopausal hormone therapy and women’s health: An umbrella review” (p. 1, lines 1–3).

2. Please add this statement to the manuscript's Competing Interests: "AS is an Academic Editor on PLOS Medicine's editorial board."

**Answer:** This statement has been added in the revised version (p. 3, line 70).

3. Abstract:
a. Please report your abstract according to PRISMA for abstracts, following the PLOS Medicine abstract structure (Background, Methods and Findings, Conclusions) <http://www.plosmedicine.org/article/info:doi/10.1371/journal.pmed.1001419>.

**Answer:** The abstract has been revised according to the PRISMA 2020 Abstract Checklist [1], and structured under Background, Methods and findings, and Conclusions following the Journal’s guidelines (pp. 5–6).

b. Please combine the design, data sources, eligibility criteria and results sections into one section, “Methods and findings”.

**Answer:** Correction has been made in the revised version (pp. 5–6).

c. Please provide synthesis/appraisal methods in the abstract.

**Answer:** The methods for evidence synthesis and quality appraisal have been added in the revised version (p. 5, lines 102–106).

d. Please ensure that all numbers presented in the abstract are present and identical to numbers presented in the main manuscript text.

**Answer:** We have confirmed that all numbers presented in the abstract are present and identical to numbers presented in the main manuscript.

e. Please include the actual amounts or percentages of relevant outcomes, not just risk ratios.

**Answer:** Correction has been made in the revised version (p. 6, lines 111–119).

f. Please quantify the main results (with *p* values in addition to 95% CI).

**Answer:** Correction has been made in the revised version (p. 6, lines 111–119).

g. In the last sentence of the Abstract Methods and Findings section, please describe the main limitation(s) of the study's methodology.

**Answer:** Correction has been made in the revised version (p. 6, lines 126–129).

4. Abstract summary - At this stage, we ask that you reformat your non-technical Author Summary. The Author Summary should immediately follow the Abstract in your revised manuscript. This text is subject to editorial change and should be distinct from the scientific abstract. The summary should be accessible to a wide audience that includes both scientists and non-scientists. Please see our author guidelines for more information: <https://journals.plos.org/plosmedicine/s/revising-your-manuscript#loc-author-summary>.

**Answer:** The Author Summary has been revised following the Journal’s guidelines (pp. 7–8).

5. Please add the following statement, or similar, to the Methods: "This study is reported as per the Preferred Reporting Items for Systematic Reviews and Meta-Analyses (PRISMA) guideline (S Checklist)."

**Answer:** This statement has been added in the revised version (p. 10, lines 212–213).

6. Thank you for providing your PRISMA checklist. Please replace the page numbers with paragraph numbers per section (e.g., "Methods, paragraph 1"), since the page numbers of the final published paper may be different from the page numbers in the current manuscript.

**Answer:** Correction has been made in the revised version (S1 PRISMA Checklist).

7. Please include *p*-values in your results section and tables.

**Answer:** We have added *p*-values in the Results section (pp. 24–28, lines 473–576). In Tables 1 and 2 (pp. 17–20), however, we found it difficult to include *p*-values, as the criteria used to categorize the evidence level include both the meta-analysis summary results and the results of the most precise study (that is, there would be two types of *p*-values). As specified in Tables 1 and 2, two of the grading criteria are whether 95% confidence interval of the pooled estimate or the most precise study excludes the null, which could be used as a proxy for *p*-values (i.e., whether *p*-value < 0.05). The exact *p*-values for outcomes with the highest levels of evidence (consistent or highly suggestive) have been provided in the Results section, and *p*-values for all outcomes are provided in S1 Appendix.

8. Please refer to high income countries rather than "Western" countries.

**Answer:** Correction has been made in the revised version (p. 9, lines 178–179).

9. For your figures, please do the following:
a. Please indicate in the figure caption the meaning of the bars and whiskers.

**Answer:** The meaning of squares and horizontal lines in the forest plots (Figs 3 and 4, and eFigs 3-5 in S1 Appendix) has been added in their respective figure captions (p. 23, lines 447–449; p. 24, 464–466).

b. Please provide titles and legends for all figures (including those in Supporting Information files).

**Answer:** The titles and legends for all figures (including those in Supporting Information files that are cited in the manuscript) have been added in the revised version (pp. 13–14, lines 289–295; p. 21, lines 397–398; p. 22, lines 413–415; pp. 23–24, lines 436–471).

10. Please reformat Ref #6 ,10, 11, 17, 24, 43, 44, 47,52, 55, 60, 61, 63, 65, 66, 69. These should have 6 names listed before “et al.” Please use the "Vancouver" style for reference formatting, and see our website for other reference guidelines <https://journals.plos.org/plosmedicine/s/submission-guidelines#loc-references>.

**Answer:** We have checked and reformatted all the references per the Journal’s guidelines in the revised version (pp. 37–53).

**Reviewer 1**

1. The presentation is confusing. It would be better organized under organ system with both beneficial and harmful effects discussed under the same subheading. Observational studies and RCTs should also be described for each subheading and stated separately. As it currently reads, the effects of hormones are both beneficial and harmful for Alzheimer's Disease, for example. The reader must therefore toggle back to the 'benefits' section to appreciate that the authors are probably indicating that combined EPT (page 15) is harmful but ET is beneficial.

**Answer:** The Results section has been reorganized by organ system, and the results from RCTs and observational studies are presented separately (pp. 24–28, lines 473–576).

2. Page 14: Under Neoplasms, it should be specified that women with a uterus taking ET alone had a higher risk of endometrial cancer. If this is not the case based on the umbrella data, then discussion needs to include the fact that more recent regimens of EPT have been fairly carefully studied and several of them significantly reduce the risk of endometrial cancer compared to background risk with not therapy. This latter point is reflected in Table 1 but not in the text.

**Answer:** In the revised version, we have indicated that “In women with a uterus, ET was associated with an increased risk of endometrial cancer…” (p. 25, lines 495–497). Based on our umbrella review data, there was suggestive evidence from RCTs that EPT was associated with a lower risk of endometrial cancer, and this was indicated in Table 1 (pp. 17–18) and described in the eResults in S1 Appendix.

3. In the discussion on page 18, the authors describe an overall beneficial effect of hormone therapy on women with pre-existing breast cancer. This is very concerning, as there is some very clear evidence of publication bias in this area, with at least one major trial being stopped early for harm (<https://www.ncbi.nlm.nih.gov/pmc/articles/PMC338128/>). It is simply not known if other studies that have not been published had similar findings. It is extremely important to point out this caveat, as clinicians often use data from meta-analyses to inform their prescribing. Breast cancer is currently an absolute contraindication to hormone therapy in the United States, and this reviewer does not know if that is also true in other countries. The author is also directed towards the recent publication by Chlebowski et al, containing some of the most comprehensive follow-up of women with breast cancer from the WHI trial (<https://jamanetwork.com/journals/jama/fullarticle/2768806>), which demonstrated a neutral effect on overall mortality with relatively short-term use of hormones. Moreover, the authors are further directed to the 2010 Chlebowski publication, which contained more ominous data on breast cancer and mortality from a shorter follow-up period (<https://jamanetwork.com/journals/jama/fullarticle/186747>). Because these data speak to the possible earlier appearance of cancers and higher mortality in the earlier years of follow-up, it is especially clinically relevant to advise caution in interpretation of the authors' umbrella analysis.

**Answer:** We would like to thank the Reviewer for raising this important issue. We agree that publication bias would be one of the major concerns in this area. In our analysis of the observational data on breast cancer survival, we also found indication of small-study effects (i.e., smaller studies tended to report more pronounced effects than larger studies). This potential bias warrants particular caution when interpreting the results. In the revised version, to further emphasize the potential caveats, we have discussed the two early stopped trials, that is, the HABITS trial (hormonal replacement therapy after breast cancer – is it safe?) and the Stockholm trial, and current guidelines regarding the use of MHT in breast cancer survivors (p. 31, lines 634–648). We have now also discussed the results on breast cancer mortality from the WHI trials (p. 31, lines 651–653).

4. Confusion is further exacerbated by the use of the term MHT, which could mean either EPT or ET. MHT does not appear to be defined and since it adds to the confusion of this relatively complex manuscript, suggest that ET+EPT be used when referring to both types of hormone therapy and that MHT be dropped.

**Answer:** We have now defined MHT in the revised version and hope that this will clarify the confusion of terms as noted by the reviewer (p. 15, lines 327–331).

5. It would be helpful to organize the presentation of results in terms of relative importance (for example, breast cancer might be most appropriate to discuss first, as it likely has the largest effect on morbidity and mortality). At the very least, the ordering of the results should parallel the ordering of the discussion.

**Answer:** The Results section has been reorganized according to the relative importance of the outcomes (pp. 24–28, lines 473–576), and made consistent with the ordering of the Discussion section.

6. On pages 18-19, the authors are to be congratulated for a concise and accurate summary of the data to date on hormone therapy and cardiovascular disease. Further on, on page 19, the authors describe the ELITE trial and its potentially beneficial findings on vascular effects of hormones in recently menopausal women. The KEEPS Trial, which was a multicenter clinical trial and found no such benefit, should also be cited for balance (<https://pubmed.ncbi.nlm.nih.gov/25069991/>).

**Answer:** The Kronos Early Estrogen Prevention Study (KEEPS) has been added and discussed in the revised version (p. 33, lines 693–697).

7. Why does Figure 3A NOT include breast cancer under Neoplasms?

**Answer:** Figure 3A (named as “Figure 3” in the revised version) summarizes the outcomes with consistent or highly suggestive evidence from RCTs. As there was only suggestive evidence on breast cancer (Table 1; p. 17), breast cancer was not included in Figure 3A. Instead, the results were described in the eResults in S1 Appendix, and discussed in the Discussion section (pp. 31–32, lines 650–658).

**Reviewer 2**

1. The data analysis section mentions that data were grouped by primary or secondary prevention, but the summary of results does not make this distinction. Please discuss any relevant differences (or similarities).

**Answer:** As there has been some confusion about the definition of primary and secondary prevention [2], based on the data available, we defined primary prevention as reducing the risk of occurrence of a disease among individuals who do not have that disease at the beginning of follow-up, and secondary prevention as reducing the severity or progression of a disease among individuals who already have that disease or the risk of recurrence among individuals who have a history of that disease (p. 15, lines 332–337).

We have summarized the evidence for all the disease outcomes (both primary and secondary prevention) in Tables 1 and 2 (pp. 17–20), which has been indicated in the Results section (p. 22, lines 422–424). For secondary prevention, most outcomes fell into the ‘insufficient’ evidence level, probably because of limited data available (eTables 9 and 15 in S1 Appendix). Outcomes with consistent or highly suggestive evidence were described in the manuscript (pp. 24–28, lines 473–576), and outcomes with other evidence levels were described in the eResults in S1 Appendix.

2. The introduction mentions study quality as a rationale for conducting the study, and Figure 2 displays relevant results, but the results regarding this are not summarized or discussed in the text.

**Answer:** We have now moved the description of the quality of included systematic reviews from Supporting Information file to the manuscript (pp. 21–22, lines 400–408).

3. It would be useful to indicate whether results from RCTs and observational studies tended to be consistent.

**Answer:** We have reorganized the Results section by organ system as suggested by Reviewer 1 (pp. 24–28, lines 473–576), and therefore overlapping outcomes between RCTs and observational studies have been presented under the same category, which enables direct comparison (consistent or conflicting) between RCTs and observational studies for these outcomes. The important discrepancies between RCTs and observational studies are further discussed in the Discussion section (including breast cancer, coronary heart disease and all-cause mortality; pp. 30–34, lines 614–708).

4. The introduction and the discussion mention the timing hypothesis, but results from the analysis do not address this directly - please clarify whether this was not possible given the nature of the data, or whether additional analyses could be done to address this question.

**Answer:** We would like to thank the Reviewer for raising this important comment. To avoid duplicate information, we would like to direct the Reviewer to our responses to the third comment by Reviewer 4.

5. p. 10: please provide a brief description of Egger's regression.

**Answer:** A brief description of Egger’s regression has been added in the revised version (p. 15, lines 317–318).

6. p. 12: please indicate in the text what is meant by "consistent or highly suggestive evidence".

**Answer:** The meaning of consistent or highly suggestive evidence has been added in the revised version (p. 22, lines 426–429).

7. p. 13: regarding breast cancer survival, it's not clear whether this is associated with pre-diagnosis MHT use or post-diagnosis MHT use (or both).

**Answer:** Correction has been made in the revised version (p. 25, lines 482–486).

8. p. 15: what is meant by "no qualitative difference" regarding breast cancer risk comparing ET and EPT?

**Answer:** A qualitative difference represents that effects do not point in the same direction, while a quantitative difference represents that effects differ in magnitude [3-5]. The meaning of qualitative difference has been added in the Methods section (p. 15, lines 327–328).

9. pp. 20-21: please explain what is meant by "the random-effects assumption is the only plausible match to the underlying effect distribution." Also, it seems that this would be an analytic approach rather than an assumption.

**Answer:** The fixed-effect model assumes that all studies in the meta-analysis share a common true effect size, whereas the random-effects model assumes that there is a distribution of true effect sizes (p. 35, lines 730–732). As studies included in meta-analysis are usually gathered from the published literature, these studies tend to differ in many ways, such as population (e.g., age, ethnicity) and intervention (e.g., formulation, dose, duration). In other words, the underlying true effects in the studies can be reasonably assumed to differ (quantitatively). Therefore, the random-effects model is the only plausible match to this underlying effect distribution.

**Reviewer 3**

1. Please provide line numbers of the manuscript in the future.

**Answer:** Line numbers have been added in the revised version.

2. Page 7: "…Often, these guidelines incorporated systematic reviews and meta-analyses as a key evidence support for the recommendations, but with little attention to their quality and scientific validity…" Is there any evidence of such claim?

**Answer:** Supporting references have now been cited in the revised version (p. 9, lines 190–191).

3. Page 8: in the "Literature Search and Eligibility Criteria", I am wondering why PubMed was not included as it is a major database for medical literature.

**Answer:** As Medline covers around 98% of PubMed (<https://kemh.libguides.com/library/search_tips/faqs/difference_between_pubmed_medline_embase>), it would be unlikely to expect noticeable differences in the search results between them.

4. Page 9: "… We performed an updated search on December 17, 2020 for studies published since November 27, 2017…" I don't understand why studies from Nov 2017 to Dec 2020 were not included in this umbrella analysis. Why the original search was only from inception to 2017? I see the details of the updated search in Sup 2. However, I still don't understand why not including the latest SR/MA in the analyses.

**Answer:** As described in the Methods section (p. 13, lines 271–287), during data extraction we devoted extensive efforts to obtaining and validating the data for individual studies in each meta-analysis, thus making this project very time-consuming. The data extraction and quality appraisal alone took approximately one and a half years to complete. Given the data extraction protocols, we estimated that inclusion of studies published after November 27, 2017 may take a minimum of 12 months, during which time we anticipate that additional reviews might have been published on the topic, which may also need to be integrated into the umbrella review. This in reality will make the process an unending one. For this reason, we decided to include only studies published up to November 26, 2017 as per our original protocol, but at the same time, perform an updated search and provide a general summary of studies published after November 26, 2017. This updated searches and the summary provided in eTable in S2 Appendix are only explorative and highlight the impact the new systematic reviews would have on the current umbrella review had they been integrated into the review. This evidence analyses will be taken into account in the future when the current umbrella review is updated. Furthermore, as current guidelines [6-9] were published between 2015–2017, we also considered that the evidence generated from our umbrella review will be useful in evaluating current guidelines and in planning for guideline development in the future.

5. Page 10: "…A subgroup analysis by MHT type was conducted to assess whether the effect varied qualitatively between ET and EPT…" I figured out what you mean by qualitative difference latter in the paper. But it would be helpful if you could explain what are the qualitative differences of effects between ET and EPT here. Also, if you conducted subgroup analysis by MHT type, I think it's more common to examine quantitative differences between the effects by therapy type.

**Answer:** A qualitative difference represents that effects do not point in the same direction, while a quantitative difference represents that effects differ in magnitude [3-5]. The meaning of qualitative difference has been added in the Methods section (p. 15, lines 327–328). Indeed, quantitative differences are commonly to be expected, whereas unanticipated qualitive differences are rare [3-5]. From a clinical point of view, identification of substantial quantitative differences can be as important as qualitative differences, which helps individualize treatment [3]. In the umbrella review, the main focus was to summarize the evidence on the effects of MHT compared to *no MHT* on diverse health outcomes in women, and try to answer a more *global* question – what is the *average* effect of MHT compared to *no MHT* on each outcome? – so as to present an overview of the evidence landscape regarding the effects of MHT. To avoid duplicate information, we would also like to direct the Reviewer to our response to the first comment raised by Reviewer 4 for more information.

6. Why did you exclude “Not largest or latest systematic review”? This is not mentioned in the method section.

**Answer:** More information about the review methods has been added in the revised version (pp. 11–12, lines 235–247).

7. Page 11: in the "Results" section, I did not see the results of the quality of studies in these SR/MA. Even some descriptions/summaries could be very useful.

**Answer:** We have moved the description of the quality of included systematic reviews from Supporting Information file to the manuscript (pp. 21–22, lines 400–408).

8. Page 15: when you show the RR and its 95% CI, it's also useful to show the number of studies these RR estimates are based (as you did for breast cancer).

**Answer:** The number of studies and participants for each outcome has been added in the revised version (pp. 24–28, lines 473–576).

9. Page 20: "…First, a fixed-effect meta-analysis model, instead of a random-effects model, was often used even when heterogeneity was present or anticipated *a priori*. By gathering studies from the published literature, the random-effects assumption is the only plausible match to the underlying effect distribution…" This is not true. Although the distribution of the true effect is usually assumed to be normal which is in line with the assumption of random-effect model. However, fixed-effect model is often preferred over random-effect model to adjust for bias due to unobserved covariates (e.g., different unobserved characteristics of the studies), especially when there is enough sample from each study to estimate the fixed effects of each study. I think that is the main reason why you found many studies using the fixed-effect model rather than random effect model.

**Answer:** By “fixed-effect model”, we are referring to fixed-effects meta-analysis (i.e., with no heterogeneity) rather than to “fixed effects of each study” as the reviewer describes. Fixed effects of each study would be estimable only if each study contributed many different estimates, which is very rarely the case in meta-analysis and was not the case in the meta-analyses we included. Statistical theory [10] shows that the fixed-effect model provides valid inference when either (i) there is no heterogeneity across studies and the meta-analyst intends to make inferences about the underlying population of studies from which the meta-analyzed studies are drawn; or (ii) there may be heterogeneity across studies, but the meta-analyst only intends to summarize the effects of the sampled studies, without drawing inferences to the heterogeneous distribution at all. The former goal is much more standard for meta-analysis, and the variance of the fixed-effect model will typically be too small for this purpose when there is in fact heterogeneity [10].

10. Page 21: "Second, …, Under the random-effects model, the summary effect is an estimate of the mean of a distribution of true effects across studies…" This is not accurate either. The interpretation of estimate from random-effect model should be the mean of the distribution of the true effect conditional on the covariates (if any) in the model and on the random effects of the studies (i.e., the RE is treated as a covariate in the model). So, the effect from a random-effect model is still conditional effect rather than marginal effect.

**Answer:** It is true that, as a general principle, coefficient estimates from general linear models (of which meta-analysis is a special case) must be interpreted conditional on other coefficients in the model, including both fixed and random coefficients. With respect to fixed coefficients, these arise in meta-analysis only in meta-regression models, and none of the included meta-analyses were meta-regressions. With respect to studies’ random effects, the coefficients in general linear models that specifically use identity link functions (which is the case in meta-analysis) are in fact “collapsible” [11], meaning that the coefficients conditional on random effects (and any fixed covariates) are the same as coefficients that are marginal with respect to the random effects (and condition only on fixed covariates). This is because the mean of the random effects is constrained to be 0.

11. Page 21: "…Third, investigators often relied heavily on statistical methods (e.g., Egger's test) to deal with publication bias. To address publication bias, there is need to obtain a representative sample of studies on a topic (e.g., through comprehensive literature searches) …" Egger's regression, along with the funnel plot, is often used to detect publication bias. To deal with the publication bias, finding studies with representative sample is ideal but may not be realistic. One can practically use the "trim-and-fill" method to alleviate the effect of publication bias. Reference: Shi, L., & Lin, L. (2019). The trim-and-fill method for publication bias: Practical guidelines and recommendations based on a large database of meta-analyses. Medicine, 98(23). <https://doi.org/10.1097/MD.0000000000015987>

**Answer:** We would like to thank the Reviewer for raising this important and interesting discussion. We fully agree that obtaining a representative sample of studies is ideal, but in many cases difficult. Many statistical methods, including the trim-and-fill method, have been proposed to deal with publication bias in meta-analysis [12]. However, because the mechanisms of publication bias are quite complex and available statistical methods are often based on certain strong assumptions, these methods are mainly used as sensitivity analyses rather than as confirmatory tests [12,13]. Therefore, to address publication bias, despite the difficulties and challenges in obtaining a representative sample, attempts (e.g., comprehensive literature search) are still strongly recommended [14], in conjunction with application of the statistical methods.

**Reviewer 4**

1. Overall, it is becoming increasingly clear that the type (ET vs EPT), route (transdermal, oral), formulation (oral conjugated equine estrogen, estradiol, MPA, micronized progesterone), dose and duration of MHT impacts its outcomes differently. This needs to be discussed in greater detail in the manuscript and an attempt to review and present findings by these characteristics should be made for each outcome (more than a line in the limitations). Page 14/15 for neoplasms is a good example. For example, much of the data on risk of meningioma is based on older data using oral higher dose estrogens and therefore doesn't provide guidance on the use of transdermal or lower dose MHT and their relationship with meningioma risk. Similarly, breast cancer risk may be related to the type, route and duration of MHT (progesterone possibly less risk than MPA, etc.) so discussion of these different relationships should be included. I recognize that this level of detail may be difficult to obtain in an umbrella review, but a discussion about these factors should accompany the results and discussion.

**Answer:** We would like to thank the Reviewer for raising this important comment. We fully agree that the effects of MHT can vary by different treatment factors, including MHT type, formulation, dose, duration and route of administration, and considering the different effects of MHT by these factors is important in the evaluation of the trade-off between benefits and harms associated with MHT use in women. In our protocol, we planned to perform subgroup analyses by type of MHT and route of administration, but we could only conduct analyses by type of MHT, because it was not possible to obtain relevant data on MHT formulation, dose, duration and route of administration. We have now added this limitation in the Abstract (p. 6, lines 126–129) and in the Discussion (pp. 29–30, lines 603–612).

2. In clinical practice, many are now using estradiol and micronized progesterone so to provide the most clinical utility, at a minimum the results could display findings based on these formulations.

**Answer:** We agree that it would be of clinical importance to present results for estradiol and micronized progesterone. As discussed in our response to the Reviewer’s first comment, unfortunately, the available data did not allow us to perform such an analysis.

3. Additionally, results for certain outcomes differ based on the timing of initiation of MHT (age 50 - 59, 60 - 69, 70-79). Specifically, these outcomes including those related to diseases of the circulatory system as well as dementia. Therefore, in my opinion, results for these outcomes should be displayed by age of initiation (this would impact results on page 13,14 and related tables and figures). These post hoc analyses have provided scientific theory to guide more recent RCTs including ELITE and KEEPS (the latter is not discussed in the manuscript but should). Current guidelines (such as NAMS) have incorporated these findings to demonstrate the low cardiovascular risk in women < age 60 (without known disease) who are symptomatic with vasomotor symptoms and considering initiating MHT for quality-of-life relieving therapy. To my knowledge, guidelines have not recommended initiation of MHT for cardiovascular benefit or primary prevention at least in the U.S. (in part likely related to the concerns discussed on page 19). On page 19, it would be of benefit to rephrase the sentence starting with "current guidelines and reviews…" since those two things are different. There is a difference between recommending MHT initiation for VMS and reassuring women that CVD risk is low, vs initiating MHT for cardiovascular benefit. I fear if this difference is not made clearer in the discussion than clinicians may misinterpret the findings and withhold quality of life benefiting therapy for symptomatic menopausal women.

**Answer:** We would like to thank the Reviewer again for raising another important, but controversial issue (i.e., the timing hypothesis). As we discussed in the manuscript (pp. 32–34, lines 660–708), the current available clinical data in support of this hypothesis are found to be only suggestive.

Although several previous meta-analyses [15-18] have made efforts to investigate the effects of MHT on coronary heart disease and all-cause mortality in young group of women (i.e., < 60 years or within 10 years of menopause), we have discussed in our manuscript (pp. 32–33, lines 672–685), however, that these subgroup analysis results have to be interpreted cautiously. For the reason of the current controversy, we did not conduct subgroup analyses by age of women for coronary heart disease and all-cause mortality. There were only limited data available for dementia (eTables 6 and 12 in S1 Appendix). We have therefore provided a general overview of the timing hypothesis and discussed the potential limitations in previous analyses.

In the revised version, we have discussed the Kronos Early Estrogen Prevention Study (KEEPS) trial (p. 33, lines 693–697), and rephrased the sentence (p. 32, lines 676–679).

We have also discussed the current practice guidelines and indicated that MHT is mainly suggested in menopausal women with low cardiovascular risks and no contradictions, for treatment of bothersome vasomotor symptoms and prevention of bone loss or fracture for those at elevated risk, not for primary prevention of cardiovascular diseases (pp. 33–34, lines 703–707).

4. P 4 - Consider including an additional position statement or guideline in the second bullet point to highlight examples that recommendations are not consistent.

**Answer:** Following the Journal’s guidelines on Author Summary which suggest a non-technical summary, some bullet points (including the second one indicated by the Reviewer) in the original manuscript have been removed. The revised version of Author Summary is available on pages 7–8.

5. P 4 - For the second bullet of the 'what this study adds' I would recommend considering changing the word 'tentative' - perhaps to 'would benefit from further attention' or something similar. The end of the sentence needs to clarify that the authors are suggesting the clinical guidelines be re-examined only for this particular outcome because as it reads now some may believe they are suggesting all outcomes need to be re-evaluated.

**Answer:** Correction has been made in the revised version (p. 8, lines 164–165).

6. P 4 - The last bullet in the section 'what this study adds' would benefit from modification. The first part of the sentence and the last part of the sentence run counter to each other. I would recommend considering changing the word 'credibility' to 'scientific strength' or something similar. I would think the requirement to assess credibility should fall to the researchers participating in the systemic reviews/research, and the peer reviewers and journals publishing them, as well as the societies that are using them to guide position statements. It is the duty of clinicians to use the highest strength EBM to guide their clinical decision making when that EBM exists. (This should be adjusted in the conclusion as well. You may simply delete the last sentence of your conclusions.)

**Answer:** Correction has been made in the revised version (p. 8, lines 166–167; p. 35, lines 750–752).

7. P 14 - Genitourinary syndrome: findings only reported for ET and increased risk of endometrial hyperplasia. Information about progestogens to reduce risk of endometrial hyperplasia should be presented here as well.

**Answer:** Based on the available data, we did not find evidence that EPT reduces the risk of endometrial hyperplasia (eTable 8 in S1 Appendix). The results therefore were not described in the main manuscript, but summarized in Table 1 (p. 17). Indeed, whether EPT could protect against endometrial hyperplasia would depend on whether the effects of estrogen are appropriately opposed by progestin.

8. The main goal of treatment for symptomatic menopausal women (vasomotor symptoms, GSM) is to relieve their menopausal symptoms. Your umbrella review found good evidence that MHT is effective at relieving these symptoms. Without acknowledging the limits of the data availability (on dose, formulations, route, age of initiation) and quality for other outcomes at the start of the discussion clinicians may misinterpret your findings and not accurately "consider the full range of effects, along with patient's values and preferences" as you state in your conclusion.

**Answer:** In the revised version, we have indicated the limitations of this umbrella review in the Abstract (p. 6, lines 126–129) and in the Discussion (pp. 29–30, lines 595–612; following the summary of key findings).

**References**

1.	Page MJ, McKenzie JE, Bossuyt PM, Boutron I, Hoffmann TC, Mulrow CD, et al. The PRISMA 2020 statement: An updated guideline for reporting systematic reviews. PLoS Med. 2021;18(3):e1003583.

2.	Lucan SC. Coronary artery disease prevention: as easy as 1, 2, 3? Am Fam Physician. 2010;82(10):1167.

3.	Oxman AD, Guyatt GH. A consumer's guide to subgroup analyses. Ann Intern Med. 1992;116(1):78-84.

4.	Yusuf S, Collins R, Peto R. Why do we need some large, simple randomized trials? Stat Med. 1984;3(4):409-22.

5.	Yusuf S, Wittes J, Probstfield J, Tyroler HA. Analysis and interpretation of treatment effects in subgroups of patients in randomized clinical trials. JAMA. 1991;266(1):93-8.

6.	The 2017 hormone therapy position statement of The North American Menopause Society. Menopause. 2017;24(7):728-53.

7.	Baber RJ, Panay N, Fenton A. 2016 IMS Recommendations on women's midlife health and menopause hormone therapy. Climacteric. 2016;19(2):109-50.

8.	de Villiers TJ, Hall JE, Pinkerton JV, Pérez SC, Rees M, Yang C, et al. Revised global consensus statement on menopausal hormone therapy. Maturitas. 2016;91:153-5.

9.	Stuenkel CA, Davis SR, Gompel A, Lumsden MA, Murad MH, Pinkerton JV, et al. Treatment of Symptoms of the Menopause: An Endocrine Society Clinical Practice Guideline. J Clin Endocrinol Metab. 2015;100(11):3975-4011.

10.	Rice K, Higgins JP, Lumley T. A re‐evaluation of fixed effect (s) meta‐analysis. J R Stat Soc Ser A Stat Soc. 2018;181(1):205-27.

11.	Ritz J, Spiegelman D. Equivalence of conditional and marginal regression models for clustered and longitudinal data. Stat Methods Med Res. 2004;13(4):309-23.

12.	Song F, Parekh S, Hooper L, Loke YK, Ryder J, Sutton AJ, et al. Dissemination and publication of research findings: an updated review of related biases. Health Technol Assess. 2010;14(8):iii, ix-xi, 1-193.

13.	McShane BB, Böckenholt U, Hansen KT. Adjusting for Publication Bias in Meta-Analysis: An Evaluation of Selection Methods and Some Cautionary Notes. Perspect Psychol Sci. 2016;11(5):730-49.

14.	Shea BJ, Reeves BC, Wells G, Thuku M, Hamel C, Moran J, et al. AMSTAR 2: a critical appraisal tool for systematic reviews that include randomised or non-randomised studies of healthcare interventions, or both. BMJ. 2017;358:j4008.

15.	Salpeter SR, Cheng J, Thabane L, Buckley NS, Salpeter EE. Bayesian meta-analysis of hormone therapy and mortality in younger postmenopausal women. Am J Med. 2009;122(11):1016-22.e1.

16.	Salpeter SR, Walsh JM, Greyber E, Ormiston TM, Salpeter EE. Mortality associated with hormone replacement therapy in younger and older women: a meta-analysis. J Gen Intern Med. 2004;19(7):791-804.

17.	Salpeter SR, Walsh JM, Greyber E, Salpeter EE. Brief report: Coronary heart disease events associated with hormone therapy in younger and older women. A meta-analysis. J Gen Intern Med. 2006;21(4):363-6.

18.	Boardman HM, Hartley L, Eisinga A, Main C, Roque i Figuls M, Bonfill Cosp X, et al. Hormone therapy for preventing cardiovascular disease in post-menopausal women. Cochrane Database Syst Rev. 2015;2015(3):CD002229.
