# Supplementary material for: Menopausal hormone therapy and women’s health: An umbrella review
Source: PLoS Med. 2021 Aug 2;18(8):e1003731. doi: 10.1371/journal.pmed.1003731 (PMC8366967; doi:10.1371/journal.pmed.1003731)
Supplement: Attachment — Submitted filename: Response to Reviewers.docx [file pmed.1003731.s009.docx]

*(The location of contents indicated in the ‘Response to Reviewers’ refers to the ‘Revised Article with Changes Highlighted’)*

**Comments from editors**

1. Please ensure that the paper adheres to the PLOS Data Availability Policy (see <http://journals.plos.org/plosmedicine/s/data-availability>).

**Answer:** We have made the dataset publicly available at the Open Science Framework, and provided the digital object identifier (DOI) for the dataset (p. 2, lines 44–47).

**Reviewer 3**

1. I now understand why you did not include SR/MA published after 2017, but I think it's better if you could include that as a limitation of the study in the limitation section.

**Answer:** We have added this limitation in the revised version (p. 29, lines 596-599).

2. I agree that the model is "collapsible" if identity link function is used. However, many systematic reviews report RR/ORs, which usually requires log transformation in the meta-analysis. So, in general, you can only interpret the coefficients as conditional effects.

**Answer:** When there are no covariates in the model and the random intercept mean is fixed to 0, as is the case in all of these meta-analyses, the use of the log transformation does not induce a conditional interpretation. This is because the transformation is applied to the effect sizes themselves (analogously to log-transforming the outcome in standard OLS regression, or indeed using any other transformation), not to the expectation of the effect sizes (as in GLMs). It is the latter kind of transformation that can induce non-collapsibility if there are covariates in the model. The use of the log transformation simply means that what is being estimated is the (marginal) mean of the effect sizes on the log-RR scale rather than the mean on the RR scale.

**Reviewer 4**

1. Line 638 - 642 is incorrect. Current guidelines do not recommend MHT for vasomotor symptoms in breast cancer survivors. There is guidance regarding low dose vaginal estrogen in this population. This line needs to be updated and it needs to be made clear the difference in recommendations between systemic HT and low dose vaginal estrogen.

**Answer:** Correction has been made in the revised version (p. 31, lines 644–648), and a new reference “*Management of genitourinary syndrome of menopause in women with or at high risk for breast cancer: consensus recommendations from The North American Menopause Society and The International Society for the Study of Women’s Sexual Health (2018)”* has been added (ref. 122, p. 52, lines 1115–1119).

2. I understand you can't include information about estradiol and micronized progesterone in detail in the paper due to lack of data, but you should at least point that out since clinicians may be reviewing this umbrella review and should know that that data is not specifically about more common formulations.

**Answer:** Correction has been made in the revised version (p. 30, lines 608–609).

3. Line 162 page 8 is not fully correct. Current practice guidelines say that for women less than age 60 or within 10 years of their LMP that the benefits greatly outweigh the risks for symptomatic women, and DO Not recommend MHT for prevention of CHD or all-cause mortality. Instead of the conclusion you have here, I would summarize your findings as saying that more data is needed to evaluate the timing hypothesis (not reexamine the guidelines which are appropriately supporting treatment for low-risk symptomatic women).

**Answer:** Correction has been made in the revised version (p. 8, lines 162–164).

4. Line 510 - why is information on VMS included in the GU system paragraph?

**Answer:** We categorized outcomes according to the International Classification of Diseases, 11th Revision (ICD-11), and vasomotor symptoms (hot flushes and night sweats) were classified into the genitourinary system (<https://icd.who.int/browse11/l-m/en#/http://id.who.int/icd/entity/222407548>).

5. Line 520 - you need to specify what parameters of sexual function improved (desire, pain, orgasm)?

**Answer:** The definition of sexual function has been added in the revised version (p. 26, lines 521-522).
